# Gauging Radical Stabilization with Carbenes

**DOI:** 10.1002/anie.202206390

**Published:** 2022-08-04

**Authors:** Kevin Breitwieser, Hilke Bahmann, Robert Weiss, Dominik Munz

**Affiliations:** ^1^ Coordination Chemistry Saarland University Campus C4.1 66123 Saarbrücken Germany; ^2^ Physical and Theoretical Chemistry Saarland University Campus B2.2 66123 Saarbrücken Germany; ^3^ Organische Chemie Friedrich-Alexander-Universität (FAU) Erlangen-Nürnberg Henkestr. 42 91054 Erlangen Germany; ^4^ Inorganic and General Chemistry Friedrich-Alexander-Universität (FAU) Erlangen-Nürnberg Egerlandstr. 1 91058 Erlangen Germany

**Keywords:** Carbenes, Density Functional Calculations, Molecular Orbitals, *N*-Heterocyclic Carbene, Radicals

## Abstract

Carbenes, including *N*‐heterocyclic carbene (NHC) ligands, are used extensively to stabilize open‐shell transition metal complexes and organic radicals. Yet, it remains unknown, which carbene stabilizes a radical well and, thus, how to design radical‐stabilizing C‐donor ligands. With the large variety of C‐donor ligands experimentally investigated and their electronic properties established, we report herein their radical‐stabilizing effect. We show that radical stabilization can be understood by a captodative frontier orbital description involving π‐donation to‐ and π‐donation from the carbenes. This picture sheds a new perspective on NHC chemistry, where π‐donor effects usually are assumed to be negligible. Further, it allows for the intuitive prediction of the thermodynamic stability of covalent radicals of main group‐ and transition metal carbene complexes, and the quantification of redox non‐innocence.

## Graphical abstract

Radical stabilization by carbenes is explored computationally. A donor/acceptor frontier orbital picture based on the allyl radical is presented, and allows one to understand and predict radical stabilization in organic radicals, carbene complexes of the transitions metals, as well as of s‐ and p‐block elements.

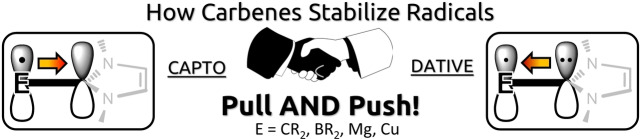

## Introduction

Persistent^[1]^ radicals are central to the chemistry of molecules and materials.^[2]^ This is due to their versatile and peculiar physico‐chemical characteristics, which are required in a plethora of applications. These range from synthetic‐ and macromolecular chemistry^[3]^ to medicine,^[4]^ non‐linear optics,^[5]^ energy storage,^[6]^ magnetism, sensoring,^[7]^ (semi‐)conductance,^[8]^ and spin‐ as well as nanoelectronics.^[9]^ Yet, carbon‐centered radicals are often only short‐lived and air‐sensitive. This renders them powerful intermediates for bond activation in synthetic chemistry.^[10]^ However, their high reactivity is also the major obstacle to bringing them to broad application in material science.^[11]^ The same is true for boron radicals^[12]^ and many open‐shell transition metal complexes.^[13]^

**Figure 1:**
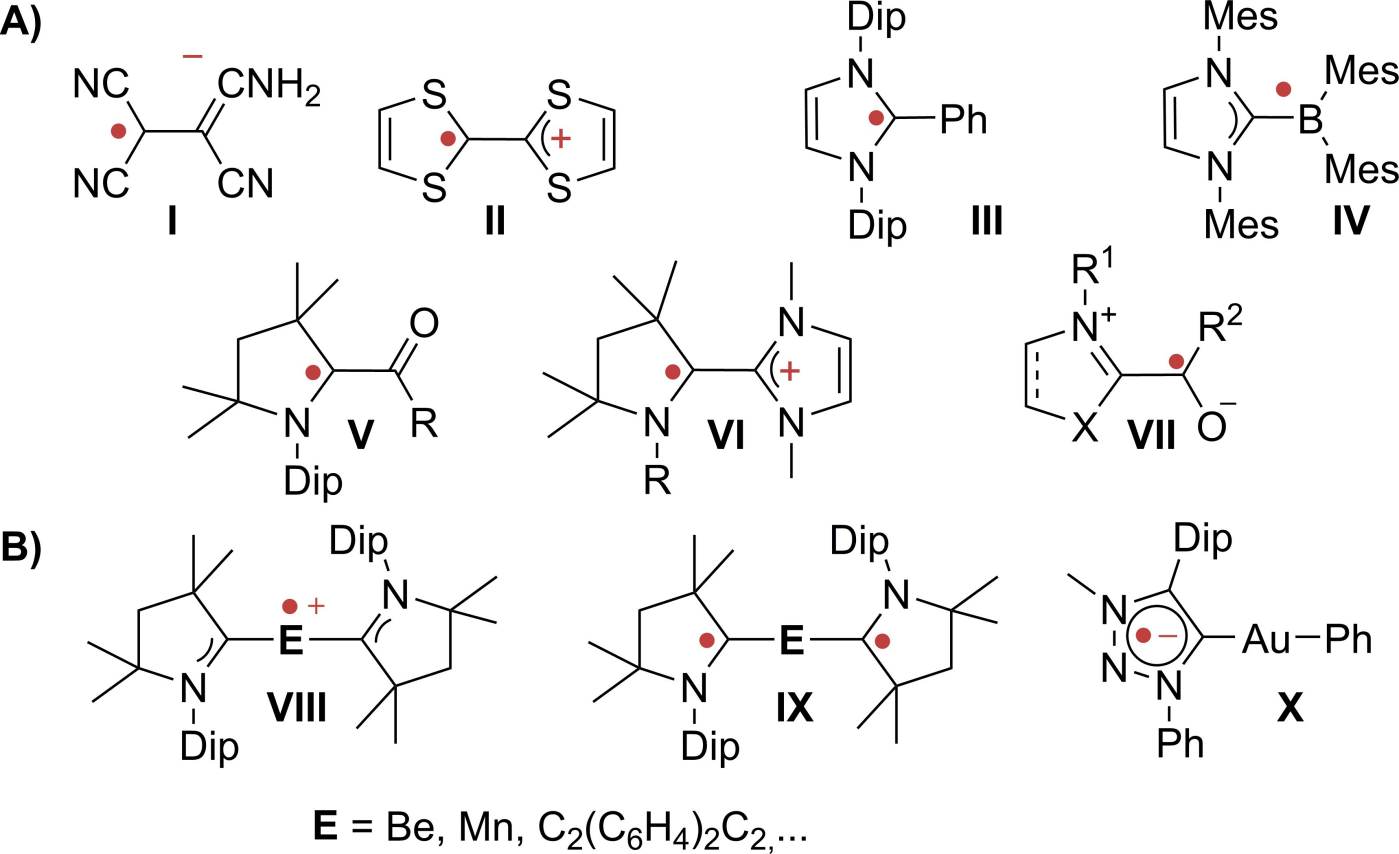
Organic carbene radicals (A) and redox‐non‐innocent carbene ligands (B).

Inspired by the rich chemistry of the tetracyanoethylene (TCNE) radical anion (Figure 1, **I**) and the tetrathiafulvalene (TTF) radical cation (Figure 1, **II**), carbenes brought new impetus to the field of “bottleable” radicals.^[14]^ For instance, *N*‐heterocyclic carbenes (NHCs)^[15]^ stabilize both formally C‐^[16]^ and B‐centered^[17]^ radicals as exemplified by **III** ^[18]^ and **IV**.^[19]^ In particular cyclic (alkyl)(amino)carbenes (CAACs) became popular,^[20]^ where carbonyl substituted CAAC radicals **V** are even air‐persistent.^[21]^

Radical **VI** and bridged congeners[^20g^, ^22^] proved useful in non‐linear optics and singlet‐fission^[23]^ and applications in batteries have been proposed.^[24]^ These cationic radicals are derived from electron‐rich olefins and related with Breslow's intermediates,^[25]^ which accordingly also emerged as transient single‐electron‐transfer reagents (**VII**).^[26]^ In fact, CAACs afford persistent radicals with elements across essentially the whole periodic table (**VIII**),[^16b^, ^27^] including even the *s*‐block metals.^[28]^ In many cases, they are redox‐noninnocent (**IX**), placing them among the most powerful redox‐active ligands reported to date, and highlighting the ambiguity to assigning the radical to one particular site. Radical stabilization by NHCs and CAACs is, inspired by the rich open‐shell chemistry of Fischer carbenes,^[29]^ commonly attributed to their π‐acidity. The latter can be assessed experimentally through the hetero‐NMR shifts of selenium and phosphinidene adducts, or through computation of their LUMO's energy.^[30]^ Whereas π‐donor capabilities of carbenes are commonly assumed to be negligible, such interactions have been proposed in 2004 by Cavallo, Nolan and colleagues,^[31]^ and discussed by Frison, Frenking and coworkers.^[32]^ Indeed, yet seemingly surprisingly, persistent radicals of mesoionic carbenes, commonly assumed to lack considerable π‐backbonding capabilities, emerged (**X**).^[33]^ Thus, and whereas the knowledge on how to thermodynamically stabilize radicals (*vide infra*) has progressed in the last decades, the comparative stability of carbene‐derived radicals is not understood.^[34]^ Neither is it known, to which extent the degree of steric protection, *viz*. kinetic stability, is crucial. Accordingly, most classes of carbenes have not yet been investigated in open‐shell chemistry.[^21^, ^26g^, ^35^] In fact, π‐electron rich C‐donors, also referred to as bent allenes, carbodicarbenes^[36]^ or carbodiphosphoranes^[37]^ remain vastly unexplored^[38]^ in this context. We elucidated selected aspects of the radical chemistry of carbenes in experimental and computational investigations. This included how carbenes control the (open‐shell) excited state properties of conjugated hydrocarbons[^23a^, ^39^] and how planarization^[40]^ is crucial to prevent disproportionation of their multi‐stage redox systems. Herein, we present a detailed and comprehensive study on the electronic structure of carbene‐derived radicals. We show how to understand and predict their thermodynamic stability by an intuitive perturbative frontier‐orbital description, which arguably is applicable to any covalent π‐radical.

## Results and Discussion

A radical's stability is quantified by the radical stabilization energy *RSE*, which is the enthalpy for isodesmic hydrogen atom transfer (*RSE*=Δ*H*).^[41]^ In case of conjugated radicals, the *RSE* is mostly controlled by the extended π‐system.^[42]^ Coote *et al*. corroborated computationally that the higher the delocalization (“dilution”) of spin density, the less reactive they are.^[43]^ Similarly, Paton and colleagues studied the connection between the *RSE* (maximum spin density, respectively) and kinetic protection introducing the radical stability score (*RSS*) as a measure for a radical's kinetic stability.^[44]^ These investigations were complemented by charge separation (R_2_C^+^−X^−.^ vs. R_2_C^−^−X^+.^)^[45]^ valence bond arguments,^[46]^ and comparable effects were found for boryl radicals.^[47]^ Note that the combination of two orbital interactions, *i.e*. captodative substitution or merostabilization by two substituents, is believed (yet by some disputed) to lead to particularly stable radicals.^[48]^

**Figure 2:**
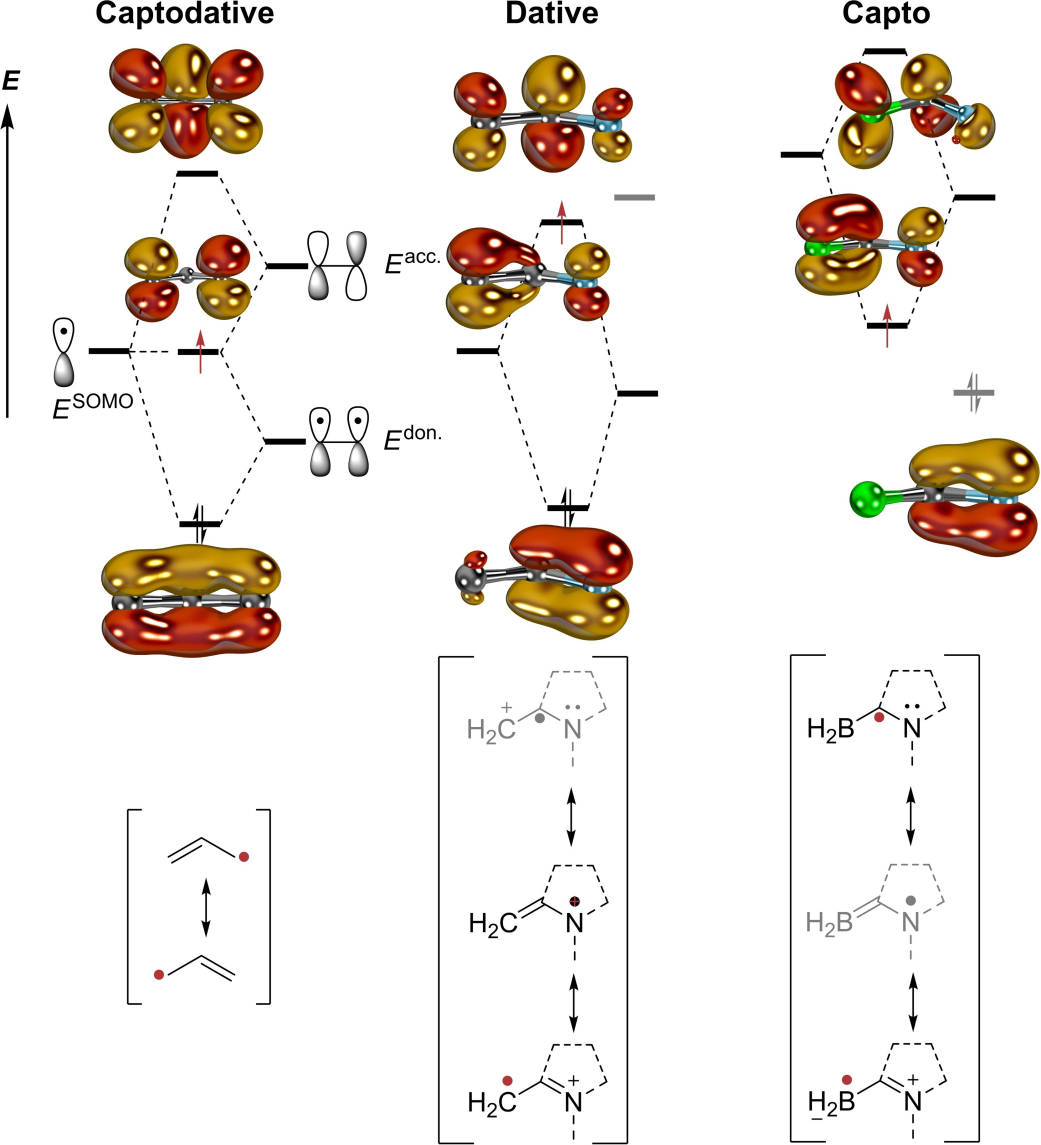
The electronic structure of the allyl radical (left, “captodative”), iminium‐stabilized, electrophilic C‐radicals (middle, “dative”), and nucleophilic B‐radicals (right, “capto”). Canonical MOs were computed with B3LYP, natural orbitals as obtained by CASSCF(3,3) calculations are given in Figure S1.

We propose to understand radical stabilization by carbenes using the textbook example of (hetero)allyl radicals (Figure 2). In case of carbene‐derived radicals, both the donor‐ as well as acceptor interaction occur in concert with only one substituent, *viz*. the carbene.^[49]^ In the case of the allyl radical, the SOMO of the ⋅CH_2_ fragment combines with both the ethenyl‐substituent's π‐ and π*‐orbital. Thereby, the interaction with the occupied π‐fragment orbital may be understood as donor stabilization, whereas combination with the vacant π*‐orbital represents the capto‐case. Replacing the ethenyl‐ by a cationic iminiumyl substituent (as is found in CAACs) lowers the energy of these frontier orbitals. Because nitrogen is more electronegative (*EN* ^Pauling^=3.0) than carbon (*EN* ^Pauling^=2.5),^[50]^ the energy level *E* ^don.^ is similar, yet lower than *E* ^SOMO^, and a predominantly **dative** radical stabilization results. In consequence, most spin‐density is expected at the terminal methylene group. Contrarily, in the formally zwitterionic boryl radicals, electropositive boron exhibits an electronegativity (*EN* ^Pauling^=2.0) similar to the one of late transition metals (*EN* ^Pauling^=1.7–2.4), thus elevating the energy *E* ^SOMO^ of the BH_2_‐fragment. This leads to stronger **capto**‐character and accumulation of spin density at both the boron‐ and the carbene carbon atom. In case of carbene ligands of transition metals, this picture coincides with strong π‐backbonding.

These considerations are equivalent to Salem's perturbative analysis of interacting π‐systems describing, *inter alia*, cycloaddition reactions.^[51]^ Assuming significant energy differences between the frontier orbitals (*E* ^HOMO^ and *E* ^LUMO^), an average interaction energy β between different atomic orbitals, and neglecting simultaneous three‐orbital mixing, the interaction energy Δ*E*_int_ between two fragments is given as follows [Eq. 1].^[51a]^

(1) $$\Delta E_{\mathrm{int}}\propto \frac{2{\left(\sum_{ij}c_{Ai}^{\mathrm{HOMO}}c_{Bj}^{\mathrm{LUMO}}\beta \right)}^{2}}{\left(E_{A}^{\mathrm{LUMO}}-E_{B}^{\mathrm{HOMO}}\right)}-\frac{2{\left(\sum_{ij}c_{Bi}^{\mathrm{HOMO}}c_{Aj}^{\mathrm{LUMO}}\beta \right)}^{2}}{\left(E_{B}^{\mathrm{LUMO}}-E_{A}^{\mathrm{HOMO}}\right)}\;$$

In case of radicals, we propose to adopt this relationship for the interaction of a π‐system with the SOMO of the radical fragment, which is in case of the allyl system ⋅CH_2_, and in case of the boryl radicals ⋅BH_2_. The atomic orbital coefficients *c* and resonance integrals *β* are expected to be similar for structurally related carbene derivatives, and we thus assume them to be constant. Applying these approximations leads to Equation 2, where *E* ^don.^ and *E* ^acc.^ relate to the orbital energies of the carbene's π‐system. We thereby introduce the modulus operator for generalization to keep the value of the orbital energy gap always positive (for further details, see S4, S5).

(2) $$RSE=\Delta E_{\mathrm{int}}\propto \;-\frac{1}{|E^{\mathrm{SOMO}}-E^{\mathrm{don}.}|}-\frac{1}{|E^{\mathrm{acc}.}-E^{\mathrm{SOMO}}|}$$

Whereas the π‐donor orbital will be typically the carbene's HOMO−1 or lower, the π‐acceptor orbital will be the carbene's vacant *p*(z) orbital, which represents sometimes, yet not always, the LUMO. If the energy difference between either the donor‐ (*E* ^don.^) or acceptor (*E* ^acc.^) orbital and the SOMO's energy (*E* ^SOMO^) is small, the respective term becomes much more important than the other. Therefore, the other contribution may be neglected [Eq. 3, 4].

(3) $${RSE}^{capto}=E_{int}\propto -\frac{1}{{|E}^{acc.}-E^{SOMO}|}\;$$

(4) $${RSE}^{dative}=E_{int}\propto -\frac{1}{{|E}^{SOMO}-E^{don.}|}\;$$

Based on Equation (3), one expects a linear dependence of the *RSE* on the reciprocal energy of the virtual π‐acceptor orbital *E* ^acc.^ for predominant capto‐stabilization. This is allegedly the case for boryl‐ and late transition metal carbenes. In case of dative stabilization, equation 4 predicts a correlation with the reciprocal energy of a π‐donor orbital *E* ^don^. If both interactions are important, *i.e*. in case of donor/acceptor stabilization, one would have to consider both energy contributions according to Equation (2).

**Figure 3:**
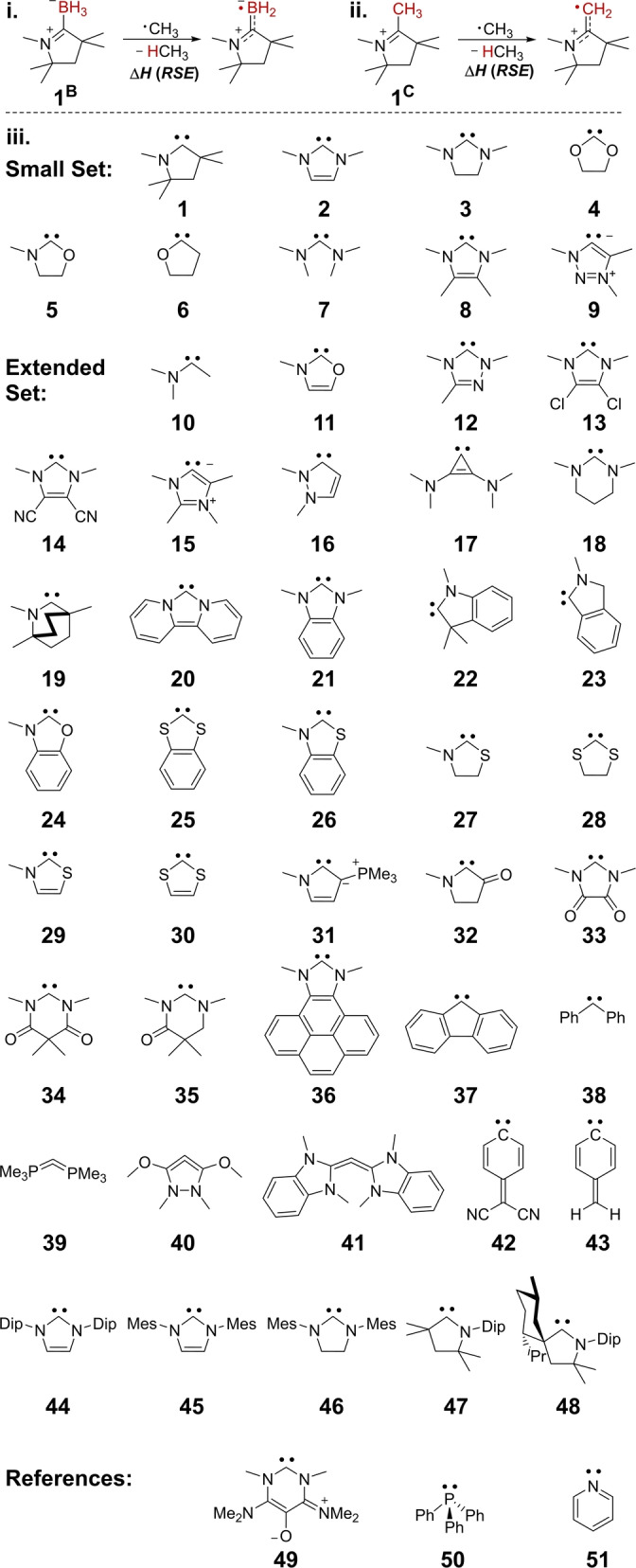
Isodesmic equations used to assess the *RSE*s (top, i. and ii.) and evaluated C‐donors (bottom); Mes: 1,3,5 trimethylphenyl; Dip: 2,5‐diisopropylphenyl.

Methane was chosen as anchor to calculate the *RSE*s of carbene‐borane adducts such as (CAAC)BH_3_ **1^B^** (Figure 3, **i**.) and methylcarbenium cations such as **1^C^** (Figure 3, **ii**.). The C‐donor ligands assessed herein are depicted in the bottom part (**iii**.) of Figure 3. The small set, which comprises simple aliphatic and aromatic heterocyclic‐ and acyclic carbenes (**1**–**8**) and the mesoionic carbene **9** will be used to illustrate general trends.^[52]^ The extended set further includes, among others, three‐membered **17**,^[53]^ benzannulated‐ (**20**–**26**)^[54]^ as well as P‐ and S substituted carbenes (**25**–**31**),^[55]^ carbonyl‐decorated, π‐acidic carbenes (**32**–**35**),^[56]^ and π‐electron rich compounds **39**–**41**.[^36a^, ^36d^, ^36e^, ^37a^] The oxyallyl **49**,^[35c]^ triphenylphosphine (**50**) as well as pyridine (**51**) were evaluated as references, and carbenes **44**–**48** were included to illustrate the minor effects of bulky substituents. The *RSE* values were calculated at the DLPNO‐CCSD(T)/def2‐TZVPP//B3LYP‐D3/def2‐SVP level of theory using tight PNO settings.^[57]^ Benchmark calculations confirmed the accurate computation of experimentally determined values (Δ*E*<4 kJ mol^−1^; Figure S4) with sufficient convergence towards the basis set limit (Figure S5). The carbenes’ orbital energies were calculated using B3LYP/def2‐TZVPP//B3LYP‐D3/def2‐SVP. The B3LYP functional^[58]^ was chosen due to consistency with previous studies.^[43]^ Calculations for the small set revealed an equivalent fit for PW6B95‐D4/def2‐TZVPP,^[59]^ marginally inferior fit for r^2^SCAN‐3c,^[60]^ and a worse fit for HF/def2‐TZVPP//B3LYP‐D3/def2‐SVP (Figures S6–S11).

**Figure 4:**
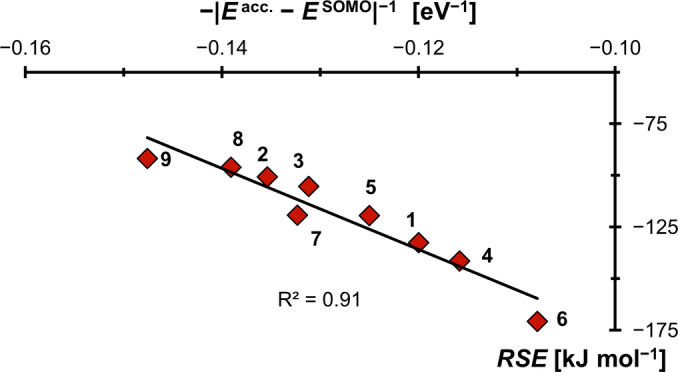
The radical stabilization of carbene‐boryl radicals is indeed capto‐controlled.

Figure 4 shows the calculated *RSE*s in reference to the inverse of the energy difference between *E* ^acc.^ (the energy of the carbenes’ symmetry adapted molecular acceptor orbital; see the Supporting Information for details) and the energy of the ⋅BH_2_‐radical fragment's SOMO (*E* ^SOMO^), which was fitted from the whole data set. A linear fit was not only obtained if using B3LYP‐eigenvalues (R^2^=0.91), but also by the r^2^SCAN‐3c composite method (Figure S6, R^2^=0.91), PW6B95‐D4/def2‐TZVPP (Figure S7, R^2^=0.93), and Hartree‐Fock (HF, Figure S8; R^2^=0.80). As is expected based on Figure 2, π‐acidic carbenes with energetically low‐lying LUMOs such as **1** (*RSE*=−132 kJ mol^−1^), **4** (*RSE*=−141 kJ mol^−1^) and especially **6** (*RSE*=−171 kJ mol^−1^) stabilize the radicals best. Those carbenes are also the ones with the smallest singlet/triplet gaps. In contrast, the mesoionic carbene, lacking strong π‐acceptor capability, affords the lowest *RSE* value (**9**, *RSE*=−92 kJ mol^−1^).

**Figure 5:**
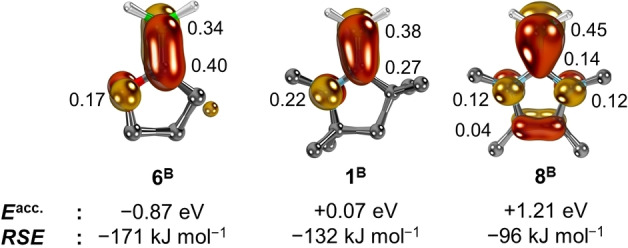
SOMOs, Löwdin's atomic spin densities, *RSE*s of **6^B^**, **1^B^**, **8^B^**, and ***E*** ^**acc**.^ of the carbenes’ π*‐acceptor orbitals. Hydrogen atoms except for the BH_2_ group are omitted for clarity, spin density values are given in [a.u.].

The shapes of the SOMOs, which coincide with the overall spin density, confirm the dilution of spin density (Figure 5). In case of **1^B^** (Figure 5, middle), the spin density is distributed rather evenly across the heteroallyl moiety (Löwdin's population analysis: B, 0.38 a.u.; C^carbene^, 0.27 a.u.; N, 0.22 a.u.). Similar values are obtained by CASSCF(3,3) calculations (B, 0.41 a.u.; C^carbene^, 0.30 a.u.; N, 0.17 a.u.; cf. Figure S1). The energy of the free carbene's LUMO *E* ^acc.^ was calculated at +0.07 eV and the *RSE* at −132 kJ mol^−1^.

**Figure 6:**
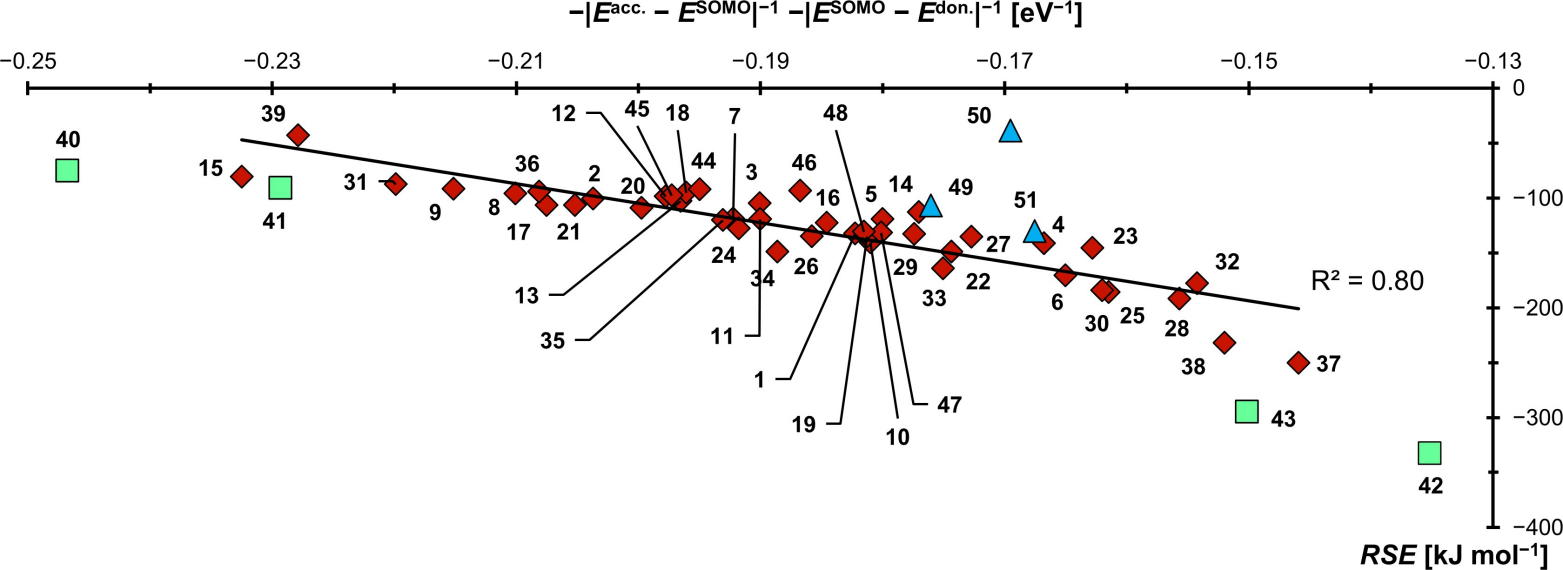
The radical stabilization of carbene boryl radicals is capto‐controlled, yet the inclusion of π‐donation improves the fit moderately. The green squares (**40**–**43**) refer to “redox active” substituents, whereas the blue triangles designate the reference compounds **49**–**51**. These carbenes are omitted from the fit.

The spin delocalization is enhanced for the adduct of the π‐acidic (*E* ^acc^=−0.87) cyclic Fischer carbene (**6^B^**, Fig. 5., left), which shows a larger *RSE* of −171 kJ mol^−1^. In fact, the calculated spin density at the carbene's carbon atom even exceeds the one at the BH_2_ group (B, 0.34 a.u.; C^carbene^, 0.40 a.u.; N, 0.17 a.u.), consequently indicating significant redox non‐innocence. Conversely, in case of comparatively π‐electron rich (*E* ^acc.^=+1.21 eV) **8^B^** with a low *RSE* of −96 kJ mol^−1^, the spin density resides mostly at the BH_2_ group (B, 0.45 a.u.; C^carbene^, 0.14 a.u.; N, 2×0.12 a.u.). We thus conclude that radical stabilization shares mostly capto‐character for the nucleophilic boraolefin radicals. An R^2^ value of 0.79 is obtained (Figure S12), if including the carbenes **1–38**. Accounting additionally for π‐donation according to Equation (1) moderately improves the fit to R^2^=0.80 (Figure 6). Whereas the donor contributions amount to around one third of the overall *RSE*s, its magnitude is in most cases similar and thus cancels largely out. Overall, the π‐donor ligands are the least powerful, whereas the push‐pull carbene **32** (−178 kJ mol^−1^), sulfur containing substituents (*e.g*., **28**, *RSE*=−192 kJ mol^−1^), and especially fluorene derivative **37** (*RSE*=−251 kJ mol^−1^) show high radical stabilization efficacy. Considering the vast application of sulfur based organic radicals in material science and organic electronics, we thus foresee a bright future for radicals based on derivatives of **37**. It is furthermore remarkable, how poor triphenylphosphine **50** (*RSE*=−39 kJ mol^−1^) performs, also in comparison to pyridine (**51**, *RSE*=−131 kJ mol^−1^). The C‐donors **40**–**43** are redox‐active and feature spin density predominantly on the backbone and have thus been omitted from the fit. For instance, in case of **41**, the spin is exclusively located at the benzimidazolium groups, which is in agreement with their bifunctional reactivity^[61]^ and a study dedicated to high valent chromium‐ and cobalt complexes.^[38]^ Also the carbenes **44**–**48**, which are representatives for experimentally common carbene ligands, fit the trend well. Eventually, the *RSE* values correlate with the dilution of spin density at the boron atom (Figure S14) as had been shown previously for other radicals (*vide supra*),^[43]^ yet of course not with Paton's *RSS* metric (Figure S24–S27), which includes steric protection.

**Figure 7:**
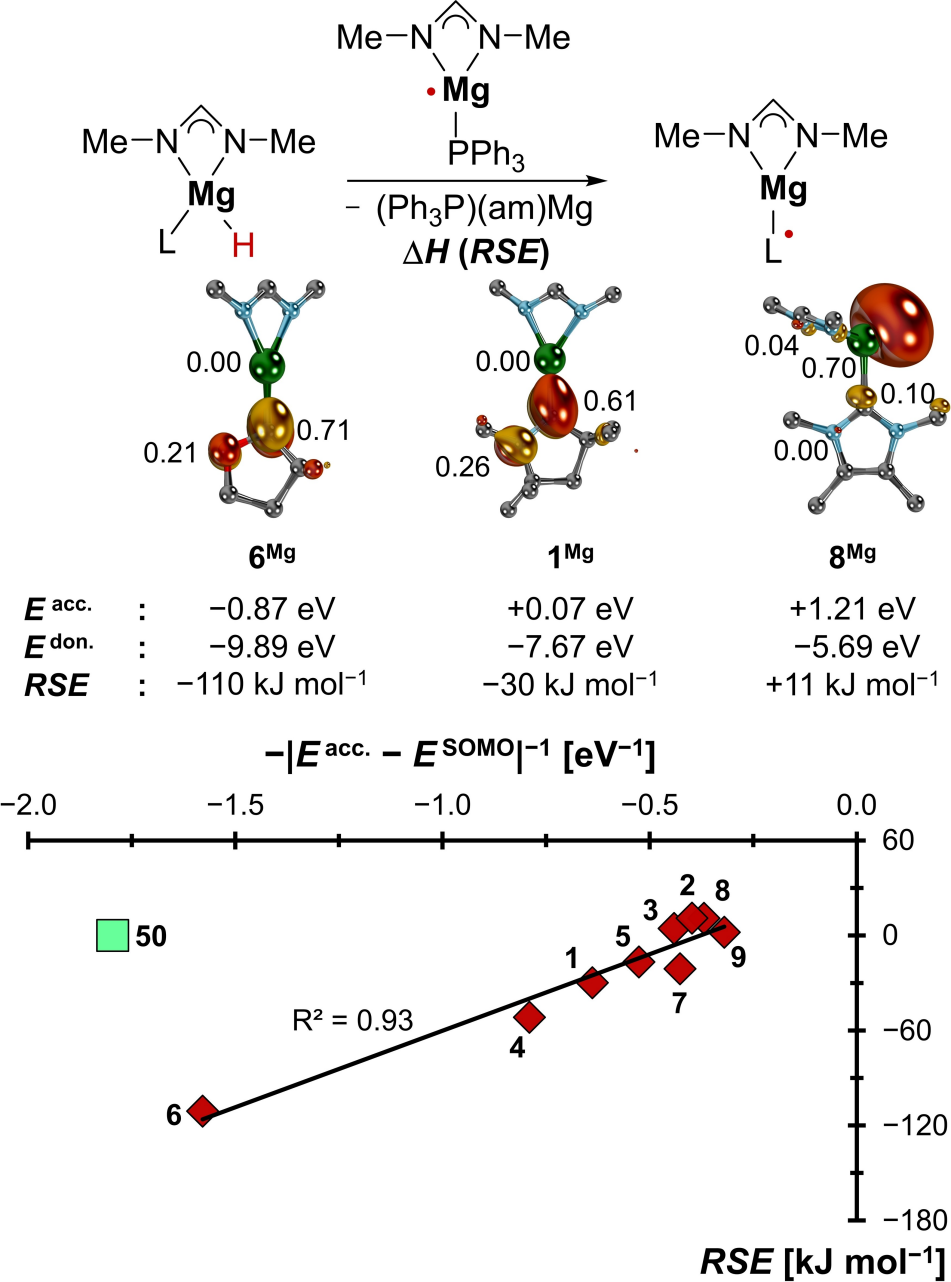
The stabilization of the magnesium radicals is capto‐controlled.

**Figure 8:**
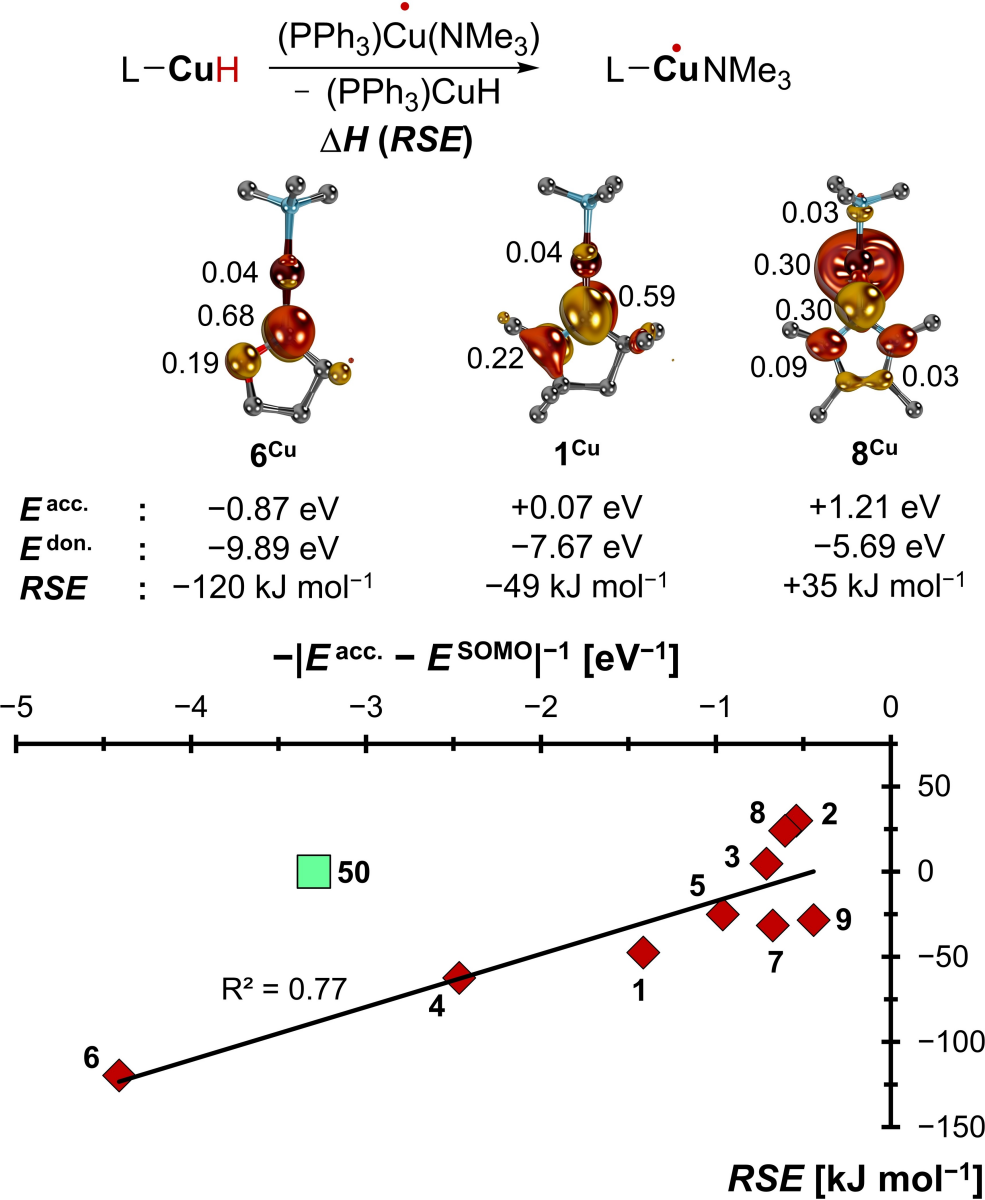
The stabilization of the copper radicals is capto‐controlled.

To verify that boryl‐radicals are appropriate model systems also for *s*‐ and *d*‐block radicals, the *RSE*s for a series of truncated paramagnetic formal copper(0) complexes^[62]^ as well as Harder's formal magnesium(I) radical^[28a]^ were computed (Figures 7, 8; Figures S15–S20). The oxidation state of such compounds is commonly understood as low‐valent, although others argued for a ligand‐based reduction.^[63]^ In both cases, the small set of carbenes (**1**–**9**) anchored versus the triphenylphosphine congeners (**50**) was used. In case of the copper radicals, where the anchor relates to Stryker's reagent, the radical redox stage was additionally coordinatively saturated by trimethylamine.

Indeed, we find the expected trend, with strong π‐acceptors stabilizing both the copper‐ and magnesium radicals through spin‐delocalization, *viz*. backbonding (redox‐noninnocence, respectively). Thus, the CAAC ligand **1** and especially the cyclic Fischer carbene **6** stabilize the radicals well (**1^Cu^**, *RSE*=−49 kJ mol^−1^; **1^Mg^**, *RSE*=−30 kJ mol^−1^; **6^Cu^**, *RSE*=−120 kJ mol^−1^; **6^Mg^**, *RSE*=−111 kJ mol^−1^), whereas the electron‐rich ligand **8** (**8^Cu^**, *RSE*=+35 kJ mol^−1^; **8^Mg^**, *RSE*=+11 kJ mol^−1^) is even less efficient than triphenylphosphine (**50^Cu^**, **50^Mg^**, *RSE*s=0 kJ mol^−1^). The stabilization is capto‐controlled for both metals, like for the boron compounds. These *RSE* values go hand‐hand with accumulation of spin‐density at the ligand's C‐donor atoms, which indicates substantial redox‐non‐innocence of ligands **6** and **1** (**6^Cu^**, 0.68 a.u.; **6^Mg^**, 0.71 a.u.; **1^Cu^**, 0.59 a.u.; **1^Mg^**, 0.61 a.u.), yet not of **8** (**8^Cu^**, 0.30 a.u.; **8^Mg^**, 0.10 a.u.) and the phosphine‐reference **50** (**50^Cu^**, 0.12 a.u.; **50^Mg^**, 0.08 a.u.; Figure S21). Note the remarkable congruency between the spin‐density‐ and *RSE* values calculated for the two metals, which suggests similar electronic structures for these *s*‐ and *d*‐block complexes.

**Figure 9:**
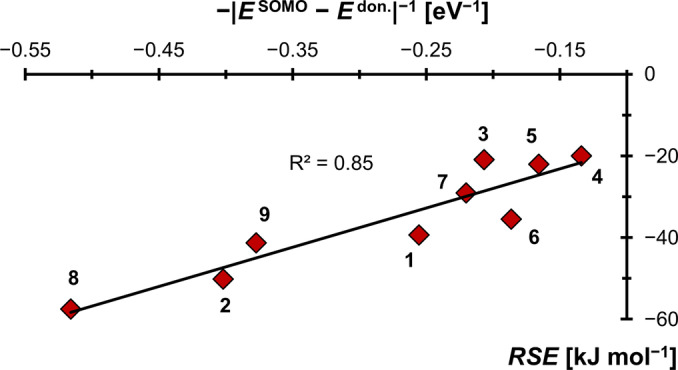
The radical stabilization of cationic carbon‐based radicals in the small set is predominantly dative in nature.

**Figure 10:**
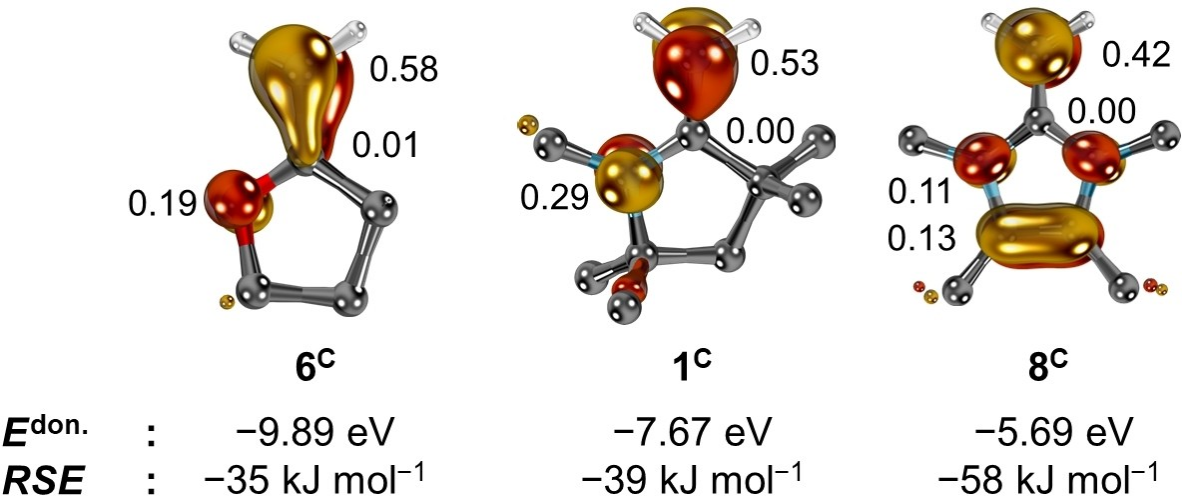
SOMOs, Löwdin's atomic spin densities, *RSE*s of **6^C^**, **1^C^**, **8^C^**, and ***E*** ^**don**.^ of the carbenes’ π‐donor orbitals. Hydrogen atoms except for the CH_2_ group are omitted for clarity, spin density values are given in [a.u.].

The carbon centered cationic radicals follow the opposite trend (Figure 9). The calculated *RSE*s as well as the R^2^ value of 0.85 for the small set are lower than for the boron radicals. Intriguingly, the order of *RSE*s is opposed to what was obtained for the boron derived radicals. For instance, remarkable small *RSE*s were computed for the π‐acceptor carbenes **4** (*RSE*=−20 kJ mol^−1^) and **5** (*RSE*=−22 kJ mol^−1^), whereas higher *RSE*s are found for π‐donating **8** (*RSE*=−58 kJ mol^−1^). This trend indicates predominant dative interaction as is corroborated by the shapes of the SOMOs, which reveal only marginal spin densities at the carbenes’ carbon atoms (Figure 10; *cf*. Figure 2).

B3LYP predicts the absence of spin density (0.00 a.u.) at the carbene carbon atom in **1^C^**, and also Löwdin's population analysis of CASSCF(3,3) calculations places only 0.04 a.u. at this position. Instead, the highest spin density is found at the terminal methylene group. The accumulation of spin decreases (**6^C^**, 0.58 a.u.; **1^C^**, 0.53 a.u.; **8^C^**, 0.42 a.u.) in line with the *RSE* values (**6^C^**, −35 kJ mol^−1^; **1^C^**, −39 kJ mol^−1^; **8^C^**, −58 kJ mol^−1^) and the energies *E* ^don.^ of the carbenes’ bonding π‐orbitals (**6^C^**, −9.89 eV; **1^C^**, −7.67 eV; **8^C^**, −5.69 eV). Energetically elevated π‐donor orbitals entail better interaction with the SOMO‐fragment, consequently boosting radical stability.

**Figure 11:**
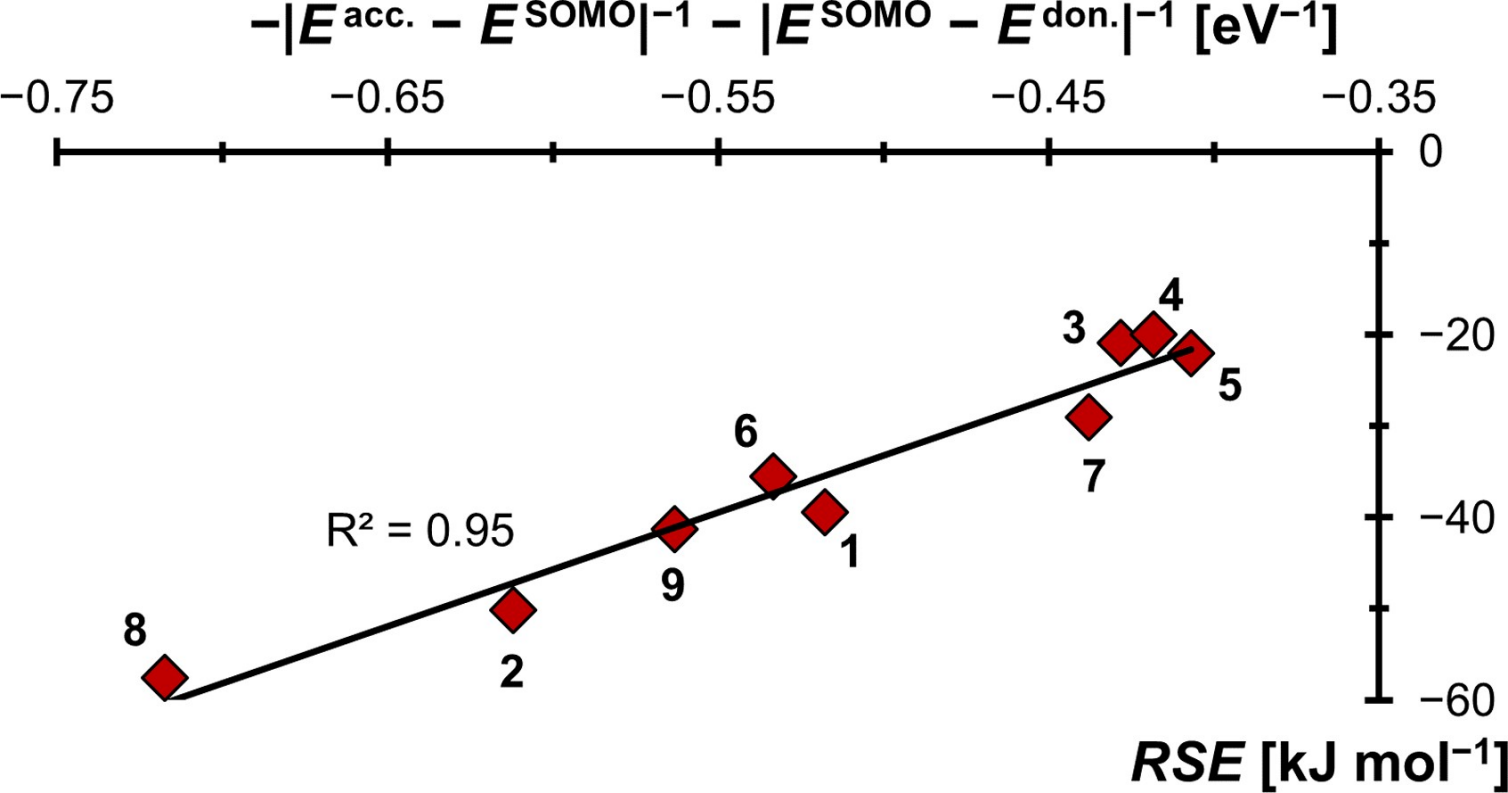
A better description of cationic carbon‐based radicals is indeed given through concomitant consideration of dative‐ and capto‐effects.

The moderate fit in Figure 9 is mostly due to negligence of the capto‐interaction for these cationic radicals. In fact, the R^2^ value improves to 0.95 upon further inclusion of acceptor‐stabilization (Figure 11). This finding confirms that whereas the donor‐interaction is more important for carbon centered cationic radicals, a captodative picture allows for a better description of the electronic structure. Indeed, a poor fit of R^2^=0.13 (Figure S22) is obtained for the whole set of carbenes, if only accounting for the donor interactions.

**Figure 12:**
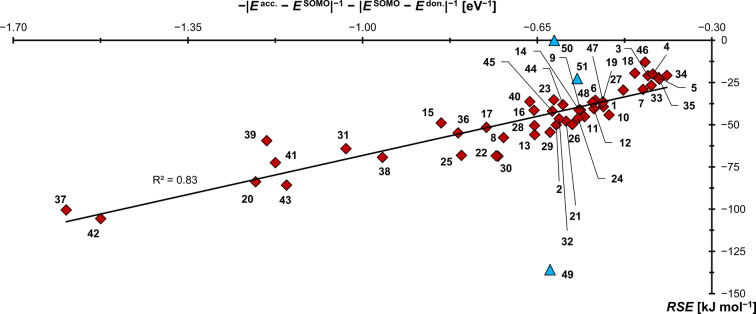
The radical stabilization of cationic olefin radicals is controlled by both capto‐ as well as dative interactions. The blue triangles (**49**–**51**) designate the reference molecules, which are omitted from the fit.

Accordingly, the captodative picture affords striking agreement between the calculated *RSE* values and the computed fragment frontier orbital energies for all investigated carbenes (R^2^=0.83; Figure 12). Also here, a correlation with the dilution of spin‐density is found (Figure S21) and the π‐electron rich carbenes with hidden carbon(0) character^[64]^ such as **20** (*RSE*=−84 kJ mol^−1^)^[54a]^ and carbodicarbene **41** (*RSE*=−73 kJ mol^−1^), as well as the triplet‐carbenes fluorene (**37**, *RSE*=−101 kJ mol^−1^), dicyanoquinodimethane (**42**, *RSE*=−106 kJ mol^−1^) and quinodimethane (**43**, *RSE*=−86 kJ mol^−1^) perform best. In contrast, “conventional” π‐acceptor carbenes such as **3** (*RSE*=−21 kJ mol^−1^), and the carbonyl decorated carbenes such as **33** (*RSE*=−27 kJ mol^−1^) perform only moderate. Finally, triphenylphosphine is computed again to be a remarkable poor radical‐stabilizing group (**50**, *RSE*=0 kJ mol^−1^).

## Conclusion

In short, we presented a donor/acceptor description of π‐radical interaction, which allows to intuitively understand and predict the thermodynamic stability of carbene‐radicals. More specifically, we described radical stabilization by a frontier orbital picture. For that purpose, radical stabilization energies (*RSE*s) of carbene‐derived boryl‐ and carbon radicals were computed at the CCSD(T)//DFT level of theory. Using selected open‐shell magnesium‐ and copper complexes, we showed that the boryl‐radicals not only serve as a model for *p*‐block radicals, but also for late transition metal complexes and low‐valent *s*‐block compounds. Overall, we provide seven convenient guidelines to rationalizing radical stabilization by carbene ligands and carbene‐derived substituents:

1. Radical stabilization by carbenes, carbene ligands, and arguably conjugated systems of sufficient covalency in general, is well approximated by perturbative analysis of the frontier orbitals of the allyl radical.
2. The *RSE*s of boryl‐, copper‐ and magnesium radicals is essentially controlled by the π‐withdrawing capabilities of the carbene‐derived substituent. Carbon‐based radicals are mostly stabilized through π‐donation from the carbene, yet the acceptor properties are also significant for these cationic molecules. This coincides with an ambiphilic push–pull perspective on radical stabilization.
3. Contrarily to common believe, both π‐acceptor and π‐donor interactions are potentially important for Fischer‐type carbene adducts and ‐ligands including NHCs. The π‐acceptor properties are typically (yet not always) associated with the LUMO of the carbene, whereas the π‐donor interaction stems often from the HOMO−1.
4. The identification and quantification of significant π‐donor effects in carbene‐derived radicals adds a new perspective to carbene chemistry. For instance, it identifies a novel key design criterium for synthesizing high‐valent and/or open‐shell carbene complexes.
5. As a rule of thumb, carbenes with small HOMO–LUMO energy gaps will stabilize radicals well, since both capto‐ and dative stabilization will then likely contribute to the overall stability.
6. Most carbenes hitherto popularized for radical stabilization do not perform better in respect to many other heterocycles. We therefore identify a vast unexplored chemical space and show that radical stabilization by carbenes relies typically on steric bulk for kinetic protection.
7. Decoration of carbenes allows to tune radical stabilization by more than 300 kJ mol^−1^. This value exceeds the radical stabilization exerted by, for instance, 2,2,6,6‐tetramethylpiperidinyloxyl (TEMPO; *RSE*=−206 kJ mol^−1^)^[41a]^ by far. We thus define long‐sought design principles for radical‐stabilizing groups of hitherto unrivaled efficacy.

## Conflict of interest

The authors declare no conflict of interest.

1

## Supporting information

As a service to our authors and readers, this journal provides supporting information supplied by the authors. Such materials are peer reviewed and may be re‐organized for online delivery, but are not copy‐edited or typeset. Technical support issues arising from supporting information (other than missing files) should be addressed to the authors.

Supporting Information

## Data Availability

The data that support the findings of this study are available in the Supporting Information of this article.
