# Supplementary material for: Gauging Radical Stabilization with Carbenes
Source: Angew Chem Int Ed Engl. 2022 Aug 4;61(37):e202206390. doi: 10.1002/anie.202206390 (PMC9545232; doi:10.1002/anie.202206390)
Supplement: Supplementary file 1 — Supporting Information [file ANIE-61-0-s001.pdf]

Angewandte Chemie
Eine Zeitschrift der Gesellschaft Deutscher Chemiker

Supporting Information

Gauging Radical Stabilization with Carbenes

*K. Breitwieser, H. Bahmann, R. Weiss, D. Munz**

Table of Contents

1. Computational Details S3
2. Derivation of Equation 2 .... S4
3. Benchmarking S6
4. Comparison of Methods S8
5. Alternative Fits & Metal Complexes S10
6. Comparison with the Radical Stability Score *RSS* S15
7. Energies S20
8. Canonical Molecular Orbitals of Carbenes S32
9. XYZ Coordinates S44
10. Author Contributions S118
11. References S118

1.) Computational Details

The calculations were performed with ORCA 4.2.1.[1] All structures were optimized at the B3LYP-D3/def2-SVP level of theory[2] with the resolution of identity and chain of spheres approximation (*RIJCOSX*) and the related auxiliary basis set (*def2/J*).[3] The B3LYP functional was chosen for consistency with previous studies.[4] Test-calculations (ORCA 5.0.2) with the fast composite method r^2SCAN-3c[5] (Fig. S6, S9; slightly inferior fit for the small set) and PW6B95-D4/def2-TZVPP[6] (Fig. S7, S10; comparable fit for the small set) gave consistent results. Tighter than default convergence- (*tightscf*) and grid (*grid5, finalgrid6*) criteria were used. All calculated structures were verified as true minima by the absence of imaginary eigenvalues in the harmonic vibrational frequency analysis. No symmetry or internal coordinate constraints were applied during optimizations. The D3 dispersion correction with Becke-Johnson (D3BJ) damping was applied.[7] The orbital eigenvalues of the C-donors were calculated at the restricted B3LYP/def2-TZVPP//B3LYP-D3/def2-SVP level of theory. For carbenes with a triplet ground state (**37**, **38**, **42**, **43**), the structural parameters of the triplet states were used. In these cases, the eigenvalue from the α-orbital set was used for the donor orbitals, whereas the eigenvalue from the β-orbital set was used for the acceptor orbitals. The energies of the fragment's SOMOs were fitted to the whole data set, yet values in a similar order of magnitude are obtained computationally for anionic boryl radicals and carbon radicals. In case of $\cdot BH_2$, a value of 8.4 eV (see Fig. S13 for $E^{SOMO} = -4$ eV), and in case of $\cdot CH_2$ a value of −3.75 eV was used. For the magnesium and copper radicals, −1.5 eV and −0.64 eV were used, respectively. The electronic energies of all structures except the carbenes were computed by single-point calculations at the DLPNO-CCSD(T)/def2-TZVPP level of theory, relying on quasi-restricted orbitals (QROs), and using tighter-than-default (*tightpno*) settings.[8] The T1 values in the CCSD(T) calculations remained in all cases <0.02. In cases of substantial spin contamination, DFT reference determinants were evaluated, yet afforded consistent/less reliable results. Benchmarking studies were performed in reference to experimentally[9] obtained enthalpies. On average, the chosen computational method deviates by <4 kJ mol^{-1} from experimentally determined enthalpies (*cf.* Fig. S4). A comparison with larger basis sets confirmed that the def2-TZVPP is sufficiently accurate (*cf.* Fig. S5). The CASSCF single point calculations were also performed at the triple-ζ level of theory. Eventually note that the meaning of Kohn-Sham virtual orbital eigenvalues remains a debated matter.[10]

2.) Derivation of Equation 2

Salem described the orbital interaction energy ΔE_{int} as follows:[11]

$$\Delta E_{int} = -2\sum_{j}^{occ}\sum_{k'}^{unocc}\frac{\left(\sum_{rr'} c_{jr}c_{k'r'}\eta_{rr'}\right)^2}{E_{k'}-E_j} - 2\sum_{j'}^{occ}\sum_{k}^{unocc}\frac{\left(\sum_{rr'} c_{kr}c_{j'r'}\eta_{rr'}\right)^2}{E_k-E_{j'}} \quad \text{(S1)}$$

Under the assumption that the frontier orbital interaction is most important, this can be simplified:

$$\Delta E_{int} = -2\frac{\left(\sum_{rr'} c_r^{HOMO}c_{r'}^{LUMO'}\eta_{rr'}\right)^2}{E^{LUMO'}-E^{HOMO}} - 2\frac{\left(\sum_{rr'} c_r^{LUMO}c_{HOMO'r'}\eta_{rr'}\right)^2}{E^{LUMO}-E^{HOMO'}} \quad \text{(S2)}$$

The atomic orbital coefficients *c* and resonance integrals β are expected to be similar for structurally related carbene derivatives, and we thus assume them to be constants:

$$\Delta E_{int} \propto -\frac{1}{(E^{LUMO'}-E^{HOMO})} - \frac{1}{(E^{LUMO}-E^{HOMO'})} \quad \text{(S3)}$$

Transferring this expression to the radical system leads to:

$$\Delta E_{int} \propto -\frac{1}{(E^{SOMO}-E^{don.})} - \frac{1}{(E^{acc.}-E^{SOMO})} \quad \text{(S4)}$$

This relationship describes the carbon radicals (SOMO = −3.75 eV) well, as the formal SOMO is fitted with an energy between the donor- and the acceptor orbitals (Fig. S1).

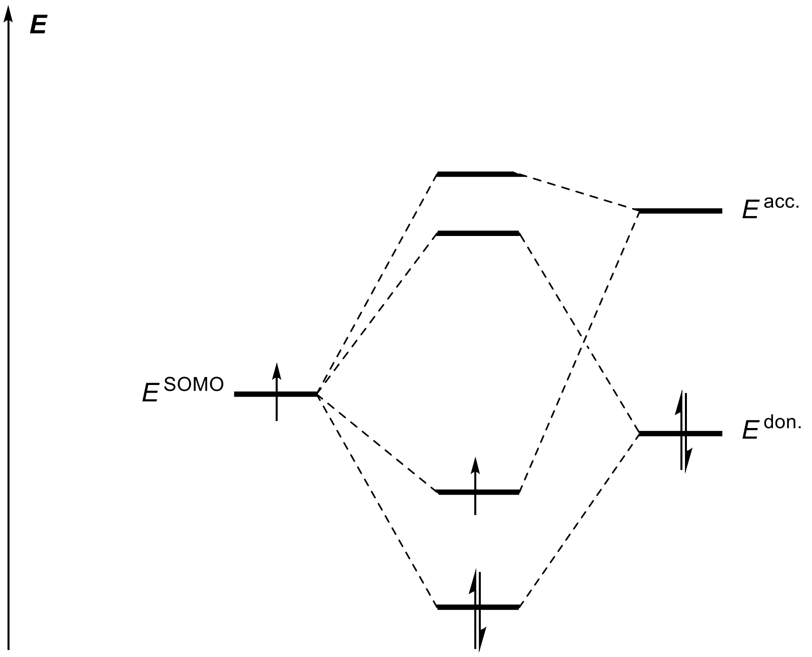

Figure S1. MO diagram for radical interaction with a π-donor and π-acceptor orbital.

This relationship is not applicable, if (1) the donor and acceptor orbitals have very similar energies, as the interaction energy will become infinite. Furthermore (2), in the boron case, we fitted the energy of the formal SOMO to a value above $E^{acc.}$, since it shares anionic character. Note that this implies “physically” a “subsequent” charge transfer.

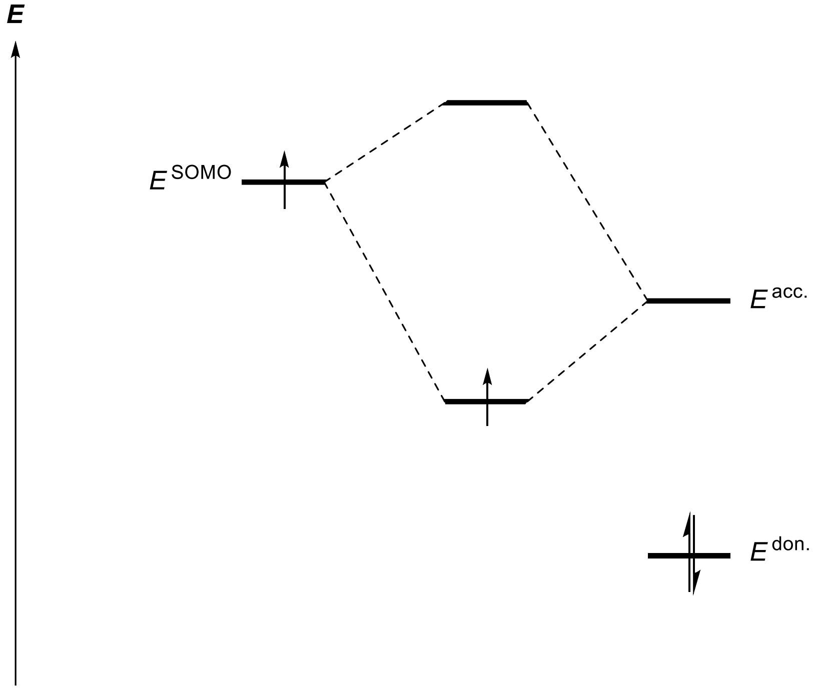

Fig. 2. MO diagram for radical interaction with the formal fragment-SOMO being higher in energy than the π-acceptor orbital, *i.e.* a charge-transfer situation.

As a result, the ($E^{acc.}$ – E^{SOMO}) term becomes negative. Hence, the order of the acceptor energy $E^{acc.}$ and the SOMO’s energy E^{SOMO} must be switched in the second term to be consistent with a stabilization due to the orbital interaction:

$$\Delta E_{int} \propto -\frac{1}{(E^{SOMO}-E^{don.})}-\frac{1}{(E^{SOMO}-E^{acc.})} \quad \text{(S5)}$$

To generalize Salem’s relationship that the denominators are always positive, we use in the main part of the manuscript the modulus operator, thus preventing in any case negative denominators:

$$\Delta E_{int} \propto -\frac{1}{|E^{SOMO}-E^{don.}|}-\frac{1}{|E^{acc.}-|E^{SOMO}|} \quad \text{(S6)}$$

3.) CASSCF & Benchmarking

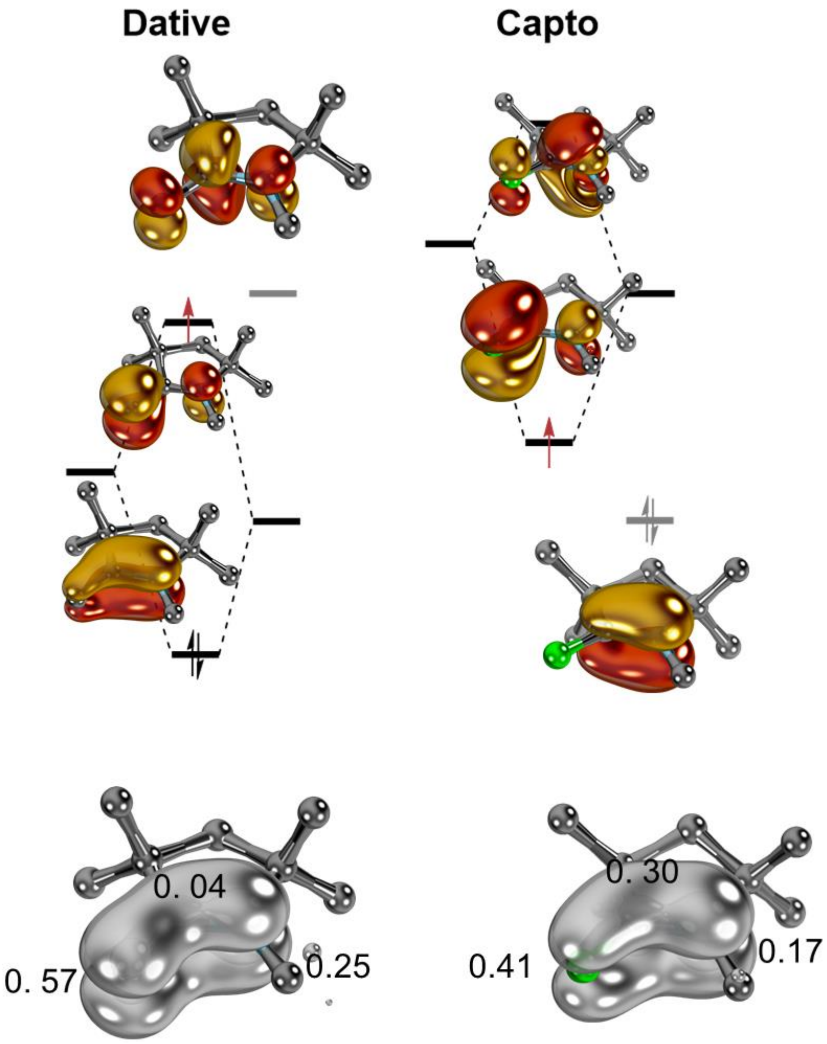

Figure S3. Orbital mixing as obtained by CASSCF(3,3) for **1**^{C} (left) and **1**^{B} (right) and related spin density maps (bottom) under indication of Löwdin's atomic spin density in [a.u.] per atom within the (hetero)allyl system.

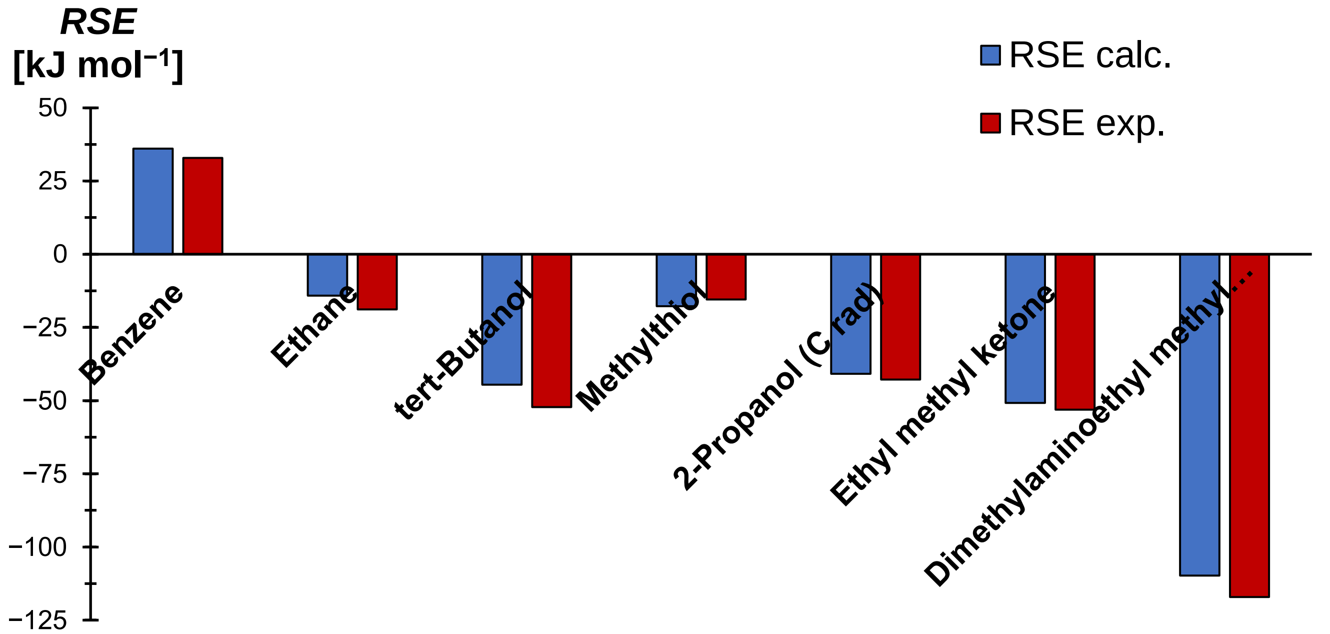

Figure S4. Comparison of *RSE* values obtained by the DLPNO-CCSD(T)/def2-TZVPP//B3LYP-D3/def2-SVP level of theory with the experimental[1] values.

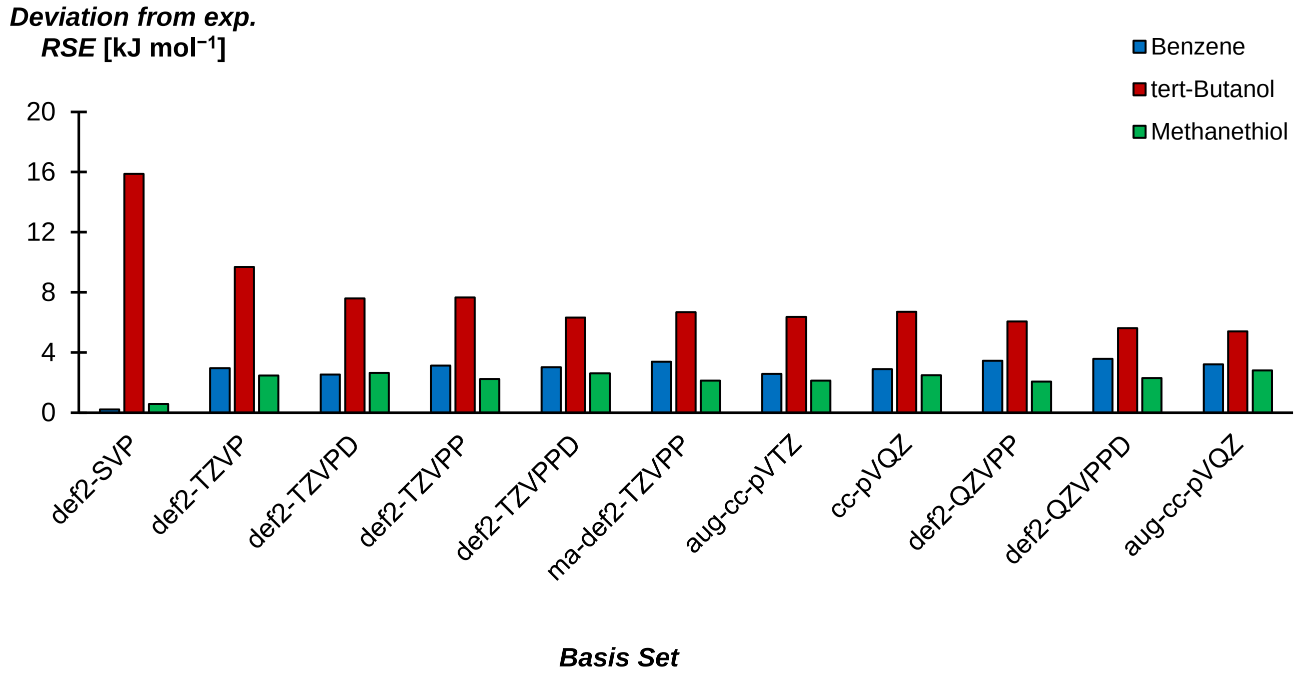

Figure S5. Basis set convergence of DLPNO-CCSD(T) single point energies for the *RSE* of benzene (anchored *vs.* methane), *tert*-butanol (anchored *vs.* water) and methanethiol (anchored *vs.* H_2S) in reference to the experimental[9] values: The def2-TZVPP basis set shows reasonable accuracy at comparatively low computational cost.

4.) Comparison of Methods

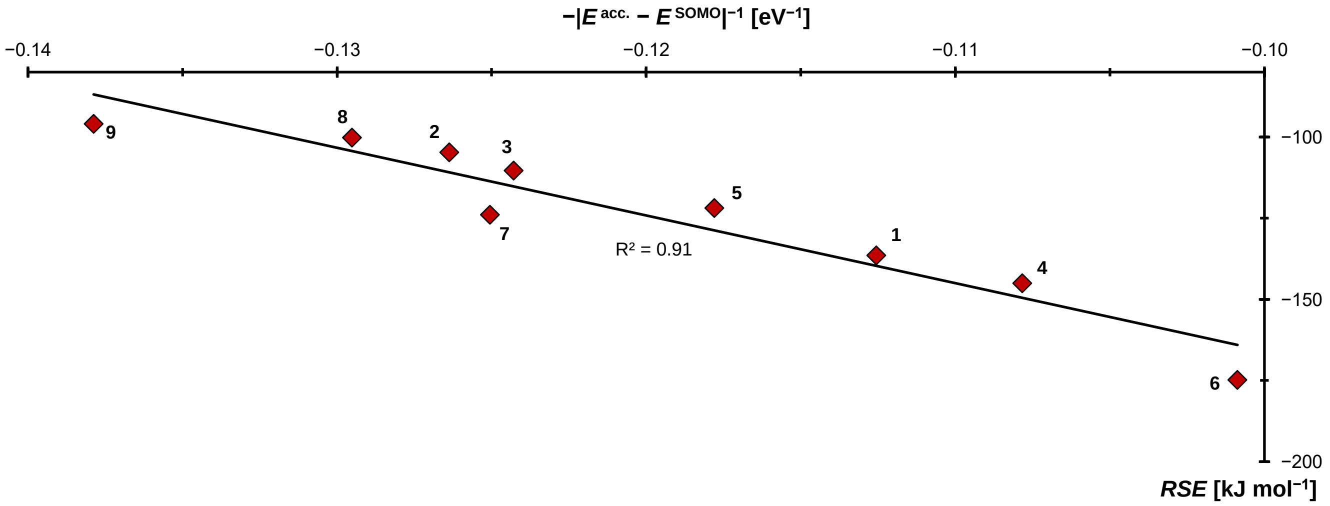

Figure S6. Fit obtained for the boryl radicals of the small set using the DLPNO-CCSD(T)/def2-TZVPP//r^{2}SCAN-3c level of theory.

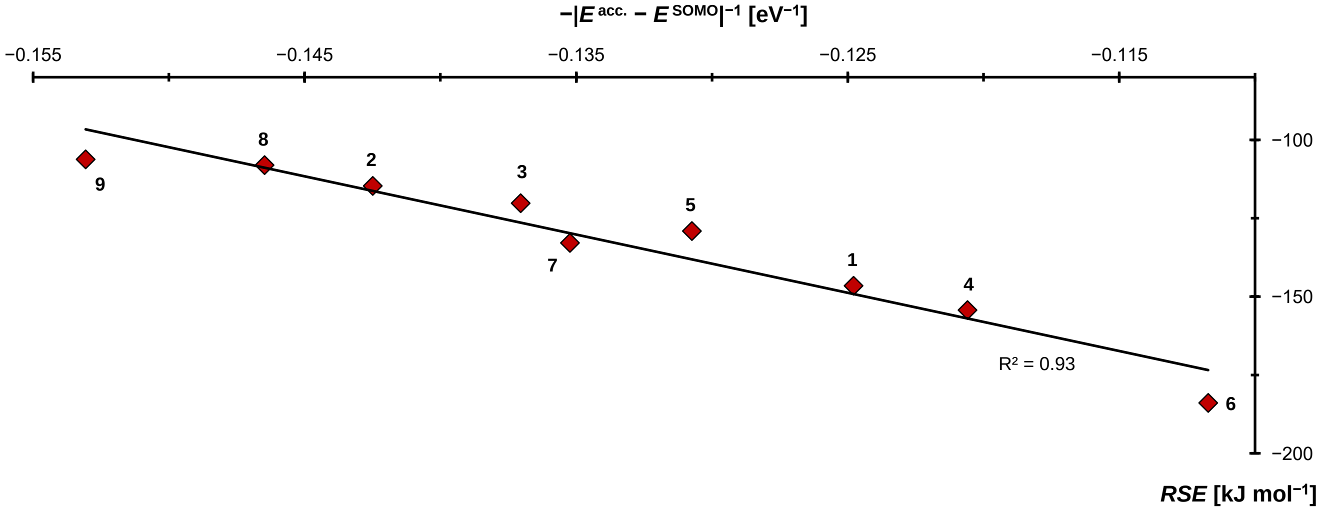

Figure S7. Fit obtained for the boryl radicals of the small set using the DLPNO-CCSD(T)/def2-TZVPP//PW6B95-D4/def2-TZVPP level of theory.

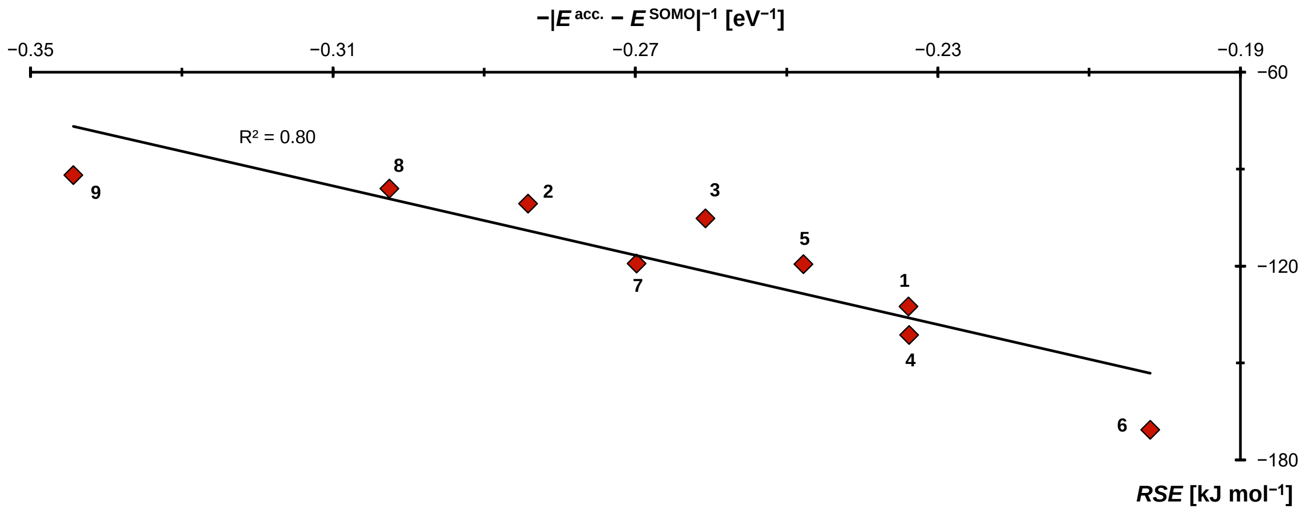

Figure S8. Fit obtained for the boryl radicals of the small set using the DLPNO-CCSD(T)/def2-TZVPP//B3LYP-D3/def2-SVP level of theory, using orbital eigenvalues as obtained by HF/def2-TZVPP singlepoint calculations.

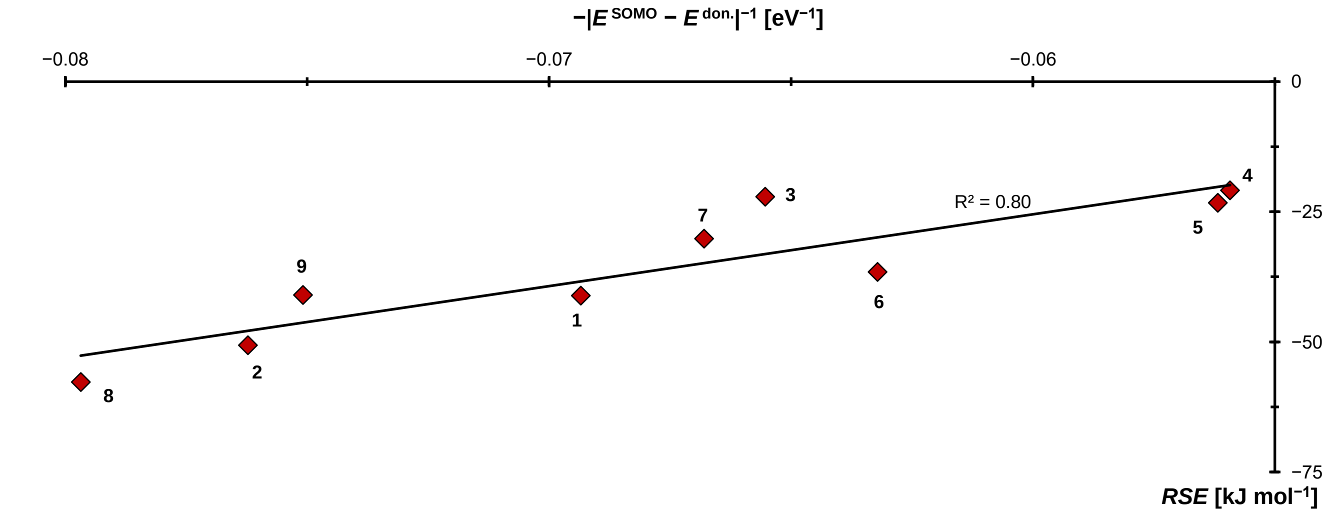

Figure S9. Fit obtained for the carbon radicals of the small set using the DLPNO-CCSD(T)/def2-TZVPP//r^{2}SCAN-3c level of theory.

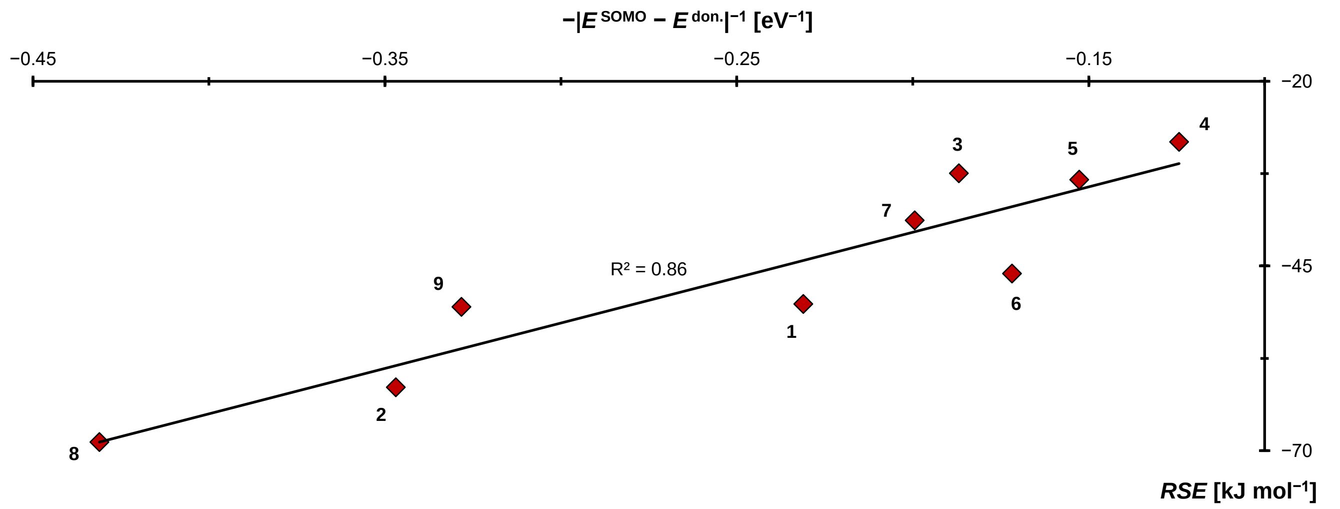

Figure S10. Fit obtained for the carbon radicals of the small set using the DLPNO-CCSD(T)/def2-TZVPP//PW6B95-D4/def2-TZVPP level of theory.

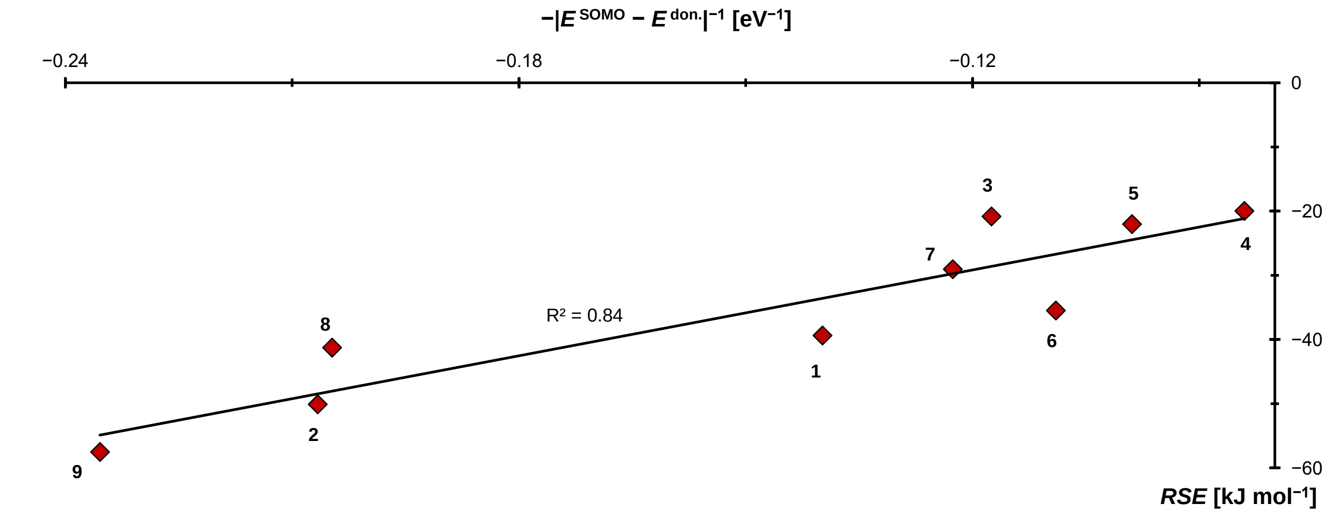

Figure S11. Fit obtained for the carbon radicals of the small set using the DLPNO-CCSD(T)/def2-TZVPP//B3LYP-D3/def2-SVP level of theory, using orbital eigenvalues obtained by HF/def2-TZVPP singlepoint calculations.

5.) Alternative Fits & Metal Complexes

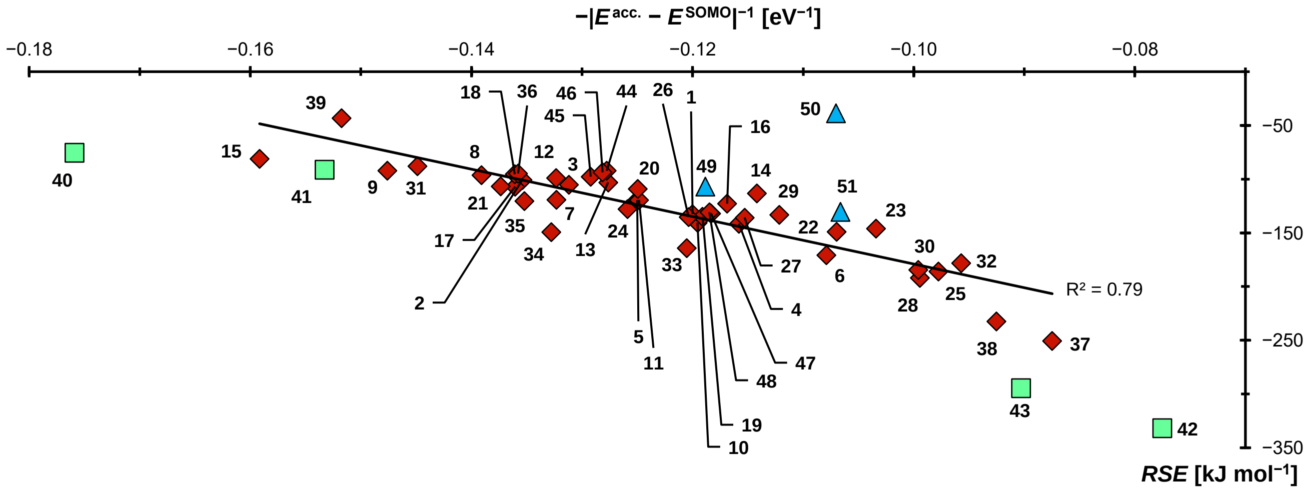

Figure S12. Fit obtained for the boryl radicals, if only considering the acceptor interaction.

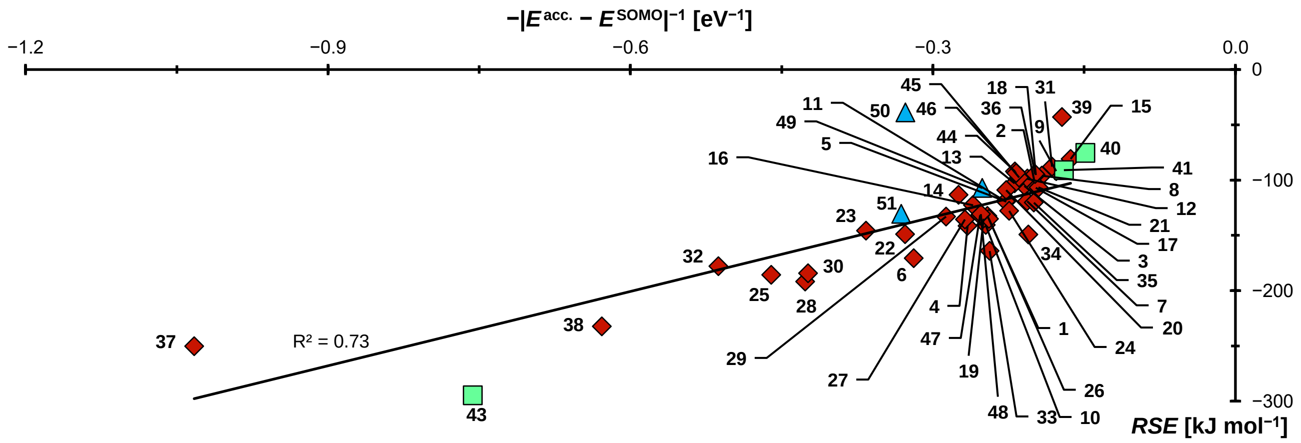

Figure S13. Fit obtained for the boryl radicals with $E^{SOMO} = -4$ eV, if only considering the acceptor interaction. Note that in this case, an unphysically high donor interaction is obtained.

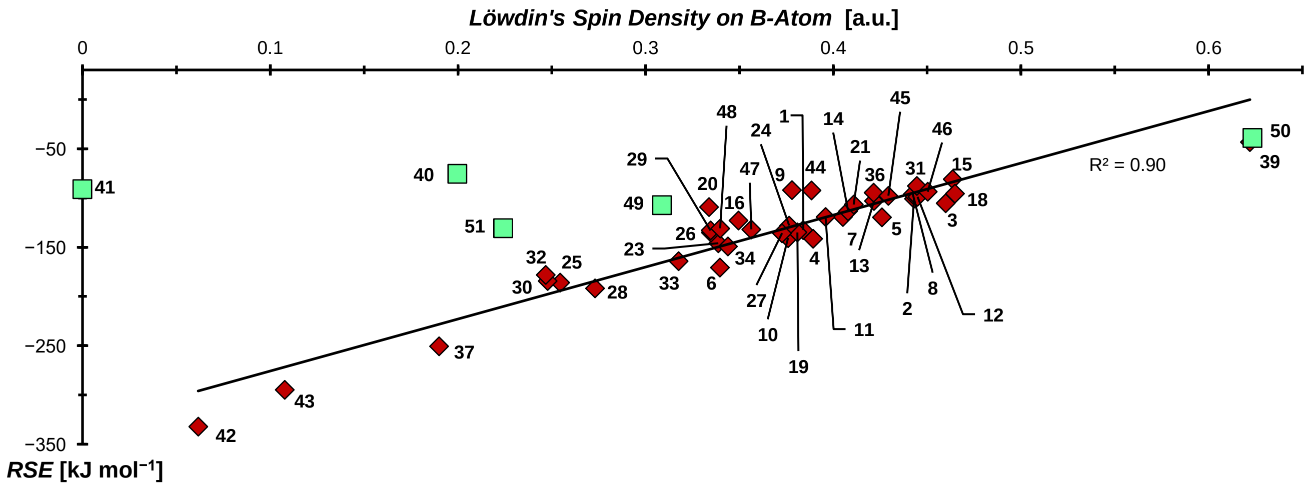

Figure S14. Löwdin's atomic spin density at the boron atoms (red squares) for compounds $\mathbf{n}^{B}$ correlates with the *RSE*s (except for the redox-active C-donors and reference molecules, green squares).

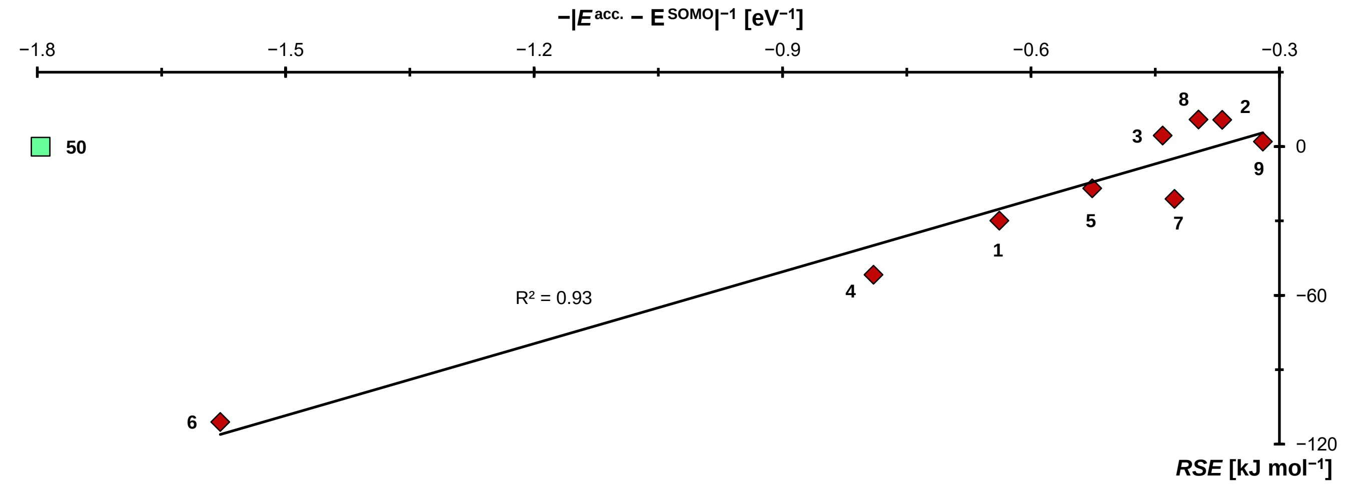

Figure S15. Fit obtained for the magnesium radicals if only considering the acceptor interaction. The reference molecule **50**^{Mg} is given as a green square.

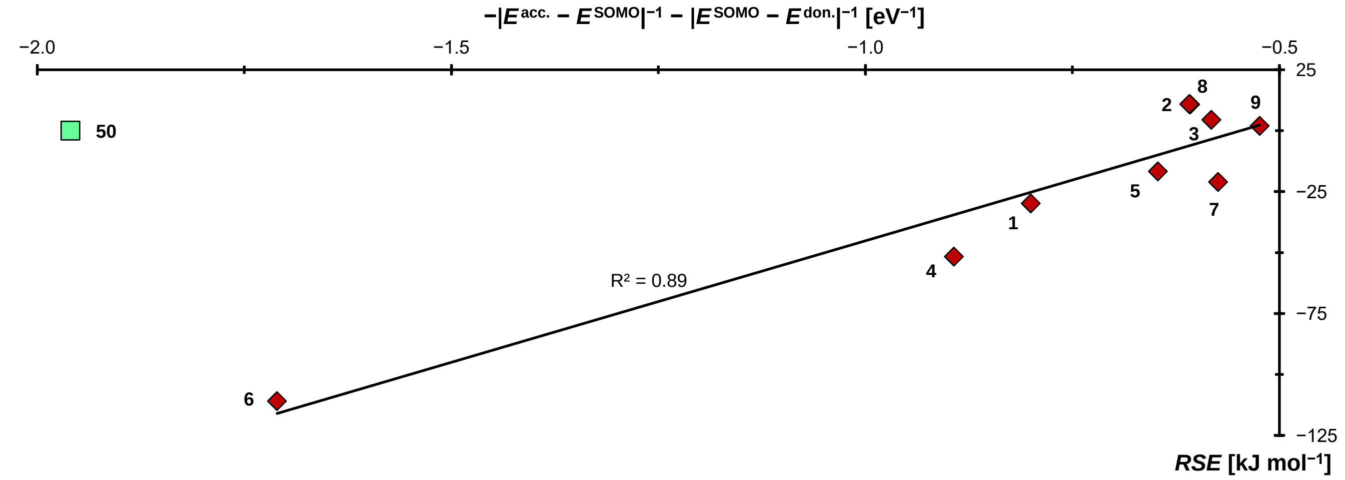

Figure S16. Fit obtained for the magnesium radicals considering the acceptor and donor interactions. The reference molecule **50**^{Mg} is given as a green square.

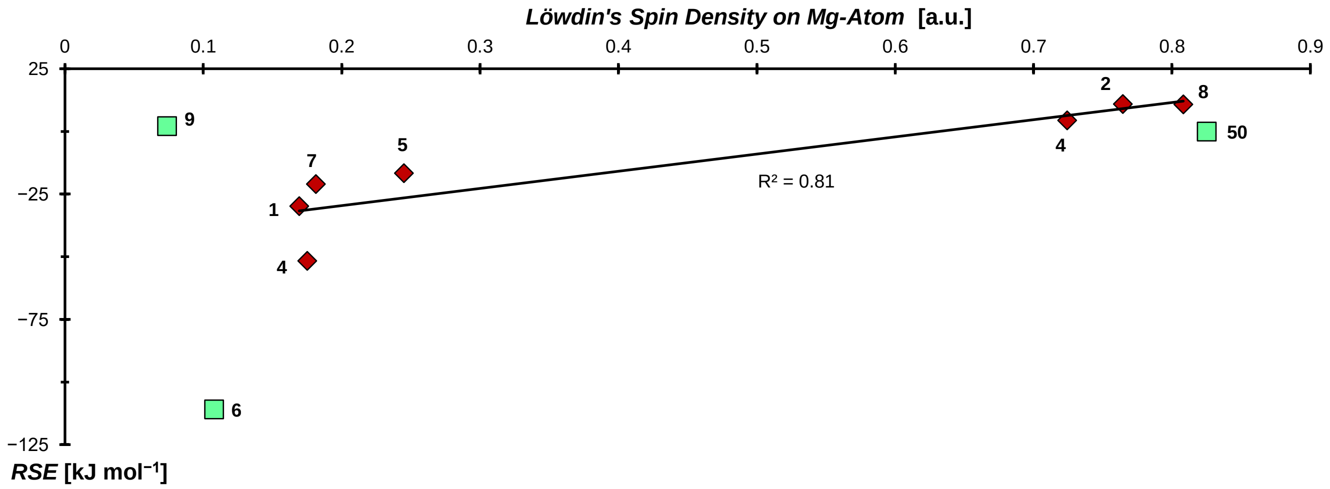

Figure S17. Löwdin's atomic spin density at the magnesium atoms (red squares) for compounds **n**^{Mg} correlates with the *RSE*s (except for the reference molecule **50**^{Mg} and the complexes **9**^{Mg} and **6**^{Mg}, green squares).

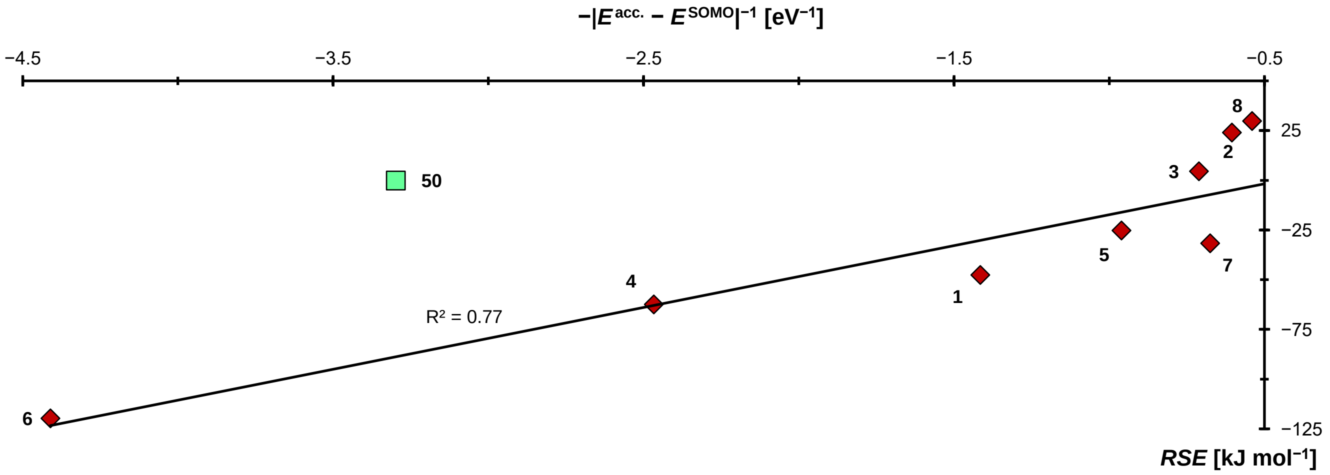

Figure S18. Fit obtained for the copper radicals if only considering the acceptor interaction. The reference molecule **50**^{Cu} is given as a green square.

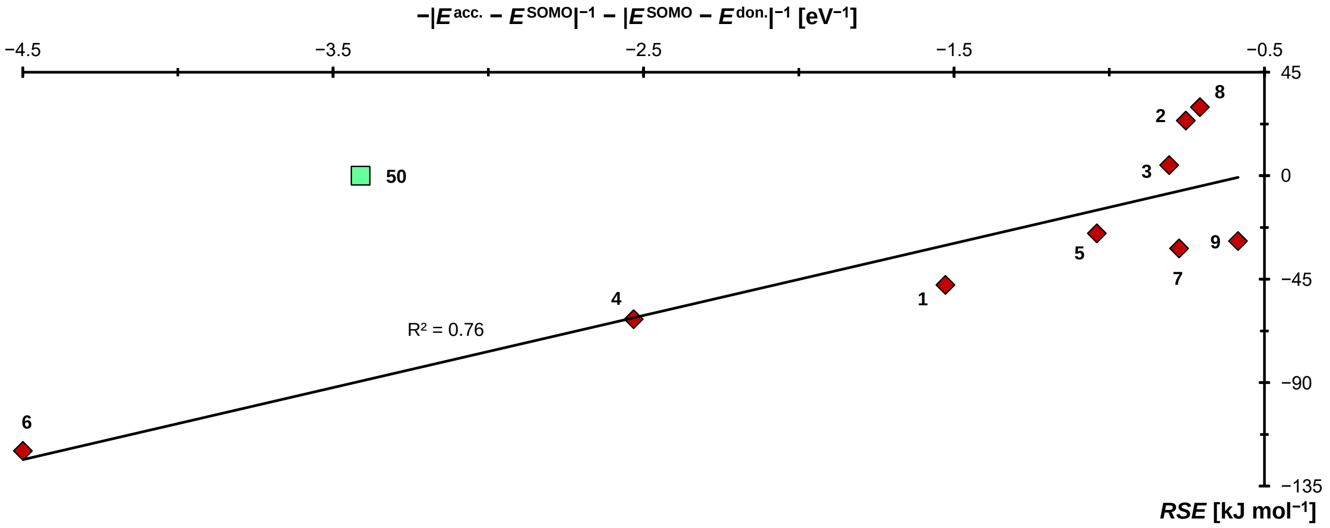

Figure S19. Fit obtained for the copper radicals considering the acceptor and donor interactions. The reference molecule **50**^{Cu} is given as a green square.

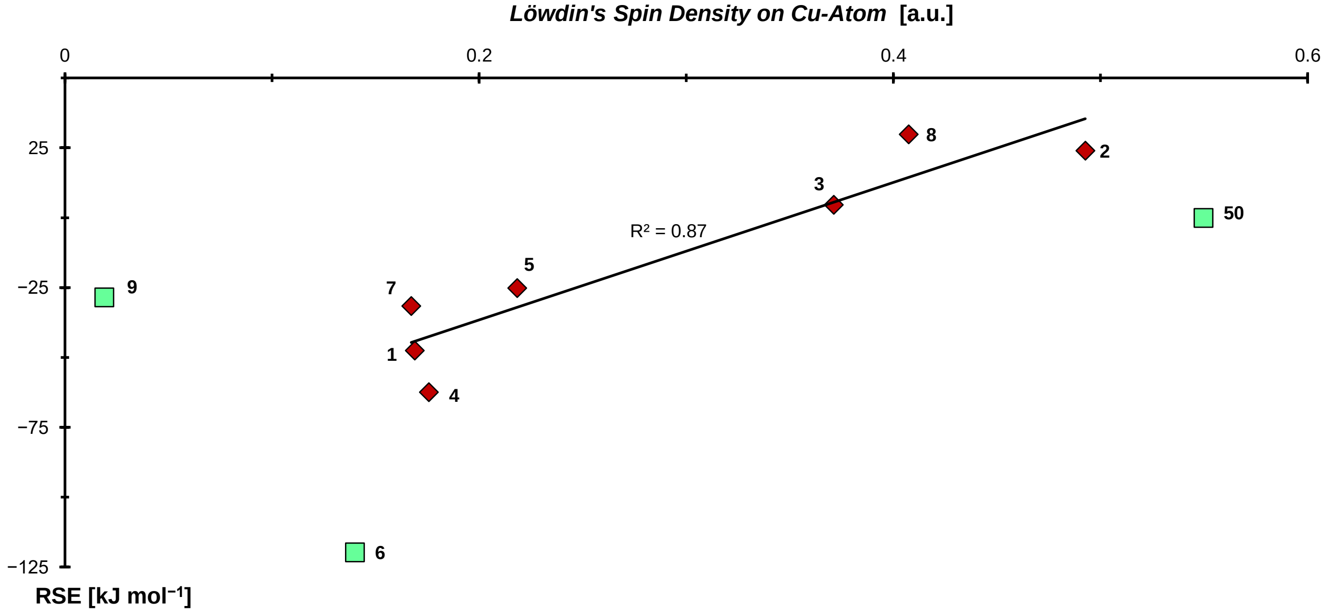

Figure S20. Löwdin's atomic spin density at the copper atoms (red squares) for compounds **n**^{Cu} correlates with the *RSE*s (except for the reference molecule **50**^{Cu} and the complexes **9**^{Cu} and **6**^{Cu}, green squares).

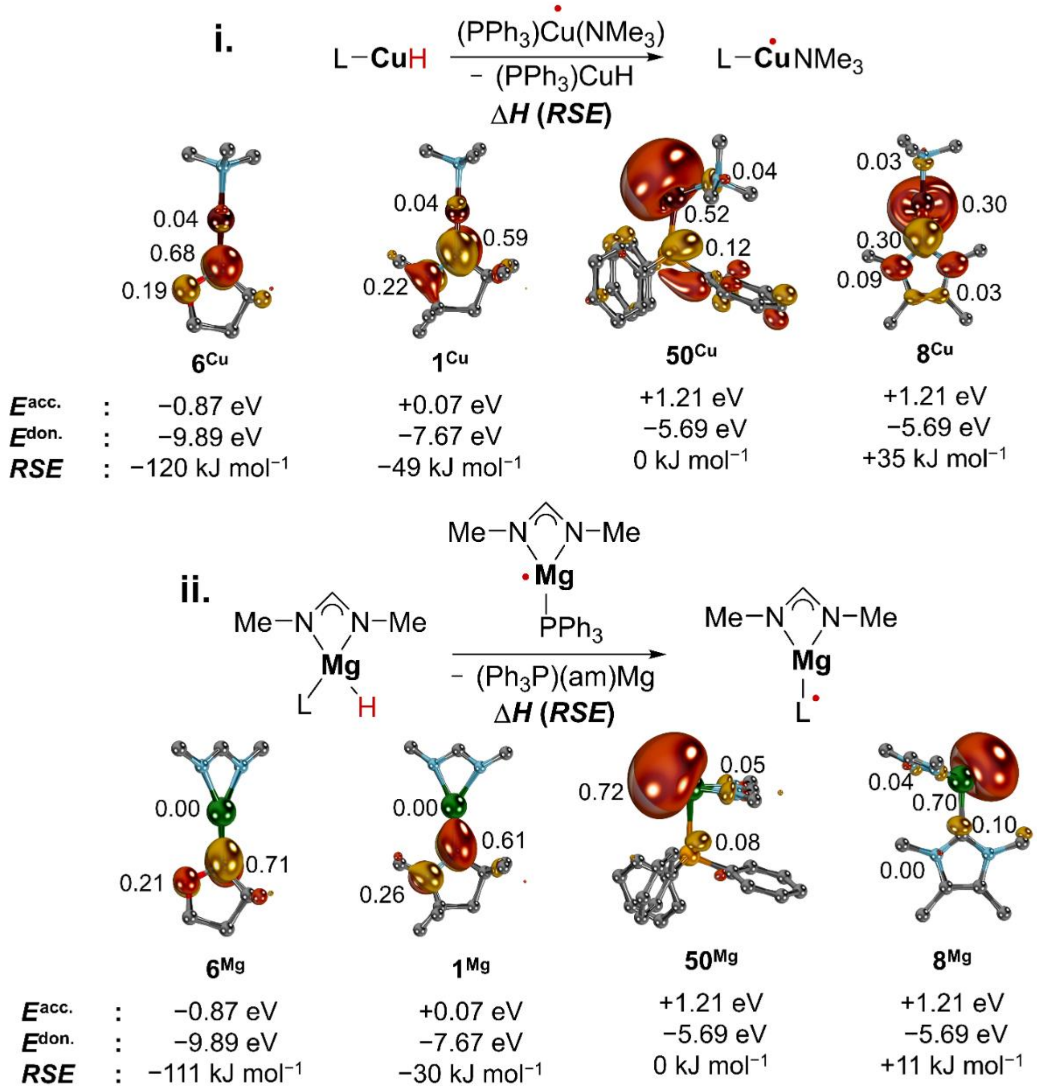

Figure S21. Combined *RSE*s and atomic spin-density values including compounds **50**^{Cu} and **50**^{Mg}.

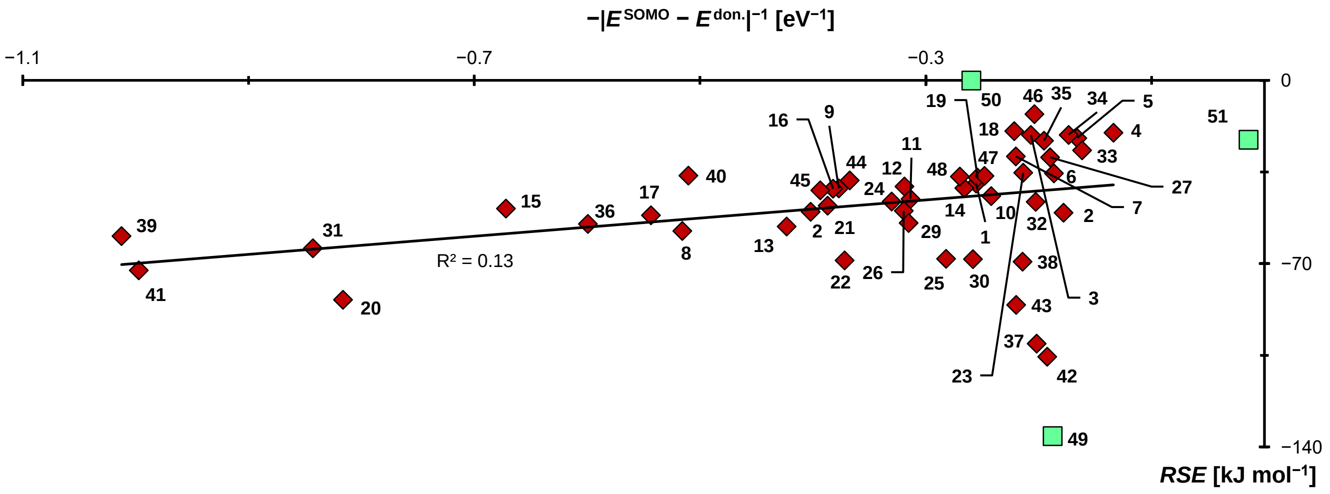

Figure S22. Fit obtained for the carbon-radicals if only considering the donor interaction.

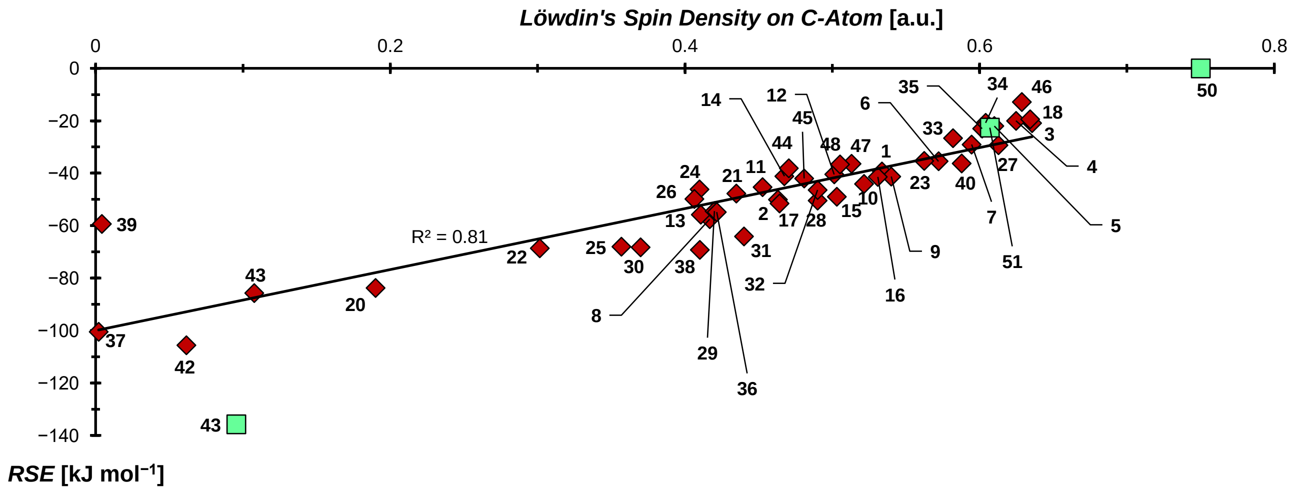

Figure S23. Löwdin's atomic spin density at the carbon atoms of the methylene substituent (red squares) for compounds **n**$^{\mathbf{C}}$ correlates with the *RSE*s (except for the redox-active oxyallyl substituent **49**, green square). The reference molecules **50**$^{\mathbf{C}}$ (PPh_3) and **51**$^{\mathbf{C}}$ (pyridine) are given in green squares as well.

6.) Comparison with the Radical Stability Score *RSS*

Paton's Radical Stability Score *RSS* (eq. S7) was calculated from the maximum spin density (Mulliken definition) S_{max} on a single atom with the sum of all spin densities normalized to 1, disregarding hydrogen atoms, and the buried volume V_{bur} around the atom with the highest spin density.[12] Buried volumes in a sphere of a radius of 3.5 Å were computed with SambVca 2.1[13] using unscaled Bondi radii and an integration grid of 0.05 Å.

$$RSS = V_{bur} + 50 \times (1 - S_{max}) \qquad \text{(S7)}$$

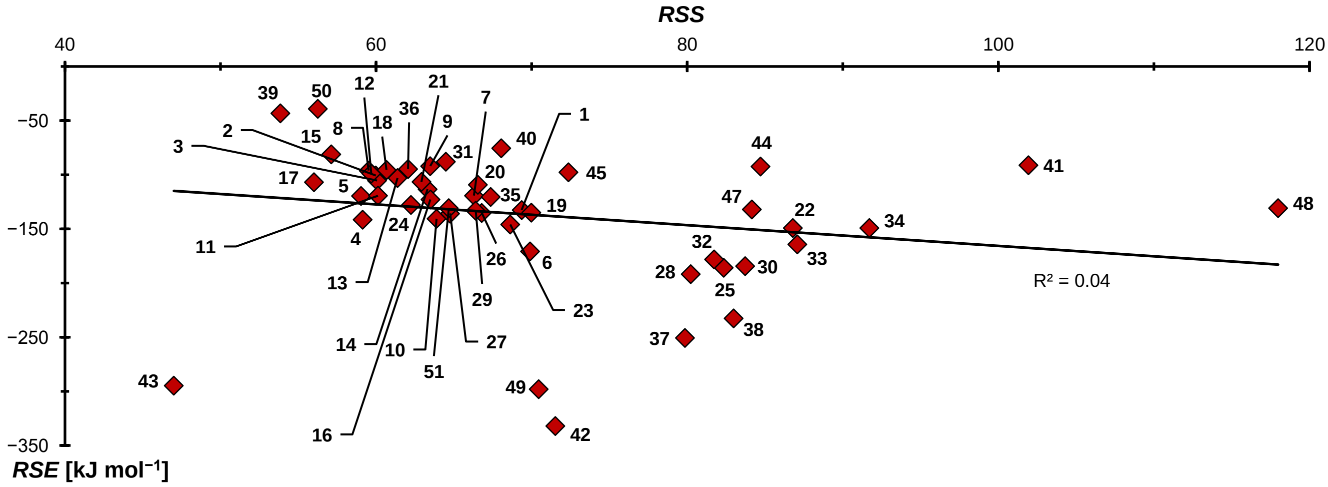

Figure S24. Correlation between *RSS* and *RSE* for boron-based radicals.

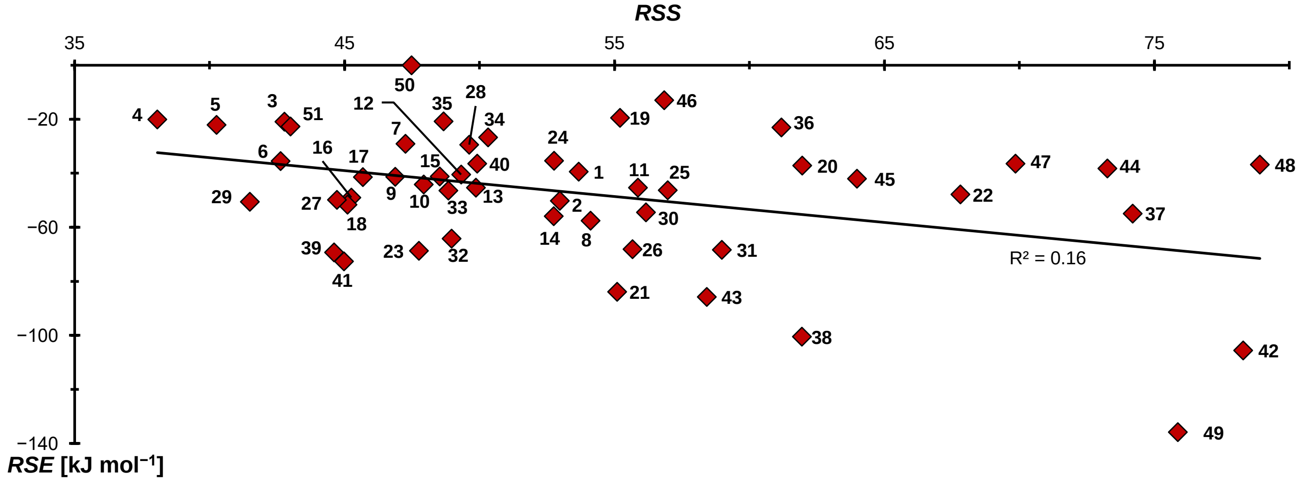

Figure S25. Correlation between *RSS* and *RSE* for carbon-based radicals.

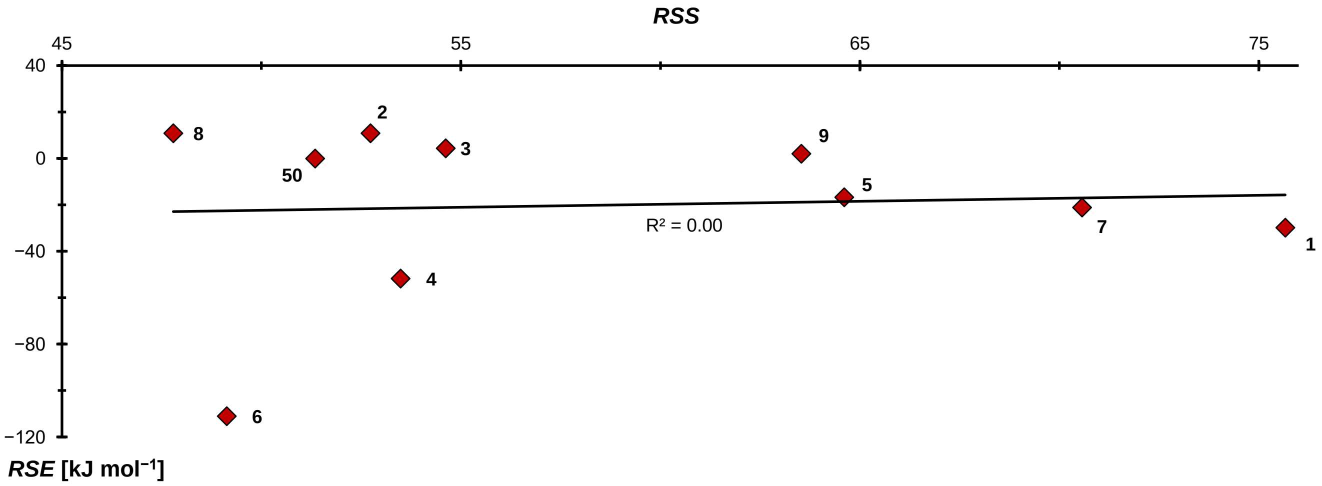

Figure S26. Correlation between *RSS* and *RSE* for magnesium-based radicals.

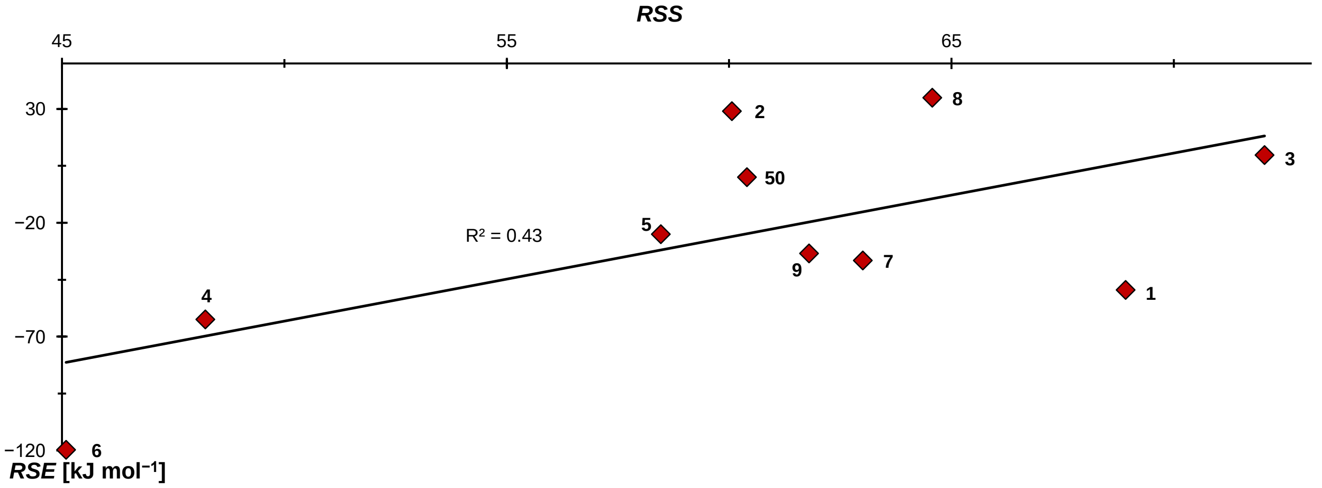

Figure S27. Correlation between *RSS* and *RSE* for copper-based radicals.

	S_{max}	Atom with highest spin density	V_{bur} [%]	*RSS*	*RSE* [kJ mol^{-1}]
**1**	0.445	27B	41.6	69.36	−132.49
**2**	0.564	15B	38.2	59.98	−100.69
**3**	0.563	17B	38.2	60.06	−105.28
**4**	0.420	9B	30.1	59.12	−141.38
**5**	0.498	11B	33.9	59.02	−119.43
**6**	0.512	1C	45.5	69.88	−170.67
**7**	0.486	19B	40.6	66.29	−119.18
**8**	0.578	15B	38.4	59.52	−96.06
**9**	0.474	17B	37.2	63.48	−91.91
**10**	0.458	14B	36.8	63.89	−140.57
**11**	0.476	11B	33.9	60.12	−119.36
**12**	0.556	14B	37.5	59.69	−98.80
**13**	0.538	15B	38.3	61.38	−102.97
**14**	0.498	15B	38.2	63.31	−113.33
**15**	0.612	5B	37.7	57.11	−80.96
**16**	0.428	9B	34.9	63.49	−122.81
**17**	0.544	21B	33.2	56.01	−106.96
**18**	0.589	20B	40.1	60.67	−95.44
**19**	0.436	28B	41.8	69.99	−134.99
**20**	0.407	21B	36.9	66.55	−109.12
**21**	0.492	21B	37.5	62.91	−106.64
**22**	0.398	0N	56.7	86.79	−149.05
**23**	0.381	24B	37.7	68.63	−145.90
**24**	0.427	13B	33.6	62.23	−127.91
**25**	0.325	2C	48.6	82.35	−185.95
**26**	0.376	13B	35.6	66.80	−135.25
**27**	0.425	11B	36.0	64.76	−135.68
**28**	0.392	4C	49.8	80.22	−191.82
**29**	0.390	11B	35.9	66.40	−133.02
**30**	0.296	4C	48.5	83.72	−184.34
**31**	0.570	24B	43.0	64.48	−87.85
**32**	0.367	0N	50.1	81.73	−178.05
**33**	0.355	1C	54.8	87.07	−164.22
**34**	0.382	2C	60.8	91.70	−149.23
**35**	0.469	25B	40.8	67.36	−120.48
**36**	0.517	35B	37.9	62.05	−94.66
**37**	0.487	6C	54.2	79.86	−250.50
**38**	0.504	1C	58.2	82.99	−232.43
**39**	0.847	3B	46.2	53.84	−43.19
**40**	0.777	11C	56.9	68.05	−75.51
**41**	0.345	19C	69.2	101.93	−91.02
**42**	0.524	10C	47.7	71.52	−332.10
**43**	0.676	10C	30.8	47.00	−294.82

**44**	0.482	31B	58.8	84.71	−92.18
**45**	0.517	47B	48.2	72.36	−97.66
**46**	0.539	49B	48.8	71.84	−93.68
**47**	0.391	49B	53.7	84.16	−131.83
**48**	0.370	8C	86.5	117.98	−130.80
**49**	0.399	33B	40.4	70.45	−298.09
**50**	0.737	1B	43.1	56.25	−38.92
**51**	0.430	0C	36.2	64.68	−130.64

Table S1. Parameters for the comparison of *RSS* and *RSE* for boron-based radicals.

	S_{max}	**Atom with highest spin density**	V_{bur} **[%]**	***RSS***	***RSE* [kJ mol^{-1}]**
**1**	0.735	27C	40.4	53.67	−39.38
**2**	0.665	13C	36.2	52.97	−50.13
**3**	0.859	17C	35.7	42.77	−20.86
**4**	0.783	9C	27.2	38.07	−20.00
**5**	0.821	9C	31.3	40.26	−22.04
**6**	0.729	11C	29.1	42.63	−35.50
**7**	0.825	19C	38.5	47.26	−29.04
**8**	0.646	15C	36.4	54.11	−57.54
**9**	0.758	17C	34.8	46.88	−41.27
**10**	0.743	14C	35.1	47.93	−44.16
**11**	0.631	11C	31.4	49.86	−45.29
**12**	0.720	17C	35.3	49.31	−40.47
**13**	0.609	13C	36.3	55.87	−45.29
**14**	0.669	13C	36.2	52.75	−55.83
**15**	0.744	21C	35.7	48.52	−41.12
**16**	0.743	9C	32.4	45.25	−48.95
**17**	0.698	21C	30.6	45.68	−41.41
**18**	0.870	20C	38.6	45.11	−51.57
**19**	0.714	24C	40.9	55.20	−19.42
**20**	0.459	21C	34.9	61.96	−37.08
**21**	0.626	21C	36.4	55.10	−83.78
**22**	0.444	25C	40.0	67.82	−47.81
**23**	0.759	21C	35.7	47.76	−68.69
**24**	0.579	17C	31.7	52.76	−35.26
**25**	0.485	13C	31.2	56.97	−46.20
**26**	0.571	17C	34.2	55.67	−68.08
**27**	0.778	13C	33.6	44.72	−49.84
**28**	0.636	9C	31.4	49.62	−29.39
**29**	0.848	11C	33.9	41.49	−50.48
**30**	0.497	7C	31.0	56.16	−54.45
**31**	0.634	24C	40.7	58.98	−68.31
**32**	0.705	14C	34.2	48.97	−64.15
**33**	0.749	15C	36.3	48.84	−46.39
**34**	0.784	24C	39.5	50.31	−26.70

**35**	0.819	25C	39.6	48.67	−20.79
**36**	0.500	35C	36.2	61.19	−23.02
**37**	0.260	1C	37.2	74.20	−54.83
**38**	0.525	0C	38.2	61.94	−100.48
**39**	0.988	3C	44.0	44.61	−69.28
**40**	0.820	23C	40.9	49.91	−36.35
**41**	0.994	27C	44.7	44.98	−72.51
**42**	0.392	10C	47.9	78.29	−105.58
**43**	0.452	10C	31.0	58.41	−85.78
**44**	0.663	31C	56.4	73.26	−38.17
**45**	0.668	47C	47.4	63.98	−41.98
**46**	0.835	49C	48.6	56.85	−12.85
**47**	0.681	49C	53.9	69.86	−36.43
**48**	0.672	49C	62.5	78.91	−36.72
**49**	0.389	3O	45.3	75.86	−135.82
**50**	0.910	1C	43.0	47.48	0.00
**51**	0.756	11C	30.8	43.00	−22.64

Table S2. Parameters for the comparison of *RSS* and *RSE* for carbon-based radicals.

	**S_{max}**	**Atom with highest spin density**	**V_{bur} [%]**	***RSS***	***RSE* [kJ mol^{-1}]**
**1**	0.864	4C	62.0	68.81	−49.50
**2**	0.789	13Cu	49.4	59.96	29.01
**3**	0.573	17Cu	50.6	71.94	9.64
**4**	0.792	1C	37.7	48.12	−62.44
**5**	0.725	4C	44.6	58.37	−25.11
**6**	0.910	8C	40.5	44.99	−119.69
**7**	0.864	0C	56.1	62.91	−36.58
**8**	0.709	0Cu	49.9	64.47	34.87
**9**	0.626	2N	43.0	61.70	−33.45
**50**	0.822	0Cu	51.4	60.30	0.00

Table S3. Parameters for the comparison of *RSS* and *RSE* for copper-based radicals.

	**S_{max}**	**Atom with highest spin density**	**V_{bur} [%]**	***RSS***	***RSE* [kJ mol^{-1}]**
**1**	0.779	2C	64.6	75.66	−29.80
**2**	0.881	13Mg	46.8	52.73	10.92
**3**	0.848	17Mg	47.0	54.62	4.46
**4**	0.748	1C	40.9	53.49	−51.66
**5**	0.666	4C	47.9	64.61	−16.72
**6**	0.893	5C	43.8	49.13	−110.96
**7**	0.775	0C	59.3	70.57	−21.05
**8**	0.970	0Mg	46.3	47.79	10.84
**9**	0.585	2N	42.8	63.53	2.06
**50**	0.903	1Mg	46.5	51.34	0.00

Table S4. Parameters for the comparison of *RSS* and *RSE* for magnesium-based radicals.

7.) Energies

	E [Eh]	*H* [Eh]	*G* [Eh]	*E* [Eh]
	def2-SVP	def2-SVP	def2-SVP	def2-TZVPP
**Methane**				
Radical	−39.778651	−39.745233	−39.769116	−39.828741
Hydrogenated	−40.453833	−40.405657	−40.429163	−40.504783
1				
Radical	−433.409709	−433.129127	−433.180747	−433.169816
Hydrogenated	−434.023182	−433.733133	−433.785246	−433.790104
**2**				
Radical	−330.442202	−330.283940	−330.326611	−330.259379
Hydrogenated	−331.067782	−330.899428	−330.942460	−330.892405
**3**				
Radical	−331.639569	−331.457682	−331.501540	−331.462575
Hydrogenated	−332.265763	−332.073841	−332.118205	−332.093797
**4**				
Radical	−292.798710	−292.700417	−292.736664	−292.689800
Hydrogenated	−293.410127	−293.302420	−293.339411	−293.306648
**5**				
Radical	−312.219444	−312.079386	−312.119554	−312.076127
Hydrogenated	−312.839208	−312.689224	−312.730023	−312.701849
**6**				
Radical	−256.928666	−256.806733	−256.843496	−256.802916
Hydrogenated	−257.529586	−257.398958	−257.436046	−257.407890
**7**				
Radical	−332.820695	−332.616874	−332.663770	−332.646287
Hydrogenated	−333.440004	−333.225745	−333.271955	−333.272615
**8**				
Radical	−408.978144	−408.761200	−408.810827	−408.750589
Hydrogenated	−409.605195	−409.378199	−409.428291	−409.385338
**9**				
Radical	−385.686757	−385.511670	−385.558919	−385.487087
Hydrogenated	−386.313226	−386.127744	−386.174139	−386.123759

**10**				
Radical	−238.283457	−238.127306	−238.169133	−238.148542
Hydrogenated	−238.894649	−238.729119	−238.770928	−238.765664
**11**				
Radical	−311.011916	−310.895825	−310.934719	−310.862208
Hydrogenated	−311.629676	−311.503759	−311.543369	−311.487858
**12**				
Radical	−385.730079	−385.554181	−385.600265	−385.533372
Hydrogenated	−386.355403	−386.169479	−386.216142	−386.167051
**13**				
Radical	−1249.247308	−1249.105107	−1249.154537	−1248.503568
Hydrogenated	−1249.871262	−1249.719107	−1249.768803	−1249.135585
**14**				
Radical	−514.699269	−514.539726	−514.590815	−514.441540
Hydrogenated	−515.319147	−515.149717	−515.201255	−515.069545
**15**				
Radical	−408.950998	−408.735082	−408.785756	−408.722069
Hydrogenated	−409.583226	−409.356703	−409.407092	−409.363125
**16**				
Radical	−330.402377	−330.244608	−330.286213	−330.218394
Hydrogenated	−331.020338	−330.852560	−330.894534	−330.842915
**17**				
Radical	−408.873616	−408.657946	−408.711321	−408.642590
Hydrogenated	−409.497485	−409.271941	−409.325813	−409.273010
**18**				
Radical	−370.895175	−370.682849	−370.729045	−370.695462
Hydrogenated	−371.523314	−371.300712	−371.347114	−371.330671
**19**				
Radical	−471.468222	−471.178450	−471.227639	−471.202802
Hydrogenated	−472.081433	−471.782145	−471.830844	−471.822188
**20**				
Radical	−558.805287	−558.607662	−558.654849	−558.455750
Hydrogenated	−559.425743	−559.217798	−559.265623	−559.085791

**21**				
Radical	−483.912696	−483.704358	−483.753525	−483.624000
Hydrogenated	−484.534520	−484.316250	−484.365734	−484.254601
**22**				
Radical	−507.137646	−506.889212	−506.939231	−506.828575
Hydrogenated	−507.744434	−507.486641	−507.536736	−507.442449
**23**				
Radical	−507.141571	−506.893198	−506.944464	−506.832322
Hydrogenated	−507.749485	−507.491615	−507.543099	−507.447534
**24**				
Radical	−464.482945	−464.316953	−464.361879	−464.227794
Hydrogenated	−465.096674	−464.921003	−464.966520	−464.850042
**25**				
Radical	−1090.855549	−1090.736591	−1090.780456	−1090.070413
Hydrogenated	−1091.449318	−1091.321825	−1091.365843	−1090.669407
**26**				
Radical	−787.384176	−787.220503	−787.266490	−786.847078
Hydrogenated	−787.994986	−787.821957	−787.868188	−787.466204
**27**				
Radical	−635.119121	−634.981807	−635.023183	−634.692109
Hydrogenated	−635.732341	−635.585624	−635.627244	−635.311118
**28**				
Radical	−938.599835	−938.507236	−938.546235	−937.923101
Hydrogenated	−939.193700	−939.092700	−939.131855	−938.519726
**29**				
Radical	−633.915933	−633.802332	−633.842343	−633.484429
Hydrogenated	−634.528207	−634.405354	−634.445716	−634.104303
**30**				
Radical	−937.388448	−937.319722	−937.357560	−936.708675
Hydrogenated	−937.982718	−937.905491	−937.943555	−937.308252
**31**				
Radical	−734.733345	−734.494489	−734.548463	−734.208496
Hydrogenated	−735.364361	−735.115372	−735.169669	−734.846451

**32**				
Radical	−350.294221	−350.148486	−350.191272	−350.122293
Hydrogenated	−350.889404	−350.734704	−350.777347	−350.724726
**33**				
Radical	−479.555733	−479.409048	−479.455182	−479.353147
Hydrogenated	−480.156800	−480.001214	−480.048844	−479.960785
**34**				
Radical	−597.343150	−597.107764	−597.161215	−597.079525
Hydrogenated	−597.950029	−597.705352	−597.760448	−597.693263
**35**				
Radical	−523.387544	−523.134443	−523.185988	−523.136148
Hydrogenated	−524.005424	−523.742603	−523.794179	−523.761263
**36**				
Radical	−866.964319	−866.642111	−866.702161	−866.414843
Hydrogenated	−867.589296	−867.256844	−867.316842	−867.050317
**37**				
Radical	−525.571216	−525.372938	−525.420219	−525.210658
Hydrogenated_intfreq	−526.143440	−525.938092	−525.984365	−525.783602
**38**				
Radical	−526.750578	−526.529419	−526.578106	−526.399152
Hydrogenated	−527.331191	−527.102500	−527.150481	−526.979441
**39**				
Radical	−985.560218	−985.290727	−985.351184	−984.798402
Hydrogenated	−986.210452	−985.929397	−985.989171	−985.454801
**40**				
Radical	−559.143520	−558.915599	−558.970159	−558.902273
Hydrogenated	−559.780054	−559.541284	−559.596825	−559.545647
**41**				
Radical	−979.871440	−979.470377	−979.541091	−979.291049
Hydrogenated	−980.492156	−980.080003	−980.149631	−979.928758
**42**				
Radical	−480.237310	−480.101417	−480.149502	−479.963321
Hydrogenated	−480.781083	−480.639147	−480.686642	−480.504160

**43**				
Radical	−295.973327	−295.839380	−295.878828	−295.771785
Hydrogenated	−296.531209	−296.389778	−296.428805	−296.328262
**44**				
Radical	−1184.609487	−1183.986126	−1184.076666	−1183.917496
Hydrogenated	−1185.234441	−1184.600776	−1184.691274	−1184.553977
**45**				
Radical	−949.047028	−948.604053	−948.681808	−948.478147
Hydrogenated	−949.672025	−949.217992	−949.297798	−949.113293
**46**				
Radical	−950.243415	−949.775678	−949.857006	−949.678382
Hydrogenated	−950.869890	−950.392077	−950.472736	−950.314060
**47**				
Radical	−860.491621	−859.977942	−860.052377	−859.997087
Hydrogenated	−861.103675	−860.580332	−860.654049	−860.617825
**48**				
Radical	−1134.119275	−1133.419216	−1133.507350	−1133.472889
Hydrogenated	−1134.730348	−1134.020301	−1134.107522	−1134.094343
**49**				
Radical	−711.279060	−710.952792	−711.014209	−710.935200
Hydrogenated	−711.826330	−711.492305	−711.554461	−711.490704
**50**				
Radical	−1061.262625	−1060.949362	−1061.012435	−1060.490593
Hydrogenated	−1061.915030	−1061.591078	−1061.653099	−1061.147740
**51**				
Radical	−273.977889	−273.860448	−273.897084	−273.806844
Hydrogenated	−274.591830	−274.464450	−274.500041	−274.428308

Table S5. Energies of boryl-radicals and their hydrogenated forms.

	E **[Eh]**	***H*** **[Eh]**	**G [Eh]**	***E*** **[Eh]**
	def2-SVP	def2-SVP	def2-SVP	def2-TZVPP
**Methane**				
Methane	−40.453845	−40.405653	−40.429159	−40.438443
Methyl Radical	−39.778558	−39.745135	−39.769014	−39.761512
**1**				

Radical	−446.424189	−446.138481	−446.189609	−446.188454
NHO	−447.076252	−446.777145	−446.828985	−446.849020
**2**				
Radical	−343.467908	−343.304014	−343.345937	−343.288365
NHO	−344.115678	−343.938418	−343.982434	−343.944799
**3**				
Radical	−344.653934	−344.466504	−344.511019	−344.479629
NHO	−345.314436	−345.113269	−345.158433	−345.147586
**4**				
Radical	−305.777211	−305.673764	−305.710050	−305.673494
NHO	−306.437720	−306.321085	−306.358190	−306.341228
**5**				
Radical	−325.221211	−325.075829	−325.116144	−325.082675
NHO	−325.881108	−325.722120	−325.762676	−325.750051
**6**				
Radical	−269.906865	−269.780189	−269.816514	−269.785575
NHO	−270.561364	−270.421931	−270.458919	−270.446935
**7**				
Radical	−345.838866	−345.629278	−345.675369	−345.667350
NHO	−346.494953	−346.271400	−346.317522	−346.332375
**8**				
Radical	−422.014878	−421.792385	−421.841665	−421.790222
NHO	−422.659873	−422.423975	−422.474112	−422.443875
**9**				
Radical	−398.718152	−398.537525	−398.583299	−398.525819
NHO	−399.370867	−399.176487	−399.222654	−399.186018
**10**				
Radical	−251.289223	−251.127887	−251.168945	−251.159610
NHO	−251.940526	−251.765886	−251.807592	−251.818260
**11**				
Radical	−324.017095	−323.895753	−323.933909	−323.871785
NHO	−324.666263	−324.531507	−324.570968	−324.530112
**12**				

Radical	−398.743945	−398.563135	−398.609260	−398.551435
NHO	−399.395493	−399.200983	−399.247817	−399.211887
**13**				
Radical	−1262.266669	−1262.119213	−1262.167763	−1261.525549
NHO	−1262.911321	−1262.750495	−1262.802038	−1262.179817
**14**				
Radical	−527.690131	−527.525634	−527.576266	−527.436112
NHO	−528.339577	−528.162563	−528.212452	−528.095132
**15**				
Radical	−422.010459	−421.788278	−421.838382	−421.786514
NHO	−422.659966	−422.424013	−422.474070	−422.443805
**16**				
Radical	−343.431089	−343.267666	−343.309903	−343.251373
NHO	−344.083565	−343.906445	−343.948782	−343.911463
**17**				
Radical	−421.915407	−421.693745	−421.746175	−421.686511
NHO	−422.562088	−422.327064	−422.380330	−422.342397
**18**				
Radical	−383.919104	−383.701229	−383.747035	−383.723526
NHO	−384.579767	−384.348073	−384.393996	−384.392112
**19**				
Radical	−484.486411	−484.191653	−484.241178	−484.221975
NHO	−485.136629	−484.828455	−484.878316	−484.883432
**20**				
Radical	−571.843536	−571.639865	−571.686499	−571.497337
Hydrogenated	−572.478046	−572.261303	−572.308924	−572.140661
**21**				
Radical	−496.933492	−496.719769	−496.768179	−496.648410
NHO	−497.581000	−497.353723	−497.403238	−497.305917
**22**				
Radical	−520.152753	−519.899083	−519.949816	−519.847355
Hydrogenated	−520.792339	−520.525622	−520.575932	−520.496403
**23**				

Radical	−520.151021	−519.897564	−519.948440	−519.845551
NHO	−520.804921	−520.538040	−520.589403	−520.507707
**24**				
Radical	−477.488800	−477.317723	−477.362149	−477.237709
NHO	−478.136457	−477.951928	−477.996945	−477.895729
**25**				
Radical	−1103.845060	−1103.721363	−1103.764492	−1103.062563
NHO	−1104.484199	−1104.347788	−1104.391783	−1103.711511
**26**				
Radical	−800.389843	−800.221155	−800.266882	−799.856295
NHO	−801.035987	−800.854057	−800.899609	−800.512716
**27**				
Radical	−648.116780	−647.974543	−648.015649	−647.693716
NHO	−648.773009	−648.617265	−648.658446	−648.358192
**28**				
Radical	−951.577228	−951.480127	−951.518383	−950.901751
NHO	−952.224235	−952.114260	−952.153271	−951.557561
**29**				
Radical	−646.920562	−646.801878	−646.841043	−646.493879
NHO	−647.566306	−647.434558	−647.474354	−647.148369
**30**				
Radical	−950.371479	−950.297957	−950.334869	−949.696061
NHO	−951.011341	−950.925182	−950.962974	−950.344842
**31**				
Radical	−747.797239	−747.552318	−747.605718	−747.274279
NHO	−748.440085	−748.181899	−748.235862	−747.925275
**32**				
Radical	−363.278384	−363.128087	−363.170144	−363.112519
NHO	−363.927235	−363.763660	−363.806509	−363.770292
**33**				
Radical	−492.514052	−492.363477	−492.409649	−492.316067
NHO	−493.170462	−493.006638	−493.054261	−492.981311
**34**				

Radical	−610.322239	−610.082931	−610.136328	−610.062905
NHO	−610.980955	−610.728201	−610.781812	−610.730596
**35**				
Radical	−536.388937	−536.131377	−536.183902	−536.141880
Hydrogenated	−537.047507	−536.776190	−536.828260	−536.809033
**36**				
Radical	−879.999870	−879.672030	−879.731479	−879.451779
Hydrogenated	−880.641193	−880.299838	−880.359796	−880.106573
**37**				
Radical	−538.562306	−538.358713	−538.405524	−538.202821
Hydrogenated	−539.191889	−538.977112	−539.022989	−538.837897
**38**				
Radical	−539.744298	−539.517956	−539.567051	−539.388470
Hydrogenated	−540.383547	−540.144421	−540.193825	−540.037031
**39**				
Radical	−998.640674	−998.364658	−998.424296	−997.878198
NHO	−999.289792	−998.999839	−999.058745	−998.531667
**40**				
Radical	−572.204398	−571.970192	−572.025660	−571.961861
NHO	−572.857333	−572.609442	−572.664783	−572.623866
**41**				
Radical	−992.938997	−992.531135	−992.600936	−992.367151
NHO	−993.579359	−993.157703	−993.227316	−993.015492
**42**				
Radical	−493.183037	−493.042112	−493.088566	−492.905853
Hydrogenated	−493.809766	−493.656782	−493.705491	−493.539864
**43**				
Radical	−308.951232	−308.811469	−308.850297	−308.748359
Hydrogenated	−309.585935	−309.433673	−309.473649	−309.390352
**44**				
Radical	−1197.649707	−1197.020655	−1197.110967	−1196.959807
Hydrogenated	−1198.298158	−1197.655696	−1197.746990	−1197.620844
**45**				

Radical	−962.088513	−961.639019	−961.718596	−961.521672
Hydrogenated	−962.736999	−962.274111	−962.354118	−962.181242
**46**				
Radical	−963.276028	−962.802769	−962.884153	−962.713030
Hydrogenated	−963.934853	−963.448930	−963.528065	−963.382961
**47**				
Radical	−873.513435	−872.994755	−873.069072	−873.022236
Hydrogenated	−874.164472	−873.632168	−873.706297	−873.684147
**48**				
Radical	−1147.146101	−1146.440945	−1146.528529	−1146.502629
Hydrogenated	−1147.795788	−1147.076929	−1147.164381	−1147.164510
**49**				
Radical	−724.266123	−723.935148	−723.995906	−723.923984
Hydrogenated	−724.880429	−724.537586	−724.599686	−724.546283
**50**				
Radical	−1074.286825	−1073.968837	−1074.032580	−1073.519476
Hydrogenated	−1074.957883	−1074.625851	−1074.688747	−1074.197181
**51**				
Radical	−286.942779	−286.820293	−286.856517	−286.775890
Hydrogenated	−287.603345	−287.466384	−287.503693	−287.443904

Table S6. Energies of carbon-radicals and their hydrogenated forms.

	***E* [Eh]**	***H* [Eh]**	***G* [Eh]**	***E* [Eh]**
	def2-SVP	def2-SVP	def2-SVP	def2-TZVPP
**MgH_2**				
Radical	−200.526029	−200.519634	−200.541555	−200.598631
Hydrogenated	−201.139949	−201.127926	−201.148496	−200.972293
**1**				
Radical	−835.151644	−834.7834535	−834.853006	−834.676925
Hydrogenated	−835.760299	−835.384428	−835.453327	−835.293059
**2**				
Radical	−732.185322	−731.937781	−731.998456	−731.771554
Hydrogenated	−732.812317	−732.558140	−732.617850	−732.402153
**3**				
Radical	−733.381978	−733.111171	−733.173117	−732.970243

Hydrogenated	−734.006353	−733.728950	−733.791430	−733.598341
**4**				
Radical	−694.548667	−694.362248	−694.417356	−694.206707
Hydrogenated	−695.149536	−694.956273	−695.012992	−694.813678
**5**				
Radical	−713.963102	−713.735071	−713.793273	−713.585978
Hydrogenated	−714.578178	−714.342775	−714.401053	−714.20679
**6**				
Radical	−658.677917	−658.467913	−658.523787	−658.319438
Hydrogenated	−659.259876	−659.043412	−659.098978	−658.903439
**7**				
Radical	−734.566310	−734.275225	−734.339936	−734.158531
Hydrogenated	−735.182590	−734.882926	−734.946256	−734.778896
**8**				
Radical	−810.723418	−810.417087	−810.483800	−810.264707
Hydrogenated	−811.350812	−811.038140	−811.104442	−810.894980
**9**				
Radical	−787.435314	−787.172259	−787.236756	−787.004989
Hydrogenated	−788.057310	−787.786046	−787.849984	−787.633788
**50**				
Radical	−1463.054120	−1462.650162	−1462.728790	−1462.041860
Hydrogenated	−1463.676070	−1463.265780	−1463.344523	−1462.667996

Table S7. Energies of magnesium radicals and their hydrogenated forms.

	***E* [Eh]**	***H* [Eh]**	***G* [Eh]**	***E* [Eh]**
	def2-SVP	def2-SVP	def2-SVP	def2-TZVPP
**1**				
NMe_3-capped Radical	−2221.865172	−2221.478712	−2221.546364	−2221.058311
Hydrogenated	−2048.217650	−2047.951290	−2048.004832	−2047.494285
**2**				
NMe_3-capped Radical	−2118.884439	−2118.619425	−2118.679413	−2118.134762
Hydrogenated	−1945.264510	−1945.119899	−1945.164874	−1944.600335
**3**				
NMe_3-capped Radical	−2120.085686	−2119.797652	−2119.858497	−2119.341578
Hydrogenated	−1946.461641	−1946.293632	−1946.339921	−1945.800149

**4**				
NMe3-capped Radical	−2081.257629	−2081.052926	−2081.106647	−2080.58523
Hydrogenated	−1907.605578	−1907.521571	−1907.560764	−1907.015676
**5**				
NMe3-capped Radical	−2100.671491	−2100.425237	−2100.482147	−2099.964217
Hydrogenated	−1927.034731	−1926.908587	−1926.951367	−1926.409468
**6**				
NMe3-capped Radical	−2045.389886	−2045.161474	−2045.215420	−2044.700310
Hydrogenated	−1871.718396	−1871.611142	−1871.650566	−1871.108493
**7**				
NMe3-capped Radical	−2121.279834	−2120.970437	−2121.033294	−2120.539384
Hydrogenated	−1947.639107	−1947.449180	−1947.497939	−1946.980907
**8**				
NMe3-capped Radical	−2197.418888	−2197.095726	−2197.162497	−2196.625142
Hydrogenated	−2023.802770	−2023.599553	−2023.651055	−2023.093407
**9**				
NMe3-capped Radical	−2174.149916	−2173.868516	−2173.930524	−2173.388669
Hydrogenated	−2000.508837	−2000.346885	−2000.395861	−1999.831405
**50**				
NMe3-capped Radical	−2849.759555	−2849.337930	−2849.416009	−2848.410393
Hydrogenated	−2676.122846	−2675.822327	−2675.887260	−2674.864213

Table S8. Energies of NMe_3-capped copper radicals and the corresponding hydrides.

8.) Canonical Molecular Orbitals of Carbenes

The canonical molecular orbitals shown below were obtained with the B3LYP/def2-TZVPP//B3LYP-D3/def2-SVP level of theory. In some cases, more than one molecular orbital features a comparatively large coefficient at the carbene position. In these cases, the orbital with the larger coefficient was chosen. These orbitals are highlighted in green in the following. The energies of the populated orbitals are in the main manuscript designated as $E^{don.}$ and the energies of the vacant orbitals as $E^{acc.}$. The fit with the radical stabilization energy *RSE* is then calculated using equation S8 (eq. 2 in the main manuscript) under assumption of $\boldsymbol{E^{SOMO}}$ **= +8.40 eV** (see Fig. S13 for E^{SOMO} = −4 eV) in case of the boryl radicals, $\boldsymbol{E^{SOMO}}$ **= −3.75 eV** for the carbon-derived radicals, $\boldsymbol{E^{SOMO}}$ **= −1.5 eV** for the magnesium complexes, and $\boldsymbol{E^{SOMO}}$ **= −0.64 eV** for the copper complexes.

$$RSE \propto -\frac{1}{|E^{SOMO} - E^{don.}|} - \frac{1}{|E^{acc.} - E^{SOMO}|} \qquad \text{(S8)}$$

1

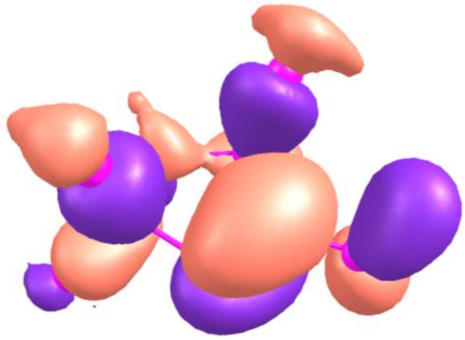

HOMO-1 (-7.66 eV)

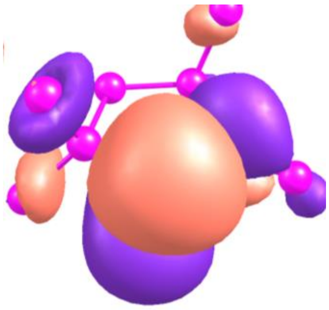

LUMO (+0.07 eV)

2

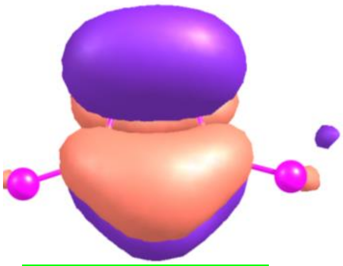

HOMO−1 (−6.24 eV)

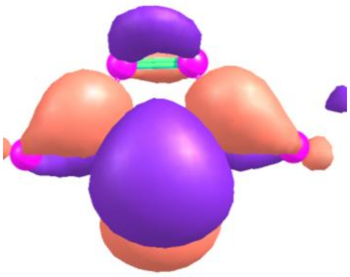

LUMO+1 (+1.01 eV)

3

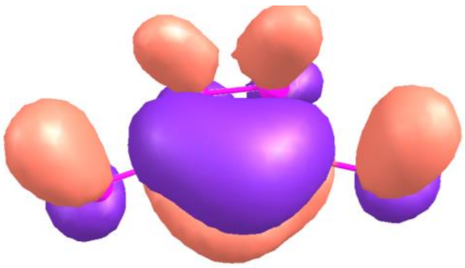

HOMO−2 (−8.58 eV)

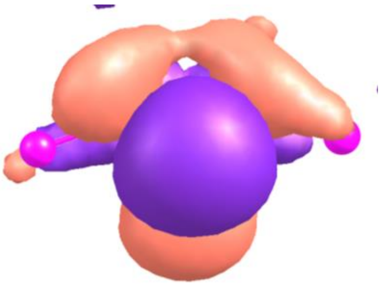

LUMO (0.78 eV)

4

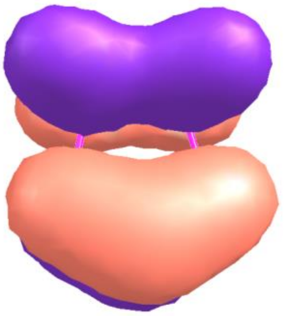

HOMO−4 (−11.22 eV)

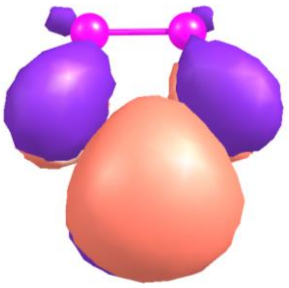

LUMO (−0.23 eV)

5

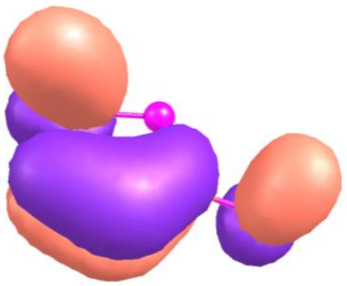

HOMO−3 (−9.78 eV)

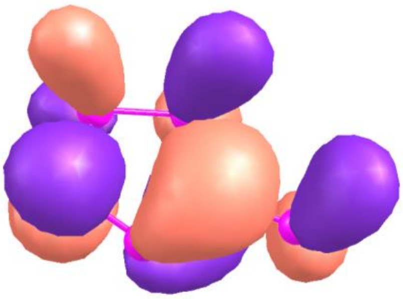

HOMO−1 (−7.02 eV)

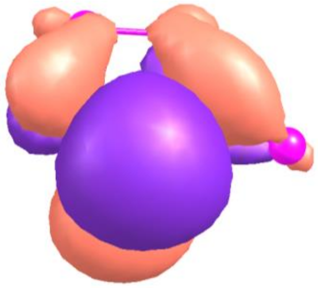

LUMO (0.40 eV)

6

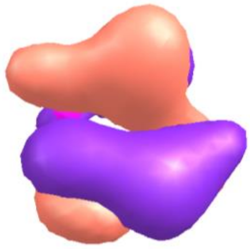

HOMO−4 (−9.89 eV)

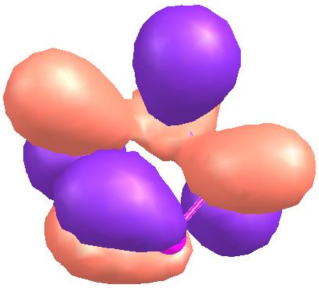

HOMO−1 (−9.11)

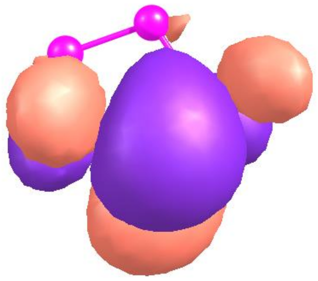

LUMO+2 (−0.87 eV)

7

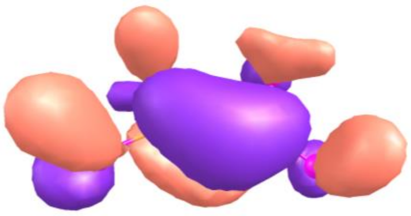

HOMO−2 (−8.29 eV)

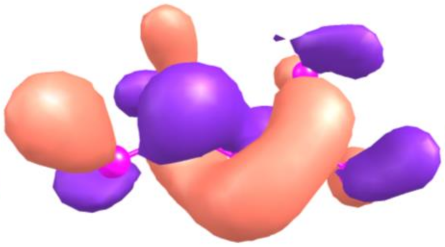

HOMO−1 (−6.25 eV)

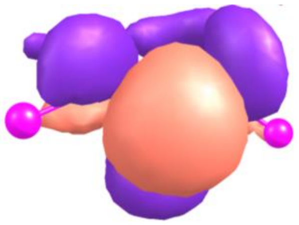

LUMO+1 (+0.84 eV)

8

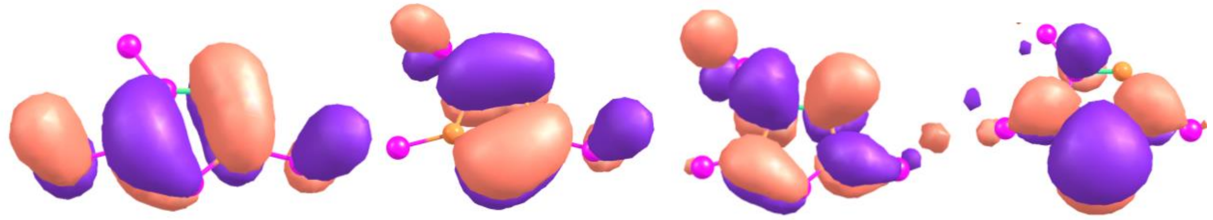

HOMO−2 (−7.76 eV) **HOMO−1 (−6.89 eV)** **LUMO (+0.46 eV)** **LUMO+2 (+0.84 eV)**

9

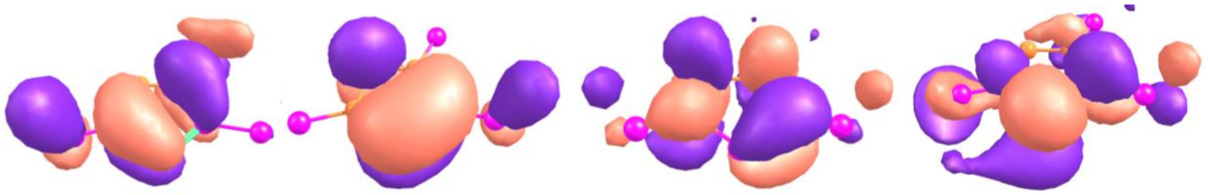

HOMO−2 (−7.90 eV) **HOMO−1 (−6.40)** **LUMO (−0.71)** **LUMO+3 (1.63)**

10

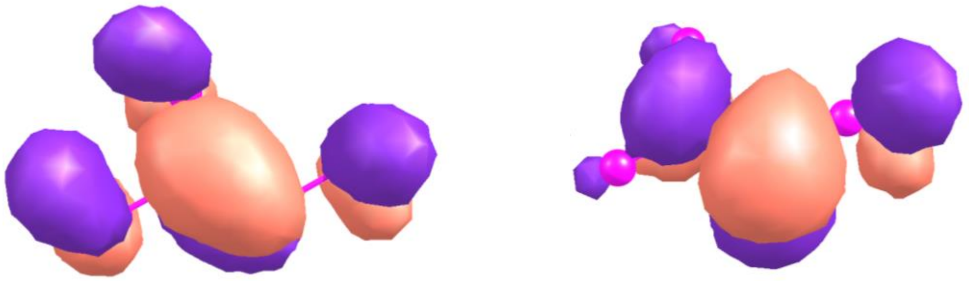

HOMO−1 (−7.88 eV) **LUMO (+0.04 eV)**

11

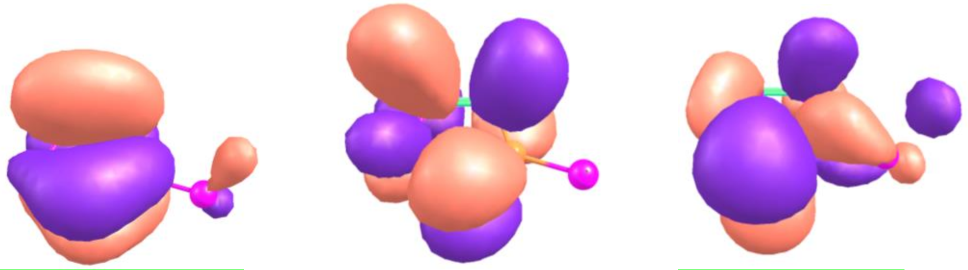

HOMO−1 (−6.94 eV) **LUMO (0.03 eV)** **LUMO+1 (0.39 eV)**

12

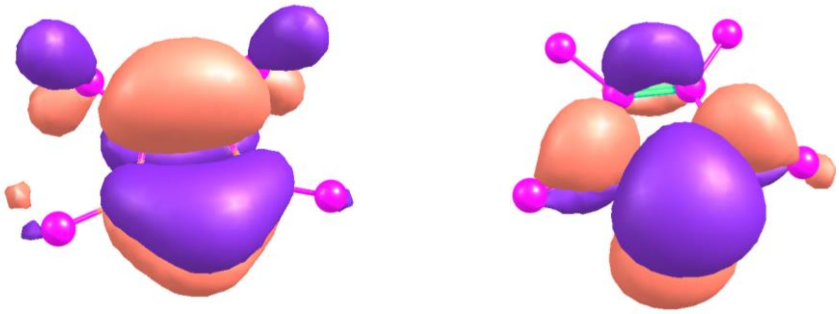

HOMO−1 (−5.69 eV) **LUMO+3 (+1.21 eV)**

13

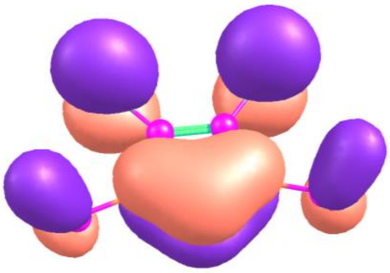

HOMO−5 (−9.61 eV)

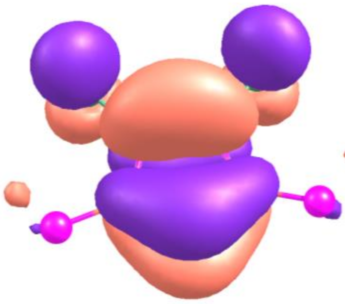

HOMO−1 (−6.11 eV)

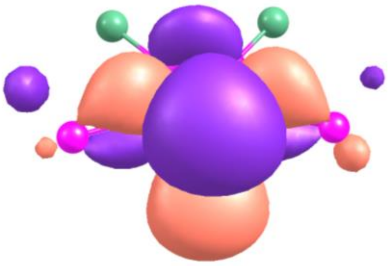

LUMO+2 (+0.57 eV)

14

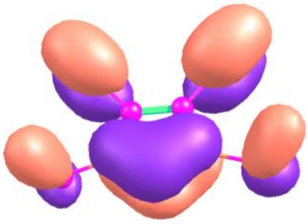

HOMO−5 (−10.37 eV)

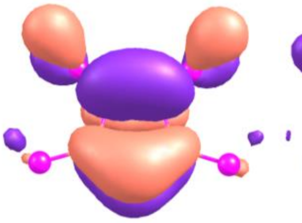

HOMO−1 (−7.51 eV)

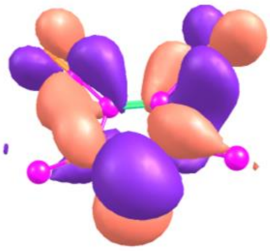

LUMO+2 (−0.42 eV)

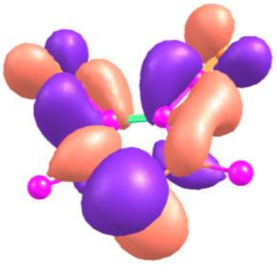

LUMO+3 (−0.36 eV)

15

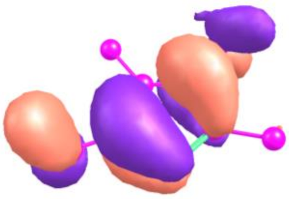

HOMO−2 (−7.35 eV)

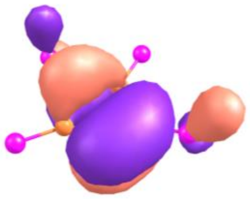

HOMO−1 (−5.24 eV)

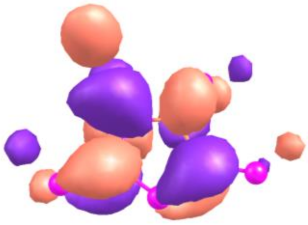

LUMO (0.04 eV)

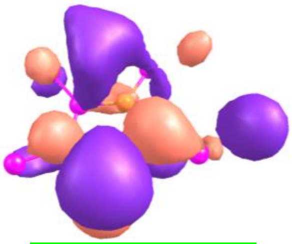

LUMO+5 (2.12 eV)

16

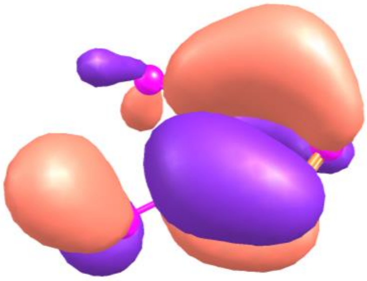

HOMO−2 (−7.14 eV)

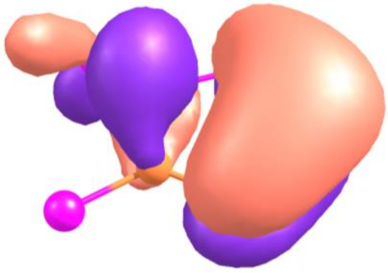

HOMO−1 (−6.37 eV)

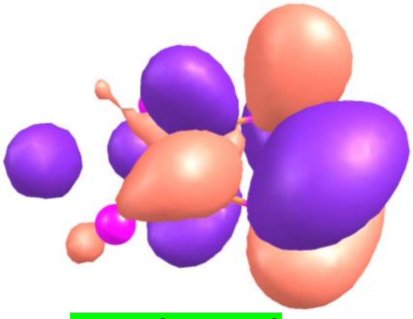

LUMO (−0.16 eV)

17

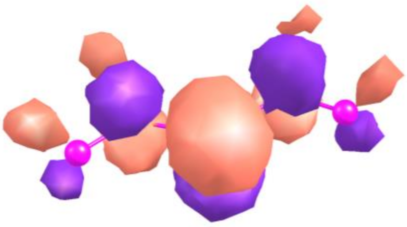

HOMO−1 (−5.59 eV)

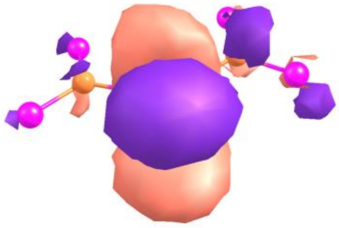

LUMO (+1.05 eV)

18

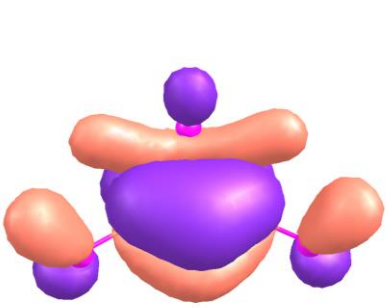

HOMO−2 (−8.26 eV)

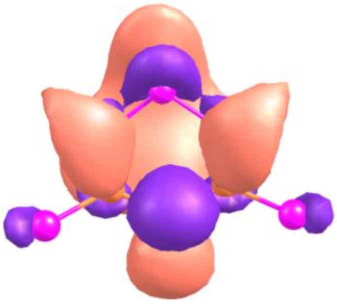

LUMO (0.55 eV)

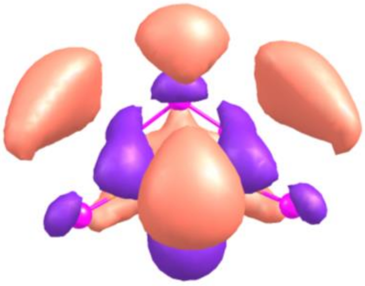

LUMO+1 (1.05 eV)

19

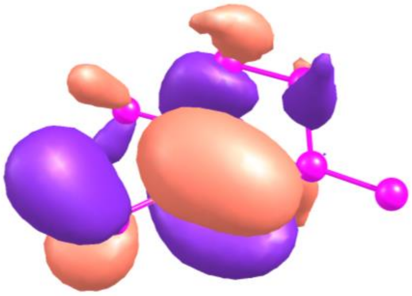

HOMO−1 (−7.68 eV)

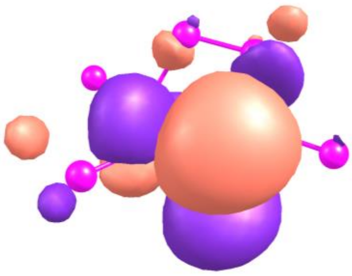

LUMO (0.00 eV)

20

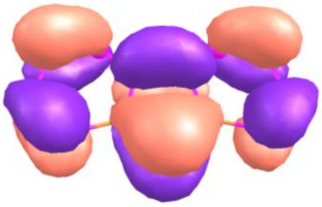

HOMO (−4.98 eV)

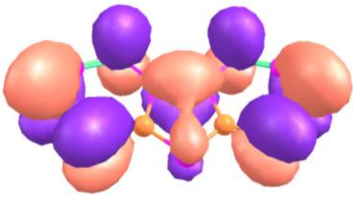

LUMO (−1.36 eV)

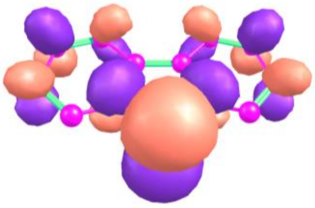

LUMO+2 (+0.40 eV)

21

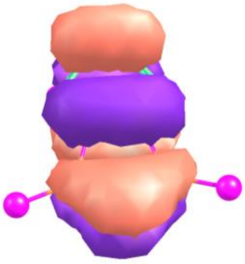

HOMO−1 (−6.33 eV)

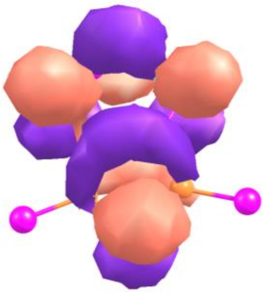

LUMO (−0.71 eV)

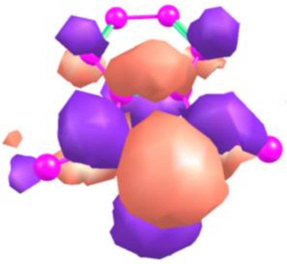

LUMO+3 (+1.12 eV)

22

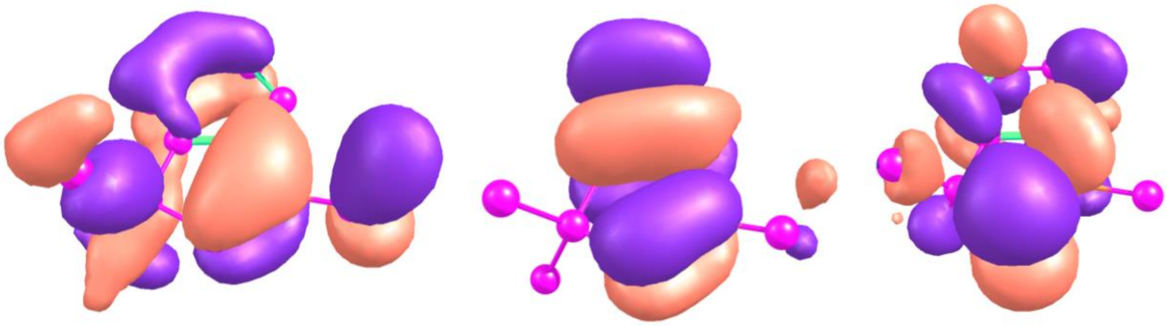

HOMO−4 (−8.88 eV) HOMO−1 (−6.44 eV) LUMO (−0.95 eV)

23

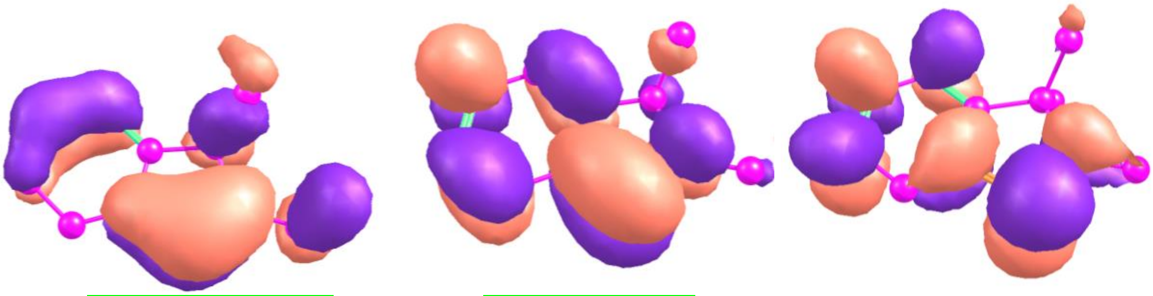

HOMO−3 (−8.43 eV) LUMO (−1.27 eV) LUMO+3 (1.13 eV)

24

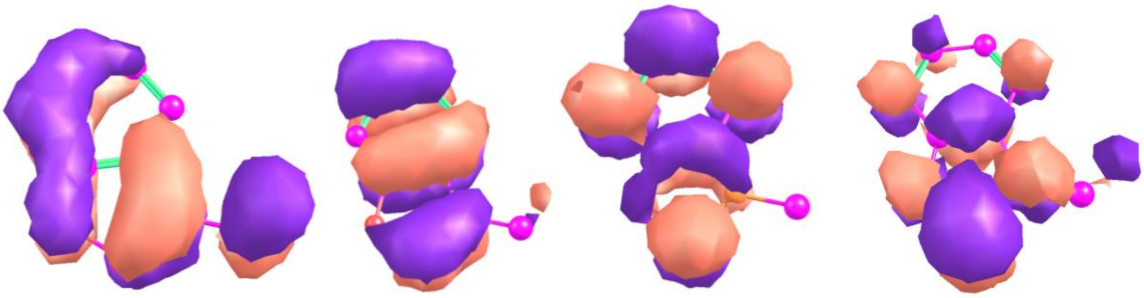

HOMO−3 (−9.08 eV) HOMO−1 (−6.78 eV) LUMO (−1.05 eV) LUMO+2 (+0.46 eV)

25

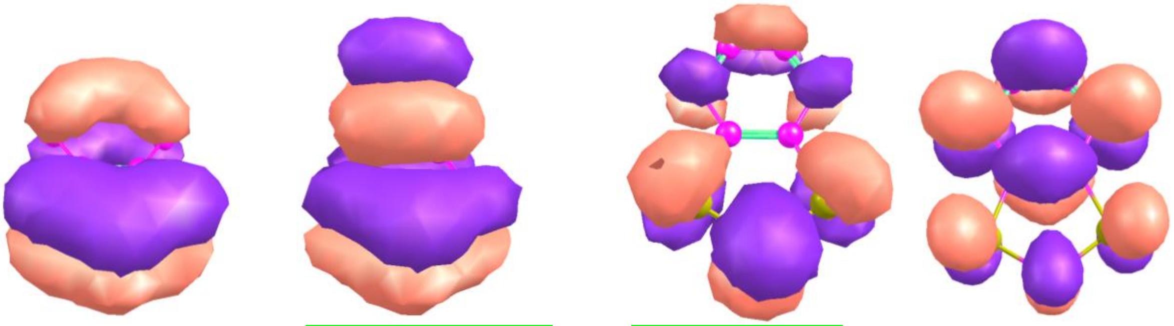

HOMO−5 (−9.67 eV) HOMO−2 (−7.30 eV) LUMO (−1.83 eV) LUMO+1 (−0.93 eV)

26

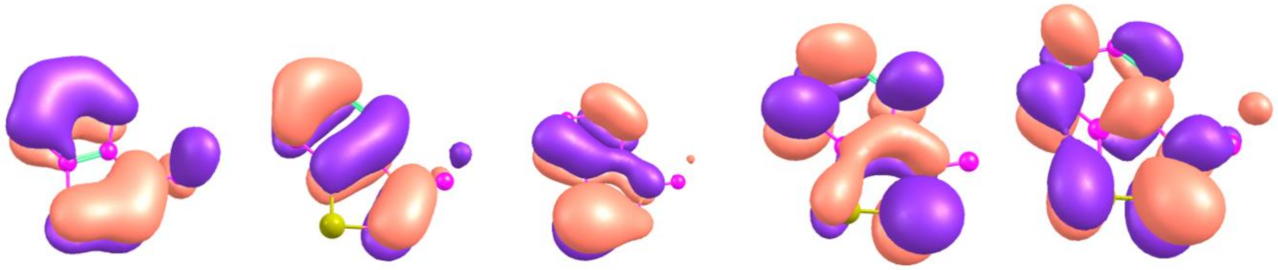

HOMO−5 (−9.49 eV) HOMO−2 (−6.88 eV) HOMO−1 (−6.71 eV) LUMO (−1.1463 eV) LUMO+2 (+0.09 eV)

27

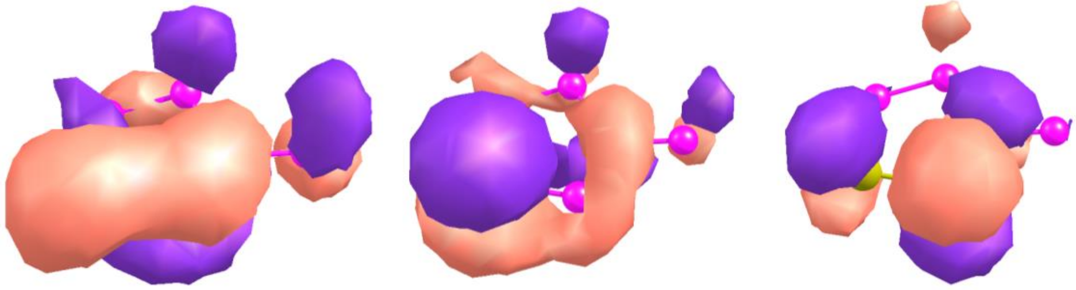

HOMO−3 (−9.02 eV) HOMO−1 (−6.40 eV) LUMO (−0.27 eV)

28

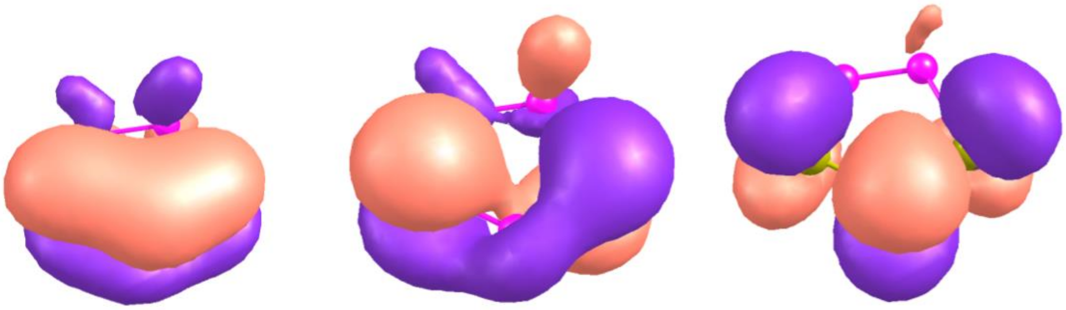

HOMO−3 (−9.37 eV) HOMO−1 (−6.92 eV) LUMO (−1.66 eV)

29

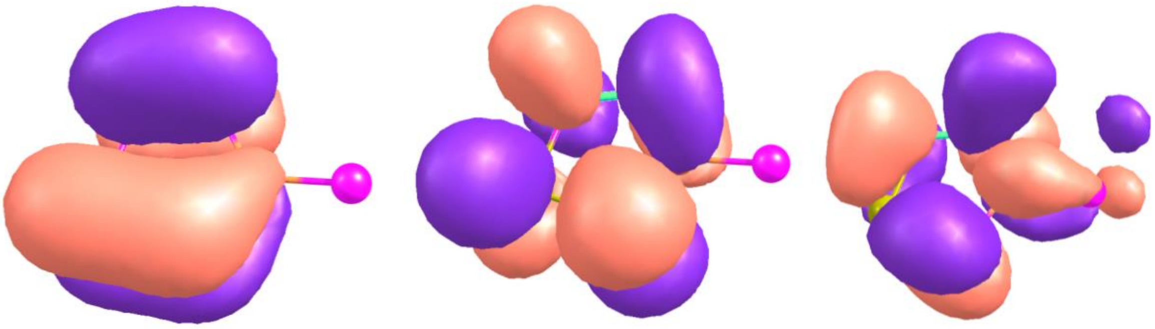

HOMO−1 (−6.92 eV) LUMO (−0.52 eV) LUMO+2 (+0.07 eV)

30

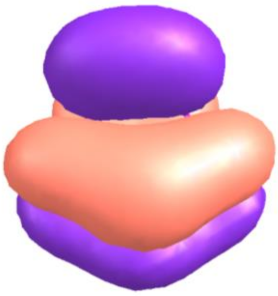
HOMO−1 (−7.62 eV)

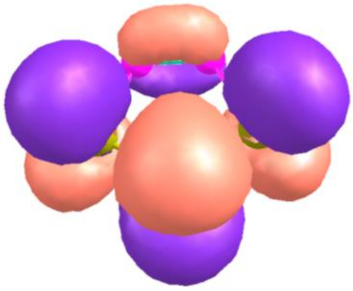
LUMO (−1.64 eV)

31

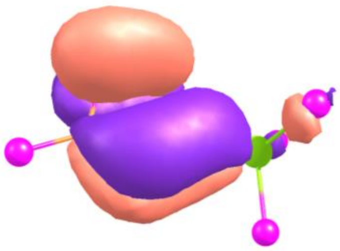
HOMO−1 (−4.94 eV)

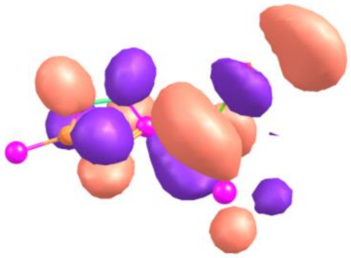
LUMO+1 (0.82 eV)

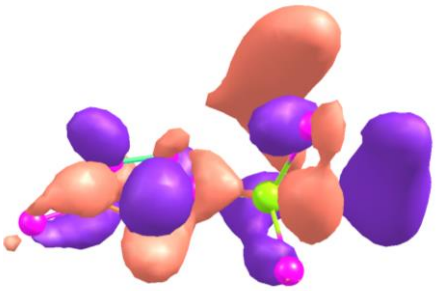
LUMO+4 (1.50 eV)

32

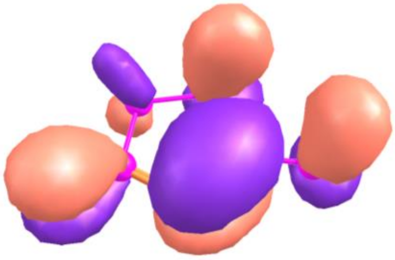
HOMO−2 (−8.68 eV)

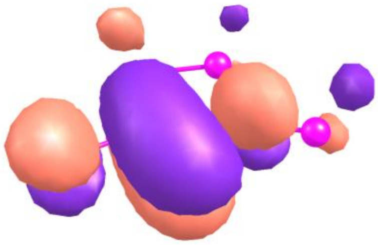
LUMO (−2.05 eV)

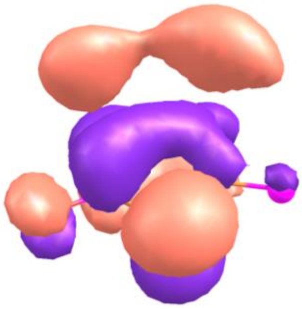
LUMO+2 (0.82 eV)

33

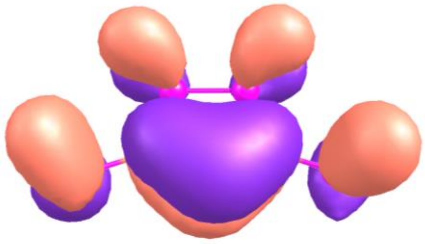
HOMO−4(−9.94 eV)

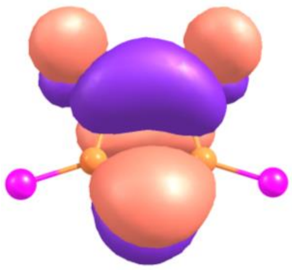
LUMO (−3.14 eV)

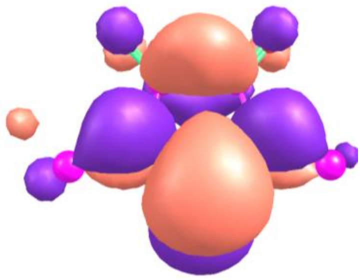
LUMO+1 (0.10 eV)

34

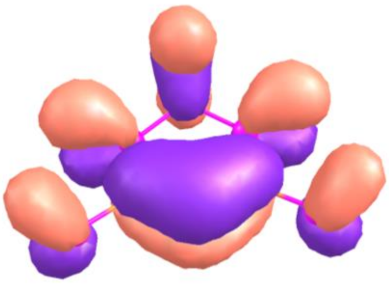

HOMO−4 (−9.51 eV)

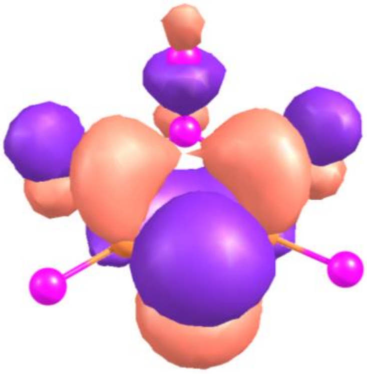

LUMO (−1.94 eV)

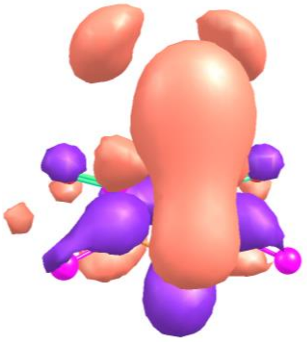

LUMO+3 (0.87 eV)

35

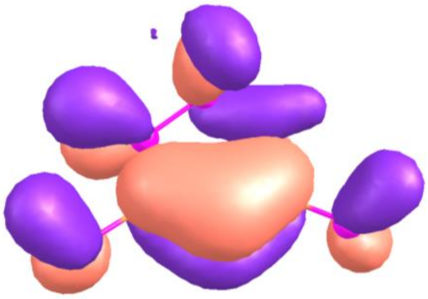

HOMO−3 (−8.87 eV)

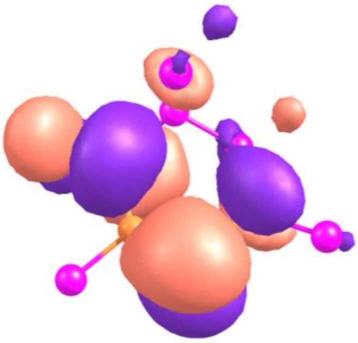

LUMO (−0.79 eV)

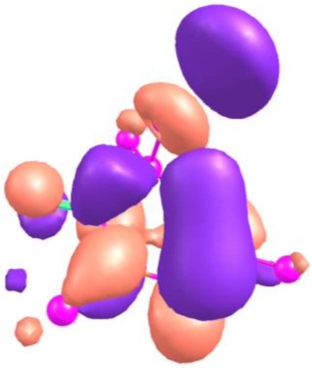

LUMO+2 (1.00 eV)

36

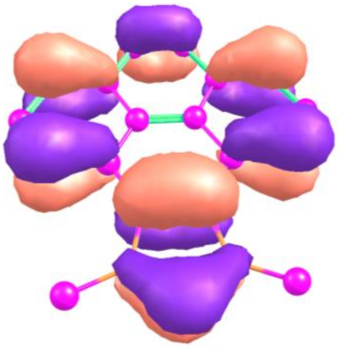

HOMO (−5.42 eV)

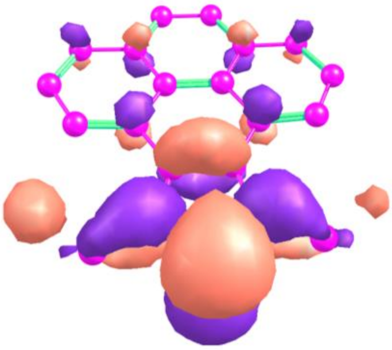

LUMO+3 (1.04 eV)

37 (triplet eV)

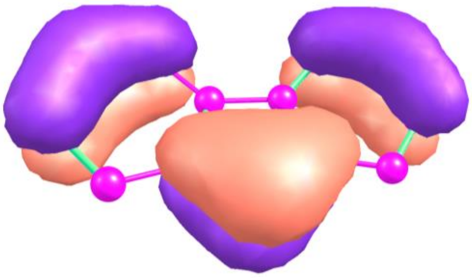

HOMO−4 (a, −8.71 eV)

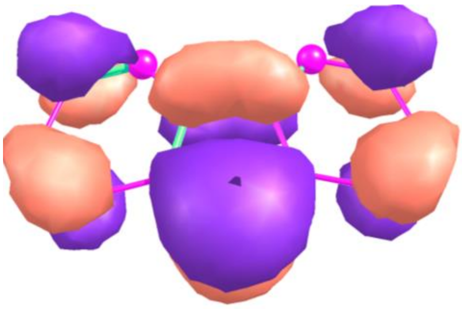

LUMO 8 (b, −3.03 eV)

38 (triplet eV)

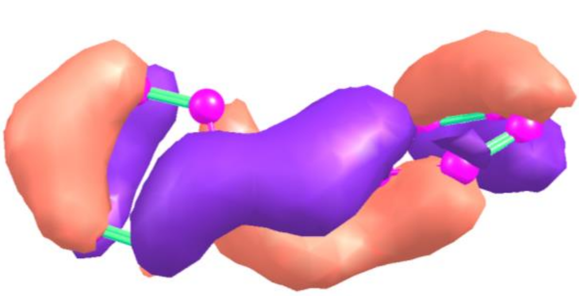
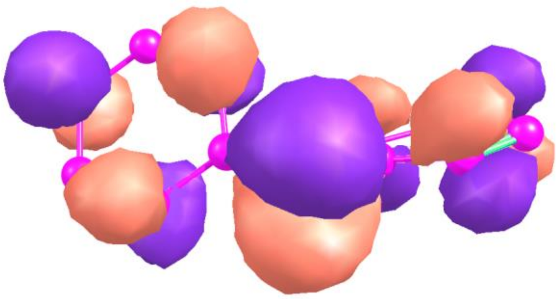

HOMO−4 (a, 8.41 eV) LUMO (b, −2.86 eV)

39

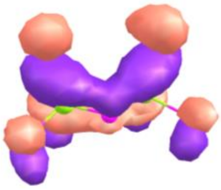
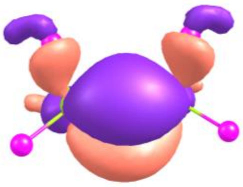
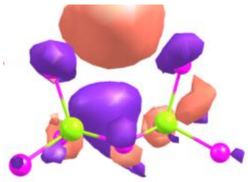
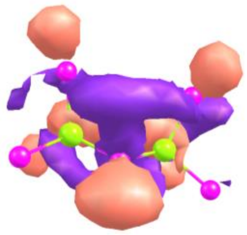

HOMO−4 (−9.14 eV) HOMO (−4.74 eV) LUMO+3 (1.81 eV) LUMO+13 (2.36 eV)

40

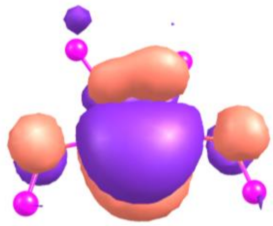
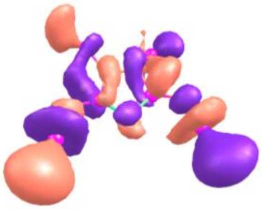
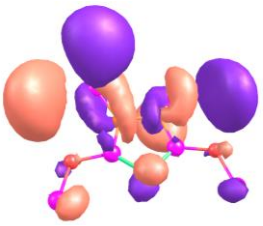
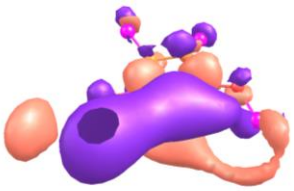

HOMO−1 (−5.71 eV) LUMO+6 (2.33 eV) LUMO+9 (2.71 eV) LUMO+11 (3.21 eV)

41

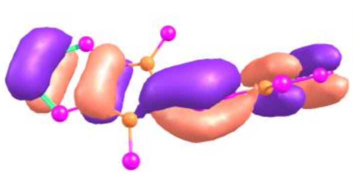
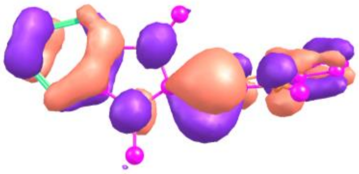
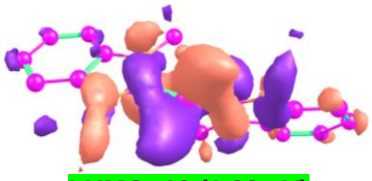

HOMO−5 (−7.04 eV) HOMO (−4.75 eV) LUMO+10 (1.88 eV)

42 (triplet eV)

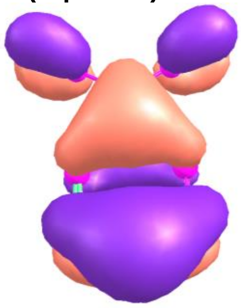
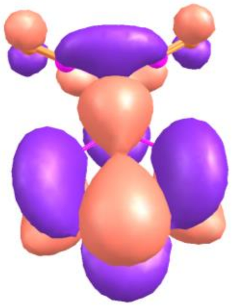

HOMO−2 (a, −8.94 eV) LUMO+2 (b, −4.5 eV)

43 (triplet eV)

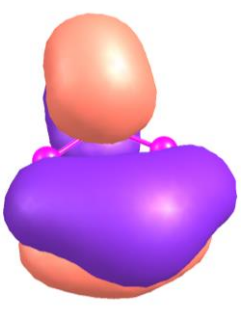

HOMO−2 (a, −8.30 eV)

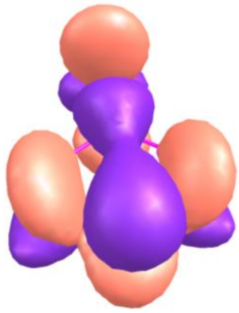

LUMO+2 (b, −2.68 eV)

44

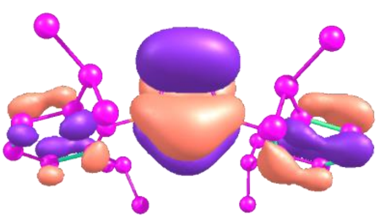

HOMO−1 (−6.47 eV)

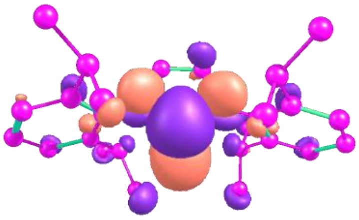

LUMO+5 (0.57 eV)

45

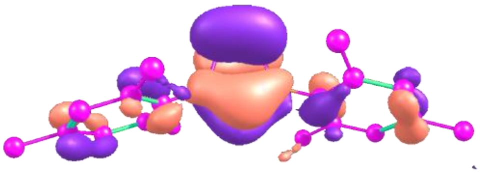

HOMO−1 (−6.29 eV)

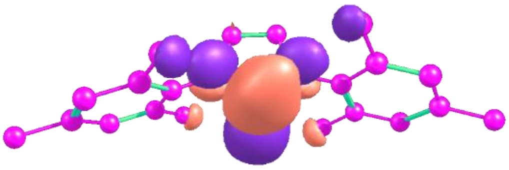

LUMO+5 (0.66 eV)

46

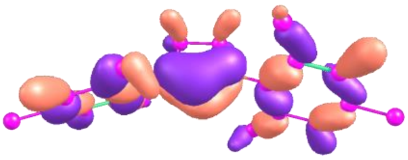

HOMO−6 (−8.66 eV)

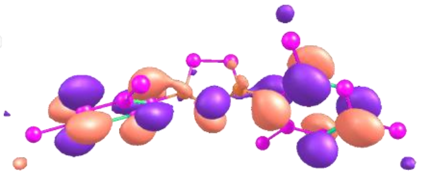

LUMO (−0.31 eV)

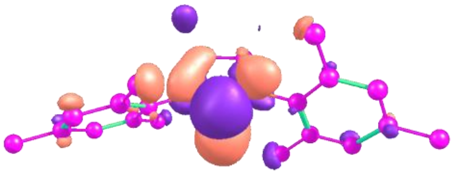

LUMO+5 (0.60 eV)

47

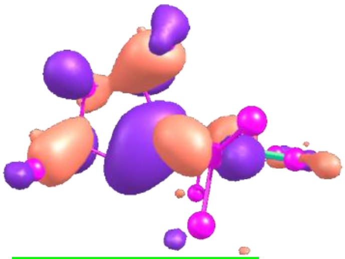

HOMO−3 (−7.78 eV)

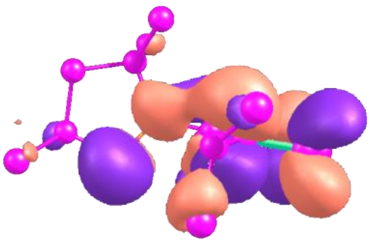

LUMO (−0.39 eV)

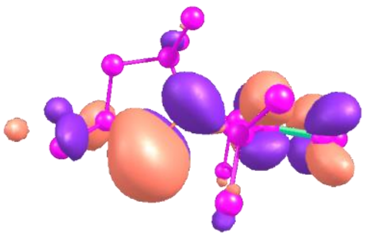

LUMO+2 (−0.05 eV)

48

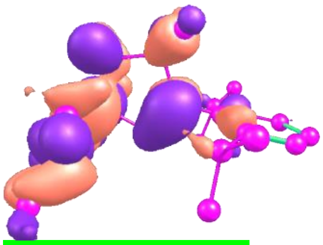

HOMO−3 (−7.46 eV)

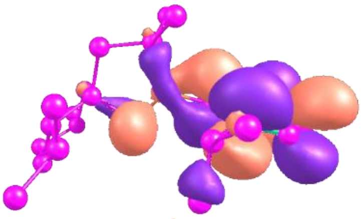

LUMO (−0.38 eV)

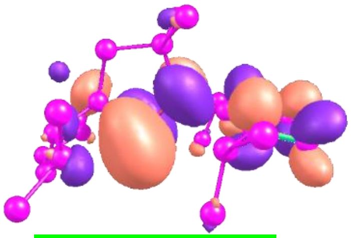

LUMO+2 (−0.04 eV)

49

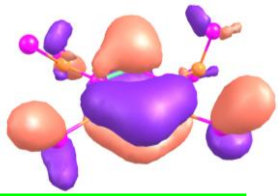

HOMO−6 (−9.08 eV)

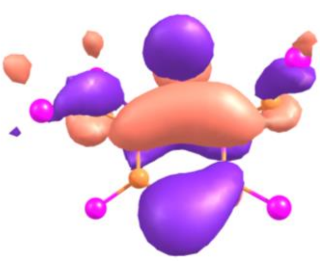

HOMO (−4.89 eV)

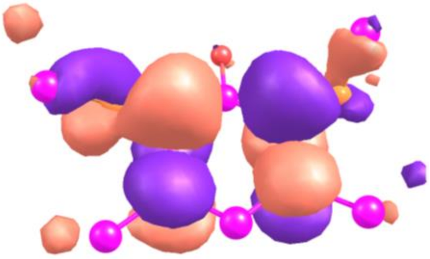

LUMO (−1.43 eV)

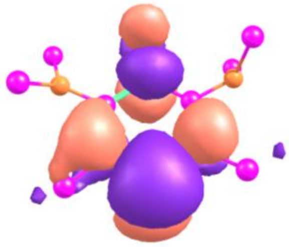

LUMO+1 (−0.01 eV)

50 (only one orbital in threefold symmetry shown)

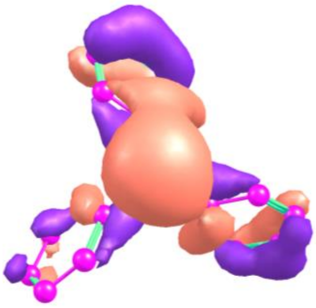

HOMO−6 (−9.14 eV)

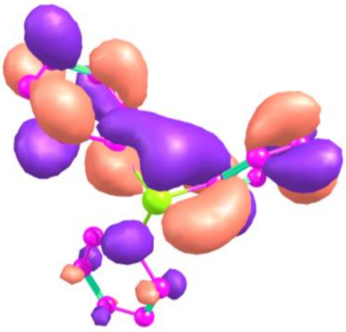

LUMO (−0.94 eV)

51

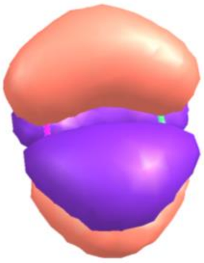

HOMO−4 (−8.01 eV)

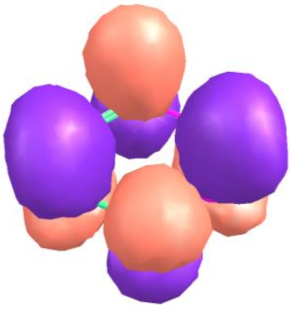

LUMO (0.06 eV)

9.) XYZ Coordinates

Carbenes / C-donors

01

27

C	-2.871059	0.453982	-1.148361
C	-1.988617	0.009384	0.014112
N	-2.803567	-0.405668	0.953801
C	-4.329185	0.457732	-0.604386
C	-4.291973	-0.388047	0.681127
C	-2.325929	-0.929637	2.226734
H	-1.230757	-0.942957	2.191395
H	-2.703197	-1.950883	2.401390
H	-2.655794	-0.293638	3.063110
C	-2.438586	1.848452	-1.623921
H	-1.409240	1.827607	-2.013845
H	-2.469545	2.573795	-0.794937
H	-3.106376	2.212340	-2.423129
C	-5.048827	0.270590	1.838681
C	-4.791894	-1.824042	0.452297
H	-5.057682	0.064520	-1.329119
H	-4.638599	1.486545	-0.361989
C	-2.694530	-0.560868	-2.293626
H	-3.022003	-1.569423	-1.996614
H	-1.638867	-0.627593	-2.596549
H	-3.291463	-0.254341	-3.168977
H	-4.198771	-2.328246	-0.323970
H	-5.843267	-1.812451	0.123362
H	-4.736274	-2.426648	1.372415
H	-4.610172	1.251140	2.081855
H	-5.038348	-0.352352	2.746723
H	-6.101489	0.430066	1.556211

02

15

N	3.003928	-0.550427	0.920875
N	3.006110	0.532483	-0.909848
C	2.145438	-0.005745	0.006409
C	4.341011	-0.361856	0.589052
C	4.342412	0.333865	-0.580772
C	2.554603	-1.245738	2.110150
H	2.822862	-2.315331	2.072407
H	1.463151	-1.148335	2.160150
H	3.001328	-0.801931	3.014481
C	2.559613	1.229625	-2.099072
H	2.841822	2.295769	-2.066013
H	1.466808	1.145894	-2.143225
H	2.995972	0.777434	-3.004338
H	5.162725	-0.733510	1.197211
H	5.165658	0.699543	-1.190487

03

17

N	2.973910	-0.637752	0.877620
N	2.976799	0.619998	-0.866354
C	2.160562	-0.005668	0.006618
C	4.405485	-0.352098	0.692400
C	4.406556	0.322923	-0.684499
C	2.520639	-1.310225	2.067414
H	2.942418	-2.330010	2.137341
H	1.425649	-1.375809	2.032320
H	2.816036	-0.759973	2.981956
C	2.526399	1.295332	-2.055619
H	2.956915	2.311276	-2.127606
H	1.432119	1.370710	-2.017904
H	2.814650	0.741603	-2.970378
H	5.011387	-1.272199	0.734052
H	4.770738	0.322005	1.490888
H	5.019601	1.238227	-0.727614
H	4.764677	-0.354011	-1.483855

04

9

O	-6.388924	1.761225	0.389119
C	-5.506537	2.735463	0.365755
O	-4.314115	2.241195	0.117026
C	-4.325321	0.779358	-0.058747
C	-5.791693	0.439214	0.139797
H	-3.653256	0.347568	0.693959
H	-3.940115	0.558801	-1.063687
H	-5.999088	-0.191283	1.014771
H	-6.281712	0.010809	-0.744634

05

13

O	2.977690	-0.437836	1.004803
N	2.990974	0.560375	-0.901845
C	2.196216	0.099679	0.068508
C	4.399158	-0.377260	0.665090
C	4.430602	0.359829	-0.675240
C	2.516391	1.243186	-2.081051
H	2.860313	2.293745	-2.096355
H	1.419371	1.221721	-2.071782
H	2.884729	0.748726	-2.996732
H	4.781586	-1.407374	0.607464
H	4.924411	0.150144	1.473682
H	4.958019	1.328612	-0.634469
H	4.877681	-0.232187	-1.491793

06

11

C	-6.472440	1.808302	-0.035950
C	-5.413341	2.828284	0.321172
O	-4.275297	2.208875	0.315544
C	-4.329507	0.757607	-0.030312
C	-5.808580	0.413780	0.055539
H	-3.677179	0.239327	0.683658
H	-3.902216	0.662887	-1.040479
H	-6.045937	-0.065336	1.017418
H	-6.122254	-0.269065	-0.745329
H	-6.794920	2.071113	-1.062718
H	-7.363307	1.936717	0.597267

07
19

C	-0.662977	2.549355	0.021831
N	-1.874564	2.069474	-0.334677
C	-2.263890	0.729743	-0.787381
C	-3.017183	2.927355	-0.048726
N	0.454627	1.896418	-0.362013
C	1.708369	2.360299	0.216521
C	0.645160	0.879816	-1.400637
H	1.481190	3.169576	0.919234
H	2.398781	2.731525	-0.564787
H	2.222038	1.543965	0.757837
H	0.755219	-0.142721	-0.993961
H	1.568072	1.110899	-1.958333
H	-0.181152	0.894259	-2.119366
H	-3.575084	3.172079	-0.972058
H	-2.642761	3.854498	0.400424
H	-3.722106	2.435275	0.647700
H	-2.463584	0.668822	-1.873293
H	-3.192668	0.440566	-0.268296
H	-1.500228	-0.009922	-0.524939

08
21

N	2.898246	-0.497735	0.894425
N	2.936905	0.425841	-1.018298
C	2.060669	-0.084551	-0.102703
C	4.248176	-0.258222	0.616411
C	4.273202	0.339091	-0.612520
C	2.422961	-1.103962	2.119798
H	1.327413	-1.127886	2.074743
H	2.734860	-0.518725	3.000523
H	2.804140	-2.132930	2.236124
C	2.512884	0.994824	-2.280088
H	2.750745	2.071021	-2.338879
H	1.426586	0.861404	-2.354878
H	2.999642	0.486673	-3.128386
C	5.356563	-0.625616	1.541587
C	5.418168	0.837306	-1.425370
H	5.461411	0.356213	-2.417352
H	6.368930	0.627854	-0.914527
H	5.363539	1.927169	-1.595369
H	6.324170	-0.316065	1.120969
H	5.403032	-1.714032	1.721293
H	5.251377	-0.138271	2.525917

09
17

N	2.985084	-0.568525	0.803892
N	3.011073	0.551779	-0.930890
N	2.170714	-0.042138	-0.085225
C	4.303147	-0.304564	0.517670
C	4.357295	0.455175	-0.663638
C	2.427784	-1.264608	1.949352
H	3.059771	-2.126567	2.200895
H	1.419015	-1.611601	1.692874
H	2.374864	-0.593307	2.820824
C	2.425249	1.254374	-2.060879
H	3.245457	1.756461	-2.585857
H	1.692197	1.993832	-1.706902
H	1.922494	0.546209	-2.737318
C	5.415954	-0.790180	1.383900
H	6.359512	-0.415381	0.966817
H	5.471299	-1.892922	1.419176
H	5.331271	-0.425178	2.422769

10
14

C	-0.747931	2.596929	-0.264073
C	-2.022715	1.820093	-0.161714
H	-2.751971	2.268639	-0.856762
H	-2.440114	2.025811	0.841490
H	-2.027030	0.720155	-0.317492
N	0.380606	1.931223	-0.377957
C	1.628436	2.691079	-0.491854
C	0.613094	0.475804	-0.436609
H	1.378447	3.753941	-0.400206
H	2.108872	2.498779	-1.467511
H	2.338057	2.396408	0.299982
H	-0.314603	-0.080287	-0.269040
H	1.344647	0.183932	0.333267
H	1.029986	0.195533	-1.418912

11
11

O	3.022720	-0.540002	0.927286
N	3.014476	0.545793	-0.906567
C	2.176223	0.013561	0.016190
C	4.332253	-0.358498	0.571999
C	4.358547	0.329203	-0.587458
C	2.551719	1.239441	-2.093198
H	2.919374	2.278260	-2.106139
H	1.455072	1.239929	-2.072047
H	2.898879	0.726091	-3.003536
H	5.102995	-0.764299	1.220907
H	5.183511	0.678470	-1.202427

12
17

N	8.140020	-0.389528	3.564186
N	9.925413	-0.013890	4.588119
C	8.728493	0.590909	4.281459
N	8.873837	-1.541621	3.406440
C	9.979392	-1.287493	4.049063
C	6.815413	-0.334851	2.988181
H	6.858258	-0.549196	1.908968
H	6.419889	0.673765	3.157692
H	6.155513	-1.074901	3.466550
C	10.984859	0.613133	5.351815
H	11.266681	-0.004577	6.219276
H	10.613870	1.582036	5.707611
H	11.881008	0.775305	4.730714
C	11.136396	-2.213324	4.187126

H	10.912779	-3.151267	3.663213
H	11.346877	-2.440914	5.245753
H	12.052422	-1.775886	3.756372

13
15

N	2.933835	-0.550175	0.929129
N	2.936068	0.532763	-0.917988
C	2.082567	-0.005201	0.006612
C	4.266868	-0.359160	0.593063
C	4.268303	0.330674	-0.585182
C	2.488784	-1.250327	2.118582
H	2.750975	-2.319827	2.067287
H	1.399401	-1.138582	2.171467
H	2.954974	-0.818771	3.017268
C	2.493823	1.235317	-2.107031
H	2.768365	2.301868	-2.059117
H	1.403058	1.135626	-2.154983
H	2.951207	0.796723	-3.006861
Cl	5.585758	-0.916046	1.536778
Cl	5.589446	0.876857	-1.532042

14
17

N	2.935255	-0.492100	0.902368
N	2.973346	0.425334	-1.026943
C	2.106522	-0.082804	-0.102227
C	4.276628	-0.254081	0.623355
C	4.301414	0.344215	-0.620609
C	2.470623	-1.104254	2.138136
H	1.375815	-1.058651	2.135906
H	2.866019	-0.556691	3.006261
H	2.801030	-2.152934	2.200372
C	2.553995	0.993777	-2.299861
H	2.769799	2.073096	-2.334400
H	1.474333	0.828512	-2.391612
H	3.079486	0.499964	-3.130332
C	5.336570	-0.601304	1.496172
C	5.399645	0.822672	-1.376067
N	6.278703	1.234362	-2.014635
N	6.170028	-0.911855	2.243738

15
21

N	2.863673	-0.976941	0.610284
C	2.986352	0.915261	-0.434174
C	2.041574	0.004841	0.020721
C	4.184088	-0.702080	0.534013
N	4.284586	0.468068	-0.126356
C	2.774599	2.219298	-1.130828
H	3.269243	3.063498	-0.616737
H	1.694556	2.419138	-1.145208
H	3.133486	2.219138	-2.178062
C	2.318654	-2.167345	1.239983
H	2.758563	-3.083991	0.815058
H	1.240308	-2.154976	1.042631
H	2.485148	-2.162275	2.330786
C	5.523076	1.153351	-0.439868
C	5.317505	-1.497095	1.084902
H	5.484709	1.540992	-1.468266
H	6.373464	0.463373	-0.360596
H	5.693012	2.003866	0.240240
H	4.938449	-2.295439	1.735786
H	5.996274	-0.872038	1.689454
H	5.922122	-1.975245	0.292958

16
15

N	-2.922270	0.237646	-0.982600
C	-2.087072	-0.208432	-0.022786
C	-2.996302	-0.621896	1.009965
N	-4.272535	0.110237	-0.655179
C	-4.297712	-0.410427	0.597826
C	-2.556872	0.722170	-2.296571
H	-2.959274	0.074454	-3.092820
H	-1.462343	0.695835	-2.331446
H	-2.903239	1.755981	-2.466449
C	-5.359255	0.684426	-1.410773
H	-5.308335	0.382811	-2.468107
H	-6.303916	0.304593	-0.998278
H	-5.369074	1.787610	-1.352793
H	-5.257198	-0.589914	1.083299
H	-2.707634	-1.052375	1.968431

17
21

N	8.903519	4.604547	14.531567
N	6.047824	4.318398	12.097900
C	8.550552	5.106264	11.976723
C	8.343064	4.724574	13.316083
C	7.310704	4.622960	12.438305
C	10.232803	5.145484	14.757207
C	8.179324	4.085580	15.671697
C	5.051989	3.908599	13.062820
C	5.629130	4.444621	10.714127
H	10.648561	5.473629	13.795201
H	10.900140	4.383916	15.198758
H	10.207076	6.012901	15.443495
H	7.317984	3.496860	15.328245
H	7.812155	4.886404	16.343356
H	8.829328	3.420717	16.267076
H	4.187300	4.597535	13.060743
H	5.490558	3.913389	14.069519
H	4.666650	2.893053	12.848274
H	4.811336	5.180454	10.608801
H	5.265620	3.477559	10.319390
H	6.484882	4.778205	10.112784

18
20

C	7.810135	5.782695	0.601960
C	6.313128	5.508932	0.558185
C	8.241351	6.500296	-0.669789
H	8.352770	4.826222	0.683306
H	8.067770	6.384985	1.486741

N	5.948377	4.895618	-0.721798
H	5.739481	6.444760	0.711813
H	6.022852	4.826493	1.374124
C	6.602936	5.048873	-1.890377
N	7.713255	5.812077	-1.849211
H	9.342533	6.525151	-0.736779
H	7.898092	7.554354	-0.659181
C	8.406428	6.089308	-3.091928
C	4.696307	4.164502	-0.720462
H	7.911007	5.526223	-3.891349
H	9.469394	5.786326	-3.036073
H	8.371370	7.168411	-3.336604
H	4.727967	3.307060	-0.023000
H	4.515718	3.796178	-1.737903
H	3.856739	4.817155	-0.413374

19
28

C	-0.385768	-0.246419	0.017124
C	1.115720	0.149065	-0.012841
C	1.184738	1.664669	-0.033814
N	0.543316	2.125725	-1.087347
C	-0.118047	1.176616	-2.046215
C	-1.112017	0.347094	-1.205570
C	0.998971	0.237887	-2.545927
C	1.739612	-0.368629	-1.337507
C	0.476274	3.564776	-1.322219
C	-0.824509	1.863517	-3.207579
C	1.850350	-0.414645	1.201894
H	0.540757	-0.539539	-3.178436
H	1.683034	0.813995	-3.189679
H	1.696658	-1.470268	-1.349895
H	-0.128743	2.435283	-3.839743
H	-1.292100	1.095348	-3.843482
H	-1.621248	2.538321	-2.861494
H	-1.945028	0.999900	-0.901138
H	-1.539007	-0.439489	-1.848803
H	-0.825136	0.130298	0.953890
H	-0.470764	-1.345312	0.044870
H	1.406194	-0.022808	2.130037
H	1.805588	-1.517113	1.227976
H	2.907584	-0.105904	1.189428
H	2.805609	-0.092037	-1.354963
H	-0.563147	3.928109	-1.288666
H	1.055753	4.041880	-0.524079
H	0.908248	3.832512	-2.298987

20
21

N	-2.981495	0.800825	-0.965248
C	-2.032104	0.324762	-0.093732
N	-2.827060	-0.231952	0.878236
C	-4.322250	0.558734	-0.570800
C	-4.220536	-0.120216	0.639045
C	-2.371160	-0.869687	2.020036
C	-5.390090	1.000310	-1.388471
C	-5.112308	1.660781	-2.559188
C	-3.747130	1.891665	-2.938700
C	-2.720126	1.462981	-2.152902
H	-1.662711	1.597379	-2.380836
H	-6.415497	0.800962	-1.068875
H	-5.919433	2.012404	-3.203883
H	-3.515143	2.413102	-3.868912
C	-5.141332	-0.662968	1.566674
C	-3.256714	-1.396173	2.911718
C	-4.671865	-1.296361	2.690533
H	-1.286795	-0.903323	2.124317
H	-2.871456	-1.896262	3.801951
H	-6.210066	-0.563954	1.363587
H	-5.364849	-1.722552	3.417681

21
21

N	3.273996	-0.565455	0.802895
N	3.197252	0.653981	-0.969223
C	2.389472	0.032023	-0.054911
C	4.602022	-0.340315	0.448336
C	4.551852	0.469598	-0.703323
C	2.871636	-1.308184	1.976917
H	3.316245	-0.867273	2.884679
H	3.188465	-2.362956	1.909047
H	1.778367	-1.258150	2.046912
C	2.693048	1.380724	-2.113694
H	3.086546	0.949694	-3.049395
H	2.981422	2.444801	-2.069239
H	1.599472	1.297964	-2.107594
C	5.817932	-0.754602	0.993839
C	6.986923	-0.311517	0.363588
C	6.936977	0.519476	-0.770390
C	5.716454	0.922893	-1.324802
H	5.858783	-1.395895	1.876139
H	7.955921	-0.619849	0.764390
H	7.867930	0.859185	-1.231187
H	5.678856	1.564668	-2.206972

22
25

N	-1.213170	0.150251	3.125395
C	-0.958767	1.085985	2.219169
C	-1.939706	2.223160	2.532187
C	-3.792369	2.275515	4.458153
H	-4.199993	3.258087	4.205928
C	-4.311013	1.549723	5.543686
H	-5.124009	1.971525	6.139783
C	-3.792295	0.292883	5.874512
H	-4.206685	-0.256936	6.723180
C	-2.748374	-0.276006	5.127228
H	-2.342498	-1.255053	5.388579
C	-2.257704	0.462666	4.054775
C	-2.754550	1.724298	3.715613
C	-0.506302	-1.118498	3.203810
H	0.247602	-1.132927	2.408637
H	-1.208868	-1.957025	3.068693
H	-0.015948	-1.227904	4.185015
C	-2.808389	2.506332	1.293701
H	-3.367936	1.610111	0.984079

H	-2.173689	2.824432	0.453080
H	-3.534953	3.306232	1.510059
C	-1.128996	3.480620	2.911051
H	-1.811810	4.307572	3.165546
H	-0.491372	3.790736	2.070189
H	-0.482724	3.291751	3.781851

23
25

C	-2.984172	0.857727	-1.056406
C	-2.033541	0.307046	-0.061117
N	-2.824518	-0.315800	0.802307
C	-4.315909	0.542019	-0.726065
C	-4.309238	-0.277110	0.539879
C	-2.326057	-0.987247	1.988357
H	-1.233804	-0.894505	1.985152
H	-2.606295	-2.054306	1.993186
H	-2.727633	-0.522930	2.904134
C	-5.034029	0.432327	1.692064
C	-4.861611	-1.691846	0.303962
H	-4.277364	-2.210277	-0.470079
H	-5.906971	-1.638297	-0.038970
H	-4.839568	-2.291309	1.227554
H	-4.568796	1.408775	1.893295
H	-5.004913	-0.170904	2.612925
H	-6.090108	0.599993	1.430436
C	-5.380590	0.971564	-1.510424
C	-5.094719	1.738785	-2.649355
C	-3.772262	2.057289	-2.990732
C	-2.706715	1.616129	-2.198537
H	-1.672202	1.857524	-2.455512
H	-6.415313	0.724404	-1.254253
H	-5.913409	2.095028	-3.279890
H	-3.575804	2.654880	-3.884530

24
17

O	3.314508	-0.528964	0.828289
N	3.206844	0.679882	-0.956684
C	2.431181	0.056639	-0.032018
C	4.609406	-0.289264	0.442228
C	4.570333	0.500617	-0.708059
C	2.668036	1.384130	-2.101383
H	3.011186	0.911435	-3.035759
H	2.987948	2.438762	-2.097902
H	1.574041	1.329665	-2.044473
C	5.798846	-0.725565	1.009867
C	6.977514	-0.314430	0.372193
C	6.947579	0.496901	-0.776679
C	5.738782	0.921872	-1.342226
H	5.805520	-1.352731	1.902523
H	7.939774	-0.635810	0.778231
H	7.887279	0.802882	-1.243067
H	5.715522	1.545047	-2.237881

25
13

S	3.125774	-0.669623	1.141429
S	2.990130	0.712139	-1.286734
C	2.125512	-0.019354	-0.043928
C	4.731504	-0.257047	0.484445
C	4.663298	0.437788	-0.734022
C	5.972129	-0.562221	1.060648
C	7.134644	-0.167262	0.401886
C	7.066123	0.528621	-0.817852
C	5.834478	0.838039	-1.391375
H	6.023319	-1.097059	2.011715
H	8.107251	-0.403785	0.840558
H	7.985902	0.831940	-1.323789
H	5.779078	1.376803	-2.340112

26
17

S	3.006911	-0.721578	1.126210
N	3.151959	0.661822	-0.936350
C	2.199058	0.136301	-0.142200
C	4.632096	-0.358074	0.537053
C	4.504458	0.436585	-0.617022
C	2.797245	1.406509	-2.138962
H	3.219982	0.912425	-3.028401
H	3.183769	2.437350	-2.087654
H	1.704374	1.421537	-2.209397
C	5.893797	-0.730514	1.010288
C	7.022416	-0.282236	0.321288
C	6.893465	0.529483	-0.819074
C	5.636471	0.897332	-1.300921
H	5.992531	-1.356674	1.899439
H	8.015826	-0.569059	0.675456
H	7.787376	0.877801	-1.342145
H	5.542564	1.522060	-2.190409

27
13

C	-2.909790	0.962877	-1.145163
C	-2.154933	0.616777	0.131050
N	-2.931946	-0.443552	0.830272
S	-4.646344	0.352159	-0.795542
C	-4.206691	-0.639028	0.540096
C	-2.259856	-1.133376	1.918768
H	-1.398180	-1.707832	1.536339
H	-2.974822	-1.816473	2.392038
H	-1.887682	-0.412268	2.668356
H	-1.134897	0.252568	-0.069993
H	-2.078523	1.487314	0.808148
H	-2.915127	2.039491	-1.360985
H	-2.522137	0.424614	-2.022504

28
9

C	-3.130015	1.077023	-0.887310
C	-2.250237	0.581307	0.242044
S	-2.526040	-1.296338	0.467496
S	-4.516556	-0.198036	-1.199694
C	-3.928307	-1.520511	-0.389151

H	-2.584382	1.170965	-1.837307
H	-1.180155	0.729406	0.040110
H	-3.620624	2.032806	-0.655388
H	-2.503174	1.042178	1.208010

29
11

C	-2.921491	0.961016	-1.022438
C	-2.195030	0.505461	0.025696
N	-2.903613	-0.416405	0.805468
S	-4.493249	0.186586	-0.973449
C	-4.165606	-0.737990	0.447743
C	-2.268238	-1.040954	1.965694
H	-1.441376	-1.695242	1.646794
H	-3.027636	-1.639629	2.481288
H	-1.872672	-0.272282	2.648289
H	-2.609461	1.680054	-1.778229
H	-1.176907	0.781585	0.301384

30
7

C	-3.127615	1.016424	-0.753547
C	-2.316291	0.440563	0.160208
S	-2.828165	-1.181106	0.640179
S	-4.494430	0.006748	-1.230715
C	-4.217024	-1.366844	-0.294711
H	-2.995641	2.008623	-1.189992
H	-1.422564	0.892972	0.593998

31
24

C	3.351323	0.431561	-0.365485
N	2.498443	-0.178521	-2.266582
C	2.327983	-0.392359	-0.919316
C	4.091680	1.129943	-1.393223
C	3.527976	0.712273	-2.571549
C	1.689594	-0.820133	-3.282894
H	1.139938	-0.080173	-3.891446
H	0.968453	-1.469571	-2.770652
H	2.307546	-1.432959	-3.962897
P	3.468890	0.601600	1.348557
C	2.552194	-0.755471	2.126653
C	2.794275	2.182744	1.977861
C	5.202926	0.606906	1.919045
H	3.784513	0.971519	-3.599600
H	4.923782	1.827178	-1.286842
H	3.318163	3.005486	1.468434
H	2.921258	2.280397	3.067559
H	1.728088	2.233841	1.711491
H	2.081189	-0.447869	3.071044
H	3.224591	-1.608326	2.296506
H	1.807564	-1.040691	1.360127
H	5.745349	1.396918	1.378851
H	5.659552	-0.360864	1.665443
H	5.279001	0.792114	3.001498

32
12

N	2.865282	-0.729471	0.698210
C	3.036609	0.420297	-1.200370
C	2.065798	-0.400540	-0.292611
C	4.243687	-0.245897	0.594930
C	4.388457	0.453928	-0.531649
C	2.474281	-1.492691	1.874046
H	3.127584	-2.371923	1.994935
H	1.435412	-1.817687	1.743347
H	2.560081	-0.866078	2.777443
O	2.745333	0.934341	-2.250942
H	5.276603	0.949523	-0.917268
H	4.955342	-0.489993	1.385158

33
15

N	-2.996187	0.561840	-0.941582
C	-2.164523	0.005801	-0.001604
N	-2.991333	-0.541826	0.947690
C	-4.353287	0.418052	-0.649858
C	-4.349949	-0.383111	0.671853
C	-2.513342	-1.229133	2.135541
H	-1.430044	-1.070132	2.201591
H	-2.729632	-2.307773	2.077641
H	-3.010102	-0.822608	3.028744
C	-2.524607	1.245866	-2.133915
H	-1.442380	1.082924	-2.207588
H	-2.736757	2.325288	-2.075678
H	-3.029094	0.840149	-3.023148
O	-5.296491	0.823511	-1.272474
O	-5.290222	-0.777138	1.306177

34
24

N	-2.658791	-0.300515	0.988943
N	-4.183534	0.405894	-0.588904
C	-3.914206	-0.395811	0.476735
C	-2.342420	-1.105942	2.175187
H	-1.479202	-1.756892	1.971747
H	-3.229173	-1.705004	2.406585
H	-2.080992	-0.452219	3.019661
C	-5.552999	0.386705	-1.116949
H	-6.039748	1.359577	-0.951721
H	-6.093336	-0.401392	-0.583753
H	-5.541378	0.188440	-2.198946
C	-3.306535	1.316198	-1.208366
C	-1.843882	1.218612	-0.798610
C	-1.633693	0.561348	0.557420
C	-1.186073	2.601762	-0.827685
C	-1.176652	0.268039	-1.834379
O	-0.617714	0.696982	1.200321
O	-3.692736	2.072626	-2.069903
H	-1.664859	-0.718590	-1.847378
H	-0.115846	0.133099	-1.576615
H	-1.255074	0.713790	-2.836682
H	-1.680904	3.283937	-0.120315
H	-1.268828	3.033585	-1.833327

H	-0.130775	2.515519	-0.537087

35
25

N	2.483944	8.331264	0.893562
O	1.215105	8.899826	2.702299
C	3.651358	7.921620	0.275561
N	4.709024	7.838826	1.078711
C	2.263798	8.472073	2.266869
C	3.381644	7.931295	3.159228
C	4.723769	8.229596	2.491252
H	4.962087	9.308115	2.571110
H	5.527909	7.682741	3.009186
C	3.310615	8.582027	4.540912
H	3.519734	9.662291	4.487673
H	4.043733	8.120503	5.220143
H	2.305500	8.457100	4.966099
C	3.160218	6.408020	3.272122
H	3.268037	5.909691	2.297533
H	2.154487	6.195687	3.665691
H	3.897339	5.973924	3.965517
C	6.003144	7.502654	0.504153
C	1.322296	8.584060	0.036801
H	6.703234	8.352210	0.598991
H	5.857467	7.267329	-0.556623
H	6.447123	6.634356	1.020365
H	1.009908	9.637894	0.101254
H	0.468019	7.966972	0.353693
H	1.616687	8.336762	-0.988801

36
35

C	-2.776712	-7.176314	-9.561086
C	-3.026111	-8.014706	-8.421923
C	-2.514604	-5.849148	-9.415486
C	-3.016884	-7.436217	-7.111966
C	-2.477498	-5.236251	-8.117073
C	-2.739594	-6.035917	-6.958545
H	-2.798427	-7.635014	-10.553039
C	-3.279212	-9.388789	-8.578817
C	-3.281741	-8.277567	-5.975364
C	-2.717477	-5.413894	-5.661696
C	-3.514005	-9.648879	-6.184891
C	-3.509805	-10.194121	-7.469908
H	-3.289270	-9.815143	-9.585179
C	-3.296628	-7.622366	-4.694756
H	-3.708095	-10.306404	-5.342690
H	-3.701618	-11.261909	-7.600097
C	-2.183230	-3.869021	-7.971361
C	-2.403587	-4.045941	-5.568442
C	-2.140602	-3.287223	-6.709740
H	-2.319382	-5.221618	-10.288735
C	-3.030096	-6.266540	-4.546919
H	-2.359199	-3.559288	-4.598635
H	-1.891232	-2.228706	-6.603365
H	-1.977744	-3.273510	-8.864222
N	-3.170307	-5.998762	-3.183277
N	-3.576688	-8.095099	-3.410356
C	-3.510300	-7.110155	-2.464467
C	-3.004100	-4.717168	-2.524901
H	-3.239480	-4.870984	-1.465136
H	-1.969426	-4.349293	-2.615230
H	-3.688124	-3.962710	-2.941453
C	-3.958135	-9.445878	-3.043562
H	-4.161026	-9.438176	-1.965997
H	-4.866906	-9.761032	-3.579354
H	-3.151488	-10.164738	-3.254911

37
21

C	-0.548940	-1.615254	-3.156885
C	-0.548935	-0.280902	-3.573000
C	-0.548940	-1.894709	-1.781876
C	-0.548935	0.765179	-2.637537
C	-0.548940	-0.819311	-0.821171
C	-0.548938	0.500133	-1.257875
C	-0.548943	-3.101119	-1.022964
C	-0.548938	-2.847666	0.379653
C	-0.548941	-1.412570	0.524251
H	-0.548942	-2.429094	-3.885303
H	-0.548932	-0.046104	-4.640303
H	-0.548931	1.800241	-2.988353
H	-0.548938	1.325086	-0.540489
C	-0.548935	-3.672998	1.514330
C	-0.548941	-0.843832	1.792069
C	-0.548937	-1.682222	2.919418
C	-0.548934	-3.078855	2.779563
H	-0.548934	-4.759909	1.404544
H	-0.548944	0.242564	1.915361
H	-0.548938	-1.238630	3.918136
H	-0.548932	-3.709295	3.671824

38
23

C	7.946543	4.725624	12.712786
C	7.970874	4.314431	14.054662
C	6.816418	4.441035	14.881659
C	9.136165	3.714408	14.625846
C	6.832133	3.999701	16.198773
C	9.136518	3.285302	15.947892
C	7.988868	3.422226	16.742606
H	5.918657	4.896275	14.458115
H	10.023526	3.585275	14.002602
H	5.936037	4.106000	16.815486
H	10.035653	2.825967	16.367803
H	7.994219	3.071061	17.777172
C	8.793157	5.138638	11.670686
C	10.026332	5.810880	11.933254
C	8.432205	4.924044	10.308691
C	10.845426	6.221386	10.888125
C	9.260983	5.343366	9.275834
C	10.473567	5.990804	9.555481
H	10.316161	6.006577	12.968172
H	7.490517	4.415512	10.091787
H	11.785286	6.733915	11.111007
H	8.962352	5.161364	8.239756

H	11.122192	6.320698	8.740956

39
27

C	-3.865944	0.192227	-0.403585
P	-5.283160	-0.581115	-0.067939
P	-2.353352	-0.439075	-0.219657
C	-1.845626	-1.801644	-1.368304
H	-2.488045	-2.675748	-1.182177
H	-0.790694	-2.099080	-1.250399
H	-2.023836	-1.456834	-2.398280
C	-1.062106	0.831974	-0.494228
H	-1.229190	1.647335	0.224196
H	-0.047693	0.420783	-0.372712
H	-1.185875	1.240804	-1.508184
C	-1.878952	-1.141789	1.430695
H	-2.493025	-2.032616	1.632282
H	-0.814417	-1.422789	1.483362
H	-2.101814	-0.386494	2.198682
C	-5.694696	-2.125918	-1.011104
H	-4.971142	-2.910113	-0.739982
H	-6.712919	-2.494448	-0.805265
H	-5.586325	-1.913803	-2.085410
C	-5.597526	-1.132674	1.676591
H	-6.609563	-1.547647	1.812691
H	-4.858793	-1.900793	1.948859
H	-5.456376	-0.267205	2.340592
C	-6.724396	0.500675	-0.403709
H	-6.696257	0.803134	-1.461141
H	-7.678768	-0.000945	-0.179015
H	-6.620997	1.405724	0.212272

40
23

N	-4.663887	6.425220	0.919675
N	-6.028775	6.562141	0.717979
C	-4.382919	5.110229	0.584661
C	-3.785633	7.559812	0.739093
H	-2.761233	7.194993	0.883341
H	-3.988130	8.341815	1.486998
H	-3.876907	7.997412	-0.272108
C	-6.763431	7.622050	1.368371
H	-6.637084	7.598090	2.466291
H	-7.822243	7.472538	1.123760
H	-6.453078	8.608557	0.988922
C	-6.485239	5.287949	0.438409
C	-5.492459	4.323757	0.302952
O	-7.818300	5.173137	0.376422
C	-8.302324	3.858190	0.113875
H	-9.386691	3.889117	0.287621
H	-7.813180	3.128714	0.775269
H	-8.087460	3.564198	-0.926009
O	-3.078769	4.804812	0.568054
C	-2.770992	3.467576	0.182542
H	-3.288282	2.746650	0.831753
H	-1.681149	3.365852	0.275321
H	-3.093765	3.276840	-0.853522

41
43

N	-2.345143	-1.608164	-0.251974
N	-2.741997	-1.482714	-2.440014
C	-1.711843	-1.701168	-1.501444
C	-3.703249	-1.399466	-0.405369
C	-3.959318	-1.315575	-1.795469
C	-1.670816	-1.803618	1.005484
H	-1.847277	-0.950683	1.682263
H	-0.594958	-1.886897	0.794958
H	-2.017025	-2.724099	1.508768
C	-2.534216	-1.389801	-3.860999
C	-0.391898	-1.871931	-1.728386
H	-1.484003	-4.283593	-1.959473
H	3.231435	-1.045167	-2.650123
C	1.038441	-4.122053	-4.165532
C	1.991464	-3.073399	-4.140613
C	-1.097727	-4.599492	-2.938620
N	0.026437	-3.769275	-3.284223
N	1.530806	-2.128264	-3.239751
C	2.172355	-0.881546	-2.910384
C	0.300320	-2.520197	-2.691815
H	-0.778109	-5.651900	-2.872760
H	2.117894	-0.160796	-3.745224
H	-1.917405	-4.527050	-3.674647
H	1.642802	-0.462444	-2.042241
H	-3.213617	-0.636496	-4.287524
H	-2.708504	-2.348953	-4.379937
H	-1.497361	-1.077164	-4.048908
C	3.131929	-3.120867	-4.938184
H	3.863972	-2.311193	-4.928631
C	3.309764	-4.250062	-5.760152
C	1.212072	-5.235088	-4.983037
H	4.199781	-4.316956	-6.390247
H	0.474398	-6.039568	-5.011870
C	2.371429	-5.286220	-5.781243
H	2.538026	-6.150589	-6.428464
C	-4.731952	-1.297102	0.524908
H	-4.531895	-1.368157	1.595753
C	-5.250398	-1.122882	-2.276115
C	-6.038240	-1.107762	0.036967
H	-5.455915	-1.064771	-3.346791
H	-6.866155	-1.029428	0.745227
C	-6.291911	-1.021482	-1.334512
H	-7.315188	-0.876295	-1.689061

42
15

C	3.022972	1.805791	-1.535147
C	1.782031	1.680073	-0.910243
C	4.093349	2.174850	-0.737781
H	0.903688	1.388136	-1.490891
C	1.647664	1.927978	0.483398
C	4.037297	2.430260	0.622671
H	4.917602	2.720423	1.201588
C	2.792117	2.305181	1.237632
H	2.688507	2.500217	2.307761
H	3.132403	1.612238	-2.605368

C	0.365855	1.796194	1.130379
C	0.202992	2.074916	2.511360
N	0.081208	2.309355	3.646341
C	-0.788208	1.379752	0.419321
N	-1.731267	1.032315	-0.170706

43
13

C	2.961611	2.211725	-1.647024
C	1.776299	1.894243	-0.985688
C	4.013549	2.682940	-0.875471
H	0.918887	1.518450	-1.551760
C	1.652454	2.044784	0.433460
C	3.975903	2.864215	0.499803
H	4.834730	3.243816	1.060780
C	2.785973	2.545551	1.151930
H	2.706589	2.679741	2.234827
H	3.047785	2.087198	-2.730243
C	0.458374	1.710154	1.097025
H	0.364165	1.822127	2.179598
H	-0.401607	1.325294	0.544815

44
65

C	-1.034015	1.096365	-0.356531
C	0.322081	1.160916	-0.310269
N	0.729542	0.091737	0.487906
C	-0.314653	-0.659309	0.955167
N	-1.391864	-0.010336	0.414409
C	-2.748883	-0.450664	0.568919
C	-3.519067	0.072978	1.624752
C	-3.261262	-1.384330	-0.353897
C	-4.590879	-1.796249	-0.191511
C	-5.374783	-1.292571	0.846546
C	-4.844017	-0.368195	1.745656
C	-2.904284	1.021598	2.643463
H	-5.468115	0.012882	2.555907
H	-5.019885	-2.521965	-0.884497
H	-6.410266	-1.625272	0.956413
C	-2.376410	-1.971208	-1.445291
C	-2.319080	0.213036	3.814494
C	-3.874447	2.101172	3.136818
H	-2.064485	1.535196	2.150525
H	-4.692320	1.675664	3.740619
H	-4.326472	2.655175	2.298720
H	-3.343362	2.823012	3.777694
H	-3.118364	-0.328049	4.347760
H	-1.814961	0.877524	4.535448
H	-1.588520	-0.523238	3.449525
C	-1.660085	-3.230174	-0.924098
H	-1.599018	-1.225248	-1.673533
C	-3.127166	-2.257485	-2.750904
H	-1.070031	-2.996589	-0.025224
H	-0.981649	-3.636607	-1.692066
H	-2.393391	-4.012408	-0.666530
H	-2.416223	-2.559029	-3.536636
H	-3.676656	-1.371816	-3.107673
H	-3.850339	-3.081440	-2.638249
C	2.108480	-0.227030	0.718420
C	2.776166	-1.039124	-0.217786
C	2.749112	0.293361	1.860327
C	4.133327	-1.312080	0.004495
C	4.104363	-0.011221	2.042852
C	4.792498	-0.801512	1.121378
H	5.850719	-1.025315	1.279486
H	4.629436	0.371501	2.920048
C	2.064617	-1.609378	-1.436506
H	4.679714	-1.936784	-0.706012
C	1.973032	1.089577	2.898589
C	2.577644	-0.962856	-2.732539
H	0.997824	-1.364271	-1.343883
C	2.166359	-3.140403	-1.487735
C	2.765713	2.265451	3.481667
C	1.471586	0.143434	4.003416
H	1.085389	1.506153	2.398612
H	3.620832	1.926031	4.088410
H	2.120585	2.866917	4.141962
H	3.154672	2.924943	2.689566
H	2.321792	-0.310033	4.539726
H	0.864288	-0.665410	3.571580
H	0.855443	0.689366	4.736409
H	2.453689	0.131340	-2.707468
H	2.025759	-1.348639	-3.605358
H	3.648092	-1.174799	-2.890314
H	3.208603	-3.477582	-1.610107
H	1.588299	-3.535030	-2.339099
H	1.767898	-3.590600	-0.566084
H	-1.768693	1.721695	-0.856402
H	1.026361	1.854630	-0.761011

45
47

C	-1.130735	0.951347	-0.150305
C	0.218701	1.108851	-0.142657
N	0.728139	0.013549	0.556738
C	-0.243921	-0.842709	0.998639
N	-1.382053	-0.233205	0.544015
C	-2.697393	-0.757503	0.761383
C	-3.336682	-0.508966	1.988644
C	-3.311848	-1.502725	-0.256085
C	-4.606057	-1.990293	-0.027697
C	-5.279676	-1.756022	1.176203
C	-4.626744	-1.016298	2.172922
C	-2.630167	0.261357	3.072919
H	-5.135965	-0.830695	3.123524
H	-5.095787	-2.575035	-0.811771
C	-6.670169	-2.291360	1.411999
C	-2.582274	-1.786969	-1.543469
C	2.126235	-0.193560	0.790680
C	2.840147	-1.055447	-0.059352
C	2.745141	0.473115	1.859384
C	4.208069	-1.228679	0.177263
C	4.117348	0.270563	2.057723
C	4.864969	-0.574132	1.228775
C	6.335721	-0.803519	1.473587
H	4.612099	0.783685	2.887546
C	2.135237	-1.781662	-1.174696

H	4.776520	-1.895647	-0.477915
C	1.942390	1.361087	2.774587
H	-2.373882	-0.862482	-2.106991
H	-3.167287	-2.455849	-2.190640
H	-1.606793	-2.257527	-1.340736
H	-2.283460	1.242508	2.709560
H	-1.732746	-0.283999	3.407243
H	-3.288524	0.423983	3.938224
H	1.079603	0.814123	3.187425
H	1.533693	2.234611	2.240133
H	2.556998	1.728914	3.608507
H	1.601202	-1.081372	-1.837502
H	1.375418	-2.466977	-0.765350
H	2.844756	-2.361955	-1.781720
H	-7.058513	-2.814768	0.525872
H	-7.373778	-1.479822	1.662248
H	-6.685144	-3.001021	2.256444
H	6.508583	-1.784510	1.949671
H	6.760910	-0.034849	2.136120
H	6.906342	-0.797717	0.531155
H	0.854927	1.882958	-0.563517
H	-1.923613	1.560876	-0.575693

46

49

C	-1.255313	1.050222	-0.223794
C	0.266326	1.179645	-0.335624
N	0.725443	0.086183	0.553334
C	-0.244764	-0.769124	0.936746
N	-1.395037	-0.266044	0.442991
C	-2.684817	-0.809455	0.717021
C	-3.211822	-0.724585	2.022450
C	-3.421008	-1.402503	-0.323683
C	-4.700384	-1.901550	-0.040276
C	-5.252352	-1.830073	1.242292
C	-4.489092	-1.240438	2.260436
C	-2.404817	-0.106610	3.134409
H	-4.902675	-1.178255	3.271787
H	-5.274708	-2.369430	-0.845543
C	-6.622049	-2.388229	1.538838
C	-2.843961	-1.517486	-1.711533
C	2.117009	-0.136515	0.772345
C	2.770839	-1.179677	0.084021
C	2.821118	0.700998	1.655041
C	4.140969	-1.360237	0.293509
C	4.195832	0.486482	1.830295
C	4.874076	-0.535178	1.159013
C	6.351343	-0.762784	1.362748
H	4.746844	1.131598	2.521234
C	2.000669	-2.086561	-0.840102
H	4.653298	-2.168978	-0.236819
C	2.113630	1.795050	2.413203
H	-2.944076	-0.573301	-2.275827
H	-3.360998	-2.296691	-2.290308
H	-1.771354	-1.760531	-1.674418
H	-2.055383	0.904089	2.865869
H	-1.501157	-0.703987	3.333820
H	-2.996062	-0.031643	4.058524
H	1.117759	1.464957	2.745171
H	1.967006	2.695542	1.790683
H	2.694077	2.102428	3.295291
H	1.443794	-1.511449	-1.598356
H	1.250950	-2.664726	-0.277211
H	2.672544	-2.783033	-1.362283
H	-7.146596	-2.684943	0.618410
H	-7.249912	-1.651395	2.066321
H	-6.556572	-3.277972	2.188557
H	6.896460	-0.734267	0.404453
H	6.543868	-1.751974	1.811828
H	6.790910	-0.002085	2.024813
H	0.642688	2.157248	-0.001189
H	0.633862	1.009812	-1.363215
H	-1.762998	1.065144	-1.199088
H	-1.705174	1.840390	0.403219

47

52

C	-1.025174	2.189394	-0.219198
C	-0.793998	0.756509	-0.717133
C	-1.503061	0.592972	-2.063013
C	0.697468	0.380173	-0.779774
H	0.883838	-0.230224	-1.676937
H	1.338555	1.270844	-0.860010
C	0.985102	-0.465656	0.495794
C	1.622954	0.366694	1.624839
C	-0.370597	-0.936633	0.997159
N	-1.272732	-0.267438	0.320406
C	-2.680753	-0.488649	0.549126
C	-3.350531	0.246256	1.549663
C	-3.334813	-1.494553	-0.195511
C	-4.709121	-1.676577	0.007945
C	-5.405768	-0.906201	0.938364
C	-4.726108	0.029616	1.715558
C	-2.593212	1.111550	2.547343
H	-5.268504	0.584804	2.483823
H	-5.238952	-2.448782	-0.553645
H	-6.476401	-1.068204	1.089819
C	-2.555303	-2.472529	-1.065078
C	-2.344707	0.292651	3.827621
C	-3.264660	2.457409	2.842438
H	-1.607042	1.328616	2.123926
H	-4.240135	2.338102	3.341447
H	-3.427070	3.036543	1.919723
H	-2.630449	3.059407	3.512520
H	-3.295441	-0.011363	4.296352
H	-1.772999	0.882904	4.562796
H	-1.771536	-0.615535	3.586953
C	-2.245156	-3.735785	-0.240508
H	-1.587216	-2.014836	-1.303153
C	-3.238034	-2.804599	-2.396560
H	-1.658301	-3.469009	0.651761
H	-1.665589	-4.458718	-0.838330
H	-3.173569	-4.230052	0.090450
H	-1.084246	1.319533	-2.776432
H	-2.583788	0.783767	-1.980193
H	-1.357725	-0.408304	-2.487667
H	-0.657834	2.899476	-0.976001
H	-0.490060	2.394268	0.717610

H	-2.095755	2.388947	-0.064080
H	2.608127	0.750707	1.310986
H	1.754816	-0.247683	2.528001
H	0.995779	1.228732	1.900562
C	1.868350	-1.679667	0.178542
H	2.833106	-1.357281	-0.249495
H	1.374988	-2.342615	-0.550399
H	2.062646	-2.269559	1.086935
H	-2.585290	-3.448813	-3.006902
H	-3.454132	-1.893509	-2.976186
H	-4.186815	-3.347065	-2.255590

48
71

C	-1.226165	2.304679	-0.078611
C	-0.892171	0.944786	-0.708917
C	-1.657808	0.838789	-2.030812
C	0.629665	0.731098	-0.860816
H	0.875838	0.382657	-1.866449
H	1.164500	1.681178	-0.721584
C	1.039428	-0.303257	0.236390
C	1.662275	0.515716	1.406105
C	-0.251624	-0.907675	0.754525
N	-1.235145	-0.209565	0.241904
C	-2.611345	-0.522265	0.552998
C	-3.223349	0.067367	1.681479
C	-3.294808	-1.459431	-0.251566
C	-4.648875	-1.699966	0.019422
C	-5.293986	-1.062551	1.077206
C	-4.578483	-0.206272	1.912936
C	-2.409196	0.835954	2.714195
H	-5.078540	0.244709	2.771515
H	-5.198872	-2.417541	-0.593003
H	-6.348479	-1.268345	1.279912
C	-2.559143	-2.335408	-1.255457
C	-1.809103	-0.157542	3.726965
C	-3.191151	1.945426	3.424324
H	-1.566157	1.307704	2.198275
H	-3.965401	1.534637	4.092575
H	-3.686845	2.622364	2.709670
H	-2.510685	2.546008	4.049886
H	-2.608038	-0.676465	4.282221
H	-1.171819	0.371617	4.455633
H	-1.198780	-0.911306	3.206276
C	-2.226888	-3.680788	-0.582645
H	-1.599182	-1.859858	-1.482757
C	-3.295115	-2.529838	-2.585070
H	-1.629587	-3.511889	0.325751
H	-1.650098	-4.325374	-1.266024
H	-3.144892	-4.218468	-0.293912
H	-1.322570	1.643586	-2.703185
H	-2.741568	0.954336	-1.878814
H	-1.482821	-0.114688	-2.542723
H	-1.000269	3.101812	-0.803888
H	-0.630637	2.499161	0.823233
H	-2.293377	2.377865	0.179220
H	2.500176	1.103221	0.987442
C	2.191075	-0.285229	2.591792
H	0.917638	1.251942	1.749971
C	2.010643	-1.442749	-0.232018
H	2.848652	-0.951343	-0.766611
C	1.350216	-2.479814	-1.199931
C	2.601521	-2.182394	0.987953
H	-2.673611	-3.119690	-3.277687
H	-3.519616	-1.565409	-3.066956
H	-4.245663	-3.073211	-2.460438
C	3.213083	-1.283496	2.057242
C	2.775115	0.632496	3.664390
H	3.371971	-2.885565	0.644020
H	1.797870	-2.782695	1.447694
H	3.616702	-1.902326	2.877325
H	4.071413	-0.724610	1.636041
C	1.026276	-1.950912	-2.607150
H	0.402059	-2.777330	-0.720359
C	2.178633	-3.764553	-1.376121
H	0.225934	-1.205198	-2.623708
H	0.691029	-2.777013	-3.254170
H	1.915945	-1.500536	-3.080370
H	3.180704	-3.543724	-1.785051
H	1.674017	-4.435222	-2.089674
H	2.310145	-4.327662	-0.443037
H	1.351919	-0.854078	3.027951
H	3.597242	1.243937	3.253094
H	3.182285	0.052458	4.508740
H	2.013016	1.321637	4.064715

49
33

C	-0.190541	11.838121	4.478548
C	0.769985	11.183922	2.311177
C	0.212294	12.348022	1.625307
O	0.348309	12.541202	0.395103
C	-0.600289	13.162197	2.470027
C	1.349402	10.019306	4.397973
C	-1.670674	13.709445	4.593838
C	0.984189	8.797672	1.746915
H	0.122429	8.708404	2.419263
H	1.794634	8.149982	2.126379
H	0.671934	8.412600	0.759822
C	2.179354	10.477631	0.416666
H	2.494038	11.524651	0.410062
H	1.598330	10.299175	-0.502889
H	3.070629	9.824605	0.418096
C	-0.551920	15.259334	1.274145
H	0.256481	15.595754	1.940774
H	-1.199379	16.127655	1.068227
H	-0.103156	14.897549	0.332654
C	-2.541997	13.832376	1.219120
H	-2.298343	13.312617	0.272916
H	-3.150856	14.721865	0.987790
H	-3.152269	13.157672	1.839549
N	0.600762	11.053427	3.666410
N	-0.777310	12.849997	3.804554
N	1.395800	10.187608	1.611904
N	-1.344992	14.237666	1.954390
H	0.775634	9.085342	4.476926
H	1.522706	10.405389	5.407400
H	2.299830	9.818291	3.887136

H	-1.379329	14.761626	4.481261
H	-1.583626	13.378517	5.633784
H	-2.709163	13.601486	4.248201

50
34

P	1.150597	-1.190037	-0.561941
C	1.836396	-2.782289	-1.202180
C	2.920956	-3.456554	-0.622653
C	1.253350	-3.314393	-2.367172
C	3.411326	-4.635471	-1.195231
C	1.730887	-4.499107	-2.926791
C	2.817523	-5.162250	-2.344088
H	3.383803	-3.062322	0.283919
H	0.408158	-2.798136	-2.832381
H	4.255355	-5.151228	-0.729587
H	1.253903	-4.907792	-3.821690
H	3.201854	-6.083676	-2.789144
C	-0.532864	-1.735702	-0.018363
C	-1.511828	-0.738654	0.125456
C	-0.884657	-3.070550	0.245480
C	-2.809455	-1.064401	0.527447
C	-2.185533	-3.397842	0.638418
C	-3.150720	-2.396299	0.780233
H	-1.251557	0.301692	-0.091356
H	-0.138310	-3.859859	0.135428
H	-3.559096	-0.276573	0.636706
H	-2.448633	-4.440249	0.838791
H	-4.166505	-2.655708	1.090139
C	2.027256	-1.012221	1.057487
C	1.580621	-1.591403	2.256273
C	3.190823	-0.224465	1.078952
C	2.281334	-1.385328	3.447561
C	3.896609	-0.024576	2.268259
C	3.438980	-0.600459	3.457341
H	0.675182	-2.200887	2.261838
H	3.542701	0.241616	0.153926
H	1.915897	-1.840480	4.372301
H	4.800320	0.590349	2.268352
H	3.982188	-0.430178	4.390616

51
11

C	-0.542778	0.025679	-4.082717
C	-0.223872	1.178760	-3.362814
C	-0.221786	1.111112	-1.965118
N	-0.511612	0.001151	-1.281350
C	-0.816901	-1.096720	-1.977750
C	-0.845764	-1.139977	-3.376119
H	-0.555196	0.035508	-5.175842
H	0.025462	2.112916	-3.871692
H	0.030766	1.997938	-1.371442
H	-1.056714	-1.993704	-1.394078
H	-1.106656	-2.065197	-3.895391

Boryl Radicals

01_BH2_rad
30

C	-2.876588	0.394266	-1.227348
C	-1.983956	-0.229869	-0.154725
N	-2.765311	-0.551077	0.910266
C	-4.295764	-0.064341	-0.809346
C	-4.226056	-0.430259	0.690991
C	-2.239422	-1.021907	2.174232
H	-1.354116	-0.435786	2.456664
H	-1.937867	-2.083491	2.129621
H	-2.998610	-0.906290	2.959197
C	-2.738733	1.929893	-1.162077
H	-1.710455	2.233598	-1.410818
H	-2.973276	2.318738	-0.159649
H	-3.422394	2.409116	-1.882170
C	-4.812803	0.672054	1.585630
C	-4.913585	-1.775401	0.966287
H	-4.580616	-0.953959	-1.391216
H	-5.059510	0.702963	-1.003910
C	-2.538616	-0.068594	-2.649829
H	-2.584515	-1.165411	-2.735899
H	-1.527477	0.252775	-2.938620
H	-3.252646	0.364866	-3.369777
H	-4.434203	-2.575650	0.382002
H	-5.974311	-1.723743	0.672900
H	-4.872348	-2.051626	2.031686
H	-4.314593	1.634189	1.398932
H	-4.701348	0.429060	2.654313
H	-5.888376	0.792833	1.383208
B	-0.485984	-0.413242	-0.226333
H	0.113870	-0.030833	-1.203620
H	0.127728	-0.939896	0.673411

02_BH2_rad
18

N	3.010267	-0.441884	0.984036
N	3.026526	0.586287	-0.955842
C	2.168508	0.160022	0.053713
C	4.323317	-0.378712	0.558199
C	4.333298	0.259051	-0.646968
C	2.542396	-1.058472	2.204812
H	1.805081	-1.845891	1.982187
H	2.053550	-0.315974	2.856242
H	3.394745	-1.504337	2.735760
C	2.575844	1.220628	-2.173377
H	1.978832	2.116735	-1.944269
H	1.942587	0.534946	-2.760340
H	3.447795	1.512321	-2.774410
H	5.141958	-0.788892	1.143414
H	5.163381	0.510007	-1.301890
B	0.673260	0.314144	0.125979
H	0.084946	0.874922	-0.768332
H	0.074912	-0.122602	1.081035

03_BH2_rad
20

N	2.907128	-0.164154	1.033872
N	2.955135	0.545521	-1.069697

C	2.137618	0.397526	0.035939
C	4.198825	-0.591264	0.517316
C	4.344377	0.301515	-0.713670
C	2.364386	-0.739366	2.238031
H	1.944177	-1.753603	2.077307
H	1.560056	-0.100622	2.623655
H	3.157171	-0.804289	2.999331
C	2.656505	1.441931	-2.157208
H	2.872206	2.499284	-1.898133
H	1.595305	1.362697	-2.424426
H	3.261282	1.167363	-3.035624
H	4.190394	-1.665185	0.231806
H	4.999773	-0.439300	1.257917
H	4.861027	1.250940	-0.457148
H	4.895241	-0.177824	-1.538353
B	0.671334	0.774388	0.133292
H	0.119312	1.313289	-0.794894
H	0.049669	0.527701	1.138029

04_BH2_rad
12

O	-6.416280	1.829389	-0.084053
C	-5.507501	2.781113	0.213685
O	-4.287267	2.233311	0.385098
C	-4.334814	0.851110	0.013149
C	-5.825854	0.540725	0.113844
H	-3.706240	0.272671	0.702489
H	-3.948549	0.737194	-1.015016
H	-6.111860	0.160558	1.110517
H	-6.196320	-0.147270	-0.656949
B	-5.811904	4.252150	0.342709
H	-4.904847	5.008689	0.574484
H	-6.951297	4.617366	0.208875

05_BH2_rad
16

C	-2.976929	0.720573	-1.101782
C	-1.954512	0.244754	-0.071696
N	-2.805844	-0.581943	0.767998
O	-3.966956	-0.310408	-1.101091
C	-3.908261	-1.002381	0.067101
C	-2.270880	-1.242417	1.933095
H	-1.759848	-0.506767	2.575240
H	-1.543022	-2.031214	1.660559
H	-3.086415	-1.706453	2.501577
H	-3.448923	1.670449	-0.791446
H	-1.496782	1.069991	0.496583
B	-4.942440	-2.041652	0.429621
H	-5.849115	-2.247926	-0.336492
H	-4.846604	-2.647839	1.469877
H	-2.572540	0.832987	-2.117598
H	-1.140019	-0.355090	-0.528332

06_BH2_rad
14

C	-6.629059	1.911282	0.084985
C	-5.750655	2.832097	0.897325
O	-4.490075	2.358568	0.894118
C	-4.374942	1.153423	0.115601
C	-5.802486	0.617615	0.003295
H	-3.672831	0.484757	0.633468
H	-3.946313	1.420620	-0.867191
H	-6.031057	-0.035787	0.859997
H	-5.969649	0.040745	-0.917368
H	-7.623300	1.780995	0.536379
H	-6.791203	2.347396	-0.919663
B	-6.087961	4.107867	1.633063
H	-7.228655	4.505564	1.620395
H	-5.223034	4.711346	2.218460

07_BH2_rad
22

C	-0.676419	2.586235	0.066338
N	-1.887216	2.089764	-0.387760
C	-2.178031	0.665097	-0.373677
C	-3.062709	2.935859	-0.428894
N	0.409615	1.781272	-0.266573
C	1.658585	1.915485	0.454443
C	0.573142	1.308737	-1.633923
H	1.466215	2.110332	1.517000
H	2.291471	2.735156	0.060188
H	2.228446	0.975022	0.368020
H	0.852939	0.240638	-1.670937
H	1.369886	1.882679	-2.146631
H	-0.357569	1.452665	-2.195538
H	-3.775318	2.526608	-1.163494
H	-2.790131	3.954184	-0.732537
H	-3.575944	2.994349	0.550572
H	-2.471528	0.286213	-1.369549
H	-3.011976	0.457031	0.322630
H	-1.298697	0.108174	-0.029569
B	-0.545847	3.882880	0.847782
H	-1.527097	4.472233	1.230040
H	0.545161	4.335862	1.095904

08_BH2_rad
24

N	2.890463	-0.428378	0.996518
N	2.910566	0.657451	-0.908434
C	2.051212	0.183995	0.074852
C	4.217886	-0.329925	0.594195
C	4.229866	0.344087	-0.599688
C	2.399372	-1.083500	2.187168
H	1.667935	-1.863375	1.922260
H	1.892592	-0.363242	2.850366
H	3.233570	-1.546951	2.728456
C	2.444475	1.334261	-2.096323
H	1.801901	2.185359	-1.823359
H	1.848179	0.654433	-2.728072
H	3.300187	1.704420	-2.674413
C	5.356983	-0.881104	1.380505
C	5.382007	0.700593	-1.473579
B	0.551306	0.301137	0.128903
H	-0.040140	0.868658	-0.759412
H	-0.052730	-0.173130	1.062477

H	5.294884	0.242752	-2.473940
H	6.318972	0.339086	-1.029011
H	5.478296	1.790994	-1.615736
H	6.309289	-0.617636	0.901187
H	5.317730	-1.981630	1.462003
H	5.381072	-0.473973	2.405790

09_BH2_rad
20

N	2.988476	-0.302762	0.934760
N	2.998855	0.613315	-0.958044
N	2.155843	0.184459	0.006133
C	4.294073	-0.203643	0.607652
C	4.357601	0.423967	-0.655972
C	2.393671	-0.893503	2.118212
H	2.036690	-1.913823	1.904458
H	1.539650	-0.275998	2.424342
H	3.135323	-0.924123	2.924990
C	2.471263	1.208616	-2.155061
H	1.384704	1.054429	-2.168432
H	2.929447	0.734293	-3.037376
H	2.697275	2.288857	-2.194139
C	5.421703	-0.708691	1.436632
H	5.305170	-0.457804	2.502812
H	6.351963	-0.254426	1.072320
H	5.525591	-1.804504	1.349997
B	5.556765	0.817818	-1.480998
H	5.425208	1.401432	-2.531987
H	6.660738	0.545627	-1.064791

10_BH2_rad
17

C	-0.671158	2.583858	0.063227
C	-1.978151	1.844162	-0.093369
H	-2.048891	0.952975	0.557133
H	-2.145189	1.496407	-1.129793
H	-2.805108	2.511333	0.179323
N	0.462614	1.933560	-0.340231
C	1.764415	2.578385	-0.293811
C	0.512059	0.533275	-0.718010
H	2.218947	2.504688	0.711228
H	1.679201	3.640397	-0.553414
H	2.436638	2.088320	-1.013768
H	1.211234	-0.017957	-0.064025
H	0.871901	0.417703	-1.757119
H	-0.470782	0.061343	-0.633051
B	-0.666895	3.980672	0.658341
H	0.344989	4.605896	0.868573
H	-1.729120	4.478921	0.952727

11_BH2_rad
14

C	-3.137383	0.743746	-0.911420
C	-2.256375	0.454729	0.072849
N	-2.759115	-0.647499	0.745112
O	-4.169881	-0.143303	-0.865341
C	-3.955631	-1.031313	0.165938
C	-2.176799	-1.279414	1.908702
H	-1.202051	-0.819590	2.119620
H	-2.039516	-2.357210	1.731127
H	-2.833670	-1.155079	2.784898
H	-3.159446	1.505429	-1.685061
H	-1.320523	0.931752	0.351343
B	-4.876402	-2.157804	0.526050
H	-5.876604	-2.337282	-0.121361
H	-4.586335	-2.866752	1.460503

12_BH2_rad
20

N	8.076894	-0.379672	3.605008
N	9.896253	-0.021209	4.708861
C	8.632457	0.526714	4.482260
N	8.911173	-1.411651	3.301162
C	10.005682	-1.179931	3.975472
C	6.765717	-0.298736	3.010760
H	6.644762	-1.167368	2.352516
H	6.659741	0.629579	2.426571
H	5.984944	-0.306565	3.788155
C	10.896863	0.571903	5.564776
C	11.212084	-2.051235	3.942017
H	11.005486	-2.908814	3.289798
H	11.471614	-2.427030	4.945437
H	12.088384	-1.510264	3.547982
B	8.039706	1.785244	5.057086
H	6.926642	2.121849	4.730524
H	8.689121	2.443790	5.834045
H	11.809369	-0.035988	5.535937
H	10.530813	0.631071	6.602314
H	11.131689	1.593550	5.227310

13_BH2_rad
18

N	2.972154	-0.441582	1.002768
N	2.979203	0.590329	-0.954685
C	2.131578	0.164352	0.068751
C	4.282463	-0.373485	0.566063
C	4.287656	0.262134	-0.644329
C	2.500960	-1.077886	2.213646
H	1.777127	-1.870176	1.968017
H	1.999461	-0.344122	2.863493
H	3.355726	-1.515264	2.743404
C	2.512800	1.223397	-2.168833
H	1.971436	2.152145	-1.931375
H	1.824849	0.554128	-2.708763
H	3.375204	1.454091	-2.805258
Cl	5.614031	-0.980640	1.450149
Cl	5.627292	0.631099	-1.641509
B	0.639989	0.324716	0.152604
H	0.049802	0.893889	-0.733146
H	0.050085	-0.114017	1.110213

14_BH2_rad
20

N	2.967766	-0.446916	1.018364

N	3.010504	0.589168	-0.932073
C	2.140598	0.158033	0.070498
C	4.285093	-0.386917	0.612729
C	4.311458	0.262684	-0.610087
C	2.479496	-1.063462	2.236921
H	1.757728	-1.857384	1.993430
H	1.970204	-0.315065	2.862997
H	3.324363	-1.494048	2.789425
C	2.577603	1.227798	-2.159285
H	1.974522	2.117324	-1.927189
H	1.960193	0.534240	-2.751061
H	3.458709	1.523770	-2.742230
C	5.372318	-0.922818	1.344929
C	5.425774	0.568085	-1.428937
B	0.645797	0.306843	0.117368
H	0.077565	0.865391	-0.787212
H	0.036936	-0.134447	1.059600
N	6.313629	0.836181	-2.128751
N	6.255493	-1.374728	1.949874

15_BH2_rad
24

N	2.881962	-0.975090	0.661421
C	3.034488	0.907564	-0.594451
C	2.143070	-0.027081	-0.022462
C	4.204765	-0.703625	0.560688
N	4.315730	0.416357	-0.200061
B	2.784872	2.150378	-1.415753
H	1.644783	2.454809	-1.695814
H	3.703674	2.844470	-1.793880
C	2.276578	-2.081475	1.377713
H	1.690554	-2.707442	0.687411
H	1.602120	-1.700573	2.159048
H	3.046375	-2.701217	1.850063
C	5.547807	1.081221	-0.546742
C	5.337284	-1.450127	1.165923
H	5.555733	1.301258	-1.625686
H	6.408578	0.448984	-0.294398
H	5.631175	2.046371	-0.018033
H	4.984351	-2.286619	1.781709
H	5.951024	-0.798762	1.813770
H	6.011780	-1.868557	0.396399
C	0.660603	-0.086203	-0.116162
H	0.303853	0.842812	-0.579278
H	0.171745	-0.189033	0.868728
H	0.306242	-0.924617	-0.745048

16_BH2_rad
18

N	-2.995218	0.328327	-1.036989
C	-2.168248	-0.127089	-0.004863
C	-3.070887	-0.483662	1.051376
N	-4.311885	0.421898	-0.586634
C	-4.341305	-0.165588	0.666229
C	-2.578627	1.153717	-2.142383
H	-3.152329	0.916459	-3.051678
H	-1.521790	0.934014	-2.332299
H	-2.685615	2.230956	-1.913212
B	-0.662325	-0.177458	-0.003894
H	-0.004803	0.208999	-0.944601
H	-0.109335	-0.614023	0.978825
C	-5.392590	0.302327	-1.544158
H	-5.387112	-0.674894	-2.064099
H	-6.339470	0.414585	-0.998662
H	-5.337965	1.104172	-2.294353
H	-5.295467	-0.293583	1.173772
H	-2.764011	-0.908565	2.004114

17_BH2_rad
24

N	8.888302	4.601921	14.491341
N	6.126178	4.037625	12.016165
C	8.544887	5.094287	11.919790
C	8.344075	4.693910	13.267235
C	7.343185	4.486121	12.366885
C	10.172940	5.234824	14.741013
C	8.117604	4.151874	15.630382
C	5.200942	3.533767	13.007939
C	5.602813	4.379566	10.703191
H	10.633145	5.509825	13.783025
H	10.845875	4.543600	15.277106
H	10.065079	6.150459	15.353004
H	7.323650	3.468902	15.298520
H	7.647601	4.989923	16.182642
H	8.768152	3.604150	16.332094
H	4.517362	2.807448	12.540043
H	4.587970	4.334515	13.468049
H	5.752517	3.016149	13.806304
H	4.850997	5.189963	10.759545
H	5.121895	3.500442	10.241528
H	6.427423	4.716690	10.061908
B	9.386062	5.696378	10.832446
H	8.946762	5.760305	9.702590
H	10.494893	6.117482	11.089366

18_BH2_rad
23

C	7.903440	5.285833	0.692169
C	6.441517	4.865638	0.644332
C	8.133432	6.336478	-0.382917
H	8.564477	4.427929	0.488970
H	8.152187	5.675312	1.691982
N	5.911601	4.848846	-0.713947
H	5.830266	5.560143	1.252331
H	6.311415	3.864749	1.101794
C	6.661534	5.055967	-1.858819
N	7.842117	5.747002	-1.676284
H	9.176504	6.683100	-0.379545
H	7.493571	7.223329	-0.185294
C	8.638520	6.159824	-2.815960
C	4.551473	4.356999	-0.794400
H	8.852204	5.308063	-3.476265
H	9.590908	6.570560	-2.451742
H	8.131842	6.932325	-3.423456
H	4.493746	3.251555	-0.779335
H	4.069372	4.713517	-1.711528

H	3.984927	4.739793	0.070876
B	6.208319	4.559526	-3.231903
H	6.823192	4.855396	-4.224783
H	5.237676	3.852724	-3.337445

19_BH2_rad
31

C	-0.315616	-0.230960	0.060590
C	1.188272	0.139709	-0.050116
C	1.286348	1.663350	-0.097434
N	0.587675	2.164518	-1.167924
C	-0.149234	1.190966	-2.015185
C	-1.111579	0.420561	-1.084294
C	0.904577	0.211239	-2.572124
C	1.706145	-0.403509	-1.407936
C	0.490028	3.585372	-1.435917
C	-0.928115	1.845399	-3.150783
C	1.959379	-0.499203	1.100312
H	0.387703	-0.560498	-3.165185
H	1.559225	0.765102	-3.263516
H	1.618385	-1.502257	-1.406055
H	-0.272028	2.370071	-3.860445
H	-1.455054	1.058004	-3.710560
H	-1.683193	2.552811	-2.778192
H	-1.864290	1.127373	-0.701227
H	-1.651821	-0.331049	-1.682258
H	-0.689659	0.100801	1.041041
H	-0.410484	-1.328931	0.031290
H	1.591106	-0.140374	2.072334
H	1.839311	-1.594456	1.066831
H	3.033146	-0.267847	1.044239
H	2.777033	-0.164694	-1.497069
H	0.694779	3.804573	-2.493725
H	-0.512376	3.976340	-1.186240
H	1.224792	4.112993	-0.822907
B	2.056085	2.501341	0.904306
H	2.094195	3.708580	0.852728
H	2.663709	1.966425	1.796360

20_BH2_rad
24

N	-2.974870	0.804349	-0.989185
C	-2.021452	0.302123	-0.078292
N	-2.821198	-0.265606	0.936450
C	-4.292271	0.543984	-0.560927
C	-4.195148	-0.128626	0.652943
C	-2.394315	-0.887108	2.076652
C	-5.377517	0.968958	-1.370649
C	-5.124085	1.628320	-2.546398
C	-3.770523	1.881637	-2.947504
C	-2.730749	1.459786	-2.163278
H	-1.680316	1.610905	-2.409365
H	-6.394787	0.753933	-1.037931
H	-5.947390	1.962805	-3.179995
H	-3.552014	2.405352	-3.878549
C	-5.143388	-0.652161	1.569342
C	-3.300987	-1.398351	2.966390
C	-4.707010	-1.279640	2.708475
H	-1.314179	-0.936569	2.208855
H	-2.936915	-1.891289	3.868315
H	-6.205280	-0.539131	1.342675
H	-5.424810	-1.687395	3.422288
B	-0.539421	0.346304	-0.172749
H	-0.009315	0.883789	-1.117034
H	0.137056	-0.160542	0.691104

21_BH2_rad
24

N	3.265556	-0.512258	0.869103
N	3.197074	0.735737	-0.957390
C	2.383156	0.153379	0.016268
C	4.572045	-0.321308	0.465467
C	4.528177	0.480930	-0.698363
C	2.904058	-1.206786	2.083185
H	3.102060	-0.580478	2.970461
H	3.488893	-2.135235	2.169690
H	1.838469	-1.459146	2.055353
C	2.733621	1.448752	-2.124272
H	3.222986	1.043979	-3.025080
H	2.961460	2.526280	-2.053036
H	1.650014	1.316607	-2.214080
C	5.784348	-0.768721	0.991717
C	6.957978	-0.384329	0.324736
C	6.914936	0.422679	-0.821689
C	5.696437	0.870968	-1.354765
H	5.821156	-1.387557	1.889208
H	7.921717	-0.721725	0.713832
H	7.846485	0.715739	-1.311722
H	5.664722	1.500963	-2.244925
B	0.885592	0.243312	0.134723
H	0.250240	0.886457	-0.665108
H	0.315455	-0.326198	1.033451

22_BH2_rad
28

N	-1.158293	0.126459	3.211440
C	-0.902921	1.118728	2.288747
C	-1.901274	2.258229	2.552342
C	-3.772885	2.266780	4.428037
H	-4.174717	3.247751	4.161201
C	-4.326577	1.540458	5.496761
H	-5.159159	1.960199	6.066049
C	-3.814510	0.283732	5.838992
H	-4.255236	-0.270724	6.671248
C	-2.747102	-0.282320	5.127636
H	-2.352388	-1.262130	5.400995
C	-2.212889	0.457304	4.069136
C	-2.711866	1.721856	3.718627
C	-0.462427	-1.137320	3.315207
H	0.362492	-1.155701	2.596433
H	-1.149247	-1.973364	3.099583
H	-0.058272	-1.271493	4.332285
C	-2.790194	2.516997	1.318893
H	-3.311134	1.599363	1.006394
H	-2.179778	2.874684	0.476487
H	-3.548695	3.282655	1.548544

C	-1.165493	3.558135	2.946326
H	-1.894109	4.345989	3.197909
H	-0.540279	3.910515	2.112805
H	-0.518756	3.397786	3.821714
B	0.157363	1.100617	1.220519
H	0.267860	2.058206	0.491627
H	0.904613	0.162663	1.066595

23_BH2_rad
28

C	-3.043842	1.257770	-0.910322
C	-2.137809	0.814004	0.147624
N	-2.928941	0.253394	1.119358
C	-4.364487	0.909033	-0.571199
C	-4.368152	0.187980	0.760325
C	-2.416050	-0.362330	2.319231
H	-1.599698	0.244824	2.733759
H	-2.017519	-1.376573	2.129955
C	-5.230365	0.918498	1.802769
C	-4.814624	-1.278153	0.597859
H	-4.169823	-1.797155	-0.126168
H	-5.849667	-1.320333	0.224739
H	-4.773855	-1.815179	1.558665
H	-4.867327	1.946297	1.948888
H	-5.221476	0.396000	2.772183
H	-6.274634	0.964818	1.458365
C	-5.429246	1.212176	-1.413203
C	-5.164069	1.879871	-2.616570
C	-3.849161	2.230603	-2.960562
C	-2.780658	1.922199	-2.116900
H	-1.758572	2.195742	-2.385646
H	-6.454095	0.935496	-1.149525
H	-5.985922	2.132692	-3.290661
H	-3.659238	2.753996	-3.901141
B	-0.627291	0.909095	0.158241
H	-0.070723	1.450136	-0.767421
H	0.031750	0.445177	1.058197
H	-3.211900	-0.434471	3.072968

24_BH2_rad
20

O	3.309270	-0.517823	0.938905
N	3.201033	0.583321	-0.984608
C	2.430056	0.017227	0.020526
C	4.583265	-0.284958	0.523796
C	4.543090	0.425956	-0.685950
C	5.773548	-0.662925	1.124849
C	6.956720	-0.276985	0.471728
C	6.926237	0.453301	-0.725338
C	5.714937	0.819481	-1.332053
H	5.779402	-1.227240	2.058144
H	7.917717	-0.554610	0.910731
H	7.864824	0.747285	-1.201011
H	5.692298	1.383991	-2.265237
B	0.941839	-0.041404	0.190735
H	0.489004	-0.595575	1.158752
H	0.227117	0.473762	-0.634427
C	2.700136	1.181947	-2.200896
H	2.919186	2.262568	-2.230153
H	1.615806	1.032094	-2.248974
H	3.171737	0.701612	-3.073545

25_BH2_rad
16

S	3.161254	-0.753507	1.163537
S	3.024358	0.689972	-1.384561
C	2.146736	-0.055971	-0.073380
C	4.705748	-0.291777	0.466996
C	4.639803	0.405990	-0.758381
C	5.948332	-0.564635	1.054762
C	7.113746	-0.139494	0.418030
C	7.047875	0.555091	-0.798973
C	5.815717	0.831304	-1.390641
H	5.996823	-1.096694	2.007210
H	8.082389	-0.350171	0.877407
H	7.965046	0.888223	-1.290104
H	5.761091	1.372107	-2.337936
B	0.644707	-0.073476	-0.011004
H	0.091926	-0.588376	0.930114
H	0.008770	0.435138	-0.902084

26_BH2_rad
20

S	3.131511	-0.809584	1.324424
N	3.180801	0.520798	-0.882539
C	2.264663	0.013020	0.027443
C	4.690981	-0.407608	0.628705
C	4.514696	0.338647	-0.559506
C	5.968509	-0.721465	1.092764
C	7.077659	-0.261145	0.374095
C	6.905208	0.503011	-0.788750
C	5.629137	0.809020	-1.269126
H	6.098219	-1.309220	2.003741
H	8.083029	-0.499118	0.729432
H	7.778417	0.871012	-1.332523
H	5.504191	1.404414	-2.174206
B	0.765871	0.104908	0.027725
H	0.161927	-0.423900	0.929073
H	0.169740	0.698332	-0.838828
C	2.785977	1.154095	-2.126585
H	3.004702	2.235232	-2.112042
H	1.711038	1.006950	-2.271727
H	3.330947	0.693624	-2.965596

27_BH2_rad
16

C	-3.175132	1.123412	-0.814393
C	-2.166497	0.468849	0.120994
N	-2.903488	-0.552614	0.854614
S	-4.261518	-0.266980	-1.327528
C	-3.896407	-1.175674	0.143758
C	-2.279079	-1.163571	2.008542
H	-1.501762	-1.897177	1.719834
H	-3.035834	-1.685829	2.607524
H	-1.808650	-0.382947	2.626306

H	-3.780729	1.879207	-0.290069
H	-1.723334	1.195554	0.820486
B	-4.608565	-2.472318	0.449540
H	-5.471783	-2.850776	-0.304736
H	-4.313740	-3.130429	1.419042
H	-1.342335	0.002030	-0.458789
H	-2.704308	1.581522	-1.694669

28_BH2_rad
12

C	-3.113744	1.058981	-0.911024
C	-2.252192	0.583945	0.248280
S	-2.213000	-1.250901	0.195584
S	-4.616869	0.006704	-0.951266
C	-3.843948	-1.457673	-0.393421
H	-2.585944	0.955963	-1.872402
H	-1.218612	0.953284	0.172301
B	-4.533108	-2.801735	-0.421949
H	-5.654423	-2.887060	-0.862090
H	-3.954540	-3.776288	-0.006067
H	-3.435980	2.104165	-0.786886
H	-2.675555	0.897272	1.215702

29_BH2_rad
14

C	-3.103901	1.018092	-0.880154
C	-2.396553	0.582552	0.194348
N	-2.864339	-0.590658	0.739395
S	-4.412750	-0.080701	-1.237461
C	-3.967250	-1.144198	0.096909
C	-2.233098	-1.251953	1.868045
H	-1.800883	-2.217371	1.558660
H	-2.973618	-1.447612	2.657651
H	-1.437229	-0.609477	2.266072
H	-2.919127	1.913319	-1.469230
H	-1.527922	1.067856	0.638491
B	-4.686832	-2.419234	0.421473
H	-5.627644	-2.752354	-0.258296
H	-4.326199	-3.106073	1.348312

30_BH2_rad
10

C	-3.148349	0.993715	-0.745350
C	-2.325905	0.435890	0.171299
S	-2.705372	-1.187251	0.678021
S	-4.485366	0.024733	-1.297797
C	-4.126555	-1.387323	-0.325818
H	-3.030192	1.999368	-1.153453
H	-1.457445	0.932506	0.606959
B	-4.942097	-2.647876	-0.353344
H	-5.907831	-2.721894	-1.074132
H	-4.619936	-3.577537	0.347004

31_BH2_rad
27

C	2.932796	0.660644	-0.247543
N	2.164481	0.035086	-2.257200
C	1.747722	0.178814	-0.927325
C	4.007676	0.771362	-1.200156
C	3.494135	0.380015	-2.410568
C	1.292624	-0.362199	-3.334940
H	0.448482	0.339818	-3.437298
H	0.865854	-1.363271	-3.154766
H	1.862813	-0.373491	-4.274766
P	2.959844	1.194354	1.389612
C	2.606016	-0.096744	2.629419
C	1.756623	2.526960	1.712985
C	4.613909	1.846238	1.779585
H	3.977912	0.312231	-3.383651
H	5.027632	1.102391	-1.013256
H	2.030455	3.395530	1.096571
H	1.725248	2.808640	2.776422
H	0.773928	2.155385	1.385793
H	1.626746	-0.534383	2.387588
H	2.592998	0.330916	3.644094
H	3.373473	-0.880396	2.555501
H	4.869104	2.642641	1.066395
H	5.355030	1.038661	1.696344
H	4.629226	2.253761	2.801222
B	0.362266	-0.125552	-0.412714
H	-0.518552	-0.527108	-1.139895
H	0.114950	0.020170	0.771360

32_BH2_rad
17

N	2.926487	-0.814165	0.742002
C	3.010179	0.363399	-1.228891
C	2.163263	-0.463840	-0.336107
C	4.252927	-0.184455	0.782269
C	4.410152	0.458795	-0.594836
C	2.451830	-1.544875	1.891441
H	1.679685	-2.262796	1.588793
H	2.012732	-0.864727	2.646064
H	3.288888	-2.087672	2.357735
O	2.695575	0.877671	-2.279096
H	5.015235	-0.947383	1.012976
H	4.285095	0.559946	1.600570
H	4.739208	1.507205	-0.566239
H	5.116735	-0.087573	-1.239077
B	0.713803	-0.827541	-0.598013
H	0.187144	-0.405359	-1.593487
H	0.087410	-1.516704	0.172414

33_BH2_rad
18

N	-2.942744	0.418390	-1.013235
C	-2.124453	-0.131553	-0.042214
N	-2.941133	-0.524672	1.002142
C	-4.286116	0.388215	-0.640368
C	-4.282087	-0.248234	0.747607
C	-2.501495	-1.123850	2.248773
H	-1.854966	-0.433229	2.809533
H	-1.950774	-2.056970	2.062624
H	-3.404664	-1.338184	2.834752

C	-2.492832	0.990248	-2.270354
H	-2.005075	0.229369	-2.897188
H	-1.782175	1.810182	-2.091865
H	-3.381007	1.379525	-2.785131
O	-5.228576	0.787100	-1.282810
O	-5.214874	-0.466303	1.483918
B	-0.616720	-0.278359	-0.109388
H	-0.022817	0.130251	-1.072142
H	-0.032345	-0.801778	0.802334

34_BH2_rad
27

N	-2.763447	0.105382	1.397380
N	-4.342376	0.871638	-0.194106
C	-4.046959	0.014472	0.862546
C	-2.490257	-0.452792	2.726300
H	-1.508290	-0.080858	3.036231
H	-2.474712	-1.552009	2.699835
H	-3.260179	-0.126081	3.436962
C	-5.737830	1.091982	-0.587976
H	-5.744211	1.931942	-1.290102
H	-6.348975	1.332043	0.290997
H	-6.151265	0.199166	-1.078971
C	-3.374099	1.557599	-0.930430
C	-1.940811	1.094221	-0.710410
C	-1.693112	0.717771	0.743215
C	-0.951966	2.175298	-1.147684
C	-1.751784	-0.195810	-1.556702
O	-0.611810	0.868727	1.274933
O	-3.670713	2.398686	-1.754307
H	-2.456021	-0.988489	-1.262552
H	-0.726916	-0.574774	-1.427033
H	-1.913935	0.037908	-2.619536
H	-1.057275	3.076507	-0.526613
H	-1.141236	2.453880	-2.192094
H	0.074473	1.802355	-1.035613
B	-5.077209	-0.966854	1.413662
H	-6.170549	-1.037451	0.922569
H	-4.792899	-1.700852	2.320088

35_BH2_rad
28

N	2.531531	8.551824	0.915383
O	1.104629	8.565293	2.698082
C	3.745738	8.176942	0.302671
N	4.795681	7.965855	1.155557
C	2.222070	8.357726	2.259640
C	3.362113	7.846362	3.142497
C	4.677732	8.350976	2.550011
H	4.736193	9.453630	2.658875
H	5.524837	7.923944	3.105653
C	3.184435	8.381822	4.566064
H	3.303696	9.476477	4.601247
H	3.930905	7.932857	5.239092
H	2.179104	8.139650	4.935743
C	3.315249	6.307417	3.140991
H	3.485026	5.895463	2.134930
H	2.337768	5.956609	3.504212
H	4.093484	5.908048	3.810493
C	6.114265	7.623219	0.648202
C	1.443026	9.055318	0.074831
H	6.606364	8.482369	0.157499
H	6.049766	6.810653	-0.086717
H	6.739976	7.289261	1.486560
H	1.836606	9.792009	-0.635981
H	0.698494	9.513070	0.734050
H	0.963762	8.242323	-0.491752
B	3.858235	8.011364	-1.207604
H	2.892811	8.123352	-1.914387
H	4.919652	7.754862	-1.713125

36_BH2_rad
38

C	-2.012594	-3.985986	-8.727492
C	-2.488145	-5.198408	-8.120546
C	-1.627871	-2.922697	-7.970463
C	-2.548291	-5.289077	-6.692343
C	-1.692281	-2.969653	-6.535870
C	-2.171726	-4.158722	-5.895900
H	-1.961570	-3.939495	-9.818364
C	-2.895259	-6.288520	-8.908882
C	-2.990467	-6.519271	-6.087097
C	-2.256804	-4.193447	-4.459127
C	-3.398604	-7.577827	-6.923162
C	-3.356155	-7.457980	-8.312438
H	-2.853774	-6.199845	-9.997276
C	-3.019243	-6.546131	-4.649412
H	-3.779302	-8.500718	-6.495433
H	-3.691241	-8.292630	-8.932741
C	-1.286377	-1.870484	-5.759401
C	-1.815240	-3.073450	-3.726907
C	-1.336425	-1.932123	-4.369743
H	-1.258747	-2.007709	-8.441324
C	-2.747741	-5.414300	-3.879692
H	-1.820977	-3.090878	-2.641080
H	-0.995326	-1.084610	-3.770497
H	-0.917156	-0.973876	-6.263371
N	-2.987149	-5.754514	-2.557745
N	-3.336754	-7.569034	-3.769360
C	-3.336018	-7.101412	-2.455285
C	-3.023693	-4.855138	-1.423099
H	-3.576393	-5.346821	-0.615746
H	-2.012669	-4.613634	-1.056320
H	-3.540618	-3.925260	-1.697300
C	-3.514388	-8.969001	-4.096323
H	-3.384238	-9.552729	-3.179100
H	-4.520210	-9.170404	-4.500418
H	-2.760182	-9.284678	-4.829475
B	-3.630429	-7.879416	-1.200112
H	-3.973162	-9.034694	-1.264608
H	-3.525363	-7.351402	-0.119935

37_BH2_rad
24

C	-0.476967	-1.645631	-3.093965
C	-0.484401	-0.318277	-3.541291

C	-0.501518	-1.900787	-1.715577
C	-0.514332	0.742982	-2.627808
C	-0.535508	-0.810697	-0.790314
C	-0.540399	0.502317	-1.241219
C	-0.497340	-3.163519	-0.988488
C	-0.534279	-2.808209	0.423998
C	-0.558023	-1.385187	0.563411
H	-0.451274	-2.473240	-3.805947
H	-0.465167	-0.104855	-4.612814
H	-0.518069	1.771918	-2.996972
H	-0.564619	1.339629	-0.539133
C	-0.547311	-3.622423	1.565200
C	-0.594529	-0.797398	1.820249
C	-0.606274	-1.627381	2.957227
C	-0.582874	-3.022249	2.829951
H	-0.529223	-4.709638	1.462902
H	-0.614431	0.289622	1.933807
H	-0.635241	-1.174657	3.951626
H	-0.593115	-3.646723	3.726245
B	-0.454581	-4.572579	-1.579082
H	-0.420922	-4.741852	-2.774364
H	-0.451499	-5.539771	-0.856001

38_BH2_rad
26

B	6.006592	4.508298	12.000657
C	7.448387	4.594738	12.517077
C	7.764044	4.268895	13.909923
C	6.817248	4.471397	14.945204
C	9.015156	3.707392	14.276573
C	7.120756	4.173418	16.270328
C	9.309300	3.394283	15.600820
C	8.369029	3.636502	16.608598
H	5.845055	4.895798	14.693685
H	9.748166	3.489207	13.498779
H	6.378846	4.365453	17.049320
H	10.274749	2.947422	15.851592
H	8.605733	3.396248	17.648399
C	8.537524	5.013487	11.630934
C	9.648359	5.748892	12.120898
C	8.508768	4.730916	10.242079
C	10.674291	6.163033	11.275588
C	9.544997	5.129463	9.402863
C	10.634476	5.847517	9.912004
H	9.680766	6.024123	13.176222
H	7.666654	4.171603	9.832835
H	11.507933	6.741921	11.681485
H	9.505110	4.873974	8.340799
H	11.441472	6.167317	9.248426
H	5.094827	4.258477	12.749374
H	5.749287	4.690572	10.836078

39_BH2_rad
30

C	-1.282370	4.612069	10.427531
P	-2.481302	3.408642	10.417774
P	-0.665126	5.517541	9.121256
B	-0.287211	4.533934	11.671530
H	0.185919	3.464284	12.018000
H	0.020716	5.547475	12.271436
C	0.793386	4.792411	8.265223
H	1.451284	4.393838	9.052252
H	0.457444	3.965988	7.619870
H	1.340384	5.531394	7.658962
C	-1.810178	5.981990	7.747443
H	-2.134089	5.095002	7.185976
H	-2.693228	6.481168	8.173158
H	-1.305910	6.673009	7.054942
C	-0.080822	7.149769	9.705140
H	-0.937181	7.699614	10.123060
H	0.652408	6.987175	10.505722
H	0.370994	7.717616	8.877629
C	-1.844200	1.699885	10.645759
H	-1.216360	1.434218	9.781943
H	-1.216875	1.692894	11.547690
H	-2.668608	0.977129	10.748413
C	-3.516529	3.246960	8.902838
H	-4.025238	4.196208	8.683756
H	-2.881468	2.969361	8.048890
H	-4.273034	2.462076	9.051564
C	-3.671296	3.600893	11.794757
H	-4.317182	2.716537	11.901860
H	-3.076417	3.749978	12.708154
H	-4.284956	4.497513	11.620620

40_BH2_rad
26

N	-4.619094	6.386676	0.789507
N	-6.006743	6.475941	0.563668
C	-4.201434	5.120410	0.522834
C	-3.819547	7.581773	0.626541
H	-2.763030	7.297217	0.688616
H	-4.037959	8.308928	1.422556
H	-4.027296	8.049701	-0.352098
C	-6.722546	7.301413	1.526297
H	-6.564337	6.959930	2.568963
H	-7.788452	7.221963	1.275054
H	-6.427339	8.357050	1.428500
C	-6.436241	5.123235	0.433368
C	-5.285913	4.255484	0.254773
O	-7.676871	4.976424	-0.102482
C	-8.548589	4.070659	0.556604
H	-9.532967	4.170905	0.076885
H	-8.636853	4.313893	1.631768
H	-8.199592	3.030350	0.453708
O	-2.885214	4.970694	0.519718
C	-2.291575	3.696100	0.294915
H	-2.670937	2.956559	1.013853
H	-1.212683	3.845545	0.428715
H	-2.509669	3.340471	-0.722843
B	-5.313373	2.819172	-0.222154
H	-6.374067	2.325466	-0.525044
H	-4.334130	2.127358	-0.327289

41_BH2_rad
46

N	-2.281637	-1.559240	-0.132377
N	-2.253872	-1.486195	-2.355306
C	-1.424459	-1.441392	-1.229405
C	-3.599037	-1.632053	-0.557257
C	-3.579566	-1.605413	-1.971029
C	-1.879549	-1.668579	1.249178
H	-1.825664	-0.686688	1.746633
H	-0.883428	-2.130064	1.301919
H	-2.600831	-2.303173	1.784899
C	-1.844583	-1.217100	-3.716146
C	0.008367	-1.573999	-1.221235
H	-1.308967	-3.965981	-1.245247
H	3.373763	-1.148478	-2.318027
C	0.645313	-3.895598	-3.991351
C	1.716083	-2.983885	-4.149438
C	-1.077086	-4.335298	-2.250279
N	-0.028997	-3.531747	-2.837002
N	1.637646	-2.082437	-3.098558
C	2.496405	-0.933210	-2.950039
C	0.575603	-2.412401	-2.249921
H	-0.745629	-5.384587	-2.172404
H	2.841380	-0.606521	-3.942320
H	-1.996943	-4.300966	-2.858020
H	1.929860	-0.118937	-2.474924
H	-2.519271	-0.469955	-4.163858
H	-1.863222	-2.127683	-4.337069
H	-0.825865	-0.813036	-3.721105
B	0.852707	-0.865748	-0.202894
H	0.352079	-0.101962	0.591062
H	2.049407	-1.037057	-0.150197
C	2.612952	-3.104100	-5.212977
H	3.454185	-2.419980	-5.333908
C	2.398387	-4.153828	-6.129274
C	0.435229	-4.936809	-4.896871
H	3.084712	-4.271899	-6.971189
H	-0.392413	-5.637861	-4.777972
C	1.337718	-5.050127	-5.975161
H	1.202254	-5.857411	-6.698820
C	-4.800701	-1.704806	0.147091
H	-4.822201	-1.701816	1.237928
C	-4.757344	-1.668484	-2.714326
C	-5.990657	-1.766948	-0.604399
H	-4.741630	-1.654096	-3.805335
H	-6.946387	-1.824335	-0.078238
C	-5.970543	-1.749190	-2.001160
H	-6.910590	-1.796201	-2.555538

42_BH2_rad
18

C	2.989407	1.841509	-1.504647
C	1.754515	1.688691	-0.893966
C	4.136398	2.249669	-0.778099
H	0.884590	1.372410	-1.473388
C	1.612825	1.941326	0.496116
C	3.975965	2.498199	0.608499
H	4.843184	2.816077	1.192288
C	2.750823	2.351961	1.238817
H	2.649549	2.553201	2.307373
H	3.085781	1.638641	-2.574240
C	0.336384	1.780656	1.147341
C	0.173044	2.049103	2.530113
N	0.047890	2.274770	3.666427
C	-0.811173	1.341904	0.439416
N	-1.750807	0.975929	-0.144708
B	5.510402	2.425017	-1.467261
H	6.465155	2.782880	-0.822606
H	5.629129	2.208493	-2.648197

43_BH2_rad
16

C	2.965278	2.219516	-1.630198
C	1.785182	1.898256	-0.987993
C	4.097535	2.714552	-0.924087
H	0.929921	1.519528	-1.554381
C	1.656869	2.046986	0.431153
C	3.962631	2.867416	0.484509
H	4.816372	3.247437	1.052410
C	2.791146	2.552579	1.145395
H	2.713186	2.686310	2.227870
H	3.040287	2.089615	-2.713432
C	0.468878	1.707321	1.092472
B	5.409142	3.062773	-1.643749
H	6.353600	3.483410	-1.017605
H	5.507662	2.919908	-2.840211
H	0.374353	1.817771	2.175459
H	-0.389711	1.318622	0.540183

44_BH2_rad
68

C	-0.988587	1.026266	-0.399419
C	0.366657	1.119219	-0.309161
N	0.806483	0.043790	0.449688
C	-0.267025	-0.749073	0.851439
N	-1.373896	-0.109292	0.297085
C	-2.721484	-0.537670	0.500983
C	-3.385636	-0.127864	1.673998
C	-3.318909	-1.381192	-0.457092
C	-4.634847	-1.800839	-0.221356
C	-5.318032	-1.402036	0.928238
C	-4.700322	-0.573640	1.864570
C	-2.694876	0.724971	2.729516
H	-5.243726	-0.276257	2.764265
H	-5.126530	-2.459057	-0.941068
H	-6.339387	-1.751066	1.102016
C	-2.542651	-1.883410	-1.667499
C	-2.442549	-0.089999	4.008968
C	-3.460066	2.022947	3.025063
H	-1.711523	1.014058	2.332191
H	-4.446883	1.824015	3.474640
H	-3.619976	2.612012	2.108180
H	-2.894114	2.646404	3.736039
H	-3.389112	-0.417572	4.470108
H	-1.895052	0.514943	4.749296
H	-1.843766	-0.984873	3.785262
C	-2.047816	-3.321595	-1.429278
H	-1.650222	-1.249132	-1.774182
C	-3.338319	-1.781873	-2.975725

H	-1.468274	-3.388489	-0.497549
H	-1.404456	-3.653681	-2.260858
H	-2.897394	-4.020617	-1.355286
B	-0.244621	-1.996282	1.685574
H	-2.707541	-2.088740	-3.825942
H	-3.692877	-0.754975	-3.160413
H	-4.220247	-2.442568	-2.971583
C	2.168777	-0.246043	0.766966
C	2.869995	-1.155134	-0.049605
C	2.746686	0.359302	1.901932
C	4.193711	-1.453630	0.298657
C	4.072610	0.027491	2.209743
C	4.788255	-0.871538	1.418084
H	5.815625	-1.132578	1.686075
H	4.549178	0.470187	3.086890
C	2.206985	-1.839722	-1.237818
H	4.763344	-2.159243	-0.310657
C	1.941075	1.285684	2.804685
C	3.027019	-1.699517	-2.528173
H	1.241403	-1.343442	-1.412835
C	1.908743	-3.313730	-0.914909
C	2.718640	2.542971	3.220515

45_BH2_rad

50

C	-1.164997	0.934046	-0.131076
C	0.186319	1.092617	-0.121633
N	0.726627	0.020997	0.573777
C	-0.280447	-0.835391	1.019721
N	-1.452236	-0.233871	0.559740
C	-2.767649	-0.753710	0.760208
C	-3.471876	-0.398752	1.923820
C	-3.313413	-1.611947	-0.206188
C	-4.609315	-2.100912	0.004265
C	-5.348736	-1.760148	1.142850
C	-4.761625	-0.911343	2.092626
C	-2.828693	0.483779	2.960613
H	-5.321602	-0.645962	2.994379
H	-5.047499	-2.772686	-0.739389
C	-6.746629	-2.286357	1.352462
C	-2.505909	-2.009224	-1.413955
C	2.122600	-0.171858	0.807361
C	2.862022	-0.962146	-0.087857
C	2.713396	0.432848	1.928465
C	4.230787	-1.123323	0.154465
C	4.085735	0.245392	2.130543
C	4.860027	-0.528648	1.256416
C	6.329516	-0.749129	1.515025
H	4.560365	0.711850	2.998766
C	2.182218	-1.628635	-1.254348
H	4.820609	-1.733808	-0.535614
C	1.875612	1.237699	2.887280
H	-2.205110	-1.131396	-2.009116
H	-3.074565	-2.687560	-2.065682
H	-1.577305	-2.517859	-1.106471
H	-2.493960	1.440676	2.528121
H	-1.933639	-0.005836	3.379287
H	-3.523765	0.700817	3.784055
H	1.058322	0.621592	3.296713
H	1.403827	2.101247	2.390023
H	2.481311	1.613124	3.724199
H	1.636880	-0.900634	-1.876541
H	1.438715	-2.360682	-0.897133
H	2.908704	-2.153223	-1.891006
H	-7.017952	-3.030903	0.589638
H	-7.489076	-1.471240	1.305860
H	-6.850456	-2.759556	2.342622
H	6.770539	0.085003	2.081746
H	6.892489	-0.863416	0.575834
H	6.490345	-1.668252	2.105576
H	0.814822	1.868944	-0.548252
H	-1.950273	1.545010	-0.566688
B	-0.137435	-2.105584	1.810497
H	-1.132014	-2.720073	2.112685
H	0.968045	-2.478083	2.122312

46_BH2_rad

52

C	-1.234189	0.971168	-0.035213
C	0.283044	1.066467	-0.220882
N	0.773152	0.001639	0.650998
C	-0.218674	-0.891693	0.991130
N	-1.398242	-0.380626	0.494665
C	-2.703585	-0.877809	0.775235
C	-3.336496	-0.551835	1.987771
C	-3.357864	-1.642413	-0.209494
C	-4.681696	-2.031477	0.017912
C	-5.359120	-1.679439	1.194744
C	-4.666117	-0.954022	2.170672
C	-2.591075	0.166504	3.082740
H	-5.172837	-0.684323	3.102286
H	-5.200795	-2.621516	-0.743623
C	-6.806604	-2.055149	1.387563
C	-2.619093	-2.052463	-1.457760
C	2.174794	-0.195456	0.804818
C	2.880961	-0.995455	-0.113325
C	2.835993	0.477118	1.847651
C	4.273018	-1.068387	0.004580
C	4.229881	0.377644	1.928971
C	4.966888	-0.374414	1.005372
C	6.472693	-0.412716	1.063083
H	4.752857	0.904091	2.733114
C	2.145465	-1.776264	-1.170957
H	4.831840	-1.680918	-0.709477
C	2.044357	1.262508	2.861425
H	-2.180599	-1.187199	-1.980733
H	-3.280876	-2.581738	-2.158276
H	-1.779288	-2.720052	-1.203497
H	-1.969397	0.988044	2.697755
H	-1.903870	-0.530078	3.593156
H	-3.282162	0.578424	3.832650
H	1.372868	0.596005	3.428339
H	1.404738	2.022180	2.383914
H	2.706796	1.773188	3.575743
H	1.502812	-1.129368	-1.789962
H	1.482210	-2.523566	-0.704514
H	2.844802	-2.300028	-1.838804
H	-6.995873	-3.101791	1.098546

H	-7.464032	-1.427146	0.762317
H	-7.122156	-1.921013	2.433055
H	6.863795	-1.401150	0.776208
H	6.845588	-0.167287	2.069203
H	6.911974	0.321146	0.365042
H	0.692841	2.044825	0.074623
H	0.583227	0.884226	-1.270862
H	-1.794006	1.104747	-0.973678
H	-1.608986	1.724510	0.684676
B	-0.049969	-2.184737	1.758842
H	-1.027687	-2.847822	2.006015
H	1.056097	-2.522896	2.104189

47_BH2_rad
55

C	-0.983456	2.156835	-0.125050
C	-0.820337	0.726591	-0.664410
C	-1.594303	0.626643	-1.981727
C	0.658791	0.326943	-0.837719
H	0.749724	-0.330767	-1.715818
H	1.300839	1.200947	-1.022502
C	1.068932	-0.457501	0.433843
C	1.763510	0.442525	1.474270
C	-0.266715	-0.946720	0.986307
N	-1.273530	-0.288198	0.341290
C	-2.660551	-0.548428	0.595288
C	-3.345074	0.165808	1.604700
C	-3.307523	-1.558004	-0.155524
C	-4.677580	-1.763105	0.050209
C	-5.384457	-1.013041	0.988418
C	-4.717327	-0.071425	1.767879
C	-2.626200	1.067287	2.600518
H	-5.267563	0.476717	2.536120
H	-5.196969	-2.534402	-0.522608
H	-6.451921	-1.193014	1.141353
C	-2.540281	-2.499013	-1.076462
C	-2.610934	0.407768	3.990503
C	-3.208961	2.483914	2.683576
H	-1.581373	1.149446	2.277206
H	-4.239515	2.478708	3.075360
H	-3.224092	2.980666	1.702404
H	-2.605550	3.103829	3.365826
H	-3.631205	0.303891	4.394459
H	-2.028905	1.019782	4.698614
H	-2.158756	-0.592879	3.946770
C	-2.368425	-3.870490	-0.398733
H	-1.531700	-2.086028	-1.211622
C	-3.176186	-2.645854	-2.465073
H	-1.853295	-3.768206	0.566997
H	-1.777587	-4.546588	-1.038057
H	-3.345149	-4.345663	-0.210445
H	-1.187709	1.364786	-2.689821
H	-2.663734	0.843352	-1.840854
H	-1.496895	-0.364335	-2.440457
H	-0.575659	2.871977	-0.855494
H	-0.455531	2.305746	0.825285
H	-2.044919	2.397583	0.024880
H	2.699947	0.857947	1.064362
H	2.004941	-0.139081	2.376634
H	1.124073	1.282776	1.782442
C	2.002115	-1.623692	0.075402
H	2.912525	-1.246155	-0.420362
H	1.503265	-2.328800	-0.607714
H	2.304002	-2.181311	0.973793
B	-0.448105	-1.966801	2.081644
H	-2.537041	-3.266508	-3.113089
H	-3.313316	-1.670103	-2.955340
H	-4.161395	-3.137058	-2.415485
H	0.529150	-2.463069	2.590546
H	-1.545251	-2.294746	2.458231

48_BH2_rad
74

C	-1.180612	2.212127	0.003800
C	-0.902546	0.842094	-0.638817
C	-1.660010	0.808842	-1.972997
C	0.609732	0.581050	-0.819901
H	0.802288	0.183634	-1.818809
H	1.176176	1.519475	-0.744211
C	1.052448	-0.422280	0.286339
C	1.628875	0.440670	1.451603
C	-0.262800	-1.039162	0.755210
N	-1.295328	-0.290542	0.258574
C	-2.665318	-0.568084	0.587559
C	-3.245764	0.021468	1.734248
C	-3.388924	-1.487291	-0.207668
C	-4.732826	-1.724984	0.106737
C	-5.337831	-1.103602	1.197638
C	-4.592756	-0.251847	2.010020
C	-2.434117	0.831514	2.738282
H	-5.059656	0.194187	2.891030
H	-5.307360	-2.428541	-0.500282
H	-6.382952	-1.315232	1.439206
C	-2.722822	-2.311591	-1.300885
C	-2.198423	0.011684	4.019711
C	-3.061095	2.195029	3.062884
H	-1.447345	1.013778	2.298105
H	-4.018402	2.087779	3.598533
H	-3.252245	2.783275	2.152926
H	-2.389665	2.780599	3.711045
H	-3.149861	-0.233596	4.519697
H	-1.577354	0.579120	4.732077
H	-1.685614	-0.933165	3.789404
C	-2.519733	-3.762814	-0.830168
H	-1.723239	-1.897137	-1.463881
C	-3.466305	-2.265181	-2.641373
H	-1.920323	-3.796935	0.090276
H	-1.997841	-4.347685	-1.605090
H	-3.483316	-4.254925	-0.619035
H	-1.299928	1.632666	-2.607975
H	-2.741813	0.943198	-1.822808
H	-1.500540	-0.126904	-2.520716
H	-0.873602	3.004230	-0.696020
H	-0.625654	2.358182	0.938763
H	-2.253212	2.340532	0.206577
H	2.375386	1.132785	1.020535
C	2.304274	-0.315780	2.589564
H	0.817911	1.072913	1.846142

C	2.128362	-1.453821	-0.229903
H	2.824104	-0.852523	-0.849138
C	1.561589	-2.598728	-1.132697
C	2.976470	-2.075316	0.906690
B	-0.472411	-2.220710	1.674654
H	-2.885217	-2.795051	-3.413744
H	-3.625810	-1.231580	-2.984193
H	-4.451606	-2.755408	-2.581143
C	3.460869	-1.108610	1.985079
C	2.761049	0.636838	3.694060
H	3.853182	-2.563202	0.457489
H	2.391457	-2.872451	1.389635
H	3.998791	-1.672264	2.766946
H	4.190828	-0.393561	1.559451
C	0.917328	-2.143142	-2.450359
H	0.777769	-3.101197	-0.539931
C	2.615795	-3.661683	-1.486097
H	0.022408	-1.531159	-2.305886
H	0.606567	-3.019227	-3.041565
H	1.628943	-1.566800	-3.066388
H	3.474325	-3.210910	-2.014769
H	2.171466	-4.412445	-2.159332
H	3.002610	-4.199996	-0.610773
H	1.579903	-1.027575	3.021185
H	3.470911	1.385678	3.301603
H	3.268637	0.091187	4.506429
H	1.908716	1.181827	4.133915
H	0.460808	-2.861658	2.079409
H	-1.581482	-2.548167	2.011768

49_BH2_rad
36

C	0.051073	12.061410	4.385678
C	0.744456	11.106727	2.239529
C	-0.034502	12.043353	1.463135
O	-0.058106	12.048061	0.202830
C	-0.792707	12.972531	2.274629
C	0.851585	9.748491	4.268217
C	-0.799349	14.357256	4.286142
C	1.386718	9.704866	0.289220
H	0.330107	9.753467	0.013963
H	1.738765	8.658199	0.247129
H	1.949018	10.296958	-0.452270
C	2.940359	10.041272	2.155426
H	3.090760	10.656856	3.050675
H	3.666290	10.378133	1.392320
H	3.172437	8.990835	2.406584
C	-1.579550	14.341076	0.351948
H	-0.541900	14.303904	0.011690
H	-1.949857	15.381956	0.325180
H	-2.179397	13.736263	-0.348935
C	-3.006406	14.007683	2.316996
H	-3.776872	13.672237	1.598501
H	-3.225208	15.056566	2.586310
H	-3.099723	13.388446	3.217532
N	0.584844	11.023084	3.598291
N	-0.552011	13.076838	3.618415
N	1.585545	10.197316	1.646083
N	-1.686938	13.854980	1.721325
H	0.859297	8.955594	3.511628
H	0.051570	9.573348	4.998319
H	1.809012	9.747939	4.810111
H	-0.863897	15.138743	3.520599
H	0.040136	14.554862	4.963779
H	-1.722646	14.351174	4.884294
B	0.111979	12.078890	5.880220
H	0.730496	11.235284	6.481327
H	-0.458132	12.933578	6.512430

50_BH2_rad
37

P	1.083859	-1.277688	-0.467121
B	1.069555	0.224300	-1.577625
H	2.069870	0.893215	-1.624047
H	0.132795	0.320753	-2.330574
C	1.785259	-2.842906	-1.161373
C	2.877196	-3.506068	-0.585448
C	1.208925	-3.353545	-2.336959
C	3.384288	-4.668662	-1.176662
C	1.706275	-4.520659	-2.914690
C	2.799054	-5.179937	-2.336710
H	3.332268	-3.117731	0.327490
H	0.365568	-2.830589	-2.797192
H	4.234506	-5.181449	-0.719444
H	1.240828	-4.920563	-3.819298
H	3.196924	-6.088057	-2.796798
C	-0.607598	-1.791357	0.023454
C	-1.603228	-0.807387	0.115557
C	-0.924648	-3.126227	0.323829
C	-2.902937	-1.157181	0.488219
C	-2.225453	-3.471431	0.699290
C	-3.216824	-2.488454	0.777779
H	-1.349692	0.228830	-0.122589
H	-0.157525	-3.899558	0.249119
H	-3.674850	-0.386224	0.550222
H	-2.468944	-4.512107	0.928876
H	-4.234046	-2.762794	1.069485
C	2.030287	-1.017360	1.077950
C	1.617036	-1.586709	2.292374
C	3.188233	-0.222487	1.046545
C	2.348135	-1.356007	3.461087
C	3.918492	0.000518	2.215026
C	3.497752	-0.561511	3.425488
H	0.716469	-2.202338	2.330551
H	3.510351	0.230663	0.107290
H	2.010432	-1.798084	4.402162
H	4.817309	0.621221	2.180847
H	4.065675	-0.373067	4.340171

51_BH2_rad
14

C	-0.587192	-0.000117	-4.127770
C	-0.543602	1.206722	-3.393132
C	-0.455029	1.196655	-2.025293
N	-0.401328	0.000994	-1.289674
C	-0.453278	-1.195245	-2.024476
C	-0.541839	-1.206386	-3.392305

H	-0.659715	-0.000558	-5.215633
H	-0.582051	2.171844	-3.903166
H	-0.420231	2.104903	-1.424791
H	-0.417069	-2.103032	-1.423361
H	-0.578808	-2.171912	-3.901686
B	-0.305258	0.001540	0.136227
H	-0.266330	-1.059218	0.703469
H	-0.267988	1.062716	0.702794

BH_3 Adducts

01_BH3

31

C	-2.910630	0.420447	-1.227015
C	-2.022567	-0.093817	-0.106248
N	-2.775493	-0.487826	0.894715
C	-4.341618	0.000827	-0.797832
C	-4.253594	-0.413166	0.689127
C	-2.214291	-0.969010	2.148694
H	-1.341082	-0.355166	2.405461
H	-1.870591	-2.010240	2.044565
H	-2.965981	-0.906788	2.944891
C	-2.746683	1.954169	-1.275100
H	-1.706748	2.222052	-1.510595
H	-3.012979	2.422410	-0.314775
H	-3.403109	2.372471	-2.054301
C	-4.849551	0.646001	1.623820
C	-4.879768	-1.790247	0.935620
H	-4.682021	-0.851767	-1.403552
H	-5.072812	0.808373	-0.946543
C	-2.527012	-0.171389	-2.590216
H	-2.586276	-1.271045	-2.582658
H	-1.501447	0.112786	-2.862328
H	-3.217051	0.204558	-3.363306
H	-4.381447	-2.555101	0.320631
H	-5.945717	-1.769355	0.659574
H	-4.811167	-2.094984	1.991490
H	-4.374370	1.624332	1.458081
H	-4.722249	0.374375	2.683446
H	-5.928998	0.748098	1.434352
B	-0.441258	-0.120742	-0.077868
H	0.041641	0.330359	-1.106469
H	-0.112881	-1.295855	0.131557
H	-0.081988	0.522779	0.916791

02_BH3

18

N	3.010267	-0.441884	0.984036
N	3.026526	0.586287	-0.955842
C	2.168508	0.160022	0.053713
C	4.323317	-0.378712	0.558199
C	4.333298	0.259051	-0.646968
C	2.542396	-1.058472	2.204812
H	1.805081	-1.845891	1.982187
H	2.053550	-0.315974	2.856242
H	3.394745	-1.504337	2.735760
C	2.575844	1.220628	-2.173377
H	1.978832	2.116735	-1.944269
H	1.942587	0.534946	-2.760340
H	3.447795	1.512321	-2.774410
H	5.141958	-0.788892	1.143414
H	5.163381	0.510007	-1.301890
B	0.673260	0.314144	0.125979
H	0.084946	0.874922	-0.768332
H	0.074912	-0.122602	1.081035

03_BH3

21

N	2.824624	-0.372208	0.893471
N	2.984651	0.700364	-1.001395
C	2.132234	0.328085	-0.027043
C	4.262457	-0.362038	0.638121
C	4.327209	0.150959	-0.807683
C	2.298070	-0.811875	2.165220
H	2.744182	-1.783028	2.435432
H	1.209923	-0.918248	2.083388
H	2.523905	-0.088336	2.969691
C	2.648504	1.344373	-2.249828
H	3.283173	2.232684	-2.408148
H	1.596278	1.650079	-2.218914
H	2.806343	0.653838	-3.098542
H	4.696992	-1.366014	0.764930
H	4.774654	0.317494	1.344775
H	5.097162	0.922522	-0.965654
H	4.515633	-0.660773	-1.535623
B	0.574103	0.656162	0.098524
H	0.448900	1.303392	1.141180
H	-0.013378	-0.419219	0.234393
H	0.154877	1.278206	-0.867167

04_BH3

13

O	-6.436635	1.790861	0.270533
C	-5.541404	2.753036	0.279565
O	-4.329659	2.279290	0.092349
C	-4.332902	0.835320	0.074019
C	-5.827216	0.507629	0.009723
H	-3.845411	0.480340	0.994053
H	-3.758706	0.498056	-0.798120
H	-6.165119	-0.200801	0.777003
H	-6.165559	0.162865	-0.978869
B	-5.857279	4.249214	0.593168
H	-5.010804	4.968233	0.092399
H	-5.666327	4.206015	1.822234
H	-7.023918	4.498262	0.345773

05_BH3

17

C	-2.967164	0.706798	-1.136889
C	-1.965948	0.244752	-0.073163
N	-2.796935	-0.681414	0.693780
O	-4.059437	-0.227107	-1.021721
C	-3.958516	-0.944176	0.092618
C	-2.298470	-1.308081	1.900153
H	-2.076967	-0.543828	2.662994
H	-1.372667	-1.867733	1.683173

H	-3.063945	-1.997499	2.273054
H	-3.363327	1.715381	-0.936825
H	-1.599425	1.068033	0.560249
B	-5.146938	-1.875498	0.560551
H	-5.631587	-2.402695	-0.432060
H	-5.954167	-1.062340	1.025721
H	-4.783357	-2.669111	1.417616
H	-2.577987	0.665963	-2.163218
H	-1.091411	-0.283668	-0.493604

06_BH3
15

C	-6.649826	1.918382	0.164113
C	-5.754948	2.843132	0.958134
O	-4.541949	2.359697	0.978860
C	-4.382506	1.180189	0.143053
C	-5.803670	0.651575	-0.046230
H	-3.697427	0.502624	0.668740
H	-3.910386	1.511184	-0.796557
H	-6.036690	-0.100094	0.722850
H	-5.950997	0.182829	-1.029113
H	-7.597230	1.751656	0.695669
H	-6.909140	2.423620	-0.781491
B	-6.096609	4.239215	1.505077
H	-7.283944	4.397647	1.733177
H	-5.868541	4.683863	0.339087
H	-5.278067	4.716569	2.264504

07_BH3
23

C	-0.651642	2.583197	0.080094
N	-1.851502	2.111738	-0.338325
C	-2.141240	0.690848	-0.499988
C	-3.049696	2.945087	-0.313234
N	0.438335	1.861736	-0.283786
C	1.731862	2.126348	0.335704
C	0.532626	1.129288	-1.542169
H	1.590759	2.471805	1.364780
H	2.288980	2.903548	-0.215379
H	2.321166	1.195704	0.334277
H	0.598193	0.035929	-1.406076
H	1.442062	1.460101	-2.070207
H	-0.324958	1.361213	-2.184530
H	-3.707924	2.635541	-1.141285
H	-2.781718	3.998375	-0.433031
H	-3.603211	2.832402	0.635467
H	-2.305508	0.395785	-1.550626
H	-3.059308	0.457513	0.063887
H	-1.330012	0.085096	-0.080483
B	-0.484621	3.878405	1.007704
H	-1.528450	4.487885	1.164095
H	0.418901	4.584970	0.564080
H	-0.124753	3.405840	2.096982

08_BH3
25

N	2.901498	-0.485273	0.922192
N	2.930519	0.469070	-1.007292
C	2.087986	-0.029401	-0.065836
C	4.244219	-0.263673	0.616080
C	4.264536	0.340819	-0.609427
C	2.416447	-1.101458	2.144117
H	1.395027	-1.459263	1.967216
H	2.402307	-0.376780	2.973714
H	3.060393	-1.948821	2.418242
C	2.520577	1.039590	-2.277853
H	2.793659	2.105708	-2.333062
H	1.432651	0.936618	-2.356256
H	3.006456	0.505547	-3.108152
C	5.356617	-0.654146	1.524616
C	5.405648	0.807188	-1.444357
B	0.489530	-0.077761	-0.040775
H	0.124292	0.522951	0.973405
H	0.151732	-1.262019	0.056108
H	0.013123	0.438555	-1.045068
H	5.439032	0.293575	-2.419754
H	6.356089	0.606883	-0.931273
H	5.353943	1.890639	-1.647411
H	6.318239	-0.321815	1.110494
H	5.411670	-1.747409	1.664900
H	5.246084	-0.198220	2.522524

09_BH3
21

N	2.929710	-0.407364	0.921948
N	2.980937	0.514548	-0.933270
N	2.140377	0.087807	-0.007384
C	4.256133	-0.304234	0.601790
C	4.309843	0.318165	-0.647859
C	2.352443	-0.985730	2.121661
H	2.534961	-2.070720	2.147683
H	1.274224	-0.788118	2.108445
H	2.806276	-0.523130	3.009268
C	2.469675	1.142704	-2.138386
H	1.389539	0.962697	-2.194221
H	2.994927	0.710404	-2.999891
H	2.686684	2.220718	-2.112087
C	5.361538	-0.791186	1.467193
H	6.308407	-0.523277	0.980227
H	5.333207	-1.886667	1.597141
H	5.338147	-0.329027	2.468166
B	5.516981	0.753946	-1.596476
H	5.479856	1.985139	-1.710415
H	5.334562	0.242006	-2.708191
H	6.576639	0.376073	-1.100833

10_BH3
18

C	-0.584452	2.582535	0.124039
C	-1.916656	1.911174	-0.088334
H	-2.030775	1.054535	0.599120
H	-2.047263	1.530010	-1.115075
H	-2.716332	2.625576	0.137563
N	0.492507	1.923290	-0.272536
C	1.831587	2.507806	-0.203721

C	0.470057	0.599271	-0.897797
H	1.769771	3.482455	0.288612
H	2.233766	2.627159	-1.223871
H	2.502322	1.838975	0.358377
H	1.357085	0.035123	-0.575255
H	0.498802	0.685155	-1.998000
H	-0.420602	0.033042	-0.607483
B	-0.627177	4.035415	0.757292
H	0.434919	4.516454	1.124290
H	-1.479214	4.010372	1.645393
H	-1.128966	4.690131	-0.172678

11_BH3
15

C	-3.072080	0.654979	-1.008050
C	-2.173518	0.317387	-0.061142
N	-2.788885	-0.674538	0.703732
O	-4.192722	-0.106186	-0.823179
C	-4.026422	-0.921149	0.230301
C	-2.224056	-1.298359	1.890479
H	-2.304910	-0.617493	2.751426
H	-1.169343	-1.552029	1.711327
H	-2.800786	-2.207836	2.097113
H	-3.067454	1.355785	-1.837114
H	-1.169009	0.678553	0.140127
B	-5.143754	-1.892161	0.785520
H	-5.901823	-2.197287	-0.121422
H	-5.729544	-1.222409	1.644772
H	-4.605354	-2.855512	1.326648

12_BH3
21

N	8.065880	-0.413632	3.638218
N	9.924668	0.009562	4.574258
C	8.700127	0.560148	4.314476
N	8.830295	-1.529553	3.442917
C	9.968741	-1.253736	4.020089
C	6.709543	-0.383021	3.127772
H	6.211267	-1.325510	3.393621
H	6.713908	-0.266851	2.033124
H	6.194792	0.468706	3.584670
C	10.982227	0.693184	5.297513
C	11.155084	-2.150726	4.062824
H	10.926105	-3.068678	3.507093
H	11.424598	-2.422278	5.096821
H	12.032970	-1.667081	3.604612
B	8.237680	2.033163	4.705266
H	9.025476	2.794412	4.138007
H	8.381928	2.158602	5.924783
H	7.084659	2.236494	4.348596
H	11.601986	1.291380	4.612134
H	11.611803	-0.040760	5.818047
H	10.516276	1.370217	6.024731

13_BH3
19

N	2.989577	-0.412211	0.975650
N	3.009859	0.465979	-1.007952
C	2.178229	-0.012343	-0.042631
C	4.320807	-0.174234	0.656798
C	4.332799	0.377430	-0.591787
C	2.483893	-1.006361	2.204739
H	1.777542	-1.806405	1.947505
H	1.943753	-0.251315	2.793893
H	3.325409	-1.408657	2.780645
C	2.598153	0.979789	-2.305951
H	2.821325	2.055041	-2.381086
H	1.519300	0.814876	-2.396257
H	3.136258	0.446636	-3.102213
Cl	5.632755	-0.536164	1.686554
Cl	5.649708	0.892375	-1.547512
B	0.588152	-0.103683	-0.008459
H	0.224782	0.524044	0.989450
H	0.297307	-1.292210	0.154975
H	0.093613	0.356147	-1.028426

14_BH3
21

N	2.938679	-0.301149	0.993766
N	2.999750	0.640409	-0.957914
C	2.154076	0.292005	0.052373
C	4.260867	-0.345525	0.574085
C	4.298303	0.256077	-0.668929
C	2.482094	-0.806536	2.284408
H	1.416089	-0.569893	2.370851
H	3.047886	-0.319837	3.091115
H	2.632558	-1.894659	2.340212
C	2.559039	1.297826	-2.181924
H	1.877677	2.115423	-1.915273
H	2.011400	0.583162	-2.812738
H	3.434010	1.685585	-2.718101
C	5.303580	-0.930818	1.332343
C	5.393736	0.473344	-1.540247
B	0.584300	0.528250	0.028204
H	0.402955	1.739398	-0.111973
H	0.051489	0.087268	1.034191
H	0.180792	-0.040795	-0.988352
N	6.140832	-1.422757	1.969156
N	6.265568	0.668943	-2.281573

15_BH3
25

N	2.883304	-1.022153	0.653704
C	3.022900	0.956735	-0.419977
C	2.142367	-0.004251	0.039902
C	4.195869	-0.719910	0.581207
N	4.284392	0.460735	-0.060076
B	2.826984	2.364644	-1.163127
H	3.371406	3.237290	-0.466883
H	1.627255	2.602716	-1.297878
H	3.399767	2.327161	-2.260460
C	2.278232	-2.179404	1.285059
H	1.707673	-2.763596	0.546914
H	1.589058	-1.856433	2.079329
H	3.047346	-2.821557	1.728904

C	5.521965	1.149480	-0.384277
C	5.347993	-1.504745	1.103483
H	5.834250	0.908793	-1.412556
H	6.315215	0.866352	0.319053
H	5.326486	2.228402	-0.328228
H	5.016615	-2.421674	1.605686
H	5.931889	-0.918344	1.831891
H	6.033064	-1.796030	0.290001
C	0.657539	-0.060194	-0.051547
H	0.311460	0.871283	-0.517743
H	0.175301	-0.156185	0.937142
H	0.306400	-0.902888	-0.672945

16_BH3
19

N	-2.982021	0.393353	-0.958006
C	-2.159704	0.012635	0.052640
C	-3.029452	-0.444474	1.079355
N	-4.300828	0.224241	-0.614913
C	-4.327705	-0.301389	0.633892
C	-2.592307	0.967260	-2.228360
H	-3.125239	0.481166	-3.058056
H	-1.514510	0.778503	-2.320977
H	-2.775779	2.054046	-2.253115
B	-0.568223	0.146802	0.020553
H	-0.311759	1.346486	0.192050
H	-0.142466	-0.204748	-1.086829
H	-0.094644	-0.528672	0.927058
C	-5.394109	0.471620	-1.524076
H	-5.349008	-0.190171	-2.403702
H	-6.327353	0.270908	-0.982389
H	-5.403427	1.518395	-1.866679
H	-5.281289	-0.539831	1.101573
H	-2.700418	-0.835183	2.038106

17_BH3
25

N	8.920229	4.580036	14.507712
N	6.036777	4.343830	12.095068
C	8.511808	5.158536	11.995261
C	8.348081	4.737678	13.311085
C	7.292082	4.649807	12.429240
C	10.266695	5.098984	14.717132
C	8.186888	4.092901	15.659365
C	5.077789	3.832818	13.052885
C	5.552099	4.694469	10.765760
H	10.682471	5.423332	13.753733
H	10.917074	4.320746	15.151270
H	10.258259	5.964251	15.404252
H	7.319478	3.504371	15.331249
H	7.829416	4.915800	16.307273
H	8.833085	3.438455	16.267139
H	4.364715	4.612587	13.380557
H	5.599848	3.443357	13.937289
H	4.497221	3.009358	12.603952
H	4.783796	5.486480	10.821850
H	5.102629	3.814032	10.275554
H	6.390609	5.061576	10.159430
B	9.423567	5.723393	10.824123
H	9.910501	4.715640	10.292773
H	10.323229	6.420395	11.292858
H	8.720646	6.321642	10.008272

18_BH3
24

C	7.869620	5.303899	0.685328
C	6.385820	4.991914	0.641077
C	8.171217	6.347315	-0.373722
H	8.456269	4.394072	0.476128
H	8.154990	5.667691	1.684122
N	5.929998	4.763209	-0.730016
H	5.802133	5.815113	1.095127
H	6.160088	4.085931	1.229400
C	6.624806	5.100908	-1.839871
N	7.719563	5.871870	-1.678918
H	9.251015	6.548183	-0.430483
H	7.675469	7.305594	-0.122979
C	8.495134	6.317365	-2.831929
C	4.604225	4.166840	-0.800925
H	7.836634	6.464788	-3.694202
H	9.268269	5.581698	-3.110167
H	8.984531	7.269738	-2.578859
H	4.624346	3.104110	-0.499865
H	4.217098	4.239567	-1.819735
H	3.933755	4.707406	-0.111411
B	6.216735	4.604456	-3.325692
H	7.243054	4.168097	-3.846230
H	5.840695	5.598461	-3.956773
H	5.354234	3.742687	-3.313266

19_BH3
32

C	-0.321120	-0.103965	0.106919
C	1.193315	0.207729	-0.054080
C	1.305981	1.720671	-0.186204
N	0.616414	2.174831	-1.223032
C	-0.151189	1.187948	-2.048546
C	-1.113839	0.467124	-1.080904
C	0.888185	0.181643	-2.577837
C	1.673233	-0.411572	-1.394939
C	0.548727	3.594121	-1.555851
C	-0.923963	1.825883	-3.196265
C	1.975039	-0.387442	1.112676
H	0.360526	-0.596363	-3.151844
H	1.552870	0.707728	-3.281238
H	1.535494	-1.502763	-1.334256
H	-0.263793	2.321773	-3.922078
H	-1.463542	1.030241	-3.731854
H	-1.668883	2.551830	-2.840919
H	-1.879017	1.185182	-0.747585
H	-1.637890	-0.324336	-1.639429
H	-0.666700	0.324525	1.059526
H	-0.441268	-1.196202	0.181041
H	1.636187	0.029762	2.070077
H	1.831358	-1.480083	1.130929
H	3.048883	-0.174773	1.024627

H	2.753175	-0.229308	-1.501809
H	0.838046	3.757805	-2.603248
H	-0.471890	3.976466	-1.401318
H	1.234888	4.128035	-0.893441
B	2.107714	2.583859	0.873033
H	1.516675	2.333179	1.933835
H	3.234941	2.099367	0.977307
H	2.142536	3.787224	0.665692

20_BH3
25

N	-2.991707	0.807835	-0.979902
C	-2.068997	0.318666	-0.100090
N	-2.826207	-0.240013	0.890170
C	-4.317771	0.561797	-0.571105
C	-4.212036	-0.119671	0.642102
C	-2.363556	-0.875049	2.032842
C	-5.386627	1.000817	-1.385280
C	-5.109639	1.661782	-2.557538
C	-3.749214	1.895402	-2.942650
C	-2.714689	1.467406	-2.165469
H	-1.655811	1.596342	-2.386093
H	-6.410114	0.798874	-1.063655
H	-5.919222	2.010921	-3.200061
H	-3.522054	2.419149	-3.872432
C	-5.135551	-0.662545	1.564489
C	-3.258866	-1.396235	2.917983
C	-4.670730	-1.297248	2.690021
H	-1.279403	-0.904991	2.131557
H	-2.878301	-1.894074	3.811341
H	-6.202177	-0.563365	1.353874
H	-5.367440	-1.725343	3.412129
B	-0.489178	0.378892	-0.276596
H	-0.220262	-0.266017	-1.295760
H	0.071214	-0.097670	0.703588
H	-0.173294	1.557450	-0.460952

21_BH3
25

N	3.221569	-0.212862	1.202430
N	3.165252	0.269879	-0.924569
C	2.374876	0.043116	0.163900
C	4.546327	-0.148681	0.787475
C	4.511166	0.163821	-0.583984
C	5.754287	-0.319408	1.463785
C	6.929166	-0.169800	0.720451
C	6.894025	0.138650	-0.651139
C	5.681941	0.308546	-1.328012
H	5.782745	-0.571313	2.525054
H	7.893117	-0.300028	1.217228
H	7.831629	0.252540	-1.199459
H	5.655007	0.558124	-2.389941
B	0.784889	0.034402	0.256910
H	0.460011	0.704181	1.237403
H	0.456207	-1.139440	0.456241
H	0.276009	0.475030	-0.764920
C	2.704150	0.590003	-2.263645
H	1.609710	0.549723	-2.269074
H	3.109079	-0.136768	-2.984058
H	3.038233	1.600046	-2.551496
C	2.800743	-0.561463	2.546577
H	1.736975	-0.317247	2.647082
H	2.951668	-1.637299	2.732844
H	3.384062	0.015074	3.278887

22_BH3
29

N	-1.231273	0.116435	3.129727
C	-0.955387	1.099862	2.275429
C	-1.925362	2.248442	2.556665
C	-3.753990	2.295779	4.484050
H	-4.132105	3.294977	4.253675
C	-4.295734	1.558547	5.549617
H	-5.097850	1.989078	6.153472
C	-3.813180	0.279954	5.851526
H	-4.243949	-0.277641	6.686364
C	-2.782889	-0.300602	5.095867
H	-2.400369	-1.293639	5.337195
C	-2.269050	0.450172	4.043277
C	-2.729139	1.733323	3.733697
C	-0.533111	-1.156114	3.200912
H	0.144946	-1.224918	2.343762
H	-1.262722	-1.979767	3.176011
H	0.044305	-1.217931	4.136832
C	-2.819698	2.482975	1.320115
H	-3.358761	1.566326	1.035921
H	-2.205258	2.806626	0.468834
H	-3.563418	3.262579	1.546186
C	-1.148388	3.535377	2.902069
H	-1.858913	4.342162	3.141060
H	-0.528940	3.840656	2.047734
H	-0.490915	3.383670	3.770506
B	0.229876	1.145417	1.245047
H	1.148087	1.544236	1.989204
H	0.537970	0.043476	0.803512
H	0.031557	1.986980	0.379531

23_BH3
29

C	-2.981852	0.901053	-1.030705
C	-2.070130	0.345577	-0.017110
N	-2.840397	-0.260465	0.882589
C	-4.302339	0.555478	-0.710277
C	-4.302388	-0.247427	0.568348
C	-2.345344	-0.951144	2.057863
H	-1.304201	-0.648996	2.217434
H	-2.378928	-2.044163	1.918670
H	-2.953107	-0.686569	2.935416
C	-5.088510	0.459798	1.682054
C	-4.799808	-1.684728	0.341074
H	-4.178919	-2.192848	-0.410962
H	-5.837646	-1.672304	-0.024602
H	-4.773613	-2.265461	1.275996
H	-4.667187	1.456802	1.877914
H	-5.075830	-0.122995	2.615646
H	-6.138214	0.581377	1.375944

C	-5.363522	0.950425	-1.519241
C	-5.075082	1.711675	-2.659650
C	-3.754459	2.065683	-2.978804
C	-2.691271	1.660439	-2.168088
H	-1.658624	1.927676	-2.398471
H	-6.394757	0.676964	-1.279990
H	-5.890329	2.037139	-3.310552
H	-3.557994	2.663879	-3.871691
B	-0.497904	0.374910	-0.096787
H	0.070346	0.016656	0.926656
H	-0.297285	-0.455132	-1.000440
H	-0.127359	1.465289	-0.524463

24_BH3
21

O	3.335853	-0.433695	0.965518
N	3.217253	0.599575	-0.949402
C	2.480954	0.101906	0.069012
C	4.623639	-0.257148	0.532834
C	4.578699	0.418269	-0.687073
C	5.810688	-0.651795	1.130738
C	6.986421	-0.306243	0.450475
C	6.952767	0.398733	-0.765307
C	5.743421	0.773171	-1.364238
H	5.818570	-1.193235	2.077322
H	7.949357	-0.590940	0.880476
H	7.891062	0.664919	-1.257183
H	5.717432	1.317797	-2.309016
B	0.930625	0.156433	0.334635
H	0.832333	0.998384	1.233646
H	0.569289	-0.927470	0.775934
H	0.324011	0.524259	-0.662657
C	2.693936	1.199130	-2.164514
H	2.957401	2.267292	-2.209539
H	1.604181	1.085245	-2.155242
H	3.122330	0.682654	-3.036884

25_BH3
17

S	3.156489	-0.696079	1.169853
S	3.019979	0.721408	-1.328484
C	2.167586	0.023046	-0.023385
C	4.721245	-0.271288	0.479768
C	4.653392	0.425347	-0.741970
C	5.962483	-0.570432	1.056199
C	7.124656	-0.165057	0.402581
C	7.056323	0.530519	-0.815402
C	5.824771	0.829756	-1.394570
H	6.013991	-1.103291	2.007907
H	8.095970	-0.391371	0.848430
H	7.974266	0.846054	-1.316331
H	5.767990	1.370363	-2.341744
B	0.601211	0.117238	0.097633
H	0.482007	1.196994	0.704711
H	0.159407	-0.776849	0.804724
H	0.089015	0.239441	-1.006887

26_BH3
21

S	3.192656	-0.747564	1.381344
N	3.212865	0.614322	-0.752467
C	2.344539	0.107068	0.140701
C	4.756873	-0.403064	0.655381
C	4.568607	0.369229	-0.506676
C	6.039752	-0.774623	1.065356
C	7.129120	-0.347976	0.303758
C	6.939899	0.436513	-0.846541
C	5.660619	0.801114	-1.267298
H	6.185827	-1.381290	1.961116
H	8.139087	-0.630274	0.610271
H	7.804574	0.767817	-1.425820
H	5.517140	1.405250	-2.164013
B	0.762982	0.219714	0.132379
H	0.486742	1.422044	0.089919
H	0.307990	-0.331271	1.124059
H	0.371217	-0.309566	-0.912456
C	2.767318	1.327237	-1.943372
H	3.243849	2.317753	-1.989458
H	1.679474	1.438086	-1.887953
H	3.037349	0.749636	-2.840876

27_BH3
17

C	-2.970444	0.908781	-1.039209
C	-2.399075	0.702800	0.358795
N	-2.924079	-0.584658	0.837826
S	-4.580106	0.008544	-1.000405
C	-4.051294	-1.024790	0.296330
C	-2.246500	-1.268151	1.927995
H	-1.334413	-1.767154	1.559196
H	-2.926390	-2.019173	2.346609
H	-1.965169	-0.540537	2.705666
H	-3.125364	1.966843	-1.286749
H	-2.727457	1.498654	1.052692
B	-4.855379	-2.344824	0.660969
H	-5.954164	-2.328914	0.124375
H	-4.895527	-2.498238	1.880406
H	-4.150624	-3.236823	0.167896
H	-1.298509	0.683879	0.358012
H	-2.338701	0.445954	-1.812803

28_BH3
13

C	-3.130962	1.040433	-0.905520
C	-2.279069	0.555909	0.255415
S	-2.332459	-1.288363	0.267285
S	-4.613276	-0.050158	-1.021173
C	-3.913062	-1.464456	-0.360904
H	-2.593737	0.970256	-1.864172
H	-1.230444	0.873215	0.157935
B	-4.689247	-2.827073	-0.276319
H	-5.549080	-2.920552	-1.137676
H	-5.237783	-2.672002	0.833133
H	-3.906937	-3.762650	-0.169597
H	-3.476444	2.075384	-0.763084

H	-2.670037	0.906581	1.223026

29_BH3
15

C	-3.006646	0.949925	-0.989497
C	-2.362460	0.554593	0.135359
N	-2.956416	-0.550028	0.737162
S	-4.370784	-0.094584	-1.280605
C	-4.044300	-1.049613	0.116193
C	-2.413241	-1.182855	1.938441
H	-1.800660	-2.054061	1.662166
H	-3.247047	-1.532948	2.559680
H	-1.803178	-0.454150	2.487770
H	-2.746583	1.779477	-1.643600
H	-1.474499	0.997592	0.583728
B	-4.886143	-2.326243	0.541995
H	-5.825723	-2.485396	-0.223154
H	-5.249541	-2.154557	1.711288
H	-4.094849	-3.276048	0.515347

30_BH3
11

C	-3.140919	0.999778	-0.752702
C	-2.329617	0.423971	0.162186
S	-2.787464	-1.184996	0.659402
S	-4.500878	0.036010	-1.265919
C	-4.203848	-1.332419	-0.283607
H	-3.002581	1.993065	-1.183899
H	-1.436418	0.881971	0.589964
B	-5.154851	-2.590774	-0.197534
H	-5.725863	-2.767184	-1.267128
H	-5.996535	-2.230946	0.640094
H	-4.534809	-3.551810	0.240183

31_BH3
28

C	2.937680	0.655997	-0.255970
N	2.154681	0.096026	-2.235554
C	1.762062	0.256932	-0.933168
C	4.034673	0.722385	-1.190398
C	3.505288	0.365706	-2.405090
C	1.246049	-0.269642	-3.305577
H	0.403359	0.435344	-3.344319
H	0.827708	-1.273554	-3.136667
H	1.787660	-0.246274	-4.261363
P	2.936529	1.179834	1.402859
C	2.606420	-0.125899	2.622063
C	1.729889	2.506785	1.676860
C	4.587004	1.839626	1.795654
H	3.977055	0.272062	-3.381341
H	5.068398	0.997699	-0.989877
H	1.996223	3.353051	1.027320
H	1.708449	2.825623	2.729957
H	0.750519	2.110433	1.368059
H	1.607664	-0.534259	2.409933
H	2.647777	0.287196	3.642017
H	3.355599	-0.921433	2.502595
H	4.843686	2.630235	1.076633
H	5.330340	1.032859	1.721497
H	4.598560	2.253951	2.814604
B	0.259345	0.031424	-0.413637
H	-0.169933	-1.049217	-0.829070
H	-0.463551	0.942420	-0.845896
H	0.231198	0.037977	0.825209

32_BH3
18

N	2.886237	-0.740629	0.725406
C	3.015771	0.446967	-1.209732
C	2.120368	-0.379558	-0.284150
C	4.281057	-0.246273	0.723263
C	4.432403	0.446840	-0.624450
C	2.449623	-1.521201	1.868337
H	3.115114	-2.388833	2.001288
H	1.419202	-1.852876	1.695782
H	2.498665	-0.900308	2.778123
O	2.673253	0.998487	-2.217587
H	4.961154	-1.099660	0.873080
H	4.418309	0.436333	1.578916
H	4.808036	1.478064	-0.561154
H	5.094071	-0.100393	-1.313788
B	0.593415	-0.601055	-0.509213
H	0.366364	-0.762871	-1.700727
H	0.195452	0.551751	-0.237193
H	0.080093	-1.421932	0.236718

33_BH3
19

N	-2.875477	0.058633	-1.182072
C	-2.222134	-0.643110	-0.203401
N	-3.104572	-0.878301	0.815494
C	-4.217432	0.272494	-0.863276
C	-4.377605	-0.371630	0.529887
C	-2.786675	-1.547334	2.070644
H	-3.677577	-1.475164	2.707582
H	-1.934619	-1.053963	2.555306
H	-2.531238	-2.601740	1.895812
C	-2.279085	0.502036	-2.434059
H	-1.870589	-0.356100	-2.984820
H	-1.464427	1.211986	-2.238252
H	-3.071946	0.989007	-3.016231
O	-5.047445	0.828354	-1.526636
O	-5.360106	-0.424778	1.214905
B	-0.756433	-1.163313	-0.313341
H	-0.333136	-1.625400	0.728568
H	-0.051184	-0.321973	-0.855806
H	-0.921502	-2.061951	-1.161807

34_BH3
28

N	-2.781194	-0.042143	1.343230
N	-4.347909	0.774771	-0.189336
C	-4.065612	-0.036079	0.877891
C	-2.403974	-0.726776	2.593528

H	-2.024722	0.014973	3.308559
H	-1.596667	-1.442068	2.384914
H	-3.280283	-1.242870	2.986381
C	-5.743106	0.933041	-0.633039
H	-5.744043	1.642308	-1.464899
H	-6.358534	1.310304	0.192085
H	-6.144353	-0.039178	-0.942506
C	-3.387829	1.525882	-0.907893
C	-1.943423	1.129423	-0.676232
C	-1.714854	0.668722	0.747243
C	-0.994616	2.273797	-1.030399
C	-1.684004	-0.107997	-1.586933
O	-0.663755	0.801045	1.325361
O	-3.713230	2.356084	-1.721350
H	-2.365477	-0.939900	-1.355354
H	-0.649062	-0.453765	-1.447435
H	-1.827072	0.182650	-2.638006
H	-1.165157	3.139942	-0.374830
H	-1.161527	2.590039	-2.067819
H	0.044349	1.944464	-0.899389
B	-5.248731	-0.894835	1.510970
H	-6.089275	-0.090574	1.916387
H	-4.913501	-1.663005	2.385889
H	-5.763059	-1.487984	0.562035

35_BH3
29

N	2.560902	8.606782	0.902760
O	1.132285	8.639079	2.685356
C	3.758888	8.235673	0.309516
N	4.793323	8.056401	1.133130
C	2.246750	8.423569	2.262700
C	3.371068	7.870212	3.134783
C	4.690736	8.365932	2.556538
H	4.788564	9.458826	2.701398
H	5.533295	7.889566	3.077537
C	3.203194	8.363239	4.574324
H	3.335555	9.454545	4.643678
H	3.946241	7.883690	5.229345
H	2.197156	8.119332	4.941079
C	3.290897	6.331446	3.083898
H	3.443870	5.942890	2.065824
H	2.308823	5.990374	3.443367
H	4.064740	5.897375	3.736014
C	6.136392	7.722866	0.669881
C	1.449419	9.056305	0.050862
H	6.807795	8.585949	0.815935
H	6.101273	7.464265	-0.391608
H	6.525600	6.874396	1.253860
H	1.849448	9.662091	-0.767064
H	0.763287	9.635246	0.677158
H	0.911429	8.198918	-0.378151
B	3.818009	8.096271	-1.285463
H	2.801881	7.528220	-1.666325
H	4.822330	7.535235	-1.687615
H	3.789771	9.263032	-1.697217

36_BH3
39

C	-1.908368	-4.034557	-8.713831
C	-2.415129	-5.232971	-8.105305
C	-1.573132	-2.951140	-7.962183
C	-2.550369	-5.294420	-6.680880
C	-1.708257	-2.968643	-6.532092
C	-2.197789	-4.150787	-5.886717
H	-1.795027	-4.014447	-9.800703
C	-2.778279	-6.341472	-8.890670
C	-3.038970	-6.506487	-6.079476
C	-2.320474	-4.163372	-4.453207
C	-3.394855	-7.585080	-6.909235
C	-3.268909	-7.498023	-8.297009
H	-2.671382	-6.278085	-9.976315
C	-3.116779	-6.506281	-4.642266
H	-3.785715	-8.502939	-6.480839
H	-3.559748	-8.349862	-8.915965
C	-1.363016	-1.842812	-5.763685
C	-1.962273	-3.010219	-3.730925
C	-1.490428	-1.867972	-4.379750
H	-1.187880	-2.043259	-8.433779
C	-2.791896	-5.395195	-3.874196
H	-2.046755	-2.990696	-2.649079
H	-1.215945	-0.991923	-3.787477
H	-0.987858	-0.950913	-6.271635
N	-2.974389	-5.772876	-2.540657
N	-3.470139	-7.514265	-3.745744
C	-3.363134	-7.075344	-2.460835
C	-2.821926	-4.922726	-1.369828
H	-3.113394	-5.517922	-0.498384
H	-1.777651	-4.597516	-1.255757
H	-3.473363	-4.041483	-1.453114
C	-3.880961	-8.874413	-4.061144
H	-4.090544	-9.383139	-3.115086
H	-4.792041	-8.869977	-4.676990
H	-3.079883	-9.406994	-4.593713
B	-3.579731	-7.982525	-1.159495
H	-4.716455	-8.457619	-1.193299
H	-2.763647	-8.903598	-1.251683
H	-3.390670	-7.346347	-0.132065

37_BH3
25

C	-1.186706	-1.622093	-3.060678
C	-1.413558	-0.298097	-3.469277
C	-0.851218	-1.861626	-1.727853
C	-1.314668	0.749891	-2.550098
C	-0.744170	-0.789123	-0.797649
C	-0.980961	0.515309	-1.199624
C	-0.565490	-3.141384	-1.074207
C	-0.255544	-2.783143	0.321874
C	-0.366061	-1.378106	0.508223
H	-1.269004	-2.460928	-3.754276
H	-1.679310	-0.082987	-4.506717
H	-1.504240	1.773652	-2.884114
H	-0.910038	1.350453	-0.498911
C	0.095422	-3.615939	1.382928
C	-0.131555	-0.810148	1.751896
C	0.214536	-1.659853	2.820900

C	0.328846	-3.041868	2.642062
H	0.183282	-4.692129	1.222407
H	-0.211771	0.267775	1.911590
H	0.401815	-1.226159	3.806790
H	0.605382	-3.675948	3.487109
B	-0.579614	-4.546414	-1.686281
H	0.554046	-5.009505	-1.440416
H	-1.226239	-5.262923	-0.899752
H	-0.921188	-4.634502	-2.851843

38_BH3
27

B	5.948564	4.645783	12.004454
C	7.415774	4.606797	12.498475
C	7.757187	4.268412	13.883784
C	6.808121	4.469260	14.914207
C	9.007782	3.701977	14.231708
C	7.118385	4.170349	16.236731
C	9.302299	3.371696	15.553226
C	8.365358	3.620153	16.560471
H	5.838219	4.889241	14.650296
H	9.733138	3.482656	13.447928
H	6.384973	4.362856	17.023056
H	10.261737	2.911408	15.800284
H	8.603219	3.370927	17.598072
C	8.525949	5.006459	11.627860
C	9.589877	5.805365	12.113424
C	8.532408	4.647925	10.261354
C	10.615793	6.223039	11.268984
C	9.576934	5.040705	9.426989
C	10.618898	5.830960	9.925131
H	9.583170	6.134261	13.153556
H	7.712692	4.040219	9.878253
H	11.415971	6.856436	11.659440
H	9.576660	4.730770	8.379199
H	11.429246	6.147905	9.263968
H	5.146048	4.093151	12.711207
H	5.843412	4.394698	10.814162
H	5.823391	5.887737	12.009254

39_BH3
31

C	-1.114264	4.290228	10.233471
P	-2.497437	3.347147	10.365743
P	-0.623226	5.459825	9.134009
B	-0.019039	4.022054	11.483547
H	-0.644276	3.782290	12.527681
H	0.716858	3.066823	11.203881
H	0.665476	5.037054	11.661929
C	1.002336	5.066192	8.381393
H	1.679554	4.788965	9.201762
H	0.884173	4.194893	7.720386
H	1.414332	5.915399	7.814762
C	-1.722168	5.797299	7.704106
H	-1.876512	4.887409	7.108329
H	-2.695110	6.167870	8.058884
H	-1.262426	6.567643	7.066996
C	-0.364716	7.128009	9.858101
H	-1.333572	7.520495	10.202007
H	0.294093	7.010886	10.729265
H	0.086452	7.819319	9.129440
C	-2.086078	1.606267	10.747756
H	-1.564600	1.168228	9.884073
H	-1.394821	1.611209	11.601585
H	-2.988266	1.023133	10.987201
C	-3.595546	3.246893	8.899963
H	-4.001915	4.236465	8.648382
H	-3.028895	2.859069	8.041273
H	-4.434162	2.567531	9.114674
C	-3.627400	3.825178	11.732478
H	-4.441207	3.097006	11.873879
H	-3.014506	3.885199	12.643461
H	-4.049009	4.819442	11.520947

40_BH3
27

N	-4.621570	6.420004	0.850315
N	-5.987130	6.520402	0.717307
C	-4.269017	5.175446	0.386591
C	-3.780465	7.596540	0.883971
H	-2.745529	7.240391	0.949993
H	-4.002797	8.213048	1.767823
H	-3.896377	8.207198	-0.028135
C	-6.739254	7.538914	1.410868
H	-6.593616	7.481036	2.504269
H	-7.796215	7.366600	1.176806
H	-6.459231	8.541976	1.054984
C	-6.443514	5.292806	0.308931
C	-5.389917	4.406378	0.029348
O	-7.764162	5.181906	0.272808
C	-8.383266	3.888818	0.216911
H	-9.388482	4.025650	0.638578
H	-7.809540	3.158982	0.802465
H	-8.436779	3.536152	-0.819850
O	-2.963269	4.956420	0.303228
C	-2.488117	3.607197	0.459914
H	-2.630685	3.287038	1.504974
H	-1.418734	3.638440	0.215801
H	-3.029379	2.930744	-0.212490
B	-5.435755	2.940660	-0.702120
H	-6.454653	2.858875	-1.383857
H	-5.406951	2.033631	0.138742
H	-4.468863	2.845965	-1.461347

41_BH3
47

N	-2.274137	-1.625591	-0.190233
N	-2.460755	-1.521896	-2.395978
C	-1.537598	-1.591163	-1.358360
C	-3.630250	-1.578597	-0.479618
C	-3.754061	-1.531653	-1.883716
C	-1.754094	-1.915590	1.134268
H	-1.748664	-1.015312	1.764732
H	-0.721745	-2.269586	1.044649
H	-2.383232	-2.692152	1.597833
C	-2.137134	-1.231838	-3.776418

C	-0.134277	-1.546300	-1.479993
H	-1.207885	-4.085407	-1.460992
H	3.580547	-1.359537	-2.672290
C	0.760218	-3.915836	-4.097220
C	1.885833	-3.068871	-4.096243
C	-1.154989	-4.255191	-2.545440
N	-0.095822	-3.444640	-3.104969
N	1.673736	-2.096744	-3.128532
C	2.586276	-0.980775	-2.958249
C	0.441326	-2.286103	-2.515506
H	-0.922884	-5.315595	-2.723646
H	2.675079	-0.422759	-3.905809
H	-2.142312	-4.027860	-2.979228
H	2.207905	-0.322465	-2.168916
H	-2.933286	-0.610399	-4.209434
H	-2.023029	-2.146914	-4.379430
H	-1.189302	-0.677891	-3.809489
B	0.692552	-0.517372	-0.461517
H	-0.048289	-0.147232	0.441662
H	1.701486	-1.073378	-0.014064
C	2.944797	-3.274516	-4.979980
H	3.819035	-2.622101	-4.979578
C	2.848314	-4.359564	-5.866920
C	0.660355	-4.989329	-4.979732
H	3.663566	-4.550940	-6.568595
H	-0.218117	-5.636802	-4.997333
C	1.728463	-5.201451	-5.867301
H	1.679766	-6.037681	-6.568424
C	-4.755109	-1.592535	0.343773
H	-4.660614	-1.617494	1.430506
C	-5.001410	-1.504058	-2.502558
C	-6.011138	-1.564683	-0.278058
H	-5.098797	-1.477599	-3.589237
H	-6.911430	-1.576317	0.340080
C	-6.133064	-1.520032	-1.674459
H	-7.125726	-1.499380	-2.129660
H	0.997001	0.504202	-1.091997

42_BH3
19

C	2.954558	1.928615	-1.563073
C	1.748408	1.735082	-0.940422
C	4.095749	2.369026	-0.829360
H	0.880248	1.391316	-1.507467
C	1.620753	1.978211	0.475525
C	3.969611	2.620757	0.570189
H	4.843349	2.960749	1.130282
C	2.772692	2.437677	1.212272
H	2.681846	2.631955	2.283561
H	3.053549	1.735350	-2.633256
C	0.403490	1.761791	1.125909
C	0.237658	1.998855	2.522187
N	0.094121	2.190405	3.659601
C	-0.749798	1.280151	0.439063
N	-1.691695	0.874693	-0.108393
C	5.392962	2.585525	-1.514048
H	6.245720	2.365030	-0.854050
H	5.472911	2.015273	-2.449594
H	5.474649	3.660363	-1.781291

43_BH3
17

C	2.978885	2.126159	-1.628063
C	1.798642	1.854867	-1.004362
C	4.131675	2.628106	-0.914859
H	0.936678	1.480134	-1.563759
C	1.652371	2.051856	0.428364
C	3.973229	2.828779	0.507159
H	4.834138	3.215136	1.057411
C	2.804655	2.561848	1.153140
H	2.705896	2.726555	2.229932
H	3.084063	1.972379	-2.704505
C	0.487481	1.756825	1.067141
H	0.380343	1.895559	2.146601
H	-0.375731	1.368457	0.520071
B	5.471135	2.928692	-1.623767
H	6.144842	3.756091	-1.014227
H	6.123401	1.859964	-1.626653
H	5.327294	3.133523	-2.832811

44_BH3
69

C	-1.029080	1.026664	-0.368800
C	0.327972	1.095144	-0.314158
N	0.751775	-0.015343	0.406882
C	-0.304685	-0.779106	0.801517
N	-1.395955	-0.126266	0.319778
C	-2.759341	-0.526721	0.540655
C	-3.417792	-0.033914	1.683593
C	-3.369866	-1.390332	-0.386211
C	-4.695536	-1.769330	-0.135209
C	-5.371799	-1.305187	0.992956
C	-4.741359	-0.443199	1.890307
C	-2.704691	0.845008	2.701873
H	-5.281074	-0.092106	2.772276
H	-5.199589	-2.447317	-0.827435
H	-6.399471	-1.626959	1.181245
C	-2.611392	-1.954444	-1.579461
C	-2.262236	0.002954	3.911994
C	-3.536205	2.060888	3.135219
H	-1.793444	1.236824	2.225309
H	-4.422035	1.767043	3.721040
H	-3.884935	2.646075	2.269250
H	-2.929779	2.722786	3.774494
H	-3.138088	-0.415105	4.435418
H	-1.700190	0.622780	4.628839
H	-1.624046	-0.834997	3.598342
C	-2.210181	-3.416312	-1.314410
H	-1.681572	-1.375861	-1.687731
C	-3.389091	-1.814585	-2.895595
H	-1.632258	-3.502027	-0.384336
H	-1.595790	-3.804723	-2.143324
H	-3.104423	-4.055001	-1.225343
B	-0.242054	-2.161271	1.593261
H	-2.769682	-2.161505	-3.738559
H	-3.682133	-0.769948	-3.089048
H	-4.307111	-2.424020	-2.894383

C	2.119665	-0.294126	0.747769
C	2.859948	-1.151832	-0.088951
C	2.653563	0.290452	1.911693
C	4.188438	-1.411675	0.267906
C	3.987584	-0.003414	2.225716
C	4.746432	-0.845869	1.415048
H	5.779085	-1.077918	1.688866
H	4.434397	0.427492	3.124315
C	2.246073	-1.756307	-1.343371
H	4.792862	-2.073173	-0.355728
C	1.825392	1.178372	2.831970
C	2.591678	-0.914846	-2.582529
H	1.154994	-1.729042	-1.214445
C	2.627106	-3.227239	-1.549521
C	2.488682	2.546435	3.059054
C	1.526808	0.470400	4.164907
H	0.858198	1.361556	2.341431
H	3.432333	2.452224	3.620843
H	1.824688	3.202597	3.644067
H	2.717497	3.050660	2.106624
H	2.456887	0.241311	4.710538
H	0.991633	-0.473359	3.991834
H	0.905725	1.112449	4.810658
H	2.261293	0.129833	-2.468621
H	2.102094	-1.328352	-3.479393
H	3.679686	-0.907347	-2.760680
H	3.693875	-3.349693	-1.800092
H	2.047104	-3.651167	-2.384336
H	2.407624	-3.818312	-0.648368
H	-1.768857	1.677911	-0.823300
H	1.031243	1.821684	-0.709184
H	-1.375120	-2.531551	1.861555
H	0.446719	-2.009970	2.597659
H	0.328201	-2.962355	0.847779

45_BH3
51

C	-1.167921	0.866810	-0.215734
C	0.182725	1.018962	-0.223612
N	0.703739	-0.018414	0.543413
C	-0.287401	-0.813445	1.031164
N	-1.436314	-0.260158	0.555927
C	-2.759684	-0.753381	0.815525
C	-3.420170	-0.337591	1.979962
C	-3.344118	-1.632906	-0.108967
C	-4.645134	-2.077163	0.147049
C	-5.349728	-1.675671	1.290698
C	-4.721394	-0.809083	2.193364
C	-2.727829	0.548942	2.980857
H	-5.254067	-0.494817	3.095409
H	-5.117068	-2.764097	-0.561420
C	-6.738246	-2.199962	1.558205
C	-2.564887	-2.112879	-1.304706
C	2.105321	-0.206148	0.792444
C	2.850839	-0.989956	-0.101081
C	2.678354	0.403998	1.917740
C	4.220011	-1.135515	0.146544
C	4.051940	0.229916	2.123689
C	4.837852	-0.533995	1.251315
C	6.308217	-0.739445	1.516104
H	4.517635	0.699224	2.994962
C	2.177435	-1.684501	-1.254456
H	4.818097	-1.743100	-0.538668
C	1.827296	1.185837	2.883304
H	-2.205130	-1.276378	-1.925695
H	-3.176523	-2.771249	-1.937398
H	-1.675677	-2.676835	-0.976686
H	-2.299286	1.446554	2.506530
H	-1.893612	0.004044	3.453543
H	-3.421658	0.872957	3.769286
H	1.096974	0.518224	3.369685
H	1.253693	1.978246	2.375493
H	2.443323	1.653888	3.663917
H	1.602938	-0.981989	-1.879631
H	1.466324	-2.437865	-0.875904
H	2.912041	-2.193372	-1.894252
H	-7.326793	-2.275242	0.630384
H	-7.285679	-1.556276	2.262944
H	-6.696321	-3.211411	1.998909
H	6.736780	0.095682	2.090712
H	6.877353	-0.841830	0.579302
H	6.474807	-1.660616	2.101666
H	0.823694	1.757477	-0.695608
H	-1.962638	1.444082	-0.678451
B	-0.133840	-2.082205	1.979784
H	0.904709	-2.668603	1.688794
H	-0.044340	-1.641746	3.135023
H	-1.132543	-2.784211	1.872363

46_BH3
53

C	-1.275075	1.096094	-0.088932
C	0.251461	1.296463	-0.125264
N	0.749857	0.087642	0.544674
C	-0.232221	-0.744778	0.934705
N	-1.412605	-0.198391	0.592014
C	-2.700830	-0.774740	0.819068
C	-3.386994	-0.466730	2.007289
C	-3.256799	-1.614012	-0.158311
C	-4.541922	-2.126027	0.064071
C	-5.263212	-1.826731	1.225168
C	-4.666016	-0.999994	2.188174
C	-2.721695	0.361208	3.074575
H	-5.208118	-0.773666	3.111307
H	-4.984942	-2.784642	-0.688436
C	-6.647113	-2.381866	1.451427
C	-2.464733	-1.991204	-1.382968
C	2.152421	-0.134119	0.708907
C	2.861426	-0.810086	-0.299887
C	2.789807	0.350594	1.861188
C	4.241423	-0.968755	-0.142642
C	4.173819	0.169173	1.977499
C	4.916519	-0.482010	0.986025
C	6.405440	-0.677848	1.126109
H	4.681842	0.538179	2.873073
C	2.134763	-1.388264	-1.486083
H	4.804441	-1.496112	-0.918609
C	1.987240	0.987119	2.964823

H	-2.105676	-1.106128	-1.933538
H	-3.067758	-2.597197	-2.073961
H	-1.575158	-2.574632	-1.094206
H	-2.365206	1.329961	2.687840
H	-1.841593	-0.172004	3.471144
H	-3.409066	0.561501	3.908704
H	1.303334	0.244865	3.409433
H	1.365470	1.820996	2.600117
H	2.640873	1.373887	3.759522
H	2.839086	-1.827427	-2.206776
H	1.536826	-0.628902	-2.016502
H	1.437646	-2.175961	-1.156568
H	-6.698114	-2.949507	2.395347
H	-6.953409	-3.053266	0.635882
H	-7.393922	-1.572819	1.522176
H	6.792483	-0.203175	2.039715
H	6.947506	-0.251741	0.265478
H	6.661445	-1.749953	1.168577
H	0.578803	2.199008	0.415551
H	0.650233	1.360858	-1.150562
H	-1.725145	1.052713	-1.093854
H	-1.797824	1.885194	0.475361
B	-0.029627	-2.158164	1.637369
H	-1.012371	-2.419593	2.319527
H	1.022696	-2.155247	2.263295
H	0.047805	-2.961384	0.696640

47_BH3
56

C	-1.126049	2.105622	-0.258526
C	-0.797540	0.677669	-0.711820
C	-1.463679	0.438455	-2.067709
C	0.715404	0.391223	-0.766582
H	0.932559	-0.239272	-1.641999
H	1.302161	1.314336	-0.879518
C	1.067515	-0.388703	0.521838
C	1.571214	0.541018	1.648387
C	-0.269158	-0.966301	0.962111
N	-1.239279	-0.346458	0.318904
C	-2.641152	-0.577611	0.589056
C	-3.272684	0.163582	1.612466
C	-3.334981	-1.558357	-0.152100
C	-4.708398	-1.708100	0.082334
C	-5.365193	-0.941482	1.041383
C	-4.646438	-0.029225	1.809938
C	-2.504804	1.045928	2.588598
H	-5.156851	0.530523	2.596342
H	-5.266683	-2.459237	-0.480245
H	-6.433718	-1.086690	1.220651
C	-2.635408	-2.545243	-1.077261
C	-2.392308	0.341506	3.952644
C	-3.100264	2.452461	2.735643
H	-1.482130	1.158962	2.210473
H	-4.101688	2.427274	3.195099
H	-3.188698	2.963258	1.764765
H	-2.461375	3.069590	3.387055
H	-3.387253	0.184752	4.400329
H	-1.798841	0.953513	4.651192
H	-1.906543	-0.638030	3.845820

C	-2.607487	-3.938633	-0.424161
H	-1.589639	-2.226130	-1.183755
C	-3.256914	-2.613216	-2.478804
H	-2.140096	-3.894695	0.568004
H	-2.029867	-4.640028	-1.047977
H	-3.626577	-4.343510	-0.313225
H	-1.061321	1.166796	-2.788159
H	-2.554136	0.573145	-2.017607
H	-1.249269	-0.565260	-2.454354
H	-0.772409	2.812031	-1.024178
H	-0.642108	2.370617	0.689712
H	-2.211149	2.242843	-0.150482
H	2.507186	1.028791	1.331954
H	1.760872	-0.038681	2.562066
H	0.844218	1.329049	1.894782
C	2.118045	-1.474304	0.260390
H	3.038457	-1.011129	-0.132036
H	1.756773	-2.208403	-0.475150
H	2.358757	-2.017788	1.184054
B	-0.414733	-2.150929	1.988996
H	-2.658380	-3.272775	-3.127045
H	-3.312512	-1.624350	-2.957541
H	-4.277029	-3.029297	-2.449089
H	0.403862	-2.008037	2.895772
H	-1.556570	-2.336664	2.373051
H	-0.026808	-3.105694	1.296267

48_BH3
75

C	-1.234466	2.203394	0.124620
C	-0.916153	0.869623	-0.562183
C	-1.680504	0.829818	-1.887969
C	0.598344	0.660330	-0.736809
H	0.818157	0.298484	-1.743498
H	1.132377	1.612732	-0.623562
C	1.057258	-0.362340	0.344307
C	1.722857	0.460961	1.495061
C	-0.251300	-0.955177	0.860731
N	-1.268052	-0.294772	0.338466
C	-2.655220	-0.608048	0.611524
C	-3.309074	-0.022364	1.716988
C	-3.314769	-1.516765	-0.246995
C	-4.679541	-1.747340	-0.036314
C	-5.362869	-1.123404	1.005963
C	-4.677012	-0.284638	1.879738
C	-2.576872	0.749704	2.808484
H	-5.206719	0.157048	2.726350
H	-5.210193	-2.445706	-0.686692
H	-6.425743	-1.325200	1.162562
C	-2.569525	-2.350534	-1.281253
C	-2.566683	-0.069863	4.111510
C	-3.150428	2.151408	3.065141
H	-1.530404	0.862587	2.497212
H	-4.171377	2.101505	3.478027
H	-3.187983	2.760050	2.150485
H	-2.529181	2.685049	3.802062
H	-3.585283	-0.182061	4.517036
H	-1.953933	0.438433	4.873687
H	-2.145483	-1.068530	3.940038

C	-2.340307	-3.774964	-0.742582
H	-1.576670	-1.909671	-1.420328
C	-3.252296	-2.383703	-2.653833
H	-1.807540	-3.750822	0.217618
H	-1.745360	-4.363105	-1.460350
H	-3.298944	-4.295697	-0.585640
H	-1.333632	1.665757	-2.514216
H	-2.763181	0.946106	-1.734035
H	-1.507545	-0.097214	-2.445836
H	-0.926732	3.024713	-0.539966
H	-0.700023	2.318329	1.076400
H	-2.312115	2.304955	0.308721
H	2.401872	1.184528	1.009812
C	2.540732	-0.302628	2.532053
H	0.944334	1.061862	1.994248
C	2.048270	-1.439393	-0.245226
H	2.732443	-0.855496	-0.892570
C	1.348603	-2.516383	-1.147088
C	2.941898	-2.099472	0.825082
B	-0.417005	-2.089149	1.956094
H	-2.619522	-2.924374	-3.375853
H	-3.432736	-1.371798	-3.048181
H	-4.221153	-2.907199	-2.617988
C	3.594862	-1.122447	1.795880
C	3.149964	0.656084	3.554067
H	3.727914	-2.672778	0.313614
H	2.342632	-2.819386	1.401167
H	4.220100	-1.679013	2.514894
H	4.276065	-0.436666	1.254446
C	0.964001	-2.020405	-2.551873
H	0.426695	-2.820158	-0.626872
C	2.175265	-3.800992	-1.326949
H	0.175324	-1.261114	-2.554228
H	0.584908	-2.857925	-3.158401
H	1.836872	-1.597263	-3.078237
H	3.145443	-3.593573	-1.811126
H	1.627318	-4.500784	-1.977690
H	2.370687	-4.323008	-0.381641
H	1.871589	-0.999200	3.061133
H	3.825670	1.380414	3.065653
H	3.738602	0.106079	4.306440
H	2.370589	1.227307	4.086236
H	0.375978	-3.001323	1.779817
H	-1.568169	-2.475764	2.058764
H	-0.083466	-1.495667	2.989410

49_BH3
37

C	-0.432933	11.925808	4.396418
C	1.024026	11.126667	2.620393
C	0.924151	12.350060	1.860753
O	1.468849	12.512382	0.747559
C	0.036456	13.318511	2.458020
C	0.622102	9.766895	4.627653
C	-1.027905	14.278621	4.443934
C	3.000110	10.241272	1.466313
H	3.396241	11.233022	1.707340
H	2.938342	10.159146	0.368256
H	3.701503	9.473663	1.842926
C	1.054168	8.783941	1.822148
H	-0.010596	8.827475	2.086393
H	1.517205	7.944647	2.372961
H	1.124791	8.559655	0.741816
C	0.521873	15.159133	0.891315
H	1.547172	14.802240	1.020070
H	0.462500	16.236693	1.135196
H	0.252111	15.020844	-0.169607
C	-1.790358	14.728420	1.615503
H	-2.054865	14.730829	0.542452
H	-2.051933	15.716675	2.035654
H	-2.405500	13.965683	2.108768
N	0.361682	10.977721	3.829735
N	-0.493875	13.107731	3.719070
N	1.704881	10.052631	2.102037
N	-0.378117	14.420879	1.764340
H	1.601405	9.374194	4.335749
H	-0.151192	9.006117	4.459832
H	0.608531	10.045752	5.685139
H	-0.565068	15.171557	4.009075
H	-0.761771	14.178650	5.499970
H	-2.119773	14.350774	4.369181
B	-1.232698	11.665097	5.773931
H	-2.150684	12.461717	5.895946
H	-1.663843	10.515158	5.759682
H	-0.430663	11.787206	6.711605

50_BH3
38

P	1.068778	-1.286682	-0.413157
B	1.059310	0.254529	-1.580114
H	2.226285	0.428942	-1.897734
H	0.343551	-0.080373	-2.513329
H	0.596052	1.157462	-0.897848
C	1.780282	-2.794796	-1.165707
C	2.851046	-3.491760	-0.589891
C	1.216944	-3.257593	-2.367025
C	3.352944	-4.641722	-1.207766
C	1.712764	-4.412072	-2.973217
C	2.783255	-5.105082	-2.395659
H	3.294481	-3.136844	0.342160
H	0.388539	-2.710892	-2.824112
H	4.187367	-5.180600	-0.752032
H	1.261782	-4.774334	-3.900539
H	3.176543	-6.004289	-2.876676
C	-0.609432	-1.805703	0.100755
C	-1.610340	-0.825624	0.190327
C	-0.923766	-3.144502	0.385483
C	-2.909311	-1.183925	0.558208
C	-2.225319	-3.497922	0.750522
C	-3.219661	-2.518536	0.835084
H	-1.368935	0.212146	-0.049022
H	-0.153992	-3.914997	0.304138
H	-3.684968	-0.416842	0.620501
H	-2.466971	-4.541925	0.966369
H	-4.237780	-2.798907	1.117599
C	2.000510	-1.027254	1.138469
C	1.600478	-1.597579	2.356569
C	3.144850	-0.213837	1.096625

C	2.336607	-1.355876	3.519183
C	3.879979	0.022106	2.260480
C	3.475346	-0.545049	3.473120
H	0.705152	-2.220478	2.401565
H	3.448656	0.242802	0.152474
H	2.011065	-1.798882	4.464040
H	4.767125	0.658956	2.220637
H	4.045789	-0.346458	4.384154

51_BH3
18

C	-4.686060	-1.647473	-9.903979
C	-4.686060	-0.449126	-9.191535
C	-4.686060	-0.505227	-7.796739
N	-4.686060	-1.647473	-7.097709
C	-4.686060	-2.789719	-7.796739
C	-4.686060	-2.845820	-9.191535
H	-4.686060	0.509532	-9.700752
H	-4.686060	0.412871	-7.211575
H	-4.686060	-3.707817	-7.211575
H	-4.686060	-3.804478	-9.700752
C	-4.686060	-1.647473	-5.627709
H	-5.706752	-1.656380	-5.228689
H	-4.168000	-2.526964	-5.228689
H	-4.183428	-0.759074	-5.228689
B	-4.686060	-1.647473	-11.443979
H	-3.665368	-1.656380	-11.842999
H	-5.204120	-2.526964	-11.842999
H	-5.188692	-0.759074	-11.842999

CH_2 Radicals

01_CH2_rad
30

C	-2.978831	0.157256	-1.288582
C	-2.138048	-0.444129	-0.172991
N	-2.774702	-0.412883	0.995783
C	-4.136346	0.813663	-0.496142
C	-4.154849	0.180301	0.909515
C	-2.253575	-0.900184	2.265680
H	-1.518542	-1.697341	2.108531
H	-3.080987	-1.295467	2.867629
H	-1.781041	-0.072093	2.817910
C	-2.181903	1.199428	-2.090783
H	-1.388088	0.732788	-2.693520
H	-1.726071	1.955350	-1.432496
H	-2.860364	1.718688	-2.785612
C	-4.352183	1.218471	2.016519
C	-5.183294	-0.955451	1.030608
H	-5.101650	0.679219	-1.001767
H	-3.955412	1.894400	-0.405684
C	-3.469929	-0.966272	-2.226239
H	-4.053944	-1.729239	-1.691706
H	-2.628333	-1.468328	-2.727822
H	-4.112752	-0.529977	-3.005723
H	-5.029299	-1.727921	0.264525
H	-6.186736	-0.526373	0.891865
H	-5.158784	-1.432947	2.021487
H	-3.549623	1.971712	2.009490
H	-4.408753	0.757749	3.013828
H	-5.304692	1.740007	1.840310
C	-0.855950	-0.978283	-0.356519
H	-0.415235	-0.971594	-1.354505
H	-0.264120	-1.400422	0.455996

02_CH2_rad
18

N	3.001424	-0.538150	0.957526
N	3.024205	0.534155	-0.972452
C	2.183892	0.004533	-0.013641
C	4.306786	-0.346711	0.605641
C	4.321048	0.322867	-0.600892
C	2.521721	-1.199923	2.168785
H	1.855093	-2.032886	1.903890
H	1.982758	-0.482510	2.805133
H	3.383230	-1.596217	2.719516
C	2.573486	1.199783	-2.192638
H	1.917470	2.045067	-1.940507
H	2.031824	0.489138	-2.834189
H	3.449568	1.579097	-2.732166
C	0.801809	0.015484	-0.023846
H	0.254715	0.480218	-0.843042
H	0.235506	-0.439658	0.787631
H	5.163094	0.656216	-1.203289
H	5.134500	-0.692612	1.220669

03_CH2_rad
20

N	2.947736	-0.511398	1.007548
N	2.935095	0.478695	-0.977345
C	2.180960	-0.015267	0.020524
C	4.367131	-0.462528	0.631789
C	4.359418	0.426692	-0.621224
C	2.528792	-1.229656	2.195521
H	2.658267	-2.318022	2.063609
H	1.480766	-1.013673	2.434215
H	3.145590	-0.904402	3.046166
C	2.500596	1.202823	-2.156242
H	2.622462	2.291232	-2.017379
H	1.452473	0.979899	-2.388357
H	3.113130	0.888500	-3.013958
H	4.728032	-1.485338	0.424882
H	4.968311	-0.045948	1.452961
H	4.725455	1.448647	-0.419243
H	4.948231	0.008744	-1.450591
C	0.756992	-0.013410	0.029989
H	0.195700	0.450182	-0.779530
H	0.204943	-0.476001	0.846395

04_CH2_rad
12

O	-6.425776	1.769240	0.386977
C	-5.514772	2.687173	0.360097
O	-4.310817	2.274950	0.128574
C	-4.323242	0.819576	-0.062371

C	-5.807435	0.460161	0.152505
H	-3.638626	0.390356	0.679600
H	-3.949415	0.624039	-1.076302
H	-6.005566	-0.152886	1.041620
H	-6.300739	0.020837	-0.723972
C	-5.816572	4.058760	0.571489
H	-5.006352	4.790100	0.541158
H	-6.850508	4.352125	0.764274

05_CH2_rad
16

C	-3.139918	0.892802	-0.993439
C	-2.090415	0.494635	0.061495
N	-2.863186	-0.398739	0.938266
O	-4.302628	0.079113	-0.666434
C	-4.082416	-0.591458	0.442528
C	-2.252944	-1.018913	2.104529
H	-1.460325	-1.712549	1.781323
H	-3.000832	-1.567556	2.688120
H	-1.804587	-0.237867	2.737469
C	-5.108534	-1.412993	0.971790
H	-6.055381	-1.461596	0.431282
H	-4.971508	-1.982219	1.890198
H	-1.231918	-0.055419	-0.357191
H	-1.707432	1.349022	0.640722
H	-3.451006	1.944875	-0.928915
H	-2.844640	0.652884	-2.023166

06_CH2_rad
14

C	-6.529090	1.782350	-0.051965
C	-5.465613	2.766424	0.307400
O	-4.287485	2.244119	0.325079
C	-4.328127	0.806952	-0.037826
C	-5.802643	0.430768	0.105712
H	-3.629329	0.303264	0.640813
H	-3.933970	0.758918	-1.065402
H	-5.999502	0.002701	1.099396
H	-6.108822	-0.306696	-0.646708
H	-7.428324	1.902433	0.570822
H	-6.843799	1.989356	-1.094119
C	-5.587950	4.144095	0.597113
H	-6.569571	4.623230	0.588201
H	-4.692824	4.726566	0.834093

07_CH2_rad
22

C	-0.647099	2.604446	0.092697
N	-1.865098	2.124739	-0.253213
C	-2.121308	0.694515	-0.418251
C	-3.053444	2.979372	-0.303561
N	0.471810	1.943745	-0.292492
C	1.740089	2.078802	0.424978
C	0.556799	1.229487	-1.566438
H	1.574519	2.370590	1.468372
H	2.401692	2.809883	-0.069798
H	2.247105	1.102377	0.425334
H	0.584435	0.136352	-1.432890
H	1.487677	1.539163	-2.066472
H	-0.286604	1.500713	-2.211607
H	-3.711344	2.613957	-1.105253
H	-2.781877	4.015676	-0.536660
H	-3.614220	2.942162	0.645839
H	-2.239492	0.408751	-1.475567
H	-3.056209	0.452924	0.109521
H	-1.310036	0.109571	0.029353
C	-0.540450	3.801221	0.856706
H	-1.425465	4.289756	1.261047
H	0.429948	4.257868	1.046526

08_CH2_rad
24

N	2.894043	-0.527861	0.958794
N	2.916908	0.505922	-0.990635
C	2.074354	-0.021657	-0.031322
C	4.209656	-0.318580	0.626109
C	4.224455	0.327935	-0.610742
C	2.392368	-1.176024	2.164831
H	1.713772	-1.997752	1.894657
H	1.854709	-0.450937	2.794464
H	3.233024	-1.589421	2.732025
C	2.444058	1.147293	-2.212088
H	1.744800	1.958588	-1.963850
H	1.937892	0.414510	-2.858553
H	3.296162	1.573544	-2.752134
C	5.353247	-0.724147	1.485440
C	5.389190	0.761096	-1.427488
C	0.698548	-0.039896	-0.056929
H	5.399653	1.853269	-1.578616
H	5.381585	0.281501	-2.419604
H	6.326526	0.482225	-0.931040
H	6.301084	-0.415815	1.027807
H	5.388333	-1.816634	1.630269
H	5.293344	-0.251379	2.479028
H	0.150176	0.393782	-0.891686
H	0.130904	-0.488633	0.756674

09_CH2_rad
20

N	3.015242	-0.382286	0.907799
N	2.999366	0.585316	-0.946238
N	2.213975	0.147757	0.005422
C	4.330010	-0.307833	0.567618
C	4.343149	0.353527	-0.682068
C	2.440486	-0.953102	2.125854
H	2.666703	-2.027650	2.173637
H	1.356776	-0.794645	2.085961
H	2.862908	-0.441567	3.001371
C	2.429330	1.209163	-2.134746
H	2.768543	2.252970	-2.205232
H	1.338254	1.172481	-2.040533
H	2.748493	0.650419	-3.025875
C	5.443363	-0.865622	1.372181
H	5.339771	-0.611067	2.438264
H	6.404162	-0.470265	1.018311

H	5.475330	-1.965882	1.284788
C	5.408797	0.728144	-1.504456
H	5.247835	1.264292	-2.439601
H	6.429507	0.486550	-1.207848

10_CH2_rad
17

C	-0.686689	2.561555	0.091773
C	-1.995407	1.820203	0.067726
H	-1.977919	0.944069	0.735355
H	-2.237486	1.466172	-0.947307
H	-2.804709	2.480400	0.401853
N	0.440942	1.967612	-0.330551
C	1.728892	2.678867	-0.385669
C	0.485587	0.574707	-0.773290
H	2.076067	2.923134	0.630028
H	1.633618	3.602047	-0.973378
H	2.468010	2.028949	-0.866108
H	-0.473493	0.072953	-0.626579
H	1.264678	0.046617	-0.202036
H	0.762831	0.542354	-1.840549
C	-0.648792	3.886188	0.562058
H	0.267029	4.473791	0.625302
H	-1.573401	4.357761	0.898651

11_CH2_rad
14

C	-3.147073	0.856216	-0.881850
C	-2.233802	0.545756	0.088251
N	-2.826438	-0.388288	0.901374
O	-4.266649	0.133249	-0.670213
C	-4.081966	-0.643522	0.418694
C	-2.243791	-0.995586	2.099710
H	-2.161275	-2.084061	1.966135
H	-2.877300	-0.769081	2.969409
H	-1.245473	-0.571435	2.257835
H	-3.126765	1.530242	-1.735702
H	-1.225190	0.919410	0.253257
C	-5.040434	-1.516972	0.879476
H	-5.991513	-1.587440	0.349332
H	-4.860900	-2.134638	1.759284

12_CH2_rad
20

N	8.095261	-0.372849	3.575637
N	9.933978	0.008884	4.661679
C	8.685604	0.538334	4.406111
N	8.903737	-1.406503	3.314649
C	10.026635	-1.172197	3.973938
C	6.767289	-0.297187	2.978248
H	6.621958	-1.218425	2.402892
H	6.702997	0.573105	2.308580
H	6.003232	-0.223127	3.765336
C	10.983114	0.637279	5.459524
H	10.533606	1.188631	6.294816
H	11.575985	1.325400	4.837931
H	11.638711	-0.140888	5.867917
C	11.215284	-2.058950	3.949435
H	10.991109	-2.930958	3.322937
H	11.476612	-2.408471	4.961514
H	12.089254	-1.533902	3.531584
C	8.140353	1.723569	4.880249
H	7.133442	2.022255	4.589720
H	8.710988	2.380329	5.535921

13_CH2_rad
18

N	3.036541	-0.958543	0.732755
N	3.045913	0.194387	-1.167198
C	2.216533	-0.444043	-0.259053
C	4.337830	-0.646060	0.444177
C	4.343713	0.070986	-0.750711
C	2.553268	-1.698045	1.895720
H	1.962601	-2.563581	1.563817
H	1.936224	-1.042375	2.527250
H	3.416774	-2.050392	2.470547
C	2.577310	0.884093	-2.367042
H	1.882159	1.687864	-2.085698
H	2.075224	0.171629	-3.037353
H	3.441702	1.319226	-2.880878
C	0.848048	-0.546333	-0.328829
H	0.298621	-0.097684	-1.155130
H	0.289045	-1.079561	0.438491
Cl	5.678211	0.691186	-1.575957
Cl	5.661522	-1.069698	1.401718

14_CH2_rad
20

N	2.949655	-0.538438	0.962085
N	2.980008	0.536457	-0.977012
C	2.143140	0.006510	-0.016208
C	4.263055	-0.351305	0.617825
C	4.282183	0.326746	-0.604293
C	2.463450	-1.198499	2.175972
H	1.800108	-2.031327	1.903756
H	1.922898	-0.474193	2.802591
H	3.323817	-1.590640	2.731764
C	2.532537	1.200147	-2.203725
H	1.880468	2.047489	-1.949660
H	1.991180	0.483262	-2.838026
H	3.412014	1.571951	-2.743270
C	0.760351	0.019330	-0.030757
H	0.216715	0.484604	-0.852428
H	0.191189	-0.434546	0.779907
C	5.404459	0.753528	-1.354963
C	5.361743	-0.796025	1.392300
N	6.260706	-1.171686	2.022856
N	6.320933	1.108766	-1.971955

15_CH2_rad
24

N	2.865581	-1.001424	0.648310
C	3.020393	0.936080	-0.474079
C	2.095107	-0.015514	-0.006425

C	4.169736	-0.684152	0.574307
N	4.276779	0.476276	-0.112532
C	2.788022	2.215877	-1.188608
H	3.341973	3.044858	-0.720205
H	1.720975	2.471943	-1.160844
H	3.094868	2.162116	-2.248173
C	2.271400	-2.161477	1.301742
H	1.752982	-2.785873	0.559259
H	1.549837	-1.826117	2.060244
H	3.047206	-2.758485	1.791374
C	5.530157	1.173968	-0.395905
C	5.320314	-1.438464	1.135866
H	5.472992	1.635075	-1.390018
H	6.361670	0.460491	-0.390041
H	5.715625	1.957173	0.354287
H	4.985986	-2.258484	1.780651
H	5.963292	-0.776097	1.735807
H	5.936412	-1.872145	0.330186
C	0.707802	-0.051828	-0.124490
H	0.188712	0.742121	-0.660028
H	0.111251	-0.859699	0.297365

16_CH2_rad
18

N	-2.954450	0.382397	-0.964581
C	-2.145848	-0.077488	0.057086
C	-3.018115	-0.534974	1.086347
N	-4.252909	0.211062	-0.606128
C	-4.304463	-0.335956	0.632943
C	-2.581778	0.907458	-2.268459
H	-2.737425	0.155302	-3.057143
H	-1.521952	1.183175	-2.238415
H	-3.159885	1.813036	-2.495906
C	-0.748920	-0.078632	0.041407
H	-0.164764	0.295424	-0.799126
H	-0.217755	-0.472043	0.908584
C	-5.364350	0.589165	-1.465543
H	-5.317412	0.045209	-2.419734
H	-6.289626	0.317819	-0.943462
H	-5.363078	1.673554	-1.652194
H	-5.264391	-0.544925	1.101409
H	-2.711861	-0.958991	2.039406

17_CH2_rad
24

N	8.896708	4.668784	14.488318
N	6.071360	4.380081	12.080857
C	8.554503	5.195469	11.944899
C	8.376711	4.806069	13.286224
C	7.316122	4.686180	12.380806
C	10.241404	5.175400	14.775399
C	8.159848	4.051162	15.588695
C	5.107221	3.927361	13.079954
C	5.592691	4.501936	10.702916
H	10.680743	5.604687	13.866861
H	10.883511	4.359028	15.141514
H	10.190733	5.957160	15.550300
H	7.318152	3.469312	15.194993
H	7.787551	4.813737	16.292216
H	8.828429	3.368076	16.133967
H	4.204402	4.554754	13.029540
H	5.539282	4.014160	14.083686
H	4.815186	2.881002	12.891077
H	4.755945	5.216233	10.656418
H	5.238522	3.523174	10.341183
H	6.403735	4.853944	10.054754
C	9.349170	5.699879	10.939654
H	10.377124	6.005123	11.140567
H	8.960176	5.809610	9.926132

18_CH2_rad
23

C	7.844925	5.735500	0.549426
C	6.372720	5.370572	0.555447
C	8.149555	6.566423	-0.686429
H	8.468989	4.827622	0.544000
H	8.093105	6.300148	1.459385
N	5.923338	4.906795	-0.767101
H	5.749840	6.231939	0.853153
H	6.169940	4.561983	1.275788
C	6.673346	5.038524	-1.884046
N	7.777583	5.815926	-1.891626
H	9.218870	6.803952	-0.751900
H	7.596491	7.523674	-0.665887
C	8.572807	6.017349	-3.107834
C	4.593186	4.292621	-0.785425
H	8.972521	5.067931	-3.494355
H	9.420939	6.664153	-2.858223
H	7.984531	6.515585	-3.894015
H	4.642529	3.196148	-0.679552
H	4.048087	4.556162	-1.700449
H	4.023821	4.695163	0.062586
C	6.297180	4.333712	-3.074571
H	6.862443	4.447134	-3.996445
H	5.459013	3.641134	-3.074438

19_CH2_rad
31

C	-0.331362	-0.072597	0.082897
C	1.198507	0.175702	-0.045607
C	1.377301	1.671539	-0.209175
N	0.684975	2.204958	-1.234952
C	-0.077698	1.227462	-2.080047
C	-1.072964	0.517764	-1.128230
C	0.975404	0.203295	-2.573339
C	1.676752	-0.465537	-1.379795
C	-0.797218	1.875651	-3.253815
C	1.945238	-0.412384	1.148831
H	0.447509	-0.530055	-3.200086
H	1.690314	0.727806	-3.226037
H	1.446732	-1.540317	-1.332213
H	-0.108660	2.399122	-3.932916
H	-1.282541	1.076993	-3.832647
H	-1.589579	2.567487	-2.933354
H	-1.846949	1.236835	-0.822052
H	-1.577137	-0.263494	-1.716396

H	-0.691160	0.369459	1.023894
H	-0.489678	-1.158311	0.158949
H	1.599934	0.021064	2.100073
H	1.760361	-1.495571	1.193696
H	3.034194	-0.271753	1.068453
H	2.770094	-0.373561	-1.456743
C	2.212028	2.440731	0.610572
H	2.755925	1.962435	1.423475
H	2.368970	3.509341	0.466278
C	0.712496	3.628218	-1.563202
H	1.531283	3.840362	-2.269829
H	-0.232918	3.915820	-2.031348
H	0.847905	4.230009	-0.658832

20_CH2_rad
24

N	-2.982625	0.866905	-1.008687
C	-2.031840	0.400268	-0.087380
N	-2.795405	-0.172933	0.941596
C	-4.279992	0.583865	-0.578860
C	-4.160328	-0.078078	0.665213
C	-2.337269	-0.770766	2.091399
C	-5.375139	0.956871	-1.374511
C	-5.142402	1.603135	-2.572941
C	-3.807491	1.881434	-2.982086
C	-2.746861	1.508728	-2.200495
H	-1.707801	1.695389	-2.467680
H	-6.385025	0.724701	-1.032745
H	-5.977109	1.903771	-3.207719
H	-3.610578	2.392840	-3.925199
C	-5.093132	-0.604798	1.572318
C	-3.238306	-1.292729	2.981078
C	-4.636816	-1.213538	2.725479
H	-1.258914	-0.797191	2.241194
H	-2.864457	-1.767382	3.889311
H	-6.157383	-0.522361	1.346197
H	-5.343751	-1.631143	3.443629
C	-0.678832	0.474829	-0.178073
H	-0.196846	0.951364	-1.029775
H	-0.039288	0.054460	0.595638

21_CH2_rad
24

N	3.287448	-0.552128	0.854965
N	3.211329	0.708107	-0.981324
C	2.435026	0.102633	-0.012382
C	4.586644	-0.364445	0.442128
C	4.537768	0.441932	-0.731502
C	2.849746	-1.290990	2.032571
H	2.363258	-0.613058	2.749799
H	3.722150	-1.748661	2.512065
H	2.150318	-2.088625	1.744088
C	2.674427	1.466010	-2.103911
H	3.503177	1.916137	-2.661359
H	2.020414	2.271048	-1.739732
H	2.107906	0.804601	-2.777023
C	5.806648	-0.817746	0.965299
C	6.959573	-0.438017	0.290710
C	6.911104	0.371406	-0.874981
C	5.708910	0.821821	-1.403899
H	5.855010	-1.435615	1.862221
H	7.928935	-0.772186	0.666964
H	7.844777	0.647211	-1.369769
H	5.681750	1.436249	-2.304074
C	1.058910	0.143515	0.073217
H	0.535032	-0.370171	0.877987
H	0.470321	0.689407	-0.662580

22_CH2_rad
28

N	-1.181095	0.108369	3.208223
C	-1.324643	0.880758	2.066608
C	-2.156854	2.118371	2.404446
C	-2.977065	2.751022	4.826541
H	-3.456486	3.686221	4.530098
C	-2.989492	2.339145	6.168654
H	-3.483888	2.961692	6.917290
C	-2.368271	1.137133	6.573041
H	-2.387503	0.850316	7.626553
C	-1.732842	0.313158	5.653235
H	-1.244734	-0.607859	5.973753
C	-1.736382	0.733038	4.309170
C	-2.340926	1.947022	3.894610
C	-0.546751	-1.202478	3.235391
H	-0.756673	-1.690854	4.192629
H	0.542454	-1.106025	3.105109
H	-0.956720	-1.823422	2.426967
C	-3.521106	2.066309	1.672445
H	-4.045346	1.117715	1.861047
H	-3.381550	2.184207	0.587641
H	-4.160407	2.888434	2.026110
C	-1.411050	3.426630	2.068340
H	-2.026629	4.289145	2.363269
H	-1.225559	3.500039	0.986476
H	-0.448609	3.489347	2.596438
C	-0.782251	0.552795	0.863349
H	-0.923372	1.213674	0.006907
H	-0.191655	-0.351184	0.708508

23_CH2_rad
28

C	-3.040654	1.286607	-0.904061
C	-2.164116	0.863742	0.179277
N	-2.899242	0.281071	1.141022
C	-4.351161	0.908755	-0.562774
C	-4.352380	0.215339	0.778666
C	-5.193667	0.975405	1.819072
C	-4.782328	-1.259935	0.660698
H	-4.148356	-1.800396	-0.056609
H	-5.819484	-1.302542	0.297522
H	-4.741675	-1.765880	1.636530
H	-4.826314	2.003143	1.951688
H	-5.196645	0.459693	2.790487
H	-6.233340	1.024171	1.465271
C	-5.408910	1.176220	-1.424769
C	-5.127586	1.828813	-2.632156

C	-3.816719	2.206729	-2.972405
C	-2.754598	1.938326	-2.111550
H	-1.738465	2.235971	-2.378890
H	-6.433243	0.886453	-1.179730
H	-5.943085	2.051706	-3.323639
H	-3.630485	2.717024	-3.919324
C	-0.764629	1.005665	0.220133
H	-0.250971	1.496944	-0.607029
H	-0.162911	0.628104	1.047429
C	-2.387306	-0.294316	2.372588
H	-3.177401	-0.281159	3.132692
H	-2.062878	-1.336561	2.214282
H	-1.540673	0.293639	2.748728

24_CH2_rad
20

O	3.296735	-0.490496	0.853361
N	3.211600	0.711603	-1.011775
C	2.464086	0.113718	-0.026257
C	4.574748	-0.289014	0.437760
C	4.545684	0.479424	-0.749710
C	2.653488	1.424801	-2.156737
H	3.468477	1.906250	-2.708568
H	1.953867	2.197861	-1.809887
H	2.131127	0.719576	-2.821038
C	5.750695	-0.737736	1.023782
C	6.925962	-0.370522	0.366845
C	6.913630	0.408160	-0.820326
C	5.730576	0.847193	-1.403688
H	5.745511	-1.331954	1.938069
H	7.884617	-0.693551	0.778949
H	7.863964	0.669629	-1.290590
H	5.730127	1.436655	-2.320786
C	1.101041	0.081945	0.114778
H	0.667440	-0.446665	0.964452
H	0.449947	0.573264	-0.607483

25_CH2_rad
16

S	3.154269	-0.723371	1.178878
S	3.043426	0.854737	-1.310258
C	2.170499	0.080707	-0.014760
C	4.684750	-0.327490	0.458210
C	4.630914	0.437234	-0.743691
C	5.921667	-0.714303	1.000280
C	7.082106	-0.334128	0.339948
C	7.028480	0.423306	-0.853314
C	5.814250	0.813204	-1.401357
H	5.966237	-1.293416	1.924491
H	8.050684	-0.626207	0.752008
H	7.956389	0.709115	-1.353594
H	5.775481	1.394443	-2.324655
C	0.802492	0.108837	0.058901
H	0.273936	-0.380117	0.881083
H	0.211441	0.623719	-0.702880

26_CH2_rad
20

S	3.137340	-0.758733	1.263890
N	3.190472	0.640653	-0.918725
C	2.305439	0.104673	-0.012586
C	4.677612	-0.380180	0.555112
C	4.518618	0.398538	-0.627643
C	2.720640	1.369332	-2.098601
H	3.578317	1.762144	-2.651754
H	2.085360	2.211731	-1.791019
H	2.151157	0.692451	-2.752760
C	5.945242	-0.749231	1.020422
C	7.052817	-0.323317	0.297273
C	6.902692	0.460633	-0.872062
C	5.651797	0.826990	-1.344902
H	6.058004	-1.347702	1.925917
H	8.053248	-0.598092	0.638862
H	7.791187	0.785738	-1.417221
H	5.559026	1.426527	-2.250286
C	0.934086	0.204438	-0.064225
H	0.326601	-0.263670	0.711793
H	0.423666	0.747216	-0.860336

27_CH2_rad
16

S	2.876887	-0.584947	1.440721
N	2.891742	0.510101	-0.907476
C	2.066540	0.062994	0.042693
C	4.496236	-0.520568	0.560560
C	4.311108	0.551135	-0.505688
C	2.507051	1.079411	-2.192266
H	2.722113	2.161154	-2.203827
H	1.442794	0.913108	-2.389202
H	3.092275	0.595133	-2.987665
H	4.690222	-1.515784	0.132272
H	5.286199	-0.272104	1.280719
H	4.526302	1.562700	-0.113729
H	4.949140	0.374987	-1.383070
C	0.655642	0.099128	0.005248
H	0.105874	0.548862	-0.822409
H	0.089454	-0.322487	0.838079

28_CH2_rad
12

S	2.813887	-0.726262	1.354809
S	3.196719	1.006863	-1.013703
C	2.128662	0.273582	0.125891
C	4.493270	-0.779755	0.601771
C	4.737632	0.539443	-0.118431
H	4.514422	-1.648532	-0.075177
H	5.216141	-0.942457	1.415115
H	4.972737	1.365431	0.570903
H	5.541182	0.453520	-0.864952
C	0.747553	0.477080	0.054103
H	0.313728	1.101397	-0.732292
H	0.077035	0.010941	0.781933

29_CH2_rad
14

C	-3.066466	0.951264	-0.935973
C	-2.288633	0.516895	0.108513
N	-2.884737	-0.438540	0.877697
S	-4.584340	0.145151	-0.963442
C	-4.149263	-0.790160	0.452515
C	-2.256507	-1.035185	2.062613
H	-2.149819	-2.120929	1.924911
H	-2.871351	-0.827814	2.950262
H	-1.264541	-0.589400	2.198264
H	-2.801122	1.700354	-1.681748
H	-1.284417	0.863930	0.352467
C	-4.964058	-1.736375	1.031528
H	-5.950144	-1.942344	0.611851
H	-4.649051	-2.298867	1.911795

30_CH2_rad
10

C	-2.999505	0.876040	-0.992143
C	-2.136440	0.537688	0.014008
S	-2.699562	-0.576445	1.190680
S	-4.553527	0.147564	-0.969856
C	-4.284750	-0.786328	0.484193
H	-2.757655	1.568254	-1.802869
H	-1.121043	0.929584	0.116055
C	-5.237843	-1.610555	1.025374
H	-6.219011	-1.717883	0.554741
H	-5.039225	-2.175248	1.940377

31_CH2_rad
27

C	2.854469	0.823867	-0.270956
N	2.108041	0.203301	-2.309903
C	1.673517	0.444384	-1.006976
C	3.954464	0.780944	-1.163497
C	3.457133	0.397098	-2.401660
C	1.238579	-0.177937	-3.410459
H	0.484464	0.601441	-3.597344
H	0.725826	-1.127337	-3.191618
H	1.846646	-0.303087	-4.314731
P	2.914107	1.366471	1.404862
C	2.324230	0.095838	2.557422
C	1.922516	2.870198	1.624559
C	4.630120	1.761893	1.827375
H	3.982532	0.246979	-3.342791
H	4.995266	1.007564	-0.944145
H	2.339310	3.658037	0.979646
H	1.945659	3.199663	2.674253
H	0.884004	2.680060	1.317168
H	1.293239	-0.192764	2.307930
H	2.359089	0.482691	3.587503
H	2.969321	-0.790491	2.470203
H	5.017634	2.518744	1.130608
H	5.248864	0.854973	1.767712
H	4.669308	2.163299	2.851093
C	0.356074	0.307230	-0.599158
H	-0.429469	-0.001387	-1.287802
H	0.063948	0.493588	0.433425

32_CH2_rad
17

N	2.885898	-0.576433	0.961842
C	2.886902	0.591443	-1.051215
C	2.060278	-0.078633	0.042435
C	4.318895	-0.333680	0.665974
C	4.347321	0.383698	-0.684609
C	2.520048	-1.308913	2.161079
H	2.717171	-2.383653	2.002871
H	1.461606	-1.157884	2.401961
H	3.143074	-0.959971	2.997479
O	2.407708	1.146545	-1.995003
H	4.836181	-1.306793	0.675687
H	4.732104	0.263696	1.495696
H	4.864769	1.354386	-0.652236
H	4.833822	-0.211545	-1.473765
C	0.670662	-0.121841	0.008024
H	0.181528	0.374232	-0.836011
H	0.059344	-0.617684	0.764422

33_CH2_rad
18

N	-2.932068	0.546474	-0.968082
C	-2.176450	-0.004841	0.017454
N	-2.945097	-0.542508	1.000351
C	-4.313419	0.387864	-0.659985
C	-4.322267	-0.358267	0.688107
C	-2.488214	-1.213782	2.217805
H	-1.940366	-0.512441	2.863796
H	-1.857501	-2.078730	1.967754
H	-3.385664	-1.564275	2.744146
C	-2.458405	1.212001	-2.182445
H	-1.916526	0.503502	-2.825670
H	-1.816911	2.067692	-1.927968
H	-3.347558	1.575696	-2.713932
O	-5.231503	0.761830	-1.317721
O	-5.248718	-0.714844	1.343722
C	-0.763604	-0.017157	0.020137
H	-0.205169	0.445511	-0.794604
H	-0.216419	-0.489684	0.836864

34_CH2_rad
27

N	-2.649087	-0.215260	1.128220
N	-4.243203	0.486140	-0.476325
C	-3.870710	-0.326805	0.547152
C	-2.295710	-0.974590	2.343825
H	-1.308786	-0.623232	2.660863
H	-2.244899	-2.050175	2.123441
H	-3.016002	-0.774148	3.147960
C	-5.629543	0.505120	-0.982684
H	-6.334698	0.723488	-0.169841
H	-5.877586	-0.450080	-1.466903
H	-5.684976	1.309472	-1.722926
C	-3.326381	1.429764	-1.073978
C	-1.860422	1.184876	-0.782609
C	-1.656487	0.716608	0.642415

C	-1.038014	2.443932	-1.062754
C	-1.415126	0.005310	-1.707167
O	-0.716325	0.992497	1.328994
O	-3.738848	2.277145	-1.811533
H	-1.998117	-0.912309	-1.536311
H	-0.353416	-0.214954	-1.526054
H	-1.538564	0.309052	-2.756678
H	-1.345661	3.269138	-0.405648
H	-1.184753	2.757677	-2.103991
H	0.025808	2.239857	-0.886864
C	-4.785105	-1.307725	1.033963
H	-5.766735	-1.431259	0.582288
H	-4.508133	-1.970167	1.850832

35_CH2_rad
28

N	2.498713	8.572606	0.902683
O	1.131354	8.705937	2.725578
C	3.695395	8.207518	0.342259
N	4.783562	8.069262	1.113230
C	2.220033	8.428997	2.302295
C	3.352411	7.842969	3.134482
C	4.681758	8.325681	2.554327
H	4.814459	9.411272	2.723946
H	5.514685	7.810131	3.049990
C	3.224757	8.320651	4.585908
H	3.354314	9.410823	4.666147
H	3.984146	7.831775	5.212849
H	2.231358	8.066537	4.978671
C	3.238690	6.305029	3.060319
H	3.385649	5.915413	2.040510
H	2.249554	5.980371	3.414629
H	3.997706	5.847689	3.711995
C	6.108216	7.766640	0.557277
C	1.387773	9.032631	0.051987
H	6.352161	8.445034	-0.270996
H	6.166446	6.722563	0.210722
H	6.851149	7.917271	1.348290
H	1.749561	9.775425	-0.670226
H	0.647941	9.494629	0.714104
H	0.906183	8.192339	-0.471516
C	3.772618	7.969800	-1.060523
H	2.879708	8.009225	-1.680535
H	4.713863	7.706788	-1.537816

36_CH2_rad
38

C	-1.958340	-4.007945	-8.696824
C	-2.460861	-5.213323	-8.103078
C	-1.582597	-2.950527	-7.924520
C	-2.558852	-5.314526	-6.681502
C	-1.687559	-3.007958	-6.494999
C	-2.191156	-4.190142	-5.869347
H	-1.878426	-3.957617	-9.784937
C	-2.864563	-6.299400	-8.907925
C	-3.027828	-6.545867	-6.103264
C	-2.308920	-4.221054	-4.436196
C	-3.438769	-7.593303	-6.950145
C	-3.361319	-7.464552	-8.338705
H	-2.792571	-6.205319	-9.993907
C	-3.079008	-6.583189	-4.669207
H	-3.852947	-8.510762	-6.541940
H	-3.696002	-8.286096	-8.974784
C	-1.292779	-1.911812	-5.699429
C	-1.875864	-3.111295	-3.685119
C	-1.373066	-1.970351	-4.313833
H	-1.193064	-2.037857	-8.381248
C	-2.818504	-5.440428	-3.876214
H	-1.898290	-3.129861	-2.599460
H	-1.038622	-1.126470	-3.707482
H	-0.905437	-1.016760	-6.191650
N	-3.091639	-5.782742	-2.572270
N	-3.395499	-7.608276	-3.808269
C	-3.421926	-7.131295	-2.503076
C	-3.171087	-4.888355	-1.425604
H	-3.807716	-5.344716	-0.659998
H	-2.177081	-4.690744	-0.997124
H	-3.635382	-3.943862	-1.734552
C	-3.557099	-9.018112	-4.137565
H	-3.329526	-9.621355	-3.251902
H	-4.582594	-9.237500	-4.471839
H	-2.844137	-9.292965	-4.923141
C	-3.702615	-7.850333	-1.373559
H	-3.645051	-7.394494	-0.387764
H	-3.995395	-8.896255	-1.431441

37_CH2_rad
24

C	-0.482868	-1.673565	-3.107039
C	-0.493921	-0.326694	-3.509333
C	-0.500846	-1.956114	-1.750514
C	-0.521756	0.740358	-2.570274
C	-0.532924	-0.874494	-0.790069
C	-0.541782	0.479419	-1.213835
C	-0.493844	-3.241128	-1.022067
C	-0.528199	-2.874605	0.408457
C	-0.551620	-1.432453	0.520584
H	-0.459756	-2.468183	-3.855292
H	-0.479699	-0.087514	-4.575800
H	-0.528131	1.769559	-2.934999
H	-0.564700	1.291960	-0.485203
C	-0.540165	-3.656095	1.552623
C	-0.587178	-0.799011	1.789028
C	-0.597613	-1.595198	2.918047
C	-0.574244	-3.012183	2.801473
H	-0.523358	-4.746336	1.497728
H	-0.606856	0.289643	1.868407
H	-0.625501	-1.141323	3.910620
H	-0.583918	-3.616377	3.711999
C	-0.460881	-4.478419	-1.548443
H	-0.435417	-4.640997	-2.628591
H	-0.456719	-5.368858	-0.915869

38_CH2_rad
26

C	6.150915	4.497675	12.044490

C	7.457324	4.588021	12.516445
C	7.747958	4.264164	13.909170
C	6.793088	4.503824	14.934057
C	8.996028	3.678254	14.263077
C	7.093313	4.212114	16.255331
C	9.281804	3.371280	15.586027
C	8.337854	3.646414	16.586791
H	5.837869	4.971308	14.689332
H	9.710387	3.423823	13.479091
H	6.366314	4.429692	17.040023
H	10.230759	2.898473	15.846418
H	8.566051	3.403774	17.627692
C	8.527583	5.009976	11.619706
C	9.635109	5.746116	12.128869
C	8.481479	4.724474	10.227438
C	10.656069	6.160945	11.285262
C	9.512355	5.128521	9.394177
C	10.602124	5.848233	9.917889
H	9.652296	6.031619	13.181671
H	7.654672	4.143722	9.814760
H	11.488706	6.744931	11.682506
H	9.481352	4.879856	8.331408
H	11.403041	6.177926	9.252121
H	5.330031	4.206099	12.702446
H	5.903042	4.719112	11.004340

39_CH2_rad
30

C	-1.122264	4.439250	10.360136
P	-2.473756	3.335066	10.393169
P	-0.622915	5.537295	9.097637
C	-0.246362	4.378149	11.511729
H	-0.223625	3.511562	12.175255
H	0.427781	5.197062	11.768687
C	0.913012	4.993942	8.273716
H	1.654136	4.761737	9.052480
H	0.715520	4.080322	7.694015
H	1.308142	5.776738	7.608722
C	-1.838518	5.855290	7.784376
H	-2.032790	4.954449	7.188973
H	-2.777335	6.235339	8.211017
H	-1.415326	6.624535	7.121289
C	-0.228282	7.177809	9.795421
H	-1.131446	7.610620	10.249546
H	0.547313	7.084026	10.568075
H	0.145791	7.837727	8.998372
C	-1.920138	1.625656	10.714947
H	-1.248802	1.303010	9.906447
H	-1.373991	1.578056	11.667525
H	-2.790952	0.954959	10.768164
C	-3.461376	3.226636	8.871854
H	-3.949748	4.181161	8.637691
H	-2.836208	2.902939	8.028265
H	-4.242231	2.470241	9.041234
C	-3.626208	3.725200	11.749830
H	-4.391396	2.941898	11.857933
H	-3.045617	3.801890	12.681173
H	-4.109451	4.694008	11.555469

40_CH2_rad
26

N	-4.662419	6.403202	0.659824
N	-6.031236	6.437379	0.717748
C	-4.270764	5.142563	0.361433
C	-3.830180	7.561724	0.927193
H	-2.794007	7.254502	0.746220
H	-3.935866	7.889940	1.972599
H	-4.084378	8.387137	0.248203
C	-6.779436	7.635303	1.052472
H	-6.566699	7.956850	2.083684
H	-7.841113	7.378348	0.964125
H	-6.547371	8.447905	0.350500
C	-6.508698	5.198916	0.454309
C	-5.420767	4.305974	0.193144
O	-7.816756	5.069403	0.483971
C	-8.477757	3.802798	0.340225
H	-9.533055	4.001163	0.559214
H	-8.080128	3.073472	1.059315
H	-8.377321	3.431178	-0.690076
O	-2.972447	4.946211	0.288654
C	-2.395658	3.652734	0.051891
H	-2.619568	3.312715	-0.969996
H	-2.755742	2.926471	0.793314
H	-1.314919	3.793192	0.166234
C	-5.472786	2.935987	-0.180276
H	-6.418066	2.419412	-0.308397
H	-4.571005	2.372891	-0.395719

41_CH2_rad
46

N	-2.298367	-1.608069	-0.148787
N	-2.453413	-1.523669	-2.358472
C	-1.567748	-1.611809	-1.310477
C	-3.657116	-1.534905	-0.458058
C	-3.758203	-1.504796	-1.861271
C	-1.799768	-1.835688	1.201685
H	-1.688755	-0.889117	1.751746
H	-0.825259	-2.333864	1.157703
H	-2.512393	-2.478280	1.736437
C	-2.113594	-1.270377	-3.747970
C	-0.136270	-1.642628	-1.412887
H	-1.236593	-4.153556	-1.458448
H	3.292816	-1.020276	-2.371654
C	0.815968	-3.950776	-4.082394
C	1.882240	-3.033320	-4.122437
C	-1.118101	-4.346752	-2.532301
N	-0.035480	-3.532549	-3.055752
N	1.622500	-2.065653	-3.150541
C	2.410338	-0.849364	-3.007048
C	0.449699	-2.368220	-2.503994
H	-0.861804	-5.405992	-2.671641
H	2.747236	-0.524653	-4.001557
H	-2.073931	-4.138545	-3.037393
H	1.794156	-0.063518	-2.556652
H	-2.897848	-0.646190	-4.196058
H	-2.020800	-2.200603	-4.328928
H	-1.160480	-0.728281	-3.796837

C	0.666296	-0.971309	-0.451979
H	0.235676	-0.221770	0.210438
H	1.723708	-1.206116	-0.338120
C	2.932262	-3.175879	-5.032296
H	3.767306	-2.474390	-5.063626
C	2.874351	-4.268970	-5.903829
C	0.752587	-5.037160	-4.957168
H	3.678774	-4.418764	-6.626839
H	-0.080095	-5.742142	-4.945022
C	1.804821	-5.181603	-5.867959
H	1.794268	-6.022676	-6.564081
C	-4.792337	-1.514000	0.353601
H	-4.719888	-1.526686	1.442039
C	-4.995126	-1.460120	-2.504041
C	-6.033101	-1.472495	-0.289391
H	-5.079358	-1.443581	-3.591670
H	-6.943705	-1.459560	0.312602
C	-6.133170	-1.445389	-1.691564
H	-7.119319	-1.413211	-2.158974

42_CH2_rad
18

C	2.994434	1.836005	-1.537426
C	1.777736	1.685531	-0.921669
C	4.151517	2.249131	-0.783709
H	0.903364	1.372286	-1.495760
C	1.648324	1.940008	0.481260
C	4.008467	2.493235	0.629589
H	4.881991	2.802488	1.208065
C	2.790506	2.345724	1.242556
H	2.683833	2.538818	2.311904
H	3.097726	1.639658	-2.607233
C	0.379528	1.788061	1.125770
C	0.217336	2.061268	2.507253
N	0.092267	2.294896	3.641735
C	-0.768057	1.357979	0.413529
N	-1.707826	0.999210	-0.174395
C	5.375902	2.415362	-1.404428
H	6.258674	2.730685	-0.839714
H	5.497338	2.240091	-2.478046

43_CH2_rad
16

C	2.978912	2.209055	-1.664990
C	1.811509	1.896110	-1.020065
C	4.111068	2.711861	-0.928113
H	0.952567	1.517669	-1.579098
C	1.699171	2.057172	0.407821
C	3.998733	2.872904	0.499792
H	4.857705	3.251228	1.058861
C	2.831256	2.560148	1.144680
H	2.744398	2.689966	2.225886
H	3.065656	2.079486	-2.746235
C	0.518677	1.729208	1.061937
C	5.291645	3.039611	-1.582173
H	6.154995	3.423101	-1.031082
H	5.389642	2.925966	-2.665817
H	0.420913	1.843137	2.145590

H	-0.344515	1.345379	0.510802

44_CH2_rad
68

C	-1.035328	1.033905	-0.418174
C	0.340668	1.098143	-0.377376
N	0.778650	0.033472	0.365895
C	-0.301076	-0.701540	0.794025
N	-1.416794	-0.070283	0.299166
C	-2.768646	-0.509957	0.546832
C	-3.415947	-0.031955	1.703215
C	-3.349281	-1.415754	-0.363607
C	-4.655262	-1.838378	-0.083753
C	-5.331700	-1.379377	1.047654
C	-4.721851	-0.487489	1.928903
C	-2.732969	0.889122	2.705904
H	-5.265855	-0.142792	2.810069
H	-5.150246	-2.537404	-0.759437
H	-6.347274	-1.728381	1.249046
C	-2.585497	-1.962029	-1.564444
C	-2.387826	0.120044	3.994501
C	-3.564914	2.144040	3.012626
H	-1.784705	1.234695	2.264637
H	-4.498931	1.894400	3.539784
H	-3.832876	2.688638	2.094097
H	-2.995949	2.826937	3.662391
H	-3.297823	-0.262293	4.483509
H	-1.870173	0.777133	4.709930
H	-1.732056	-0.739954	3.790832
C	-1.977116	-3.341662	-1.242401
H	-1.749647	-1.275481	-1.775200
C	-3.443819	-2.031671	-2.835208
H	-1.263992	-3.292369	-0.406040
H	-1.440232	-3.741103	-2.117366
H	-2.764319	-4.061466	-0.967771
C	-0.265403	-1.852552	1.561850
H	-2.824512	-2.344821	-3.690050
H	-3.899584	-1.058316	-3.074988
H	-4.256768	-2.767649	-2.738418
C	2.142018	-0.285416	0.712056

45_CH2_rad
50

C	-1.132152	0.937357	-0.151195
C	0.237037	1.089354	-0.145690
N	0.766912	0.033220	0.547596
C	-0.252407	-0.785767	0.980876
N	-1.423077	-0.209755	0.539035
C	-2.739648	-0.747563	0.776509
C	-3.401914	-0.395684	1.964242
C	-3.292066	-1.611681	-0.185013
C	-4.566766	-2.124436	0.073735
C	-5.275468	-1.797770	1.240226
C	-4.675360	-0.938007	2.171173
C	-2.760137	0.518174	2.976709
H	-5.213313	-0.680754	3.087107
H	-5.019406	-2.800583	-0.656249
C	-6.659809	-2.342935	1.471137

C	-2.535188	-1.971434	-1.437900
C	2.167113	-0.200956	0.799525
C	2.886288	-0.998069	-0.107344
C	2.742699	0.365581	1.949267
C	4.240433	-1.215401	0.167169
C	4.100563	0.115262	2.174267
C	4.866110	-0.666400	1.296516
C	6.333979	-0.890540	1.545926
H	4.572872	0.541883	3.062864
C	2.220055	-1.598446	-1.318804
H	4.823416	-1.832531	-0.521334
C	1.925817	1.202607	2.900131
H	-2.346364	-1.086537	-2.068227
H	-3.098650	-2.694963	-2.041798
H	-1.554242	-2.418930	-1.206335
H	-2.457622	1.478495	2.528322
H	-1.854119	0.064489	3.413318
H	-3.451571	0.736465	3.801285
H	1.058709	0.644659	3.291305
H	1.532882	2.109080	2.410408
H	2.530962	1.524218	3.758050

46_CH2_rad
52

C	-1.263107	1.173360	-0.059685
C	0.265851	1.395075	-0.025587
N	0.770669	0.161277	0.603578
C	-0.244080	-0.630065	0.982850
N	-1.434636	-0.105850	0.653447
C	-2.706105	-0.746851	0.818467
C	-3.558558	-0.313954	1.853737
C	-3.070325	-1.772628	-0.074091
C	-4.325305	-2.369398	0.096084
C	-5.203369	-1.970840	1.112150
C	-4.799356	-0.943986	1.979546
C	-3.152469	0.791958	2.794199
H	-5.471582	-0.626676	2.781509
H	-4.627191	-3.165186	-0.589667
C	-6.551331	-2.620665	1.280097
C	-2.140335	-2.229836	-1.170095
C	2.165109	-0.140169	0.745968
C	2.768652	-0.990083	-0.200494
C	2.886303	0.429106	1.812834
C	4.133085	-1.262693	-0.055175
C	4.247150	0.122692	1.912871
C	4.890470	-0.714526	0.989426
C	6.364787	-0.996154	1.109272
H	4.822274	0.547279	2.740157
C	1.968943	-1.600145	-1.324137
H	4.619094	-1.920478	-0.780401
C	2.222116	1.338652	2.814984
H	-1.657614	-1.385486	-1.686543
H	-2.682152	-2.818714	-1.922466
H	-1.332585	-2.871169	-0.776561
H	-3.295709	1.785263	2.334173
H	-2.093827	0.716235	3.087673
H	-3.760162	0.772096	3.709158
H	1.217471	0.984210	3.094205
H	2.110870	2.362551	2.417592
H	2.820607	1.414660	3.733071
H	1.349675	-0.851883	-1.844170
H	1.284877	-2.384973	-0.958043
H	2.629438	-2.065132	-2.068213
H	-6.720704	-3.407919	0.532199
H	-7.359868	-1.877769	1.183697
H	-6.647853	-3.072477	2.280828
H	6.952578	-0.156244	0.700662
H	6.652505	-1.901477	0.555675
H	6.665902	-1.120588	2.160507
H	0.555420	2.267064	0.580079
H	0.710278	1.512747	-1.024911
H	-1.664130	1.078938	-1.081065
H	-1.822093	1.969827	0.451503
C	-0.077989	-1.861343	1.674656
H	-0.957677	-2.431459	1.972416
H	0.926470	-2.208413	1.916045

47_CH2_rad
55

C	-0.996700	2.156303	-0.137231
C	-0.810056	0.736169	-0.693582
C	-1.570191	0.624929	-2.015074
C	0.673065	0.326171	-0.819634
H	0.797692	-0.310589	-1.707684
H	1.321676	1.201774	-0.955358
C	1.043675	-0.483043	0.451234
C	1.710795	0.380821	1.541520
C	-0.320370	-0.923187	0.944017
N	-1.305025	-0.280728	0.320362
C	-2.703638	-0.539881	0.579869
C	-3.363136	0.177321	1.601749
C	-3.333511	-1.550882	-0.181090
C	-4.699994	-1.759763	0.041768
C	-5.397702	-1.015145	0.992084
C	-4.731814	-0.073257	1.773077
C	-2.639164	1.089605	2.585234
H	-5.280786	0.467792	2.546000
H	-5.224481	-2.528103	-0.528686
H	-6.462747	-1.198763	1.151906
C	-2.566316	-2.478426	-1.117988
C	-2.562305	0.418482	3.969198
C	-3.263402	2.487667	2.689125
H	-1.605863	1.218874	2.237005
H	-4.280865	2.450931	3.108901
H	-3.322070	2.983389	1.708997
H	-2.660127	3.123539	3.354761
H	-3.566060	0.220201	4.376313
H	-2.031033	1.068386	4.681456
H	-2.027674	-0.543126	3.928554
C	-2.341865	-3.845778	-0.444173
H	-1.571144	-2.045049	-1.295515
C	-3.239241	-2.641525	-2.487952
H	-1.748382	-3.754116	0.479244
H	-1.804311	-4.526524	-1.122252
H	-3.298554	-4.319085	-0.172495
H	-1.145698	1.363096	-2.710930
H	-2.638661	0.851349	-1.890379
H	-1.465281	-0.364115	-2.475129

H	-0.595519	2.866179	-0.874430
H	-0.463502	2.315130	0.808179
H	-2.059942	2.386970	0.009348
H	2.670373	0.770234	1.168614
H	1.914350	-0.214657	2.444788
H	1.086142	1.236943	1.834448
C	1.934622	-1.688647	0.113861
H	2.858828	-1.336151	-0.369053
H	1.430077	-2.380415	-0.578042
H	2.225893	-2.248518	1.015965
C	-0.561527	-1.875040	1.941470
H	0.273931	-2.372250	2.434580
H	-1.578151	-2.130865	2.239250
H	-2.601532	-3.241426	-3.154696
H	-3.420419	-1.669335	-2.970145
H	-4.206053	-3.162097	-2.409364

48_CH2_rad
74

C	-1.193744	2.233510	0.013152
C	-0.895330	0.882906	-0.657855
C	-1.663116	0.838045	-1.982267
C	0.622356	0.623248	-0.818453
H	0.837642	0.249988	-1.821366
H	1.180917	1.560370	-0.703382
C	1.054965	-0.401212	0.273668
C	1.640818	0.424872	1.465000
C	-0.274804	-0.966321	0.720507
N	-1.303254	-0.263660	0.245401
C	-2.677311	-0.580514	0.573906
C	-3.256261	-0.005027	1.727028
C	-3.355102	-1.521044	-0.234748
C	-4.691634	-1.794044	0.081890
C	-5.311843	-1.190412	1.174796
C	-4.594715	-0.322638	1.996125
C	-2.467034	0.829753	2.730210
H	-5.077405	0.104688	2.876805
H	-5.247989	-2.509603	-0.526546
H	-6.351340	-1.429129	1.411642
C	-2.661500	-2.324676	-1.327676
C	-2.179382	0.009820	4.002446
C	-3.155543	2.159034	3.074602
H	-1.494531	1.072878	2.284466
H	-4.090013	2.000319	3.634952
H	-3.402467	2.738103	2.172313
H	-2.498210	2.775639	3.706465
H	-3.111397	-0.316680	4.490310
H	-1.611073	0.612650	4.727711
H	-1.588194	-0.892646	3.780993
C	-2.418380	-3.770836	-0.856133
H	-1.674170	-1.883133	-1.504580
C	-3.410760	-2.303944	-2.666344
H	-1.788732	-3.808963	0.045888
H	-1.909359	-4.348981	-1.642748
H	-3.364399	-4.280246	-0.615088
H	-1.297408	1.661379	-2.612915
H	-2.741896	0.982356	-1.826789
H	-1.507393	-0.096440	-2.531819
H	-0.899374	3.028509	-0.686901
H	-0.629172	2.379332	0.941996
H	-2.266087	2.347696	0.220549
H	2.411855	1.086888	1.036640
C	2.285062	-0.362652	2.601259
H	0.853421	1.089917	1.854348
C	2.071070	-1.494314	-0.233755
H	2.806787	-0.925759	-0.833136
C	1.445771	-2.590410	-1.161581
C	2.864113	-2.161014	0.916599
C	-0.484941	-2.062111	1.570566
H	-2.815887	-2.817522	-3.437702
H	-3.604352	-1.277152	-3.010641
H	-4.378677	-2.824687	-2.601322
C	3.394340	-1.220036	1.995255
C	2.798074	0.577955	3.692061
H	3.712130	-2.700601	0.475286
H	2.245913	-2.941841	1.394230
H	3.904438	-1.806568	2.776422
H	4.158022	-0.547121	1.564695
C	0.912201	-2.080226	-2.509471
H	0.591649	-3.032068	-0.613304
C	2.416028	-3.745462	-1.462963
H	0.057508	-1.402673	-2.421709
H	0.573450	-2.927302	-3.124477
H	1.701247	-1.558386	-3.076123
H	3.323044	-3.376916	-1.971220
H	1.928950	-4.464474	-2.139298
H	2.729715	-4.302833	-0.570961
H	1.530698	-1.030093	3.060011
H	3.539235	1.284387	3.282596
H	3.286740	0.014603	4.502006
H	1.979264	1.167822	4.135687
H	0.356091	-2.644989	1.934181
H	-1.493613	-2.353986	1.860292

49_CH2_rad
36

C	-0.156286	11.932175	4.450275
C	0.897271	11.115324	2.419209
C	0.115010	12.053761	1.617727
O	0.050049	11.954739	0.371678
C	-0.677353	13.032628	2.351716
C	1.866455	10.550364	4.620931
C	-1.007518	14.242020	4.480979
C	2.230534	10.310691	0.504238
H	2.424311	11.367453	0.300014
H	1.587057	9.912835	-0.293227
H	3.185300	9.762160	0.512909
C	1.600381	8.761140	2.317213
H	0.850687	8.635753	3.106203
H	2.585266	8.440293	2.691896
H	1.318190	8.104714	1.478998
C	-1.243285	14.368178	0.350028
H	-0.172331	14.351763	0.131039
H	-1.613154	15.404103	0.294527
H	-1.761754	13.755291	-0.401291
C	-2.858100	14.140959	2.201788
H	-3.563534	13.926973	1.383573
H	-2.995319	15.188325	2.514069

H	-3.101030	13.474550	3.036785
N	0.824555	11.160368	3.784059
N	-0.656301	13.034017	3.720343
N	1.611816	10.141752	1.825173
N	-1.502854	13.878128	1.709142
H	2.762167	10.395664	4.010077
H	1.551687	9.590719	5.056490
H	2.103894	11.246912	5.435177
H	-0.937856	15.107752	3.812565
H	-0.280812	14.358011	5.295872
H	-2.018130	14.198607	4.912152
C	-0.515791	11.692586	5.726023
H	-0.092252	10.863627	6.288985
H	-1.245069	12.319516	6.234770

50_CH2_rad
37

P	1.046830	-1.443846	-0.382998
C	1.054091	-0.040492	-1.475065
H	1.952528	0.571739	-1.584324
H	0.189567	0.164196	-2.112578
C	1.364341	-2.950696	-1.331111
C	2.435240	-3.797443	-1.002595
C	0.499098	-3.269903	-2.395166
C	2.638333	-4.964192	-1.744505
C	0.713203	-4.436376	-3.128420
C	1.781748	-5.281848	-2.802969
H	3.103359	-3.552984	-0.175200
H	-0.339772	-2.616540	-2.648355
H	3.466475	-5.628652	-1.489292
H	0.044865	-4.690541	-3.953957
H	1.947960	-6.194055	-3.380302
C	-0.562660	-1.566687	0.411473
C	-1.251863	-0.391555	0.758806
C	-1.061134	-2.825489	0.787111
C	-2.441757	-0.481711	1.481619
C	-2.250439	-2.902565	1.514473
C	-2.936056	-1.733957	1.863042
H	-0.859739	0.587407	0.470828
H	-0.523659	-3.736029	0.513681
H	-2.983635	0.426603	1.753885
H	-2.645850	-3.876231	1.812768
H	-3.865899	-1.799983	2.432551
C	2.331075	-1.223286	0.860349
C	2.019779	-1.390595	2.218852
C	3.646682	-0.923004	0.460265
C	3.029235	-1.265846	3.175309
C	4.646947	-0.806167	1.424352
C	4.338326	-0.979642	2.779160
H	0.998956	-1.618930	2.530525
H	3.898109	-0.791092	-0.595716
H	2.788101	-1.394648	4.232829
H	5.669199	-0.574354	1.117440
H	5.125338	-0.885135	3.530430

51_CH2_rad
14

C	-0.581991	-0.000064	-4.081243
C	-0.539595	1.207894	-3.369961
C	-0.453145	1.193847	-1.993953
N	-0.406254	0.000962	-1.301141
C	-0.451648	-1.192433	-1.993170
C	-0.538083	-1.207500	-3.369168
H	-0.651676	-0.000476	-5.171096
H	-0.575148	2.169693	-3.884875
H	-0.416577	2.099477	-1.387582
H	-0.413883	-2.097621	-1.386213
H	-0.572375	-2.169680	-3.883457
C	-0.317458	0.001472	0.068703
H	-0.280326	-0.955418	0.587497
H	-0.281558	0.958754	0.586864

CH_3 Adducts
01_CH3
31

C	-2.888934	0.430460	-1.222555
C	-2.036739	-0.089118	-0.089550
N	-2.744825	-0.520507	0.905921
C	-4.308219	-0.035569	-0.806719
C	-4.241667	-0.426199	0.687897
C	-2.210963	-1.049382	2.159409
H	-1.205854	-0.655331	2.347904
H	-2.170737	-2.148299	2.117552
H	-2.869376	-0.750576	2.983804
C	-2.759944	1.971741	-1.254362
H	-1.734265	2.289308	-1.496025
H	-3.055708	2.432736	-0.300377
H	-3.421958	2.364458	-2.039769
C	-4.800822	0.662322	1.609615
C	-4.879975	-1.788569	0.961712
H	-4.605403	-0.907569	-1.406168
H	-5.060326	0.745123	-0.980334
C	-2.461340	-0.152648	-2.580629
H	-2.455377	-1.253261	-2.572528
H	-1.466721	0.203571	-2.887754
H	-3.178434	0.175456	-3.347974
H	-4.397862	-2.579739	0.367570
H	-5.940857	-1.749218	0.672011
H	-4.837370	-2.064630	2.025846
H	-4.305976	1.629499	1.437690
H	-4.698646	0.398387	2.673094
H	-5.874459	0.786608	1.406468
C	-0.550083	-0.075968	-0.084493
H	-0.154195	0.351183	-1.012098
H	-0.157542	-1.100334	0.025452
H	-0.165161	0.513605	0.763396

02_CH3
19

N	3.050230	-0.407859	0.992249
N	3.021451	0.500660	-0.984460
C	2.240091	0.013460	0.000455
C	4.367661	-0.182138	0.627899
C	4.349499	0.386406	-0.612109
C	2.616933	-0.994833	2.264577
H	1.935883	-1.836798	2.083729

H	2.117957	-0.237033	2.885515
H	3.501539	-1.367839	2.794210
C	2.523929	1.062826	-2.241801
H	1.842937	1.900784	-2.036899
H	1.994981	0.292628	-2.821489
H	3.375715	1.432960	-2.824403
H	5.199785	-0.446888	1.274880
H	5.162312	0.716095	-1.253719
C	0.758990	-0.055163	-0.041068
H	0.328362	0.932131	-0.271485
H	0.348998	-0.396009	0.916934
H	0.425133	-0.755340	-0.824273

03_CH3
21

N	2.875253	0.294146	1.028694
N	2.905042	-0.159308	-1.127011
C	2.194538	0.332497	-0.114435
C	4.220389	-0.275210	0.824438
C	4.242535	-0.588000	-0.685446
C	2.485392	0.748714	2.353210
H	2.556797	-0.085950	3.068253
H	1.457542	1.129163	2.354728
H	3.158826	1.554171	2.686070
C	2.515027	-0.280777	-2.520715
H	1.457026	-0.025602	-2.652825
H	2.674500	-1.317125	-2.857994
H	3.123695	0.387222	-3.150810
H	4.346147	-1.171321	1.452085
H	4.982814	0.459388	1.125665
H	5.015171	-0.025309	-1.231372
H	4.384855	-1.658308	-0.903655
C	0.801616	0.842842	-0.274412
H	0.764498	1.614874	-1.057420
H	0.410863	1.274473	0.652547
H	0.135960	0.020567	-0.581252

04_CH3
13

O	-6.443654	1.837007	0.292518
C	-5.531722	2.735344	0.362322
O	-4.327489	2.316061	0.222353
C	-4.327740	0.852157	0.065101
C	-5.831392	0.526354	0.016620
H	-3.800494	0.438850	0.934419
H	-3.778470	0.624015	-0.856357
H	-6.177217	-0.163193	0.795974
H	-6.199700	0.202976	-0.965920
C	-5.839217	4.151719	0.612423
H	-5.145794	4.787495	0.044428
H	-5.671991	4.353092	1.686690
H	-6.886058	4.366444	0.363260

05_CH3
17

C	-2.919447	0.089251	-1.482001
C	-1.944417	0.029078	-0.292114
N	-2.832297	-0.438992	0.791399
O	-4.228299	-0.150001	-0.871463
C	-4.079162	-0.469904	0.382332
C	-2.329879	-0.702341	2.136259
H	-1.950672	0.230627	2.581854
H	-1.508394	-1.431088	2.082240
H	-3.128820	-1.109005	2.767162
H	-2.965676	1.062462	-1.985443
H	-1.519426	1.008705	-0.020459
C	-5.279640	-0.804169	1.187070
H	-6.158705	-0.826208	0.531484
H	-5.432199	-0.045934	1.971909
H	-5.160030	-1.782429	1.678665
H	-2.761389	-0.704999	-2.224119
H	-1.119839	-0.681990	-0.442652

06_CH3
15

C	-6.624806	1.986326	0.047828
C	-5.722581	2.895524	0.805126
O	-4.544763	2.444038	0.911931
C	-4.385229	1.121178	0.233582
C	-5.823485	0.671998	-0.032745
H	-3.803153	0.500558	0.925066
H	-3.787568	1.336884	-0.663693
H	-6.161768	-0.033680	0.738790
H	-5.923048	0.177087	-1.007632
H	-7.617441	1.910713	0.518670
H	-6.795017	2.459017	-0.940696
C	-6.053432	4.205047	1.384463
H	-6.788661	4.050741	2.196206
H	-6.566815	4.822026	0.626782
H	-5.164165	4.714630	1.776195

07_CH3
23

C	-0.642472	2.568674	0.062271
N	-1.860407	2.118354	-0.261419
C	-2.156506	0.697964	-0.491851
C	-3.057679	2.970027	-0.207697
N	0.466333	1.920494	-0.312374
C	1.761341	2.168564	0.332564
C	0.559340	1.109060	-1.532462
H	1.638660	2.504106	1.367663
H	2.346098	2.915604	-0.228798
H	2.323930	1.224374	0.346415
H	0.643076	0.034138	-1.311132
H	1.464263	1.424163	-2.073319
H	-0.302339	1.293227	-2.182294
H	-3.787402	2.574749	-0.926503
H	-2.827027	4.000543	-0.501058
H	-3.515311	2.959485	0.794971
H	-2.348918	0.478781	-1.552993
H	-3.060735	0.448630	0.082450
H	-1.339442	0.069242	-0.124283
C	-0.500391	3.806600	0.900291
H	-1.410182	4.412496	0.895940
H	0.340629	4.426251	0.563147

H	-0.316518	3.512826	1.948420

08_CH3
25

N	3.134746	-1.112665	0.153109
N	2.787812	0.891375	-0.596644
C	2.293252	-0.362561	-0.585529
C	4.188235	-0.327425	0.622763
C	3.967078	0.942899	0.151851
C	2.996782	-2.538517	0.433668
H	2.083352	-2.924891	-0.029478
H	2.944766	-2.703767	1.518952
H	3.859515	-3.085084	0.027766
C	2.230132	2.056073	-1.280663
H	1.297421	1.788542	-1.786956
H	2.945092	2.426936	-2.028220
H	2.023810	2.851624	-0.551885
C	5.289315	-0.892685	1.451825
C	4.744255	2.201656	0.326500
C	1.059021	-0.860849	-1.253167
H	0.516488	-0.048592	-1.749816
H	0.377269	-1.322042	-0.521203
H	1.300499	-1.617807	-2.016555
H	5.081508	2.611298	-0.639894
H	5.636001	2.014432	0.937599
H	4.149492	2.980153	0.830937
H	5.895674	-1.614565	0.879583
H	4.900868	-1.411962	2.342360
H	5.957901	-0.093920	1.797279

09_CH3
21

N	2.961442	-0.419202	0.883643
N	2.999091	0.528143	-0.968017
N	2.182609	0.073443	-0.048534
C	4.292859	-0.297093	0.584035
C	4.318354	0.340466	-0.647645
C	2.374667	-1.010543	2.084310
H	2.571676	-2.092110	2.100244
H	1.295270	-0.822717	2.055827
H	2.816361	-0.538189	2.971912
C	2.457209	1.140541	-2.179365
H	1.365340	1.058887	-2.134141
H	2.838865	0.604863	-3.059099
H	2.757767	2.196951	-2.227019
C	5.388157	-0.791776	1.457938
H	6.362642	-0.540343	1.020489
H	5.339111	-1.886466	1.576469
H	5.338936	-0.337372	2.459893
C	5.451989	0.786774	-1.498389
H	5.511082	1.887060	-1.534253
H	5.352020	0.414469	-2.529858
H	6.399618	0.408967	-1.093929

10_CH3
18

C	-0.613688	2.580345	0.098218
C	-1.948366	1.931017	-0.098274
H	-2.110876	1.166084	0.682929
H	-2.038917	1.434515	-1.075135
H	-2.747420	2.676199	0.006541
N	0.475703	1.977029	-0.288435
C	1.822480	2.570635	-0.242262
C	0.456069	0.615902	-0.859408
H	1.790388	3.630665	0.019129
H	2.277269	2.462674	-1.237966
H	2.437210	2.023562	0.487582
H	1.431298	0.150111	-0.671536
H	0.287866	0.665197	-1.947048
H	-0.321658	0.004303	-0.391138
C	-0.610498	3.929759	0.735038
H	0.340627	4.223480	1.191011
H	-1.400094	3.967622	1.499974
H	-0.888018	4.679379	-0.029286

11_CH3
15

C	-3.007702	0.446885	-1.105426
C	-2.339907	0.493030	0.068718
N	-3.025038	-0.365517	0.933986
O	-4.063370	-0.414502	-0.955217
C	-4.053554	-0.890995	0.274373
C	-2.636303	-0.654236	2.319515
H	-2.326878	0.280927	2.803209
H	-1.801381	-1.369565	2.328966
H	-3.491812	-1.075675	2.859746
H	-2.879964	0.920950	-2.074924
H	-1.455019	1.046324	0.373944
C	-5.082095	-1.843983	0.742394
H	-5.654759	-2.209826	-0.120180
H	-5.777731	-1.346075	1.439236
H	-4.620479	-2.697682	1.261230

12_CH3
21

N	9.085618	0.052757	5.110372
N	8.514476	-0.907721	3.282725
C	8.019766	-0.323529	4.400409
N	10.245614	-0.266368	4.483486
C	9.897283	-0.849768	3.365892
C	9.160474	0.709308	6.412176
H	9.811376	1.588131	6.320264
H	9.591882	0.009217	7.140739
H	8.159681	1.017466	6.733442
C	7.713942	-1.476338	2.199357
H	7.214043	-0.679182	1.629337
H	6.962181	-2.162794	2.611731
H	8.373744	-2.037250	1.527838
C	10.837868	-1.354561	2.334807
H	10.741754	-2.443737	2.198104
H	11.861314	-1.131991	2.662216
H	10.661024	-0.865148	1.364126
C	6.579793	-0.151474	4.710251
H	6.095872	-1.130236	4.862655
H	6.432623	0.449430	5.614716

H	6.068106	0.351087	3.874432

13_CH3
19

N	3.059557	-0.654907	0.885380
N	3.851937	-0.999012	-1.117465
C	3.557367	-1.596526	0.056851
C	3.025821	0.565807	0.223430
C	3.524233	0.351689	-1.036572
C	2.611890	-0.854261	2.265014
H	1.528199	-0.682006	2.334857
H	3.131960	-0.145225	2.922276
H	2.845565	-1.877661	2.576813
C	4.410021	-1.650545	-2.306392
H	5.188296	-2.363189	-2.009248
H	4.858981	-0.878429	-2.941246
H	3.616539	-2.165433	-2.867085
Cl	3.748603	1.454390	-2.301845
Cl	2.455087	1.988820	0.940416
C	3.731496	-3.035942	0.385014
H	4.142704	-3.585819	-0.469000
H	4.417579	-3.169301	1.236305
H	2.765828	-3.495806	0.650053

14_CH3
21

N	2.910096	-0.668741	0.818284
N	3.583426	-0.840159	-1.252851
C	2.981869	-1.499023	-0.241666
C	3.463826	0.558842	0.471510
C	3.894403	0.447715	-0.837821
C	2.384507	-1.003299	2.150849
H	2.483667	-0.116954	2.789030
H	2.970182	-1.824419	2.585344
H	1.324999	-1.285568	2.083092
C	3.884526	-1.406904	-2.574718
H	4.637701	-2.201464	-2.476548
H	4.282590	-0.609188	-3.213274
H	2.968159	-1.812351	-3.024250
C	2.478402	-2.892031	-0.318850
H	3.274103	-3.574258	-0.656637
H	2.108586	-3.238047	0.653624
H	1.649844	-2.960550	-1.043994
C	3.520565	1.671719	1.346194
N	3.549637	2.582322	2.064666
C	4.539110	1.399412	-1.664864
N	5.078068	2.158010	-2.357537

15_CH3
25

N	2.881562	-0.980182	0.652423
C	3.035606	0.954428	-0.429512
C	2.136547	0.025028	0.031434
C	4.194579	-0.687413	0.576406
N	4.302156	0.483814	-0.080356
C	2.830416	2.255873	-1.124268
H	3.148451	3.100362	-0.490170
H	1.768054	2.399757	-1.359465
H	3.392669	2.313806	-2.069452
C	2.270204	-2.146482	1.283568
H	1.735321	-2.743407	0.530767
H	1.560310	-1.818471	2.055074
H	3.039237	-2.768178	1.752587
C	5.553603	1.149370	-0.426234
C	5.338356	-1.476243	1.108446
H	5.739562	1.066020	-1.507468
H	6.383725	0.685864	0.116285
H	5.498909	2.209959	-0.145283
H	4.995201	-2.351082	1.671195
H	5.952829	-0.862723	1.785762
H	5.985761	-1.832730	0.291132
C	0.650945	-0.025177	-0.045880
H	0.267022	0.852719	-0.579623
H	0.190109	-0.038080	0.955203
H	0.303594	-0.920601	-0.585995

16_CH3
19

N	-2.951769	0.413301	-0.963064
C	-2.156088	0.009307	0.059531
C	-2.991152	-0.481610	1.069965
N	-4.253717	0.201874	-0.625258
C	-4.293448	-0.343490	0.604112
C	-2.575856	1.003586	-2.240136
H	-3.080232	0.481558	-3.063854
H	-1.493709	0.882378	-2.362102
H	-2.825479	2.075586	-2.266489
C	-0.672082	0.119850	0.033295
H	-0.349717	1.173612	0.005252
H	-0.237657	-0.395827	-0.837222
H	-0.255977	-0.337974	0.939181
C	-5.361887	0.521678	-1.514478
H	-5.310931	-0.079163	-2.433521
H	-6.288517	0.277940	-0.980947
H	-5.363366	1.592103	-1.765920
H	-5.245131	-0.602589	1.064398
H	-2.673524	-0.891172	2.025382

17_CH3
25

N	8.917291	4.628039	14.474801
N	6.052365	4.416443	12.094648
C	8.527999	5.195593	11.967560
C	8.389089	4.793651	13.280253
C	7.303467	4.710007	12.379013
C	10.279199	5.094835	14.750682
C	8.177461	4.026647	15.582934
C	5.092543	3.977017	13.104555
C	5.555888	4.547479	10.723083
H	10.737054	5.479210	13.830639
H	10.891968	4.266239	15.138634
H	10.262616	5.901106	15.501585
H	7.299710	3.491377	15.201781
H	7.856263	4.791770	16.308391
H	8.823168	3.301967	16.101643

H	4.205500	4.628390	13.080468
H	5.542449	4.040826	14.102348
H	4.768511	2.940826	12.911897
H	4.743877	5.289991	10.679541
H	5.164792	3.579273	10.371338
H	6.368275	4.866508	10.058703
C	9.377129	5.746972	10.889131
H	9.891501	4.931190	10.352319
H	10.147364	6.414802	11.300675
H	8.773514	6.304318	10.158459

18_CH3
24

C	7.888798	5.196714	0.659176
C	6.389087	4.975342	0.629577
C	8.262328	6.241571	-0.376013
H	8.420478	4.256457	0.439966
H	8.199121	5.527730	1.660610
N	5.904124	4.796594	-0.752068
H	5.849612	5.823045	1.084949
H	6.113620	4.071400	1.194033
C	6.630019	5.137092	-1.820257
N	7.766942	5.827828	-1.699606
H	9.351688	6.358149	-0.445199
H	7.836439	7.228148	-0.120349
C	8.535015	6.259710	-2.874054
C	4.549749	4.240834	-0.857439
H	9.033095	5.410992	-3.368959
H	9.305999	6.961319	-2.536558
H	7.897986	6.781672	-3.601332
H	4.563253	3.140515	-0.920008
H	4.012846	4.655577	-1.717663
H	3.996489	4.530651	0.046135
C	6.163834	4.754145	-3.196767
H	7.015105	4.440988	-3.814799
H	5.688252	5.615462	-3.695628
H	5.445817	3.928976	-3.171608

19_CH3
32

C	-0.458559	0.030981	0.025752
C	1.091545	0.185699	-0.023720
C	1.344511	1.652833	-0.275163
N	0.773329	2.139754	-1.345469
C	0.002693	1.147509	-2.194905
C	-1.088221	0.551325	-1.277319
C	1.024198	0.050131	-2.560142
C	1.608685	-0.574500	-1.282965
C	0.846609	3.540185	-1.773587
C	-0.600073	1.776573	-3.444350
C	1.733304	-0.359025	1.251108
H	0.506884	-0.698438	-3.177443
H	1.809308	0.496482	-3.190071
H	1.306211	-1.626397	-1.172394
H	0.159954	2.219057	-4.103820
H	-1.100475	0.979171	-4.012114
H	-1.361941	2.533644	-3.211979
H	-1.853040	1.316558	-1.079637
H	-1.583471	-0.255005	-1.837833
H	-0.845754	0.573437	0.901308
H	-0.680315	-1.033537	0.191045
H	1.367752	0.156821	2.151346
H	1.477776	-1.423630	1.354939
H	2.830955	-0.289488	1.229177
H	2.708395	-0.559961	-1.293974
H	1.449607	3.620175	-2.688483
H	-0.164478	3.906298	-1.987125
H	1.286684	4.165311	-0.993978
C	2.177658	2.446782	0.667976
H	1.640085	2.530831	1.628061
H	3.099592	1.889258	0.889365
H	2.448577	3.448798	0.323141

20_CH3
25

N	-2.961212	0.813765	-1.006810
C	-2.062182	0.320093	-0.112744
N	-2.787799	-0.248089	0.891039
C	-4.275816	0.563732	-0.585180
C	-4.164707	-0.121398	0.637292
C	-2.338611	-0.884223	2.038392
C	-5.358558	0.995001	-1.378097
C	-5.104512	1.660832	-2.555484
C	-3.757840	1.900655	-2.956985
C	-2.705133	1.476945	-2.195864
H	-1.659523	1.627778	-2.460929
H	-6.375028	0.786743	-1.040215
H	-5.925561	2.006569	-3.184504
H	-3.545816	2.426544	-3.889311
C	-5.100322	-0.650163	1.549585
C	-3.248751	-1.397983	2.917989
C	-4.650519	-1.288019	2.682164
H	-1.261171	-0.941480	2.182153
H	-2.876324	-1.897411	3.813948
H	-6.163586	-0.539142	1.330628
H	-5.356037	-1.707197	3.400239
C	-0.590074	0.372529	-0.264536
H	-0.261738	-0.190818	-1.154965
H	-0.085428	-0.063565	0.605464
H	-0.235383	1.411413	-0.370755

21_CH3
25

N	3.252008	-0.848149	0.560337
N	3.200880	0.923439	-0.735614
C	2.442433	0.022757	-0.077135
C	4.581899	-0.504669	0.311155
C	4.549623	0.620850	-0.528718
C	2.842330	-1.972491	1.391772
H	3.031651	-1.748728	2.452636
H	3.415184	-2.863236	1.099064
H	1.775931	-2.177692	1.246607
C	2.748399	2.063340	-1.524956
H	2.888092	1.864980	-2.598145
H	3.334725	2.946504	-1.237109
H	1.692177	2.269316	-1.321799

C	5.784965	-1.069161	0.742643
C	6.953509	-0.465281	0.279991
C	6.921321	0.655227	-0.578374
C	5.719509	1.222471	-1.000025
H	5.814668	-1.936010	1.403896
H	7.917627	-0.872171	0.592199
H	7.860983	1.091373	-0.923596
H	5.699777	2.088884	-1.662138
C	0.958276	-0.025196	-0.042929
H	0.518049	0.699242	-0.735543
H	0.581827	0.191289	0.970143
H	0.597079	-1.023657	-0.334886

22_CH3
29

N	-1.165041	0.147092	3.164036
C	-0.953706	1.115428	2.306668
C	-1.918444	2.259016	2.547464
C	-3.780173	2.275944	4.434036
H	-4.188382	3.257255	4.182714
C	-4.312032	1.536165	5.500576
H	-5.138051	1.950366	6.082344
C	-3.798802	0.275344	5.836385
H	-4.231371	-0.278538	6.671794
C	-2.740363	-0.290166	5.113519
H	-2.341853	-1.270516	5.378068
C	-2.236409	0.466894	4.061295
C	-2.725168	1.728626	3.713463
C	-0.441171	-1.118891	3.259183
H	0.367026	-1.154884	2.522403
H	-1.142313	-1.946824	3.080273
H	-0.018551	-1.216191	4.269388
C	-2.788242	2.511208	1.293192
H	-3.315549	1.600314	0.973313
H	-2.182238	2.890193	0.456887
H	-3.542073	3.274360	1.533493
C	-1.127108	3.536724	2.927150
H	-1.842065	4.334282	3.175763
H	-0.507972	3.883028	2.086564
H	-0.483815	3.370878	3.803541
C	0.107077	1.104400	1.270446
H	1.100497	1.185677	1.746878
H	0.094045	0.175329	0.679475
H	-0.011418	1.953928	0.587489

23_CH3
29

C	-3.036206	1.255456	-0.919752
C	-2.176069	0.872916	0.177190
N	-2.890154	0.315602	1.133079
C	-4.351381	0.883824	-0.578044
C	-4.352851	0.233248	0.783601
C	-5.182968	1.033638	1.798482
C	-4.782487	-1.241015	0.715311
H	-4.151255	-1.805816	0.014272
H	-5.821107	-1.298827	0.358662
H	-4.737674	-1.715200	1.706971
H	-4.812472	2.065065	1.888508
H	-5.176558	0.556098	2.789372
H	-6.226720	1.072037	1.455640
C	-5.401751	1.125415	-1.454511
C	-5.109125	1.743254	-2.678412
C	-3.796453	2.112721	-3.021240
C	-2.740554	1.872411	-2.144838
H	-1.722299	2.163307	-2.410951
H	-6.428291	0.843002	-1.210150
H	-5.919680	1.945809	-3.382108
H	-3.605036	2.595652	-3.981374
C	-2.363550	-0.222430	2.379063
H	-3.117070	-0.114327	3.168723
H	-2.112780	-1.289906	2.268038
H	-1.463321	0.327168	2.680292
C	-0.702931	1.052808	0.249593
H	-0.200354	0.084686	0.405979
H	-0.315685	1.506183	-0.669832
H	-0.432049	1.704598	1.096207

24_CH3
21

O	3.308520	-0.402133	0.985861
N	3.195760	0.661994	-0.915769
C	2.501701	0.143330	0.093525
C	4.611303	-0.238316	0.548884
C	4.564631	0.453122	-0.662267
C	5.788419	-0.667593	1.142824
C	6.956242	-0.340268	0.447105
C	6.925606	0.377129	-0.767140
C	5.727434	0.789800	-1.355008
H	5.795898	-1.217882	2.084116
H	7.918561	-0.650616	0.859602
H	7.866628	0.617966	-1.265841
H	5.709743	1.339476	-2.296700
C	1.039188	0.130771	0.306147
H	0.663077	1.155531	0.463129
H	0.812478	-0.470846	1.194783
H	0.514611	-0.299217	-0.561035
C	2.660088	1.299599	-2.116608
H	2.981006	2.350871	-2.151317
H	1.565518	1.248121	-2.106590
H	3.042069	0.769312	-3.000344

25_CH3
17

S	3.137055	-0.690365	1.160997
S	3.014709	0.999611	-1.172359
C	2.188819	0.156681	0.042241
C	4.692084	-0.272705	0.473220
C	4.631995	0.558799	-0.667553
C	5.925234	-0.698741	0.985815
C	7.083863	-0.278943	0.339081
C	7.023670	0.551590	-0.796836
C	5.803928	0.980438	-1.311140
H	5.974897	-1.337644	1.869645
H	8.053603	-0.602801	0.723485
H	7.947279	0.867799	-1.286457
H	5.758591	1.621388	-2.193881

C	0.698892	0.130279	0.102884
H	0.271307	1.060484	-0.298216
H	0.350278	-0.012082	1.135886
H	0.314577	-0.707992	-0.503776

26_CH3
21

S	3.177576	-0.716094	1.405381
N	3.181628	0.599444	-0.775075
C	2.354907	0.109716	0.149582
C	4.727572	-0.385662	0.658586
C	4.542151	0.364599	-0.518454
C	6.004467	-0.759031	1.088931
C	7.090143	-0.342533	0.322197
C	6.907510	0.430827	-0.841050
C	5.636615	0.792397	-1.279886
H	6.146377	-1.351844	1.993983
H	8.099138	-0.620622	0.634284
H	7.778312	0.755696	-1.413844
H	5.506781	1.390322	-2.182466
C	0.873013	0.244874	0.140417
H	0.578974	1.307144	0.166905
H	0.438459	-0.253550	1.015950
H	0.437263	-0.212216	-0.762720
C	2.745571	1.296680	-1.988294
H	3.032784	2.357276	-1.933614
H	1.659695	1.212960	-2.096517
H	3.229543	0.829772	-2.856945

27_CH3
17

C	-3.013152	0.896738	-1.101235
C	-2.433070	0.655824	0.290309
N	-2.980143	-0.636072	0.761413
S	-4.633916	0.013855	-1.092855
C	-4.105433	-1.002838	0.192823
C	-2.326377	-1.312995	1.879729
H	-1.321081	-1.641516	1.572778
H	-2.905755	-2.185248	2.198310
H	-2.233238	-0.611781	2.723208
C	-4.894767	-2.203418	0.587380
H	-5.839037	-2.252057	0.031604
H	-5.113326	-2.197808	1.666558
H	-4.313728	-3.113502	0.358868
H	-1.334968	0.608213	0.276681
H	-2.740042	1.433630	1.010661
H	-3.176723	1.959539	-1.319436
H	-2.403415	0.449754	-1.900038

28_CH3
13

C	-3.110948	1.019921	-0.789205
C	-2.494729	0.664224	0.557081
S	-2.594023	-1.165290	0.780524
S	-4.561139	-0.081673	-1.083182
C	-3.968129	-1.388921	-0.181008
H	-2.418832	0.855694	-1.629294
H	-1.434653	0.953807	0.608667
C	-4.635612	-2.719200	-0.252588
H	-5.728517	-2.600603	-0.317830
H	-4.382220	-3.349599	0.609915
H	-4.305390	-3.232529	-1.173869
H	-3.470117	2.059576	-0.814803
H	-3.029649	1.112448	1.408065

29_CH3
15

C	-3.098087	0.958223	-0.935563
C	-2.426665	0.556732	0.175127
N	-2.964434	-0.590804	0.740852
S	-4.413691	-0.114178	-1.267539
C	-4.028403	-1.079119	0.089310
C	-2.424698	-1.195684	1.970540
H	-2.326812	-2.281282	1.842170
H	-3.094636	-0.977416	2.814969
H	-1.435898	-0.767396	2.170450
H	-2.884784	1.814738	-1.573740
H	-1.556085	1.026276	0.630673
C	-4.765584	-2.312401	0.473894
H	-5.667265	-2.436113	-0.139416
H	-5.067466	-2.279327	1.533210
H	-4.127562	-3.201143	0.327336

30_CH3
11

C	-3.187661	0.967736	-0.738892
C	-2.339360	0.366926	0.133274
S	-2.791185	-1.221569	0.645834
S	-4.583631	0.051914	-1.190054
C	-4.229074	-1.315400	-0.246767
H	-3.056245	1.963991	-1.168402
H	-1.416619	0.802903	0.523207
C	-5.122890	-2.507435	-0.172955
H	-5.508966	-2.774971	-1.168526
H	-5.988417	-2.286429	0.475607
H	-4.589735	-3.371001	0.248713

31_CH3
28

C	2.848450	0.666638	-0.347289
N	2.195989	0.071164	-2.389500
C	1.748089	0.237575	-1.108666
C	3.984552	0.742767	-1.224273
C	3.541476	0.369200	-2.469373
C	1.368126	-0.326084	-3.519976
H	0.538053	0.381286	-3.661098
H	0.953454	-1.335106	-3.372189
H	1.985531	-0.324505	-4.426440
P	2.858861	1.162595	1.345708
C	2.474922	-0.192413	2.487874
C	1.672027	2.504696	1.628493
C	4.514251	1.771538	1.756145
H	4.076183	0.288269	-3.412847
H	4.997876	1.041718	-0.968020

H	1.932755	3.346996	0.971111
H	1.698545	2.832146	2.678947
H	0.657988	2.161029	1.378951
H	1.469501	-0.589303	2.285357
H	2.522243	0.173255	3.525470
H	3.208345	-0.999283	2.344627
H	4.791065	2.585263	1.071076
H	5.243243	0.953376	1.667260
H	4.517788	2.150467	2.789201
C	0.327877	-0.035140	-0.734799
H	0.041430	-1.075150	-0.964599
H	-0.372597	0.620319	-1.278115
H	0.156594	0.116454	0.338992

32_CH3
18

N	2.826695	-0.661153	0.783846
C	3.017858	0.371928	-1.262124
C	2.117880	-0.340493	-0.250025
C	4.265697	-0.258143	0.696313
C	4.428444	0.320162	-0.707505
C	2.378262	-1.326795	2.005045
H	3.038708	-2.183084	2.203449
H	1.344720	-1.675193	1.903407
H	2.460849	-0.618078	2.843891
O	2.600317	0.863609	-2.268382
H	4.876463	-1.149977	0.899202
H	4.458167	0.470375	1.499100
H	4.880169	1.322877	-0.728378
H	5.044269	-0.319026	-1.361640
C	0.688862	-0.570495	-0.519726
H	0.306120	0.285218	-1.098298
H	0.078933	-0.743181	0.375240
H	0.596171	-1.445695	-1.190496

33_CH3
19

N	-2.918037	0.527062	-0.960198
C	-2.177588	-0.000185	0.030186
N	-2.930397	-0.518126	1.013888
C	-4.307908	0.374973	-0.663157
C	-4.318163	-0.349085	0.699552
C	-2.472166	-1.177055	2.242198
H	-1.945823	-0.462267	2.890306
H	-1.822209	-2.029289	2.002220
H	-3.368600	-1.542246	2.760111
C	-2.419573	1.173555	-2.176560
H	-1.843732	0.461061	-2.784274
H	-1.797296	2.042547	-1.919737
H	-3.296390	1.512277	-2.743505
O	-5.218906	0.734303	-1.334096
O	-5.238773	-0.703192	1.359537
C	-0.700994	-0.013942	0.002874
H	-0.273622	-0.371629	0.945675
H	-0.361614	-0.673258	-0.815238
H	-0.317922	0.994765	-0.220152

34_CH3
28

N	-2.802336	-0.012604	1.362245
N	-4.361257	0.696209	-0.241946
C	-4.041663	-0.017122	0.849470
C	-2.496344	-0.656631	2.659089
H	-1.470392	-0.375167	2.916211
H	-2.562141	-1.750105	2.573696
H	-3.174752	-0.289769	3.439338
C	-5.760307	0.800859	-0.705520
H	-5.771775	1.528965	-1.522686
H	-6.409684	1.160389	0.102986
H	-6.116329	-0.170285	-1.076488
C	-3.381834	1.479535	-0.973360
C	-1.936679	1.119139	-0.705338
C	-1.709586	0.711676	0.735360
C	-1.009501	2.276530	-1.087002
C	-1.641046	-0.144910	-1.577707
O	-0.689317	0.876052	1.336030
O	-3.747783	2.274771	-1.787011
H	-2.304321	-0.989820	-1.338077
H	-0.599730	-0.458490	-1.416917
H	-1.773991	0.116434	-2.637280
H	-1.200512	3.157245	-0.457702
H	-1.177797	2.555865	-2.134614
H	0.035922	1.972339	-0.950165
C	-5.118709	-0.797203	1.530149
H	-5.754252	-0.112302	2.118975
H	-4.713259	-1.554852	2.205474
H	-5.765075	-1.287681	0.790784

35_CH3
29

N	2.514570	8.569410	0.886174
O	1.120418	8.620290	2.698922
C	3.728819	8.294911	0.346173
N	4.796756	8.189452	1.115748
C	2.224145	8.393240	2.290965
C	3.369176	7.829827	3.119515
C	4.679075	8.388409	2.569352
H	4.772842	9.469424	2.778481
H	5.528095	7.882611	3.047483
C	3.205105	8.248980	4.585002
H	3.275832	9.340835	4.703335
H	3.987174	7.780622	5.199688
H	2.225580	7.927443	4.962801
C	3.327915	6.291120	2.988755
H	3.496143	5.946497	1.956222
H	2.356373	5.904130	3.327873
H	4.109352	5.849530	3.624880
C	6.152360	7.951349	0.603145
C	1.379897	8.961170	0.029445
H	6.278907	8.358633	-0.405197
H	6.385566	6.874560	0.605414
H	6.859322	8.465428	1.266693
H	1.699922	9.708386	-0.706051
H	0.617789	9.393537	0.685997
H	0.944622	8.088826	-0.481343
C	3.833612	8.131641	-1.139436

H	4.020926	9.107394	-1.619628
H	2.902361	7.729265	-1.553364
H	4.650266	7.454805	-1.411544

36_CH3
39

C	-1.989377	-4.011798	-8.698410
C	-2.442707	-5.229325	-8.087321
C	-1.660232	-2.929087	-7.944094
C	-2.548427	-5.309837	-6.661675
C	-1.747962	-2.970738	-6.511498
C	-2.199888	-4.166069	-5.863718
H	-1.909337	-3.977173	-9.787201
C	-2.780994	-6.343723	-8.876291
C	-2.994874	-6.542639	-6.070791
C	-2.276906	-4.188126	-4.427811
C	-3.320066	-7.628335	-6.901464
C	-3.214761	-7.525076	-8.290107
H	-2.697127	-6.266006	-9.962566
C	-3.068099	-6.540060	-4.631678
H	-3.670005	-8.568304	-6.486569
H	-3.478658	-8.381599	-8.913495
C	-1.384659	-1.852113	-5.739800
C	-1.880907	-3.052007	-3.701164
C	-1.442972	-1.897395	-4.353002
H	-1.312024	-2.006828	-8.414989
C	-2.748244	-5.427140	-3.858922
H	-1.894248	-3.045453	-2.616120
H	-1.140123	-1.030773	-3.761835
H	-1.040668	-0.949114	-6.249295
N	-2.960790	-5.793878	-2.524077
N	-3.442051	-7.549494	-3.738775
C	-3.356233	-7.083890	-2.475243
C	-2.854386	-4.908977	-1.366048
H	-3.269436	-5.404658	-0.483945
H	-1.805431	-4.645070	-1.173646
H	-3.434642	-3.997455	-1.554260
C	-3.871973	-8.905339	-4.076220
H	-4.123592	-9.447235	-3.159560
H	-4.765115	-8.868541	-4.714018
H	-3.067279	-9.439808	-4.598210
C	-3.610720	-7.897438	-1.254939
H	-4.666016	-8.207655	-1.182117
H	-2.991039	-8.807231	-1.268050
H	-3.355192	-7.342911	-0.346091

37_CH3
25

C	-0.547187	-1.641731	-3.131477
C	-0.531900	-0.313801	-3.581358
C	-0.554851	-1.877867	-1.749848
C	-0.529977	0.737648	-2.661729
C	-0.548234	-0.796034	-0.815089
C	-0.538034	0.507254	-1.261064
C	-0.567972	-3.121760	-1.023260
C	-0.563293	-2.815795	0.381534
C	-0.554267	-1.393123	0.543964
H	-0.552207	-2.468009	-3.846239
H	-0.523989	-0.098626	-4.651347
H	-0.522468	1.766947	-3.029073
H	-0.535760	1.352275	-0.569779
C	-0.568396	-3.670863	1.494004
C	-0.553818	-0.838136	1.803919
C	-0.558233	-1.711573	2.923264
C	-0.563228	-3.101023	2.774634
H	-0.576343	-4.756412	1.371696
H	-0.550406	0.243050	1.956475
H	-0.558765	-1.280436	3.927607
H	-0.565915	-3.742046	3.657685
C	-0.561759	-4.466026	-1.629603
H	0.461887	-4.684243	-1.993568
H	-0.844812	-5.255579	-0.922265
H	-1.213560	-4.509646	-2.515546

38_CH3
27

C	7.504159	4.584893	12.535878
C	7.882632	3.719859	13.623585
C	6.918415	3.272008	14.571191
C	9.222832	3.250874	13.753233
C	7.284967	2.430676	15.611729
C	9.572136	2.385175	14.781333
C	8.609702	1.979634	15.716347
H	5.893711	3.642059	14.520935
H	9.959339	3.512046	12.992979
H	6.540874	2.120741	16.347880
H	10.593104	2.004878	14.851549
H	8.890957	1.300148	16.524532
C	8.419961	5.543472	11.980930
C	9.498133	6.071019	12.751366
C	8.232949	6.040403	10.658153
C	10.357521	7.020282	12.213502
C	9.103303	6.977899	10.126145
C	10.168368	7.469271	10.899505
H	9.610784	5.776140	13.794826
H	7.424121	5.654575	10.037195
H	11.163581	7.432484	12.823448
H	8.958202	7.338092	9.105619
H	10.843718	8.218133	10.478296
C	6.143285	4.446677	11.936849
H	6.235903	3.812947	11.033588
H	5.442552	3.921551	12.595426
H	5.717797	5.406790	11.616433

39_CH3
31

C	-1.241313	4.428632	10.357242
P	-2.201889	3.007464	10.172662
P	-0.654465	5.389534	9.045693
C	-0.731134	4.715112	11.774005
H	-1.206215	4.042716	12.505063
H	0.356166	4.566582	11.897203
H	-0.975144	5.734142	12.116116
C	0.492636	4.553049	7.889480
H	1.338113	4.143858	8.462151
H	-0.023532	3.722933	7.384591

H	0.866299	5.251949	7.125041
C	-1.939394	6.121345	7.976569
H	-2.504649	5.342677	7.447168
H	-2.630615	6.702595	8.604339
H	-1.469831	6.781141	7.230750
C	0.273460	6.792087	9.729626
H	-0.381174	7.397481	10.371949
H	1.123922	6.427685	10.322292
H	0.648429	7.412373	8.902587
C	-1.315729	1.447629	10.532631
H	-0.494288	1.328783	9.810739
H	-0.884958	1.490734	11.544483
H	-1.994433	0.583246	10.464915
C	-2.880898	2.780935	8.498895
H	-3.589268	3.584767	8.258563
H	-2.080671	2.747847	7.747051
H	-3.418951	1.821854	8.473344
C	-3.640060	3.020088	11.293199
H	-4.192650	2.070528	11.234870
H	-3.298387	3.171911	12.327573
H	-4.298320	3.856294	11.017199

40_CH3
27

N	-4.625223	6.390763	0.770826
N	-5.992884	6.443566	0.771918
C	-4.244531	5.158192	0.349592
C	-3.788031	7.512760	1.153910
H	-2.749196	7.193485	1.012838
H	-3.946364	7.782489	2.209142
H	-3.987393	8.382492	0.512058
C	-6.742871	7.631050	1.137571
H	-6.522213	7.930940	2.172921
H	-7.803979	7.369831	1.051964
H	-6.522407	8.461911	0.452124
C	-6.464648	5.248665	0.339748
C	-5.387393	4.389842	0.038295
O	-7.774519	5.124261	0.253139
C	-8.409167	3.839597	0.393350
H	-9.414673	4.040892	0.782585
H	-7.855192	3.209891	1.103638
H	-8.486304	3.341532	-0.582651
O	-2.945614	4.938291	0.299200
C	-2.414378	3.608282	0.193002
H	-2.972282	2.915337	0.837932
H	-1.374724	3.675242	0.534793
H	-2.436059	3.266647	-0.851292
C	-5.467536	3.027867	-0.598122
H	-6.355553	2.953513	-1.238519
H	-5.514029	2.209788	0.137991
H	-4.606104	2.844088	-1.251124

41_CH3
47

N	-2.329745	-0.719722	-0.733201
N	-2.151950	-1.031684	-2.915574
C	-1.469975	-1.121228	-1.726115
C	-3.562775	-0.376548	-1.292587
C	-3.461336	-0.608624	-2.675428
C	-2.123519	-0.905465	0.693442
H	-1.816169	0.027960	1.189137
H	-1.352945	-1.670077	0.853211
H	-3.062606	-1.253734	1.145494
C	-1.578989	-1.174799	-4.239763
C	-0.118881	-1.537025	-1.563347
H	-1.949337	-3.669384	-1.287814
H	3.255073	-1.910634	-1.617669
C	0.442771	-4.704112	-3.264655
C	1.752766	-4.205324	-3.173743
C	-1.773015	-4.015249	-2.316692
N	-0.399915	-3.739120	-2.699131
N	1.664214	-2.932541	-2.602072
C	2.768950	-1.984825	-2.600566
C	0.354688	-2.657553	-2.281364
H	-1.936487	-5.099080	-2.356422
H	3.511657	-2.318766	-3.335693
H	-2.501779	-3.527193	-2.979090
H	2.409161	-0.993482	-2.903811
H	-2.076483	-0.472764	-4.921630
H	-1.698638	-2.197659	-4.630547
H	-0.508302	-0.933474	-4.197804
C	0.792886	-0.777943	-0.608547
H	0.211525	-0.170011	0.095115
H	1.419842	-1.454799	-0.008293
C	2.848052	-4.949287	-3.609468
H	3.869456	-4.576071	-3.522725
C	2.586795	-6.212749	-4.149588
C	0.179681	-5.959934	-3.809052
H	3.419708	-6.825247	-4.499283
H	-0.834287	-6.354131	-3.893363
C	1.276930	-6.707576	-4.249621
H	1.108127	-7.696798	-4.679581
C	-4.747119	0.083738	-0.717090
H	-4.827698	0.288019	0.351594
C	-4.542750	-0.409294	-3.532644
C	-5.832158	0.287810	-1.575117
H	-4.471565	-0.608703	-4.603044
H	-6.774663	0.650502	-1.160246
C	-5.732846	0.045029	-2.956493
H	-6.601808	0.216481	-3.594501
H	1.460136	-0.069581	-1.124603

42_CH3
19

C	2.954558	1.928615	-1.563073
C	1.748408	1.735082	-0.940422
C	4.095749	2.369026	-0.829360
H	0.880248	1.391316	-1.507467
C	1.620753	1.978211	0.475525
C	3.969611	2.620757	0.570189
H	4.843349	2.960749	1.130282
C	2.772692	2.437677	1.212272
H	2.681846	2.631955	2.283561
H	3.053549	1.735350	-2.633256
C	0.403490	1.761791	1.125909
C	0.237658	1.998855	2.522187
N	0.094121	2.190405	3.659601

C	-0.749798	1.280151	0.439063
N	-1.691695	0.874693	-0.108393
C	5.392962	2.585525	-1.514048
H	6.245720	2.365030	-0.854050
H	5.472911	2.015273	-2.449594
H	5.474649	3.660363	-1.781291

43_CH3
17

C	3.013531	2.072455	-1.624080
C	1.831272	1.781302	-0.990985
C	4.103281	2.642533	-0.904837
H	0.995016	1.344469	-1.542787
C	1.674503	2.050869	0.420395
C	3.960275	2.918393	0.485232
H	4.799097	3.361169	1.026723
C	2.784681	2.638653	1.136100
H	2.672061	2.859102	2.200672
H	3.130528	1.867315	-2.690423
C	0.502806	1.750775	1.063799
H	0.372751	1.948264	2.132972
H	-0.340290	1.301366	0.529448
C	5.392703	2.916467	-1.593096
H	6.015533	2.000435	-1.543366
H	5.251556	3.149207	-2.658958
H	5.963629	3.717357	-1.103255

44_CH3
69

C	-1.013489	0.882853	-0.410061
C	0.348893	0.958500	-0.389366
N	0.798152	-0.110981	0.370034
C	-0.254948	-0.827553	0.805880
N	-1.367258	-0.230323	0.340467
C	-2.718355	-0.641904	0.646779
C	-3.330696	-0.088954	1.788730
C	-3.335699	-1.582097	-0.200874
C	-4.647021	-1.955080	0.119970
C	-5.290588	-1.420827	1.237833
C	-4.641178	-0.503009	2.063098
C	-2.602717	0.866097	2.727660
H	-5.158270	-0.104261	2.937589
H	-5.171077	-2.675870	-0.509636
H	-6.309072	-1.735834	1.477242
C	-2.620632	-2.151234	-1.419026
C	-2.159900	0.136144	4.010023
C	-3.432277	2.115050	3.062894
H	-1.692224	1.218818	2.218026
H	-4.319114	1.867938	3.666883
H	-3.777799	2.629721	2.153142
H	-2.827417	2.823818	3.649571
H	-3.029652	-0.252399	4.563272
H	-1.615158	0.822825	4.676379
H	-1.500119	-0.717197	3.792727
C	-2.893811	-3.645171	-1.634112
H	-1.536362	-2.047390	-1.245651
C	-2.956654	-1.341135	-2.682709
H	-2.696735	-4.231067	-0.723075
H	-2.246390	-4.032022	-2.435508
H	-3.933949	-3.833335	-1.942949
C	-0.180379	-2.058277	1.625579
H	-2.414221	-1.741273	-3.553330
H	-2.683892	-0.279778	-2.573970
H	-4.035211	-1.390913	-2.900825
C	2.165513	-0.371299	0.754648
C	2.952042	-1.189694	-0.076842
C	2.618877	0.189144	1.965988
C	4.272417	-1.420476	0.331423
C	3.947559	-0.073747	2.322246
C	4.763627	-0.866387	1.513670
H	5.792632	-1.068906	1.820206
H	4.349180	0.340884	3.248267
C	2.404682	-1.791780	-1.362974
H	4.923249	-2.046587	-0.281412
C	1.719791	1.049522	2.846842
C	2.866748	-0.989951	-2.591037
H	1.305169	-1.718925	-1.323549
C	2.752409	-3.279804	-1.512889
C	1.984328	2.545654	2.613188
C	1.833192	0.698705	4.336659
H	0.675011	0.857798	2.555012
H	3.023334	2.805835	2.871541
H	1.319596	3.157442	3.242026
H	1.813250	2.832063	1.563752
H	2.807669	0.996762	4.752995
H	1.702780	-0.379685	4.518047
H	1.062205	1.236240	4.909120
H	2.562316	0.066858	-2.526370
H	2.434353	-1.412956	-3.511378
H	3.963935	-1.017631	-2.685383
H	3.830571	-3.434747	-1.674263
H	2.229450	-3.704044	-2.383585
H	2.457979	-3.858536	-0.623392
H	-1.764909	1.513717	-0.874541
H	1.039982	1.668362	-0.833333
H	-1.187931	-2.436355	1.835893
H	0.393715	-2.830925	1.091490
H	0.338286	-1.857682	2.575177

45_CH3
51

C	-1.135952	0.984749	-0.097434
C	0.218940	1.137572	-0.085112
N	0.742140	0.056502	0.612265
C	-0.264910	-0.740413	1.020158
N	-1.415986	-0.186463	0.592257
C	-2.729353	-0.741762	0.818035
C	-3.429637	-0.355662	1.971647
C	-3.241600	-1.649697	-0.124662
C	-4.510953	-2.180215	0.122896
C	-5.253956	-1.827170	1.260379
C	-4.696224	-0.918109	2.170001
C	-2.838667	0.621636	2.955821
H	-5.262912	-0.636194	3.060945
H	-4.933737	-2.889279	-0.593731
C	-6.629134	-2.399877	1.479671
C	-2.453123	-2.029840	-1.352249

C	2.146284	-0.177440	0.854322
C	2.846110	-1.017784	-0.025441
C	2.746238	0.446410	1.962300
C	4.206962	-1.223436	0.231661
C	4.106657	0.204775	2.172617
C	4.854786	-0.621623	1.318942
C	6.324937	-0.835667	1.563197
H	4.598040	0.674329	3.028836
C	2.156589	-1.686979	-1.187135
H	4.776586	-1.872883	-0.437760
C	1.950123	1.333509	2.885081
H	-2.223283	-1.150501	-1.976228
H	-3.012508	-2.742917	-1.972108
H	-1.488904	-2.499317	-1.093731
H	-2.608234	1.589379	2.481010
H	-1.897102	0.246164	3.391765
H	-3.533505	0.812143	3.784368
H	1.061505	0.816100	3.283792
H	1.589201	2.238531	2.368766
H	2.559410	1.658438	3.738810
H	1.543390	-0.979095	-1.766887
H	1.486540	-2.495522	-0.846403
H	2.888670	-2.138049	-1.870206
H	-6.654687	-3.476891	1.253285
H	-7.361571	-1.911739	0.814239
H	-6.970347	-2.254477	2.514374
H	6.528249	-1.036620	2.626504
H	6.897650	0.066940	1.289843
H	6.718815	-1.673226	0.970424
H	0.862841	1.904518	-0.505576
H	-1.927014	1.591114	-0.529128
C	-0.140021	-1.998667	1.792582
H	0.889884	-2.119586	2.151355
H	-0.401972	-2.865662	1.165153
H	-0.831544	-1.993977	2.647770

46_CH3
53

C	-1.259302	0.959076	-0.393136
C	0.271354	1.157668	-0.449324
N	0.775195	0.057195	0.397449
C	-0.222257	-0.703782	0.834948
N	-1.406359	-0.267457	0.419878
C	-2.687342	-0.811159	0.774690
C	-3.241999	-0.474377	2.022524
C	-3.353156	-1.643496	-0.145255
C	-4.607699	-2.140244	0.219818
C	-5.201481	-1.826548	1.452039
C	-4.501709	-0.996830	2.338025
C	-2.495803	0.396097	3.002457
H	-4.949101	-0.747173	3.303521
H	-5.137717	-2.795018	-0.477251
C	-6.564635	-2.364037	1.799126
C	-2.741022	-1.987576	-1.479343
C	2.167449	-0.156078	0.677682
C	2.930852	-0.937786	-0.208781
C	2.720135	0.432659	1.829526
C	4.283756	-1.126434	0.092283
C	4.078383	0.214668	2.083908
C	4.877258	-0.557657	1.228638
C	6.344293	-0.752213	1.506838
H	4.525715	0.660603	2.976029
C	2.318533	-1.545174	-1.446050
H	4.892735	-1.736338	-0.580310
C	1.877007	1.269362	2.759131
H	-2.850672	-1.158359	-2.199813
H	-3.232226	-2.864687	-1.922158
H	-1.664935	-2.207408	-1.396832
H	-2.051468	1.281415	2.520987
H	-1.672914	-0.156124	3.488975
H	-3.162791	0.748590	3.800736
H	1.024506	0.703387	3.170491
H	1.458083	2.151982	2.247652
H	2.471540	1.632838	3.607862
H	2.097052	-0.776458	-2.206156
H	1.372564	-2.068735	-1.231926
H	3.003068	-2.269308	-1.907905
H	-6.625679	-3.447050	1.608486
H	-7.340944	-1.883465	1.180086
H	-6.817146	-2.184290	2.853573
H	6.943728	-0.011950	0.949174
H	6.685309	-1.749912	1.192402
H	6.575148	-0.625662	2.574451
H	0.598101	2.124860	-0.041217
H	0.679905	1.060032	-1.465858
H	-1.711075	0.807880	-1.383368
H	-1.786441	1.789520	0.100088
C	-0.019551	-1.909771	1.677260
H	0.465555	-1.626488	2.623113
H	0.662379	-2.605108	1.165594
H	-0.975187	-2.405494	1.883127

47_CH3
56

C	-1.085677	2.138457	-0.200975
C	-0.795049	0.724866	-0.711135
C	-1.503082	0.519144	-2.047228
C	0.711466	0.400169	-0.793867
H	0.904013	-0.210890	-1.687460
H	1.315222	1.312373	-0.888957
C	1.078718	-0.413333	0.471671
C	1.677696	0.465836	1.594340
C	-0.271987	-0.905430	0.929410
N	-1.253149	-0.311775	0.325383
C	-2.655563	-0.570150	0.602205
C	-3.297840	0.155889	1.630163
C	-3.311418	-1.567377	-0.154175
C	-4.680897	-1.749644	0.076302
C	-5.358976	-0.996741	1.032777
C	-4.669075	-0.070930	1.811597
C	-2.562490	1.057441	2.615392
H	-5.201941	0.477503	2.590434
H	-5.222274	-2.507115	-0.492996
H	-6.425852	-1.161815	1.200092
C	-2.581316	-2.524449	-1.089214
C	-2.523733	0.399413	4.007422
C	-3.156211	2.470269	2.705242
H	-1.521331	1.159244	2.279372

H	-4.173105	2.456095	3.127716
H	-3.207650	2.958917	1.721758
H	-2.539663	3.099207	3.365331
H	-3.537750	0.276529	4.417858
H	-1.952171	1.024846	4.710462
H	-2.055981	-0.595925	3.980897
C	-2.479932	-3.916928	-0.437043
H	-1.553084	-2.158677	-1.227136
C	-3.224250	-2.623260	-2.479360
H	-1.982856	-3.877486	0.543199
H	-1.909310	-4.603277	-1.081640
H	-3.478136	-4.353931	-0.280086
H	-1.104587	1.258485	-2.757444
H	-2.587790	0.677811	-1.965953
H	-1.315825	-0.476180	-2.466966
H	-0.714239	2.856694	-0.945682
H	-0.589946	2.359050	0.752948
H	-2.165512	2.302691	-0.088210
H	2.627111	0.897290	1.243459
H	1.889274	-0.126986	2.496604
H	1.011284	1.294576	1.874325
C	2.029905	-1.577523	0.150905
H	2.945282	-1.179305	-0.312203
H	1.573580	-2.286758	-0.557072
H	2.325697	-2.127834	1.056517
C	-0.446390	-1.942288	1.971316
H	0.013260	-2.879100	1.617229
H	-1.497174	-2.123123	2.219790
H	-2.610550	-3.258643	-3.135826
H	-3.327021	-1.637790	-2.956155
H	-4.225372	-3.078890	-2.431513
H	0.103548	-1.643076	2.877563

48_CH3
75

C	-1.256154	2.201840	0.056837
C	-0.909087	0.876576	-0.629028
C	-1.661221	0.810252	-1.956993
C	0.614837	0.672211	-0.770473
H	0.861059	0.312923	-1.771314
H	1.137152	1.627726	-0.640436
C	1.067585	-0.346542	0.317867
C	1.715159	0.473242	1.487257
C	-0.250075	-0.903761	0.795611
N	-1.276945	-0.287472	0.289413
C	-2.657383	-0.619863	0.600797
C	-3.269630	-0.054890	1.742511
C	-3.322576	-1.536168	-0.246149
C	-4.672114	-1.797176	0.021395
C	-5.322775	-1.199368	1.099389
C	-4.622458	-0.354383	1.956556
C	-2.519698	0.750105	2.799036
H	-5.131115	0.070029	2.823942
H	-5.217054	-2.495529	-0.616312
H	-6.373841	-1.424043	1.295203
C	-2.608477	-2.337003	-1.328348
C	-2.441733	-0.042079	4.118559
C	-3.130237	2.137136	3.051899
H	-1.488567	0.902774	2.449401

H	-4.135748	2.059031	3.494269
H	-3.215489	2.725669	2.128063
H	-2.506034	2.703145	3.760164
H	-3.441194	-0.179226	4.558793
H	-1.826120	0.499081	4.853684
H	-2.008161	-1.042905	3.978375
C	-2.373776	-3.782638	-0.848632
H	-1.618071	-1.895293	-1.490086
C	-3.338413	-2.323358	-2.677840
H	-1.786389	-3.818684	0.080511
H	-1.827325	-4.355598	-1.613856
H	-3.327050	-4.297818	-0.653517
H	-1.324973	1.652877	-2.578776
H	-2.746224	0.910920	-1.811352
H	-1.464633	-0.111548	-2.514606
H	-0.956948	3.021926	-0.611624
H	-0.728394	2.334790	1.009817
H	-2.336640	2.288276	0.230374
H	2.429254	1.162366	1.006815
C	2.482564	-0.295563	2.560858
H	0.942800	1.114167	1.944753
C	2.045558	-1.458568	-0.231776
H	2.764445	-0.894323	-0.854462
C	1.354126	-2.522855	-1.156030
C	2.885901	-2.128861	0.877229
C	-0.406427	-1.980444	1.803732
H	-2.730417	-2.835450	-3.439714
H	-3.534532	-1.299195	-3.028153
H	-4.303678	-2.850345	-2.625853
C	3.528357	-1.163583	1.867163
C	3.097122	0.667001	3.577659
H	3.674767	-2.720098	0.394556

49_CH3
37

C	-0.415497	11.922789	4.388311
C	1.060273	11.126016	2.651334
C	1.009577	12.398126	1.940945
O	1.611318	12.595730	0.882269
C	0.055999	13.347879	2.497525
C	0.652979	9.752123	4.649100
C	-1.115603	14.260519	4.449466
C	2.977301	10.188789	1.429226
H	3.450728	11.133882	1.711521
H	2.875179	10.162761	0.333417
H	3.622719	9.353929	1.751078
C	0.983827	8.793722	1.836608
H	-0.076787	8.871943	2.110530
H	1.434274	7.946807	2.381995
H	1.035995	8.568707	0.757798
C	0.546372	15.201787	0.950297
H	1.584205	15.005766	1.233531
H	0.322225	16.272121	1.096449
H	0.423130	14.948066	-0.114068
C	-1.785091	14.705696	1.594532
H	-1.998340	14.729161	0.512634
H	-2.082242	15.678000	2.023135
H	-2.402704	13.914653	2.038571
N	0.388568	10.970914	3.855258

N	-0.507672	13.112106	3.741151
N	1.681146	10.050769	2.095630
N	-0.365235	14.426439	1.793372
H	1.603563	9.334610	4.305669
H	-0.139763	9.003469	4.517625
H	0.740436	10.018310	5.707370
H	-0.712132	15.170846	3.995749
H	-0.823740	14.229175	5.504808
H	-2.210448	14.270324	4.365201
C	-1.164109	11.679424	5.662753
H	-2.055433	12.314430	5.724685
H	-1.510773	10.639270	5.723328
H	-0.547918	11.878408	6.558721

50_CH3

38

P	1.066321	-1.442629	-0.324054
C	1.075557	0.002285	-1.422884
H	2.096831	0.178312	-1.788395
H	0.416641	-0.193354	-2.280596
H	0.725380	0.889442	-0.876779
C	1.797977	-2.853145	-1.174312
C	2.939601	-3.485793	-0.659169
C	1.185308	-3.331852	-2.347504
C	3.470127	-4.594985	-1.322980
C	1.723249	-4.439091	-3.001259
C	2.864807	-5.069025	-2.489624
H	3.408941	-3.118805	0.255472
H	0.284475	-2.854797	-2.742764
H	4.354789	-5.093385	-0.921145
H	1.246463	-4.819155	-3.907447
H	3.283072	-5.936228	-3.005707
C	-0.639772	-1.838505	0.103488
C	-1.575148	-0.803443	0.278313
C	-1.021710	-3.177071	0.298853
C	-2.889773	-1.112481	0.626185
C	-2.339298	-3.474556	0.652743
C	-3.272372	-2.445589	0.810314
H	-1.288812	0.241654	0.141417
H	-0.298888	-3.983779	0.162452
H	-3.618145	-0.309413	0.755499
H	-2.641399	-4.513670	0.801602
H	-4.303051	-2.683739	1.083191
C	2.001488	-1.038784	1.161500
C	1.561953	-1.508677	2.410008
C	3.169593	-0.260705	1.067001
C	2.287574	-1.192125	3.560102
C	3.883428	0.055526	2.223041
C	3.441915	-0.407867	3.467447
H	0.650535	-2.104955	2.486872
H	3.528091	0.104310	0.102010
H	1.941688	-1.554589	4.530762
H	4.785802	0.666488	2.151545
H	4.001771	-0.150216	4.369438

51_CH3

15

C	-0.530961	0.023890	-4.074153
C	-0.496757	1.226333	-3.363474
C	-0.503506	1.194373	-1.976184
N	-0.543883	0.015562	-1.312129
C	-0.574779	-1.160661	-1.985196
C	-0.569001	-1.184774	-3.370115
H	-0.528502	0.026807	-5.166722
H	-0.465033	2.190210	-3.874162
H	-0.476034	2.098643	-1.366453
H	-0.605126	-2.067264	-1.378359
H	-0.597279	-2.145242	-3.887260
C	-0.533664	-0.009144	0.166809
H	-0.660642	1.011180	0.545735
H	-1.359125	-0.637669	0.524519
H	0.424602	-0.418092	0.517277

Mg Radicals

MgH_rad

2

Mg	-1.044570	0.120000	2.040068
H	-2.788988	0.120000	2.040068

01_Mg_rad

40

C	1.716217	1.601387	-1.652319
N	0.821038	1.396762	-0.491733
C	0.229767	0.141501	-0.438533
C	0.896776	-0.733726	-1.503680
C	2.077088	0.142101	-2.000708
C	2.967637	2.395944	-1.257056
C	0.980001	2.318349	-2.801405
C	1.411078	-2.061420	-0.915793
C	-0.090603	-1.069385	-2.643880
C	0.186964	2.526522	0.135446
Mg	-1.300718	-0.539508	0.759680
N	-2.282816	-0.611919	2.606397
C	-3.036135	-1.579772	2.107043
N	-2.835337	-1.941836	0.850750
C	-2.376678	-0.250900	3.998614
C	-3.700473	-2.892324	0.203285
H	2.294279	0.010681	-3.072531
H	2.988183	-0.133890	-1.446816
H	2.732202	3.436823	-0.981035
H	3.467232	1.917916	-0.400782
H	3.676298	2.429875	-2.100264
H	1.625894	2.396324	-3.690227
H	0.067462	1.776060	-3.085197
H	0.684681	3.339586	-2.510222
H	0.577009	-2.666149	-0.518092
H	1.922063	-2.671449	-1.681598
H	2.117188	-1.880148	-0.090763
H	0.401399	-1.661196	-3.436599
H	-0.936443	-1.662213	-2.257306
H	-0.511597	-0.163835	-3.103121
H	0.871401	3.386172	0.194565
H	-0.732250	2.854291	-0.397982
H	-0.106632	2.267993	1.167659
H	-3.809464	-2.077715	2.730047
H	-3.189462	-0.794397	4.516682

H	-1.439521	-0.473781	4.543554
H	-2.571468	0.829800	4.125203
H	-4.300329	-3.477456	0.927526
H	-4.410722	-2.396500	-0.487152
H	-3.115667	-3.608670	-0.399217

02_Mg_rad
28

N	3.298406	0.371284	0.842745
N	3.504880	-0.480853	-1.117941
C	2.630425	-0.383205	-0.075753
C	4.564866	0.716550	0.396197
C	4.694525	0.178931	-0.849995
C	2.758013	0.691765	2.154306
H	3.104442	-0.033750	2.908319
H	1.661664	0.653607	2.095883
H	3.074233	1.700685	2.456002
C	3.184723	-1.162971	-2.359029
H	2.576538	-2.053494	-2.129579
H	4.109452	-1.484054	-2.859062
H	2.613589	-0.509461	-3.036348
Mg	0.746303	-1.529793	0.234482
N	-0.758037	-0.587113	-0.923535
N	-0.608662	-0.276106	1.328957
C	-1.241019	-0.055904	0.183870
H	-2.172579	0.553870	0.160671
C	-1.200751	0.159823	2.567029
H	-1.596968	-0.685467	3.163824
H	-2.042089	0.863201	2.408472
H	-0.463944	0.677466	3.210887
C	-1.382400	-0.342459	-2.195212
H	-1.695447	-1.285377	-2.682909
H	-0.693298	0.161786	-2.900644
H	-2.283671	0.296557	-2.110878
H	5.518099	0.215882	-1.558571
H	5.256577	1.306792	0.991766

03_Mg_rad
30

N	3.076622	1.002084	0.708679
N	2.695232	-0.455964	-0.857125
C	2.113751	0.494861	-0.092382
C	4.358456	0.305142	0.575716
C	4.155789	-0.459039	-0.738133
C	2.860316	1.941583	1.777726
H	2.884899	1.447708	2.767377
H	1.882649	2.428075	1.642273
H	3.635620	2.727098	1.765108
C	2.045943	-1.119373	-1.962623
H	2.263522	-0.616541	-2.924964
H	0.959980	-1.108966	-1.795195
H	2.389444	-2.164708	-2.031876
H	4.522029	-0.375202	1.434775
H	5.200543	1.015707	0.548249
H	4.613075	0.069427	-1.597548
H	4.555431	-1.484815	-0.721062
Mg	0.103739	1.353549	-0.572993
N	-1.454742	0.692901	0.701090
N	-1.225898	-0.106438	-1.418515
C	-1.911556	-0.055013	-0.286578
C	-2.167547	0.776248	1.946365
H	-1.526922	0.483356	2.799658
H	-3.059864	0.119938	1.964649
H	-2.517625	1.806707	2.149981
C	-1.767898	-0.808163	-2.552846
H	-0.996395	-1.423351	-3.053296
H	-2.166037	-0.119371	-3.323759
H	-2.592600	-1.492425	-2.270822
H	-2.863214	-0.622052	-0.174983

04_Mg_rad
22

O	-5.680451	1.203624	-0.796153
C	-5.240509	1.921938	0.303101
O	-4.405976	1.110884	1.049709
C	-4.120881	-0.070091	0.303736
C	-5.329670	-0.165289	-0.621190
H	-4.013603	-0.917718	0.995900
H	-3.180399	0.051576	-0.268897
H	-6.163407	-0.718146	-0.146022
H	-5.109719	-0.611992	-1.601783
Mg	-5.817325	3.864221	0.663998
N	-6.865920	5.507550	-0.046968
N	-6.102627	5.343016	2.087226
C	-6.788784	6.013190	1.174541
H	-7.304792	6.961923	1.431707
C	-7.600403	6.191588	-1.081977
H	-8.229949	5.486236	-1.651257
H	-6.930292	6.683155	-1.813269
H	-8.268798	6.971795	-0.669965
C	-6.015461	5.818755	3.444821
H	-6.311192	5.033164	4.162584
H	-6.674073	6.689825	3.622170
H	-4.985564	6.124277	3.709643

05_Mg_rad
26

C	-2.404385	1.970173	0.139731
C	-1.481060	0.782678	-0.116130
N	-2.153069	-0.233334	0.676663
O	-3.681704	1.368172	0.356518
C	-3.525409	0.040476	0.708584
C	-1.705164	-1.596221	0.589255
H	-1.839852	-2.024205	-0.430027
H	-2.277679	-2.220511	1.294029
H	-0.641081	-1.675097	0.864818
Mg	-5.095783	-1.265888	1.006558
H	-1.456106	0.510021	-1.196040
H	-0.447497	0.957017	0.224984
H	-2.094270	2.527828	1.043707
H	-2.471762	2.671976	-0.705626
N	-5.484711	-3.253293	1.488914
N	-7.072797	-1.629849	1.527725
C	-6.743342	-2.901676	1.700083
H	-7.501059	-3.650249	2.014029
C	-5.053264	-4.620895	1.625317

H	-4.692282	-5.034667	0.664528
H	-5.863045	-5.283209	1.986880
H	-4.214290	-4.711439	2.339100
C	-8.415074	-1.163468	1.770106
H	-8.867146	-0.729410	0.859118
H	-8.435401	-0.370231	2.539158
H	-9.082561	-1.974583	2.118626

06_Mg_rad
24

Mg	-0.974964	-0.382975	-2.710482
O	1.878554	-0.368982	-2.116093
N	-1.836317	-1.166852	-4.432762
N	-2.878641	0.357766	-3.115156
C	2.971182	-0.652400	-1.238172
C	0.655109	-0.464607	-1.450741
C	0.943510	-0.755533	0.007985
C	2.434847	-0.393223	0.169902
C	-2.893277	-0.408759	-4.195217
C	-1.763002	-2.001621	-5.603600
C	-3.966198	1.260225	-2.839592
H	2.952559	-0.989599	0.935401
H	2.547876	0.670833	0.433785
H	-3.770843	-0.415988	-4.876311
H	-2.729606	-2.053638	-6.140492
H	-1.007481	-1.632794	-6.322636
H	-1.468775	-3.032351	-5.336856
H	-4.853169	1.043394	-3.465138
H	-4.282783	1.193567	-1.783946
H	-3.683653	2.314539	-3.024091
H	0.290164	-0.185059	0.690733
H	0.779216	-1.826020	0.254321
H	3.280636	-1.709207	-1.365563
H	3.821545	-0.013759	-1.521248

07_Mg_rad
32

C	-0.726090	2.127006	-0.174303
N	-1.809615	1.878858	-1.034656
C	-2.567909	0.651767	-0.814283
C	-2.643689	3.018491	-1.320118
N	0.171214	1.032434	-0.119313
C	1.104197	1.076759	0.979303
C	0.799399	0.665866	-1.383772
H	0.562198	1.171548	1.933309
H	1.824177	1.930669	0.903981
H	1.703808	0.153442	1.014587
H	1.265031	-0.331712	-1.314367
H	1.586393	1.396046	-1.676972
H	0.041869	0.653617	-2.177984
H	-3.389346	2.766507	-2.090214
H	-2.033185	3.853296	-1.699553
H	-3.204878	3.377233	-0.421742
H	-3.117854	0.360965	-1.724679
H	-3.304093	0.768833	0.011799
H	-1.877130	-0.153247	-0.534521
Mg	-0.362811	3.882587	0.888869
N	-0.344795	5.966032	1.081316
N	0.644086	4.570189	2.574615
C	0.377752	5.803767	2.180862
C	-0.682974	7.286025	0.611726
H	-1.762813	7.501267	0.727293
H	-0.444706	7.402229	-0.460867
H	-0.131779	8.076225	1.156270
H	0.760972	6.680503	2.745491
C	1.409004	4.302076	3.763948
H	2.259948	3.631584	3.547930
H	0.795964	3.798416	4.535110
H	1.823521	5.223403	4.217944

08_Mg_rad
34

Mg	-2.226793	-0.120452	-1.889313
N	0.603310	0.023253	-0.225971
N	-2.480510	0.281085	-3.992714
N	-2.555693	-1.828826	-3.152172
N	1.023965	0.093258	-2.323087
C	-0.104799	-0.060836	1.040132
C	0.853059	0.103613	-3.767093
C	2.893842	0.154611	0.848756
C	3.552536	0.275244	-2.413106
C	2.259743	0.167138	-1.679235
C	-0.004721	-0.001913	-1.441366
C	1.990036	0.120612	-0.336285
C	-2.756440	-1.005307	-4.161968
C	-2.780499	1.231427	-5.031350
C	-2.890287	-3.222847	-3.262451
H	0.062184	-1.036634	1.523409
H	0.230611	0.734615	1.722194
H	-1.181243	0.064357	0.841404
H	1.273442	-0.810170	-4.215280
H	-0.222138	0.160083	-3.991176
H	1.362219	0.974969	-4.205352
H	2.709914	1.038110	1.483465
H	2.764715	-0.735926	1.486633
H	3.943762	0.188631	0.525854
H	3.614104	1.203738	-3.006068
H	4.393973	0.274274	-1.706403
H	3.700737	-0.565608	-3.110848
H	-3.175533	-1.374644	-5.126680
H	-3.263796	0.759789	-5.910699
H	-3.460844	2.023578	-4.669250
H	-1.870319	1.748672	-5.399559
H	-3.232285	-3.499244	-4.280907
H	-2.027482	-3.869503	-3.014247
H	-3.697299	-3.503520	-2.560226

09_Mg_rad
30

N	2.610280	-0.691029	1.364355
N	2.669108	-1.076467	-0.745197
N	1.846049	-1.234871	0.357284
C	3.744079	-0.068646	0.874284
C	3.801180	-0.286304	-0.487342
C	1.971024	-0.494084	2.637480
H	2.716417	-0.195532	3.386683

H	1.498783	-1.434424	2.963144
H	1.181714	0.281871	2.587554
C	2.051447	-1.247470	-2.028508
H	1.478224	-0.351225	-2.350629
H	1.356495	-2.099056	-1.986018
H	2.822250	-1.461698	-2.785305
C	4.709611	0.632323	1.774001
H	4.238317	1.461360	2.329324
H	5.541041	1.053207	1.189192
H	5.143001	-0.055372	2.522659
Mg	5.255361	0.571228	-1.678967
N	7.069005	1.568699	-1.503573
N	6.063479	1.151869	-3.497508
C	7.077311	1.674811	-2.822146
H	7.905187	2.192833	-3.350619
C	6.027424	1.218010	-4.937289
H	5.051707	1.591469	-5.294595
H	6.183211	0.225469	-5.401175
H	6.804584	1.893032	-5.341586
C	8.126248	2.128219	-0.702631
H	7.730490	2.848722	0.035943
H	8.876371	2.660791	-1.318172
H	8.661217	1.347381	-0.130495

50_Mg_rad
47

P	-0.141317	0.976141	0.623323
Mg	2.110145	1.488054	2.045645
N	1.295896	3.453392	2.204751
N	0.979388	1.860997	3.794560
C	0.149907	-1.782516	0.588064
C	-0.271357	-3.114065	0.579243
C	-1.635870	-3.417799	0.600606
C	-2.577563	-2.384542	0.626335
C	-2.159223	-1.051373	0.629779
C	1.089467	2.791361	-2.842459
C	0.343569	2.155221	-3.839148
C	-0.574246	1.156330	-3.494878
C	-0.745434	0.788233	-2.157671
C	0.922868	2.422596	-1.504625
C	-3.514979	3.612857	2.398050
C	-3.033575	2.492730	3.085824
C	-3.011098	3.914047	1.129548
C	-2.031697	3.102722	0.547883
C	-2.046432	1.690382	2.515097
C	0.001205	1.421435	-1.152223
C	-1.541415	1.982398	1.235344
C	-0.790073	-0.738315	0.609460
C	1.041075	4.757153	1.651838
C	0.460324	1.418317	5.060851
C	0.766678	3.094270	3.361747
H	-3.645222	-2.618478	0.645931
H	-2.899620	-0.249361	0.656473
H	-1.967487	-4.459483	0.602572
H	0.469628	-3.917096	0.564822
H	1.218679	-1.545779	0.585477
H	-1.454000	-0.000006	-1.893973
H	0.482033	2.438681	-4.885986
H	-1.157699	0.655931	-4.271847
H	1.513517	2.914348	-0.727520
H	1.809453	3.569109	-3.108334
H	-3.386910	4.782865	0.583185
H	-1.647373	3.344348	-0.445118
H	-1.660969	0.829508	3.064543
H	-4.280020	4.248594	2.850390
H	-3.425415	2.243889	4.075597
H	0.399768	4.705793	0.750638
H	1.979206	5.253585	1.343537
H	0.529005	5.426683	2.369486
H	-0.059450	2.228061	5.609818
H	1.263569	1.033095	5.715732
H	-0.265175	0.590046	4.942927
H	0.149978	3.807019	3.951440

MgH Adducts
MgH_2
3

Mg	-1.127279	0.120003	2.040068
H	-2.827573	0.119999	2.040069
H	0.573015	0.119999	2.040069

01_MgH
41

Mg	-2.346082	-0.028301	-1.994488
N	-2.657764	0.266689	-4.091866
N	-2.607956	-1.810097	-3.162780
N	0.875755	0.109892	-2.360180
C	0.721201	0.050248	-3.810748
C	-0.246420	1.283227	0.701776
C	0.030729	-1.211008	0.498605
C	2.773219	-1.424553	-1.899683
C	3.222434	1.005205	-2.382637
C	2.251973	0.011275	-1.741180
C	-0.132985	0.156895	-1.536852
C	0.398998	0.156314	-0.117616
C	1.929343	0.362928	-0.276353
C	-2.871005	-1.037771	-4.198957
C	-3.005096	1.149401	-5.174724
C	-2.924555	-3.213046	-3.200237
H	1.073332	-0.920016	-4.192971
H	-0.342608	0.175335	-4.057001
H	1.308725	0.847682	-4.287516
H	3.760041	-1.512550	-1.419952
H	2.098643	-2.149881	-1.424130
H	2.888970	-1.701549	-2.958758
H	2.793997	2.019432	-2.403430
H	4.148308	1.040285	-1.788165
H	3.497652	0.716842	-3.409003
H	0.439824	-1.285978	1.519114
H	-1.062997	-1.323025	0.556373
H	0.425682	-2.050568	-0.093350
H	-1.336106	1.145642	0.766591
H	0.179677	1.299524	1.718939
H	-0.054887	2.264501	0.237885
H	-3.324308	0.476056	-0.644848
H	2.519866	-0.240791	0.427500
H	2.178908	1.418360	-0.085971

H	-3.284501	-1.468206	-5.140383
H	-3.462245	0.611002	-6.029354
H	-3.726526	1.921250	-4.850077
H	-2.122689	1.694649	-5.567671
H	-3.272303	-3.545014	-4.199917
H	-2.051834	-3.835659	-2.928137
H	-3.722009	-3.468667	-2.477464

02_MgH
29

N	3.130227	-0.704869	0.465416
N	3.663389	0.703479	-1.059805
C	2.585604	0.128803	-0.460628
C	4.514463	-0.654450	0.447491
C	4.853226	0.242571	-0.521246
C	2.352688	-1.519157	1.387797
H	1.307025	-1.523072	1.047945
H	2.408823	-1.100747	2.404954
H	2.741391	-2.548331	1.398149
C	3.581134	1.707664	-2.111363
H	2.548581	1.731516	-2.491276
H	4.263343	1.443973	-2.932708
H	3.858482	2.697114	-1.714168
Mg	0.412832	0.403961	-1.054664
N	-0.682093	-1.191770	-0.102463
N	-0.830409	0.978234	0.572370
C	-1.250818	-0.267912	0.660923
H	-2.074581	-0.539896	1.358903
C	-1.457407	2.013843	1.348415
H	-0.718899	2.578819	1.947415
H	-2.212700	1.611765	2.052640
H	-1.970419	2.752643	0.703238
C	-1.172315	-2.545066	-0.074824
H	-0.377267	-3.266223	0.200823
H	-1.557469	-2.864625	-1.061686
H	-1.995178	-2.681684	0.654614
H	0.380547	1.088101	-2.666199
H	5.823659	0.584838	-0.871908
H	5.132394	-1.256679	1.108377

03_MgH
31

N	3.395175	-0.241018	1.171960
N	2.975632	-0.038835	-0.945347
C	2.425093	-0.302369	0.248363
C	4.705018	0.139667	0.624500
C	4.428911	0.173248	-0.887610
C	3.234628	-0.467012	2.590165
H	3.947330	-1.233655	2.938359
H	2.217852	-0.835540	2.786376
H	3.420187	0.457079	3.166958
C	2.286578	-0.082984	-2.214736
H	2.453485	0.848766	-2.782095
H	1.211134	-0.195924	-2.025688
H	2.645683	-0.926287	-2.832492
H	5.475441	-0.595559	0.908263
H	5.019576	1.120137	1.024632
H	4.705165	1.131620	-1.357239
H	4.955644	-0.628225	-1.434997
Mg	0.243871	-0.835276	0.702879
N	-0.876993	0.963138	0.888280
N	-0.931904	-0.288959	-1.016220
C	-1.370122	0.748717	-0.315381
C	-1.420046	1.998553	1.725074
H	-0.619651	2.598815	2.195159
H	-2.026934	1.580319	2.552159
H	-2.070512	2.698607	1.163170
C	-1.475596	-0.538312	-2.326053
H	-1.914486	-1.550829	-2.403375
H	-0.704094	-0.469233	-3.120732
H	-2.271458	0.183014	-2.595656
H	-2.154252	1.419304	-0.734177
H	0.233207	-2.199925	1.789045

04_MgH
23

O	-5.889657	2.224931	-0.512700
C	-4.808325	2.694475	0.051225
O	-4.199774	1.766485	0.741736
C	-4.891021	0.479507	0.657947
C	-6.143121	0.833650	-0.136608
H	-5.084167	0.135380	1.681824
H	-4.219585	-0.225256	0.148921
H	-7.069814	0.813155	0.453361
H	-6.278806	0.256384	-1.059940
Mg	-4.349828	4.955003	0.208295
N	-6.367991	5.483399	-0.143477
N	-5.616780	4.901865	1.922108
C	-6.617557	5.265898	1.132210
H	-7.648722	5.379592	1.538955
C	-7.424081	5.826874	-1.054419
H	-7.226140	6.789435	-1.562614
H	-8.405117	5.913859	-0.545517
H	-7.535964	5.069872	-1.853615
C	-5.841909	4.674219	3.322323
H	-5.243546	5.355743	3.954851
H	-5.553939	3.647119	3.622250
H	-6.904455	4.816587	3.607097
H	-2.799733	5.543605	-0.262748

05_MgH
27

C	-3.459583	0.984286	-0.723011
C	-2.173856	0.174886	-0.520964
N	-2.499699	-0.548499	0.716186
O	-4.227459	0.735089	0.495157
C	-3.660210	-0.192829	1.250057
C	-1.627191	-1.577664	1.247304
H	-1.597778	-2.444211	0.567963
H	-2.005697	-1.895967	2.228983
H	-0.606235	-1.182483	1.369862
Mg	-4.626944	-1.493685	2.860042
H	-1.973147	-0.532189	-1.340431
H	-1.277179	0.801113	-0.377410
H	-3.299599	2.066873	-0.809293
H	-4.064760	0.629358	-1.568726

N	-5.046178	-2.886595	1.275110
N	-6.661459	-1.660855	2.306560
C	-6.318710	-2.545703	1.385544
H	-7.085742	-2.986891	0.709405
C	-4.609249	-3.754182	0.220120
H	-3.964360	-3.219428	-0.509464
H	-5.449693	-4.189800	-0.357512
H	-4.006213	-4.596126	0.608333
C	-8.041027	-1.301415	2.496360
H	-8.195251	-0.211915	2.382781
H	-8.397787	-1.568269	3.509386
H	-8.709610	-1.806819	1.772292
H	-3.655217	-1.628325	4.295748

06_MgH
25

Mg	-2.586391	0.740496	-2.014006
O	0.510417	0.670515	-2.404253
N	-2.011929	-1.305371	-2.461355
N	-2.288498	0.282611	-4.063033
C	-1.709083	-2.651977	-2.064913
C	-2.177347	0.757862	-5.416629
C	-1.959442	-0.948447	-3.742147
C	1.688622	-0.238107	-0.551203
C	0.182315	-0.045243	-0.263497
C	-0.401244	0.599888	-1.487455
C	1.779171	0.004045	-2.056674
H	2.585886	0.674369	-2.379805
H	1.814844	-0.916809	-2.657689
H	-0.360789	-0.992049	-0.099388
H	-0.036822	0.580240	0.616936
H	-2.602501	-3.192742	-1.697077
H	-0.981753	-2.676255	-1.228484
H	-1.270544	-3.254395	-2.886072
H	-1.442115	1.580029	-5.497199
H	-3.139449	1.163643	-5.779946
H	-1.859403	-0.035948	-6.120811
H	-1.625414	-1.672546	-4.518850
H	2.288417	0.500335	-0.000516
H	2.049540	-1.237190	-0.271901
H	-3.768820	1.591839	-1.091923

07_MgH
33

C	-1.035261	2.344950	-0.011124
N	-2.080952	1.591478	-0.394120
C	-2.016975	0.190549	-0.816015
C	-3.444686	2.043760	-0.120072
N	0.202835	1.978179	-0.417086
C	1.364257	2.561763	0.251341
C	0.527849	1.305159	-1.674789
H	1.073853	2.946401	1.236238
H	1.805119	3.382459	-0.339590
H	2.129236	1.780471	0.393071
H	0.858325	0.259615	-1.548338
H	1.351186	1.858040	-2.155118
H	-0.329772	1.332480	-2.356972
H	-4.065060	1.924373	-1.024248
H	-3.442324	3.103826	0.174522
H	-3.896838	1.438871	0.686041
H	-2.158889	0.053355	-1.901166
H	-2.820711	-0.359404	-0.302579
H	-1.063226	-0.256723	-0.513394
Mg	-1.297416	4.400918	1.017401
N	0.444323	5.621070	0.825628
N	-0.088596	4.581899	2.776655
C	0.658549	5.453360	2.116922
C	1.244990	6.540571	0.064767
H	0.613239	7.219453	-0.536202
H	1.911639	6.016457	-0.650969
H	1.892154	7.173308	0.705613
H	1.442510	6.040875	2.646572
C	0.074192	4.425540	4.197949
H	-0.856383	4.667061	4.745346
H	0.868001	5.082195	4.605704
H	0.341641	3.385791	4.468440
H	-2.918388	4.999704	0.750177

08_MgH
35

Mg	-2.214941	-0.120157	-1.926854
N	0.562018	0.116751	-0.246906
N	-2.524207	0.329517	-4.003541
N	-2.563827	-1.799463	-3.204057
N	1.026982	0.100756	-2.335481
C	-0.181271	0.102494	1.004896
C	0.876063	0.059054	-3.780874
C	2.832331	0.230524	0.871034
C	3.559433	0.215524	-2.382058
C	2.251277	0.164464	-1.669742
C	-0.019742	0.064493	-1.472421
C	1.952771	0.173940	-0.331337
C	-2.773409	-0.958562	-4.197189
C	-2.791543	1.283649	-5.047700
C	-2.897783	-3.191942	-3.335469
H	0.019105	-0.823314	1.567014
H	0.104953	0.961856	1.629944
H	-1.254363	0.164917	0.769741
H	1.241856	-0.898189	-4.184470
H	-0.190344	0.171728	-4.022060
H	1.443238	0.876917	-4.249095
H	2.662544	1.145904	1.463113
H	2.660346	-0.626937	1.542847
H	3.889687	0.216487	0.571719
H	3.649924	1.115742	-3.013610
H	4.387445	0.231897	-1.659904
H	3.704984	-0.658062	-3.039230
H	-3.133124	0.333103	-0.508222
H	-3.167224	-1.318366	-5.175757
H	-3.267268	0.818058	-5.934367
H	-3.465688	2.086394	-4.698349
H	-1.868166	1.786726	-5.402758
H	-3.213228	-3.459199	-4.364813
H	-2.042698	-3.841180	-3.069860
H	-3.723479	-3.478057	-2.657062

09_MgH

31

N	2.551530	-0.445371	1.086958
N	3.118276	0.007248	-0.852081
N	2.048737	-0.129286	-0.088304
C	3.920235	-0.518019	1.068377
C	4.323069	-0.207006	-0.233021
C	1.663360	-0.670328	2.212900
H	1.707804	-1.723365	2.529236
H	0.644497	-0.416700	1.899150
H	1.966894	-0.033723	3.055503
C	2.932082	0.365004	-2.253512
H	2.367127	1.304640	-2.324427
H	2.376606	-0.431292	-2.768436
H	3.931302	0.479019	-2.696539
C	4.733031	-0.875741	2.265713
H	4.663420	-0.111637	3.058513
H	5.784937	-0.963438	1.955735
H	4.416734	-1.838488	2.702158
Mg	6.403540	0.062094	-0.993797
N	6.602859	2.114932	-1.577759
N	6.254047	0.461155	-3.102846
C	6.530073	1.731747	-2.836260
H	6.713703	2.455123	-3.664019
C	6.298796	-0.022151	-4.458060
H	7.099934	-0.770905	-4.604635
H	6.477934	0.788409	-5.193245
H	5.354183	-0.524776	-4.746882
C	6.918486	3.475869	-1.237369
H	7.795246	3.528127	-0.565780
H	6.086879	3.972212	-0.700380
H	7.151335	4.095582	-2.126631
H	7.531562	-0.879967	-0.045993

50_MgH
48

P	0.807148	0.155372	-0.599143
Mg	3.007565	-0.630777	0.807488
N	2.519661	-2.674801	0.844237
N	1.564371	-1.184891	2.272951
C	-0.078986	-2.438799	-0.993152
C	-0.870758	-3.560691	-0.754858
C	-2.002993	-3.458206	0.062419
C	-2.348356	-2.224130	0.617989
C	-1.558107	-1.094959	0.374533
C	1.790394	1.842918	-4.199051
C	0.811910	1.375687	-5.081740
C	-0.180956	0.503528	-4.623973
C	-0.207098	0.107588	-3.282251
C	1.778044	1.434740	-2.862935
C	-1.148529	3.857210	1.416856
C	-0.042837	3.228991	1.996156
C	-1.666985	3.379110	0.206776
C	-1.086087	2.273102	-0.414216
C	0.534959	2.115023	1.377811
C	0.770025	0.575465	-2.389925
C	0.015765	1.626091	0.171081
C	-0.413088	-1.195018	-0.426281
C	2.695216	-4.021733	0.364939
C	0.608971	-0.867750	3.301735
C	1.658632	-2.421272	1.817322
H	-3.234862	-2.137087	1.251264
H	-1.825976	-0.136461	0.822998
H	-2.614579	-4.339580	0.269023
H	-0.588208	-4.519411	-1.198338
H	0.817463	-2.530746	-1.608552
H	-0.986524	-0.572216	-2.930973
H	0.821045	1.691256	-6.128623
H	-0.943724	0.129239	-5.311451
H	2.554035	1.794135	-2.180443
H	2.572965	2.519544	-4.551510
H	-2.528641	3.870950	-0.251853
H	-1.488414	1.906374	-1.361898
H	1.392016	1.622289	1.840108
H	-1.606210	4.722950	1.902304
H	0.383407	3.610384	2.927704
H	3.704393	-4.410753	0.596547
H	2.578470	-4.080347	-0.733838
H	1.960538	-4.722816	0.805209
H	0.086754	-1.765865	3.683795
H	-0.175984	-0.178627	2.938045
H	1.094591	-0.371128	4.161481
H	1.016352	-3.228408	2.229425
H	4.353717	0.394978	0.525470

Cu Radicals
01_Cu(NMe_3)
41

Cu	-2.536848	-2.013423	0.765790
N	-0.879265	0.401229	0.794516
N	-4.089558	-3.247459	1.113221
C	0.157582	1.050179	-0.035902
C	-1.038107	-0.950637	0.474564
C	0.224295	-1.423355	-0.238649
C	1.105833	-0.143814	-0.286358
C	0.869574	2.178556	0.718075
C	-0.440856	1.602762	-1.345381
C	0.917460	-2.551921	0.550603
C	-0.096738	-1.962184	-1.648433
C	-2.019332	1.148497	1.253442
C	-3.615226	-4.322958	2.000421
C	-4.575147	-3.802335	-0.161332
C	-5.150448	-2.465100	1.767405
H	1.664537	-0.035922	-1.229443
H	1.844089	-0.179357	0.530445
H	0.195894	3.027590	0.915950
H	1.260672	1.813215	1.680622
H	1.713141	2.563486	0.122541
H	0.350839	1.976446	-2.014684
H	-1.007481	0.822288	-1.873319
H	-1.129126	2.439471	-1.143316
H	1.837535	-2.901991	0.047536
H	1.180563	-2.217581	1.566805
H	0.234745	-3.412494	0.651240
H	-0.799118	-2.809193	-1.572118
H	-0.578128	-1.197449	-2.274775
H	0.813628	-2.317992	-2.164780
H	-1.722810	2.140016	1.630478

H	-2.797292	1.290266	0.468732
H	-2.496832	0.597621	2.080747
H	-3.761497	-4.345131	-0.661339
H	-4.900795	-2.983883	-0.817873
H	-5.424665	-4.495975	0.000850
H	-4.768155	-2.030689	2.702079
H	-6.031273	-3.097704	1.996474
H	-5.461902	-1.643532	1.107446
H	-2.812744	-4.882685	1.500732
H	-4.436085	-5.021648	2.260098
H	-3.207044	-3.886997	2.922924

02_Cu(NMe$_3$)
29

N	2.673103	-0.707528	0.854916
N	3.073131	1.011463	-0.400645
C	2.034459	0.220894	0.053712
C	4.051629	-0.549215	0.817993
C	4.302363	0.531876	0.027785
C	1.950621	-1.776082	1.496353
H	1.456350	-2.415130	0.737309
H	1.166211	-1.364011	2.151298
H	2.634290	-2.390257	2.099805
C	2.848676	2.089453	-1.330169
H	2.199950	2.858208	-0.881314
H	2.338498	1.700409	-2.234150
H	3.804519	2.551097	-1.616877
Cu	0.328550	-0.020264	-0.804384
H	5.241890	1.004377	-0.248294
H	4.731460	-1.197037	1.365912
C	-2.399227	0.877800	-1.602132
H	-1.042663	2.430367	0.059009
N	-1.675337	0.476134	-0.393526
C	-1.557791	1.592394	0.550609
H	-2.548690	1.940509	0.912284
C	-2.291401	-0.693040	0.239035
H	-3.325424	-0.481815	0.584338
H	-1.687833	-1.002597	1.104591
H	-2.312369	-1.527304	-0.476940
H	-0.941854	1.283109	1.405941
H	-3.433160	1.211900	-1.370493
H	-2.435689	0.033753	-2.305060
H	-1.861446	1.702191	-2.092910

03_Cu(NMe$_3$)
31

N	2.976483	0.690501	1.070432
N	3.148141	-0.002562	-1.014739
C	2.227680	0.377930	-0.056650
C	4.256646	-0.010252	1.049644
C	4.458900	-0.304053	-0.450435
C	2.327329	0.914972	2.331846
H	1.950125	-0.025785	2.796542
H	1.462328	1.576425	2.175421
H	3.015652	1.407239	3.038845
C	2.771567	-0.517834	-2.296370
H	3.540166	-0.283434	-3.053373
H	1.829415	-0.043346	-2.607227
H	2.612273	-1.620425	-2.294000
H	4.201425	-0.957796	1.630689
H	5.063178	0.600819	1.487989
H	5.247981	0.321355	-0.908370
H	4.742453	-1.362831	-0.619569
Cu	0.379958	-0.055907	-0.045541
N	-1.590066	0.468245	-0.347535
C	-2.407669	-0.672909	-0.777356
H	-2.427657	-1.430911	0.017957
H	-1.957371	-1.129029	-1.670583
H	-3.446218	-0.361118	-1.016011
C	-1.520488	1.490926	-1.401897
H	-0.833124	2.288950	-1.089705
H	-2.518289	1.925411	-1.618996
H	-1.120868	1.040465	-2.321898
C	-2.099686	1.047512	0.901705
H	-2.082188	0.286261	1.694796
H	-3.136144	1.426125	0.784326
H	-1.454137	1.882371	1.208838

04_Cu(NMe$_3$)
23

O	-6.271405	1.585559	-0.480366
C	-5.615617	2.450647	0.396653
O	-4.489330	1.792372	0.885813
C	-4.297207	0.601033	0.139891
C	-5.713016	0.291520	-0.343279
H	-3.864480	-0.173418	0.791308
H	-3.610952	0.778224	-0.713829
H	-6.272631	-0.301684	0.408207
H	-5.758576	-0.229348	-1.312128
Cu	-5.943202	4.282223	0.436737
N	-6.457951	6.215138	0.605916
C	-7.917183	6.279510	0.796158
H	-8.198852	5.712584	1.693923
H	-8.423495	5.830341	-0.069107
H	-8.258493	7.328244	0.908135
C	-6.072190	6.921186	-0.628119
H	-4.985575	6.846309	-0.770452
H	-6.362431	7.990878	-0.585298
H	-6.566520	6.451508	-1.489499
C	-5.766685	6.786357	1.774931
H	-6.055465	6.230991	2.678149
H	-6.027213	7.855213	1.910319
H	-4.678965	6.697908	1.645940

05_Cu(NMe$_3$)
27

C	-2.971937	-0.145083	-1.397039
C	-2.142564	-0.253780	-0.119881
N	-3.172699	0.006644	0.872386
O	-4.276483	-0.557027	-0.995491
C	-4.383361	-0.529848	0.390042
C	-2.837384	-0.194431	2.253576
H	-2.633184	-1.266385	2.491218
H	-3.678152	0.131220	2.885485
H	-1.953018	0.403474	2.525733
Cu	-5.560306	-1.671623	1.308295

H	-1.711765	-1.276688	-0.007520
H	-1.317768	0.475585	-0.070439
H	-3.000224	0.897048	-1.770039
H	-2.612791	-0.798021	-2.209495
N	-7.013072	-2.644191	2.339419
C	-6.420170	-3.162740	3.583498
H	-6.006580	-2.331345	4.171953
H	-5.600009	-3.851737	3.339010
H	-7.175842	-3.696632	4.194470
C	-8.081844	-1.677879	2.642559
H	-8.492165	-1.273375	1.706595
H	-7.669761	-0.844138	3.227240
H	-8.900246	-2.152202	3.221280
C	-7.537553	-3.751382	1.522907
H	-6.719247	-4.436764	1.262146
H	-7.966235	-3.353203	0.592581
H	-8.322392	-4.311194	2.070279

06_Cu(NMe_3)
25

Cu	2.576093	0.316984	-1.781969
O	1.214591	-0.055575	0.708923
N	4.149740	0.658752	-3.000032
C	5.173206	1.367151	-2.212058
C	4.690520	-0.616706	-3.500802
C	3.691179	1.500413	-4.118765
C	-1.094792	0.164132	0.293542
C	-0.364639	-0.116943	-1.034058
C	1.093444	0.069368	-0.678244
C	-0.053598	-0.302096	1.311899
H	3.244803	2.424784	-3.726287
H	4.531455	1.762571	-4.792851
H	2.925501	0.963228	-4.695143
H	5.486152	0.741212	-1.365053
H	6.061055	1.602260	-2.832745
H	4.754865	2.302134	-1.813804
H	5.555507	-0.447065	-4.173205
H	5.014881	-1.236750	-2.653734
H	3.910857	-1.159955	-4.051720
H	-1.283244	1.244561	0.407592
H	-2.054605	-0.364864	0.399075
H	-0.585071	-1.153471	-1.376579
H	-0.687668	0.547917	-1.853138
H	-0.163326	-1.385132	1.525421
H	-0.099932	0.237847	2.271811

07_Cu(NMe_3)
33

C	-0.672256	2.452090	0.058734
N	-1.693364	2.230359	-0.893908
C	-2.485802	1.022579	-0.706188
C	-2.489974	3.383615	-1.225986
N	0.257441	1.378832	0.097812
C	1.079506	1.336924	1.280266
C	1.013408	1.177063	-1.131085
H	0.441321	1.348367	2.177122
H	1.779547	2.207811	1.352452
H	1.691725	0.419472	1.293897
H	1.527820	0.200391	-1.121820
H	1.779894	1.971531	-1.284372
H	0.322677	1.210367	-1.984562
H	-3.142267	3.165855	-2.088224
H	-1.830969	4.226274	-1.486501
H	-3.144250	3.714484	-0.382907
H	-3.019268	0.753175	-1.634057
H	-3.241284	1.142488	0.103622
H	-1.815556	0.200437	-0.421956
Cu	-0.463837	4.030004	1.053393
N	-0.147881	5.722418	2.112564
C	0.733590	6.581248	1.304664
H	0.242032	6.819706	0.351238
H	1.668602	6.047818	1.083820
H	0.970907	7.524703	1.837470
C	0.510347	5.363068	3.379628
H	-0.156248	4.725330	3.975855
H	0.769614	6.266829	3.967100
H	1.430711	4.800047	3.169319
C	-1.431201	6.398167	2.361780
H	-1.915944	6.630010	1.402811
H	-1.285177	7.340019	2.927688
H	-2.094858	5.736499	2.934685

08_Cu(NMe_3)
35

Cu	2.606196	-1.561202	1.686510
N	0.373567	0.316569	1.273917
N	0.328927	-1.322213	-0.151588
N	3.825155	-2.800654	2.837901
C	-0.372569	0.757754	0.177391
C	0.901591	-0.949469	1.057471
C	-0.400700	-0.274288	-0.720997
C	-1.007083	2.105250	0.112089
C	-1.070751	-0.378553	-2.049144
C	0.664384	-2.570703	-0.781277
C	0.776169	1.101741	2.409256
C	5.031856	-2.089228	3.275353
C	4.180551	-3.950097	1.997977
C	3.009817	-3.217387	3.984794
H	-0.257690	2.916696	0.115757
H	-1.601667	2.208959	-0.806246
H	-1.685730	2.286675	0.964942
H	-1.631168	0.539590	-2.274201
H	-0.344214	-0.531521	-2.866761
H	-1.787662	-1.218389	-2.090760
H	0.016511	-2.758398	-1.647507
H	1.725445	-2.579044	-1.113352
H	0.535934	-3.393177	-0.060078
H	0.180230	2.020943	2.481866
H	0.635731	0.516098	3.331045
H	1.853896	1.367055	2.342211
H	2.718217	-2.331410	4.567689
H	3.557712	-3.919497	4.646646
H	2.092488	-3.702397	3.622676
H	4.730192	-3.596117	1.113170
H	3.266443	-4.455765	1.653652
H	4.809430	-4.677891	2.550975
H	5.580803	-1.726904	2.394085

H	5.691826	-2.746794	3.878934
H	4.747033	-1.215222	3.878380

09_Cu(NMe$_3$)
31

N	3.081538	-0.588725	1.034471
N	3.394955	-1.029964	-1.054713
N	2.483196	-1.235047	-0.035348
C	4.171698	0.183449	0.596337
C	4.383332	-0.090387	-0.727538
C	2.186644	-0.211941	2.097255
H	2.763731	0.151625	2.960028
H	1.606814	-1.093769	2.410299
H	1.467925	0.575214	1.784509
C	2.932059	-1.315373	-2.381467
H	3.794196	-1.332511	-3.065011
H	2.201297	-0.563804	-2.750327
H	2.439320	-2.300706	-2.398585
C	4.977532	1.009457	1.541493
H	4.388577	1.809023	2.026453
H	5.805636	1.484894	0.995559
H	5.412235	0.396614	2.354532
Cu	5.699837	0.608110	-1.878449
N	7.103569	1.410710	-3.073434
C	7.679939	2.585071	-2.392917
H	8.142816	2.272258	-1.446660
H	6.885876	3.310893	-2.169806
H	8.448561	3.073535	-3.023480
C	8.146697	0.401274	-3.328295
H	7.700132	-0.476121	-3.816318
H	8.594322	0.079141	-2.377632
H	8.942936	0.808420	-3.982297
C	6.461071	1.813746	-4.338166
H	5.669140	2.547294	-4.134676
H	6.005439	0.936077	-4.818097
H	7.197256	2.263623	-5.033132

50_Cu(NMe$_3$)
48

Cu	3.180212	3.272360	-2.366267
P	1.539523	2.218012	-1.206568
N	4.399650	1.979672	-3.558911
C	5.460014	2.724614	-4.237823
C	4.960061	0.998758	-2.626663
C	3.503669	1.333861	-4.523917
C	0.837533	0.744740	-2.016594
C	0.068614	3.245100	-0.815402
C	2.144221	1.648195	0.424352
C	1.492509	0.658426	1.182843
C	3.293005	2.262965	0.952060
C	3.788722	1.886966	2.202599
C	3.143149	0.891284	2.942933
C	1.994330	0.278193	2.427703
C	1.384966	-0.544983	-1.839460
C	-0.082713	0.939240	-3.073079
C	-0.461431	-0.122425	-3.894122
C	0.068482	-1.403225	-3.691279
C	0.996776	-1.603919	-2.658812
C	0.179385	4.635311	-0.945479
C	-1.128863	2.687289	-0.335598
C	-0.892837	5.462956	-0.601225
C	-2.080317	4.905202	-0.120132
C	-2.197574	3.514705	0.008826
H	3.016796	2.101369	-5.144326
H	4.056625	0.636962	-5.187182
H	2.725069	0.772472	-3.990821
H	6.085722	3.239588	-3.494292
H	6.102631	2.053180	-4.846155
H	5.011765	3.484381	-4.893776
H	5.609796	0.265040	-3.147468
H	5.555981	1.517212	-1.860180
H	4.146500	0.456447	-2.126427
H	-1.224601	1.603222	-0.236676
H	1.118972	5.052081	-1.325089
H	-0.797042	6.546540	-0.711225
H	-2.917017	5.551595	0.157015
H	-3.124602	3.074080	0.385840
H	-0.505981	1.932110	-3.244929
H	-1.183069	0.051691	-4.696876
H	-0.237538	-2.234343	-4.330779
H	1.420187	-2.596994	-2.482910
H	2.111496	-0.722545	-1.043519
H	1.480183	-0.493871	3.006365
H	0.593923	0.177572	0.791183
H	4.680347	2.377218	2.602547
H	3.533396	0.589916	3.918718
H	3.788930	3.045062	0.368211

CuH Adducts
01_CuH
29

C	0.076558	1.148441	-0.556910
N	-0.974205	0.482871	0.290143
C	-0.698313	-0.718883	0.734872
C	0.704248	-1.089300	0.292538
C	1.284894	0.220135	-0.306405
C	0.333329	2.582560	-0.085745
C	-0.380536	1.142011	-2.023635
C	1.500802	-1.594717	1.505896
C	0.588977	-2.220868	-0.748315
C	-2.254520	1.139784	0.529171
Cu	-1.840248	-1.915575	1.720016
H	1.859064	0.047442	-1.228111
H	1.966336	0.693650	0.416169
H	-0.542993	3.230789	-0.241101
H	0.605081	2.605490	0.981016
H	1.168001	3.013080	-0.659549
H	0.408264	1.570452	-2.660443
H	-0.589537	0.119847	-2.372032
H	-1.287470	1.748284	-2.171007
H	2.522782	-1.881479	1.206755
H	1.570963	-0.822006	2.287455
H	1.004752	-2.472755	1.947179
H	0.096817	-3.098885	-0.303498
H	-0.002621	-1.914263	-1.624433
H	1.591176	-2.518032	-1.098196
H	-2.127925	2.009657	1.191178

H	-2.700796	1.482130	-0.417013
H	-2.923279	0.414576	1.009485
H	-2.747781	-2.900397	2.470980

02_CuH
17

N	2.938057	-0.486870	0.950652
N	2.954541	0.454224	-0.977827
C	2.107384	-0.035484	-0.030160
C	4.269910	-0.285875	0.624199
C	4.280398	0.314800	-0.598457
C	2.469117	-1.105896	2.178978
H	1.374018	-1.029939	2.195324
H	2.884887	-0.584323	3.054068
H	2.761172	-2.167567	2.217921
C	2.507708	1.056997	-2.222525
H	1.418649	0.932001	-2.283348
H	2.981665	0.558661	-3.081411
H	2.752530	2.130965	-2.246953
Cu	0.165618	-0.072695	-0.057402
H	-1.364179	-0.100590	-0.071621
H	5.106528	0.652545	-1.218657
H	5.085557	-0.585721	1.276941

03_CuH
19

N	3.026287	-0.503377	0.982346
N	3.017228	0.475797	-0.954119
C	2.228099	-0.012403	0.018513
C	4.449843	-0.456322	0.635651
C	4.444375	0.423348	-0.623678
C	2.568978	-1.219172	2.148451
H	2.832279	-2.292058	2.089602
H	1.476504	-1.122069	2.209331
H	3.022038	-0.802797	3.064394
C	2.549210	1.192804	-2.115201
H	2.817972	2.264540	-2.060140
H	1.455648	1.100515	-2.162861
H	2.989478	0.773768	-3.036167
H	4.827762	-1.476340	0.435194
H	5.048494	-0.032674	1.457745
H	4.828467	1.441901	-0.427669
H	5.031974	-0.002627	-1.452497
Cu	0.281758	-0.009185	0.025286
H	-1.249123	-0.006025	0.028549

04_CuH
11

O	-6.376130	1.678483	0.394669
C	-5.483553	2.641657	0.366447
O	-4.288496	2.162286	0.109680
C	-4.309015	0.716751	-0.076738
C	-5.781879	0.373594	0.137003
H	-3.631190	0.272829	0.663533
H	-3.938894	0.501534	-1.088551
H	-5.975891	-0.263169	1.010684
H	-6.276682	-0.054400	-0.744974
Cu	-5.896501	4.493647	0.634119
H	-6.240402	5.971892	0.820374

05_CuH
15

C	-3.041660	0.940230	-1.031405
C	-2.031015	0.559452	0.059166
N	-2.837184	-0.349840	0.876212
O	-4.246026	0.208431	-0.677186
C	-4.076411	-0.521584	0.420267
C	-2.285003	-1.019803	2.035095
H	-1.469365	-1.699726	1.737315
H	-3.084692	-1.603389	2.511534
H	-1.886648	-0.283923	2.753364
Cu	-5.431821	-1.673859	1.174939
H	-1.140923	0.039898	-0.333420
H	-1.689446	1.420345	0.658124
H	-3.289275	2.011334	-1.048456
H	-2.733878	0.623654	-2.038613
H	-6.461154	-2.612968	1.803410

06_CuH
13

C	-0.433667	-0.363094	1.283511
O	0.748841	0.328546	0.742272
C	0.703163	0.539511	-0.538194
C	-0.585290	-0.024149	-1.073789
C	-1.149951	-0.909190	0.052447
Cu	2.029323	1.473522	-1.529062
H	-0.061822	-1.118271	1.988026
H	-1.009740	0.394218	1.837287
H	-1.224154	0.851815	-1.300584
H	-0.428073	-0.536604	-2.034719
H	-2.242772	-0.842343	0.143144
H	-0.883244	-1.963335	-0.118358
H	3.094386	2.200716	-2.362032

07_CuH
21

C	-0.643444	2.551513	0.055375
N	-1.850421	2.089761	-0.341710
C	-2.195898	0.702283	-0.641434
C	-3.012301	2.955369	-0.174052
N	0.464752	1.866125	-0.303354
C	1.735620	2.220462	0.319742
C	0.601670	0.994275	-1.467512
H	1.552885	2.927932	1.139364
H	2.419330	2.685974	-0.412241
H	2.225205	1.320271	0.730117
H	0.674510	-0.075802	-1.206075
H	1.524993	1.271060	-2.001819
H	-0.236466	1.143467	-2.157638
H	-3.586466	3.007925	-1.114878
H	-2.675449	3.965566	0.095468
H	-3.680121	2.573954	0.618828
H	-2.382400	0.518030	-1.713591
H	-3.119553	0.452206	-0.094828

H	-1.410740	0.023627	-0.290610
Cu	-0.497835	4.180909	1.145153
H	-0.372159	5.434039	2.012581

08_CuH
23

C	-0.730576	0.793822	0.044688
N	-0.084992	0.360979	1.203433
C	0.802772	-0.636801	0.950620
N	0.704874	-0.827604	-0.391928
C	-0.226244	0.032403	-0.976212
C	-1.738911	1.889869	0.044834
C	-0.532572	0.024459	-2.434683
C	1.506725	-1.792628	-1.119113
C	-0.326479	0.888688	2.532695
Cu	1.954539	-1.541071	2.227725
H	-1.301465	2.843309	0.387771
H	-2.135953	2.050883	-0.967201
H	-2.593201	1.662084	0.703894
H	-1.332622	0.741377	-2.663293
H	0.344968	0.300645	-3.043635
H	-0.868936	-0.969073	-2.776460
H	0.866984	-2.512367	-1.653393
H	2.161267	-1.286123	-1.846095
H	2.126307	-2.330840	-0.389707
H	-1.370844	0.716288	2.837979
H	0.340949	0.364077	3.227898
H	-0.110128	1.968187	2.572241
H	2.861799	-2.229782	3.247162

09_CuH
19

N	2.958832	-0.421659	0.919522
N	2.973965	0.547039	-0.910191
N	2.150959	0.115809	0.030654
C	4.274455	-0.335424	0.551073
C	4.312373	0.319738	-0.685584
C	2.407760	-0.988961	2.138098
H	2.626155	-2.066141	2.188622
H	1.323571	-0.827369	2.127755
H	2.849923	-0.490714	3.012629
C	2.404580	1.195090	-2.083086
H	3.222514	1.716575	-2.594839
H	1.627968	1.904857	-1.769700
H	1.966295	0.443442	-2.756662
C	5.393322	-0.857881	1.382072
H	5.406199	-0.407458	2.388968
H	6.337653	-0.607514	0.881207
H	5.346093	-1.953771	1.503226
Cu	5.804446	0.889652	-1.782916
H	6.987765	1.369587	-2.625077

50_CuH
36

Cu	-3.358863	-2.445561	0.150958
P	-1.620277	-1.029428	0.259518
C	2.269891	-3.236204	-0.957982
C	1.506584	-3.664608	0.135225
C	1.873564	-2.111957	-1.685812
C	0.717811	-1.410865	-1.325068
C	0.347026	-2.975463	0.490216
C	-1.913704	2.627706	-2.572152
C	-1.074966	2.700465	-1.453410
C	-2.665394	1.472288	-2.807198
C	-2.578100	0.390974	-1.925970
C	-0.982590	1.619737	-0.573947
C	-0.847949	0.511298	4.561956
C	0.236988	0.363702	3.692346
C	-2.140971	0.206599	4.125828
C	0.031442	-0.088691	2.386766
C	-2.349264	-0.251772	2.823270
C	-1.729480	0.454509	-0.808961
C	-1.264843	-0.398685	1.943713
C	-0.049387	-1.836183	-0.232675
H	-3.326001	1.411578	-3.675500
H	-3.172010	-0.510687	-2.100244
H	-1.988942	3.476486	-3.256725
H	-0.493016	3.605056	-1.259138
H	-0.331953	1.686063	0.300564
H	-3.355133	-0.516134	2.485050
H	-2.989937	0.318769	4.804294
H	-0.683302	0.865283	5.582877
H	1.247370	0.600712	4.036078
H	0.883299	-0.219520	1.716174
H	3.170945	-3.783846	-1.245115
H	1.812774	-4.544344	0.706762
H	-0.258421	-3.321754	1.332683
H	2.466154	-1.773361	-2.539271
H	0.413344	-0.532421	-1.896476
H	-4.528088	-3.426645	0.084282

10.) Author Contributions

K.B. performed half of the calculations, contributed to deriving equations 2–4, performed the analysis of the relation with the *RSS*, and contributed to the composition of the manuscript. D.M. developed the idea, performed the other half of the calculations, and composed the manuscript. H.B. contributed to the analysis of the computational data. R.W. suggested to explore boryl radicals and contributed to the discussion of the results.

We thank S. Huber for helpful discussions and J. Messelberger for exploratory calculations.

11.) References

[1] a) F. Neese, *WIREs Comput. Mol. Sci.* **2012**, *2*, 73–78; b) F. Neese, *WIREs Comput. Mol. Sci.* **2018**, *8*, e1327.
[2] a) C. T. Lee, W. T. Yang, R. G. Parr, *Phys. Rev. B: Condens. Matter* **1988**, *37*, 785–789; b) A. D. Becke, J. *Chem. Phys.* **1993**, *98*, 5648–5652; c) F. Weigend, R. Ahlrichs, Phys. Chem. Chem. Phys. **2005**, *7*, 3297–3305.
[3] a) F. Neese, F. Wennmohs, A. Hansen, U. Becker, *Chem. Phys.* **2009**, *356*, 98–109; b) R. Izsák, F. Neese, J. *Chem. Phys.* **2011**, *135*, 144105; c) F. Weigend, *J. Comput. Chem.* **2008**, *29*, 167–175.
[4] M. L. Coote, C. Y. Lin, A. L. J. Beckwith, A. A. Zavitsas, *Phys. Chem. Chem. Phys.* **2010**, *12*, 9597–9610.
[5] S. Grimme, A. Hansen, S. Ehlert, J.-M. Mewes, *J. Chem. Phys.* **2021**, *154*, 064103.
[6] a) Y. Zhao, D. G. Truhlar, *J. Phys. Chem. A* **2005**, *109*, 5656–5667; b) E. Caldeweyher, S. Ehlert, A. Hansen, H. Neugebauer, S. Spicher, C. Bannwarth, S. Grimme, *J. Chem. Phys.* **2019**, *150*, 154122.
[7] a) S. Grimme, J. Antony, S. Ehrlich, H. Krieg, *J. Chem. Phys.* **2010**, *132*, 154104; b) S. Grimme, S. Ehrlich, L. Goerigk, *J. Comput. Chem.* **2011**, *32*, 1456–1465.
[8] a) C. Riplinger, F. Neese, *J. Chem. Phys.* **2013**, *138*, 034106; b) C. Riplinger, B. Sandhoefer, A. Hansen, F. Neese, *J. Chem. Phys.* **2013**, *139*, 134101; c) F. Neese, A. Hansen, F. Wennmohs, S. Grimme, *Acc. Chem. Res.* **2009**, *42*, 641–648; d) A. Hansen, D. G. Liakos, F. Neese, *J. Chem. Phys.* **2011**, *135*, 214102; e) D. G. Liakos, A. Hansen, F. Neese, *J. Chem. Theory Comput.* **2011**, *7*, 76–87; f) F. Neese, A. Hansen, D. G. Liakos, *J. Chem. Phys.* **2009**, *131*, 064103.
[9] Y.-R. Luo, Comprehensive Handbook of Chemical Bond Energies, CRC Press, Boca Raton, **2007**.
[10] a) R. Stowasser, R. Hoffmann, *J. Am. Chem. Soc.* **1999**, *121*, 3414–3420; b) R. van Meer, O. V. Gritsenko, E. J. Baerends, *J. Chem. Theory Comput.* **2014**, *10*, 4432–4441; c) P. Geerlings, F. De Proft, W. Langenaeker, *Chem. Rev.* **2003**, *103*, 1793–1874; d) E. J. Baerends, O. V. Gritsenko, R. van Meer, *Phys. Chem. Chem. Phys.* **2013**, *15*, 16408–16425.
[11] L. Salem, *J. Am. Chem. Soc.* **1968**, *90*, 543–552.
[12] Shree Sowndarya S. V., P. C. St. John, R. S. Paton, *Chem. Sci.* **2021**, *12*, 13158–13166.
[13] L. Falivene, Z. Cao, A. Petta, L. Serra, A. Poater, R. Oliva, V. Scarano, L. Cavallo, *Nat. Chem.* **2019**, *11*, 872–879.
